# The neuropeptide complement of the marine annelid *Platynereis dumerilii*

**DOI:** 10.1186/1471-2164-14-906

**Published:** 2013-12-20

**Authors:** Markus Conzelmann, Elizabeth A Williams, Karsten Krug, Mirita Franz-Wachtel, Boris Macek, Gáspár Jékely

**Affiliations:** 1 Max Planck Institute for Developmental Biology, Spemannstrasse 35, 72076, Tübingen, Germany; 2 Proteome Center Tübingen, University of Tübingen, Auf der Morgenstelle 15, 72076, Tübingen, Germany

**Keywords:** (3–10), Transcriptomics, Peptidomics, Lophotrochozoa, Neurobiology, Diuretic hormone, Allatostatin, Allatotropin, Neuroendocrinology, Proenkephalin

## Abstract

**Background:**

The marine annelid *Platynereis dumerilii* is emerging as a powerful lophotrochozoan experimental model for evolutionary developmental biology (evo-devo) and neurobiology. Recent studies revealed the presence of conserved neuropeptidergic signaling in *Platynereis*, including vasotocin/neurophysin, myoinhibitory peptide and opioid peptidergic systems. Despite these advances, comprehensive peptidome resources have yet to be reported.

**Results:**

The present work describes the neuropeptidome of *Platynereis*. We established a large transcriptome resource, consisting of stage-specific next-generation sequencing datasets and 77,419 expressed sequence tags. Using this information and a combination of bioinformatic searches and mass spectrometry analyses, we increased the known proneuropeptide (pNP) complement of *Platynereis* to 98. Based on sequence homology to metazoan pNPs, *Platynereis* pNPs were grouped into ancient eumetazoan, bilaterian, protostome, lophotrochozoan, and annelid families, and pNPs only found in *Platynereis*. Compared to the planarian *Schmidtea mediterranea*, the only other lophotrochozoan with a large-scale pNP resource, *Platynereis* has a remarkably full complement of conserved pNPs, with 53 pNPs belonging to ancient eumetazoan or bilaterian families. Our comprehensive search strategy, combined with analyses of sequence conservation, also allowed us to define several novel lophotrochozoan and annelid pNP families. The stage-specific transcriptome datasets also allowed us to map changes in pNP expression throughout the *Platynereis* life cycle.

**Conclusion:**

The large repertoire of conserved pNPs in *Platynereis* highlights the usefulness of annelids in comparative neuroendocrinology. This work establishes a reference dataset for comparative peptidomics in lophotrochozoans and provides the basis for future studies of *Platynereis* peptidergic signaling.

## Background

Neuropeptides, including peptide transmitters and hormones, are a diverse group of signaling molecules involved in chemical communication among neurons and neuroendocrine regulation. Neuropeptides represent by far the largest group of neurotransmitters and neuromodulators [1] and are considered the oldest neuronal signaling molecules in metazoans [2]. Peptidergic signaling has deep evolutionary origins: essential enzymes for proneuropeptide (pNP) processing, maturation, and secretion have even been identified in organisms without a nervous system, such as the sponge *Amphimedon queenslandica*[3] and the placozoan *Trichoplax adhaerens*[4].

pNPs are translated as inactive precursors typically consisting of an N-terminal signal peptide (SP) that directs the pNP to the secretory apparatus, and one or several peptide elements flanked by basic cleavage sites [5]. After pNP cleavage by neuronal prohormone convertases, the liberated peptides can be further modified. C-terminal alpha-amidation and N-terminal pyroglutamination are common forms of modification [6] and can affect peptide stability [7], peptide structure [8,9], and bioactivity [10,11].

Structurally and functionally important peptide elements often show sequence conservation among homologous pNPs or between the mature peptides within a single pNP [12]. The strongest conservation is often restricted to a few key residues that confer bioactivity to the processed peptides [13,14]. pNPs often contain spacer sequences between the conserved peptide stretches. These spacers are less conserved and therefore considered to be biologically inactive [12].

Most neuropeptides signal via G-protein coupled receptors (GPCR) [15]. It has become clear in recent years that GPCRs and their peptide ligands are stably associated in evolution [16-18]. This co-evolutionary relationship of peptide-ligand pairs has been exploited to establish several conserved metazoan pNP families over large evolutionary distances [19,20].

The classical approach to identifying novel bioactive neuropeptides was biochemical purification from the species of interest, followed by functional analysis [21-24]. With increasing genomic and transcriptomic sampling, pNP identification has been accelerated by *in silico* sequence analyses based on homology to previously described pNPs [25,26], or on the presence of sequence features such as a SP, conserved C-terminal amidated motifs (e.g., RFa, “a” for amide) or cleavage sites [26-30].

Mass-spectrometry (MS) is also widely used as a powerful tool for the direct identification of bioactive peptides. This method relies on mapping the obtained peptide masses to a reference dataset (genome or transcriptome), and can be impeded by the presence of extensive post-translational modifications [31-33]. A combination of genomics and MS approaches has revealed the complete neuropeptide repertoire of several species in many metazoan phyla [34-38].

Annelids represent a diverse and species-rich phylum and have long been used in neuroendocrinological and behavioral studies [39]. Comparative genomic approaches [19,20,40] and other studies identified multiple annelid pNPs and neuropeptides, including RFa [13,41-47], FVRIa [48-50], excitatory peptide (EP) [51-53], egg-laying hormone (ELH) [54], myomodulin [55-57], RGWa [13], L11 or elevenin [28], vasopressin [39,58,59], gonadotropin releasing hormone (GnRH) [60,61], insulin-related peptides [62], neuropeptide Y (NPY) [63,64] and myoinhibitory peptide (MIP) [65]. Despite these advances, a complete picture of annelid neuropeptide diversity is still missing.

Here we describe the neuropeptide complement of the marine polychaete annelid, *Platynereis dumerilii*, using a combination of transcriptomics, *in silico* pNP searches and MS-based peptide identification. *Platynereis* has recently been established as a powerful experimental organism for comparative and experimental neurobiology [50,58,65,66]. *Platynereis* has a biphasic life cycle including free-swimming planktonic larval stages, followed by settlement and metamorphosis into the adult bottom-dwelling worm [67,68]. The *Platynereis* larval nervous system is highly peptidergic and several neuropeptides were shown to be involved in the regulation of larval behaviors such as ciliary swimming and larval settlement [28,65]. Previous studies described 15 *Platynereis* pNPs that are expressed in specific neuronal populations. Peptides generated from these pNPs include various RFa/RYa related peptides [28], vasotocin/neurophysin [58], FVRIa [50], RGWa [13], and MIP/allatostatin-B [65].

To complement this list, we used an integrative approach and identified 98 *Platynereis* pNPs, most of them verified by MS analysis. Our pNP catalog represents the most extensive list of annelid peptides to date. This catalog will provide a valuable resource for further studies of the peptidergic control of annelid behavior and physiology, and for the reconstruction of ancient metazoan peptide signaling systems and cell types [20].

## Results

### Establishing the *Platynereis* transcriptome, predicted proteome and secretome datasets

To identify novel pNPs, we performed deep sequencing of the *Platynereis* transcriptome using a combination of Sanger, Roche/454 and Illumina technologies. We sequenced 77,419 expressed sequence tags (ESTs) from an arrayed, full-length normalized, mixed-stages cDNA library [GenBank JZ391525 - JZ468943]. This library was further sequenced using the Roche/454 technology. We also obtained Illumina paired-end sequencing reads from 13 *Platynereis* developmental stages including larvae, juveniles and adults. We assembled all acquired sequences into a reference transcriptome. The *Platynereis* transcriptome dataset contains 351,625 reads, with 87,686 of the contigs longer than 500 bp and 28,067 longer than 1000 bp. The transcriptome was annotated using the Basic Local Alignment Search Tool (BLAST) against SwissProt and well-annotated bilaterian proteomes (Additional files 1, 2, 3 and 4). We also searched the transcriptome for open reading frames (ORFs) from which we derived a protein dataset. The predicted protein dataset contained 51,767 sequences longer than 120 amino acids (Additional file 5). To generate a *Platynereis* dataset of secreted proteins, the predicted protein dataset was analyzed for the presence of SPs [69]. We identified 11,075 protein sequences with a SP. After the *in silico* removal of the SP, this secreted proteome database (Additional file 6), as well as the full predicted proteome database, were used for MS-based peptide identification.

### Identification of *Platynereis* pNPs

In order to identify pNPs in the *Platynereis* transcriptome and predicted proteome datasets, we performed BLAST searches in these datasets using a large curated set of metazoan pNP query sequences [19]. We also conducted pattern searches for repetitions of the motif x(3–10)-K[K/R]. The resulting sequences were examined for the presence of a SP, for cleavage sites, conserved peptide motifs, and other hallmarks of bioactive peptides and their processing (e.g. amidation signature C-terminal Gly, pyroglutamination signature N-terminal Gln, Cys-containing stretches, mono- or dibasic cleavage sites). These searches identified more than 80 *Platynereis* pNPs, including those previously described.

**Figure 1:**
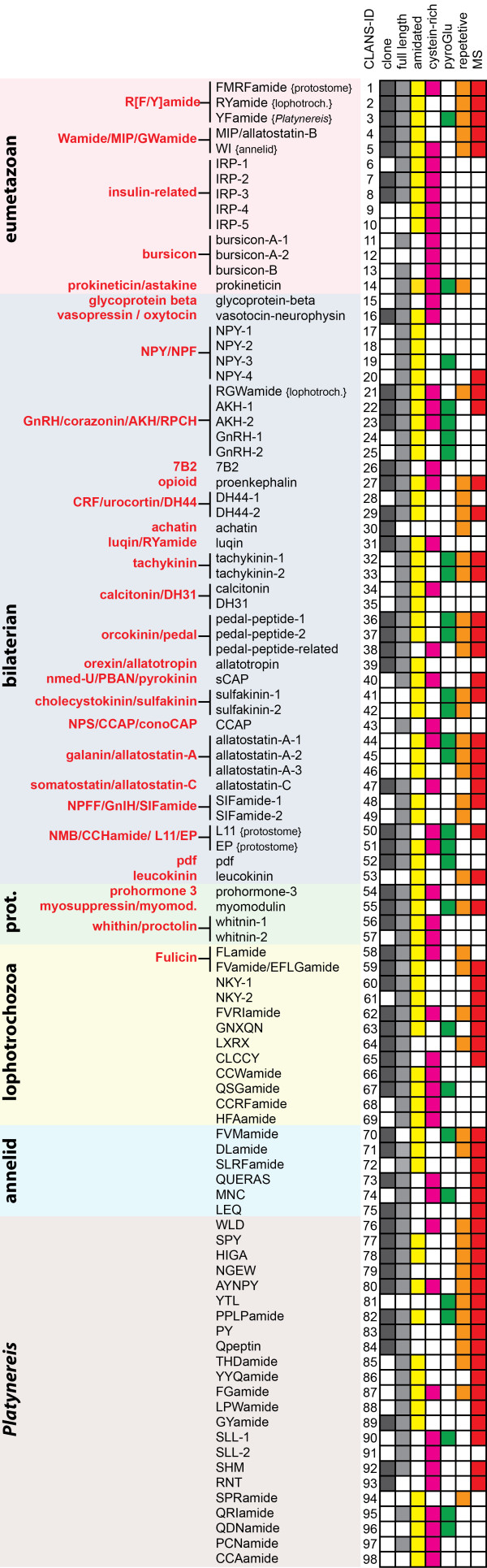
**Neuropeptide precursor complement of** ***Platynereis dumerilii*.** Neuropeptide precursors (pNPs) were classified based on their phylogenetic distribution into eumetazoan, bilaterian, protostome (prot.), lophotrochozoan, annelid and *Platynereis*-specific. Previously established metazoan pNP families are indicated in bold red [19]. For each pNP we indicated whether a cDNA clone, a full-length ORF sequence, and MS evidence are available. The presence of amidated, Cys-rich, or pyroglutaminated peptides, or a repetitive pNP structure, are also indicated. If a pNP family has multiple *Platynereis* members, we also indicate the likely origin of this paralog (e.g. {protostome}).

To complement the bioinformatics screen, and to find evidence for the presence of the predicted active peptides, we performed liquid chromatography - tandem mass spectrometry (LC-MS) on peptide extracts from various *Platynereis* larval and juvenile stages. We mapped the obtained MS hits to the *Platynereis* datasets. Using the MS analyses we discovered 15 further pNPs that were missed during our bioinformatic searches, extending the *Platynereis* repertoire to a total of 98 pNPs (Figure 1 and Additional files 7, 8, 9). We also analyzed the *Platynereis* neuropeptides for some of the most common peptide modifications and detected several modified peptides (Figure 1 and Additional files 8 and 9).

All full-length *Platynereis* pNPs have a SP and at least one potential basic cleavage site, and they lack non-neuropeptide protein domains as defined in the PFAM database. Besides, all 98 pNPs had to meet at least one of several criteria to be considered as bona fide prohormones. These include homology to known metazoan prohormones (e.g., NPY, AKH, 7B2 [70,71]), a confirmed expression in *Platynereis* neurons (e.g., FVMa, SPY [28]), MS evidence (e.g., NGEW, GYa), conservation across lophotrochozoans or annelids (e.g., CCWa, QSGa) and other structural and functional hallmarks of a prohormone, such as a repetitive structure or peptide modifications (e.g., SPRa, QRIa).

### Overview and classification of *Platynereis* pNP diversity

For most *Platynereis* pNPs we obtained the full-length open reading frame sequence (including a SP) and for 51 pNPs we have an available EST clone or a PCR-cloned cDNA. We annotated all pNP sequences with various sequence features, including the presence of a SP, cysteine-rich stretches (potentially also involved in processing [72]), prohormone-convertase cleavage-sites, modified (amidated or pyroglutaminated) or non-modified active peptides and the presence of a MS-hit. For all *Platynereis* pNPs containing repetitive peptides we also generated sequence logos (Figure 1 and Additional file 8). We classified the *Platynereis* pNP families according to their phylogenetic distribution, distinguishing ancient eumetazoan, ancient bilaterian, and ancient protostome families, as well as annelid/lophotrochozoa specific pNPs, and pNPs with no currently recognizable homologs outside *Platynereis* (Figure 1)*.*

**Figure 2:**
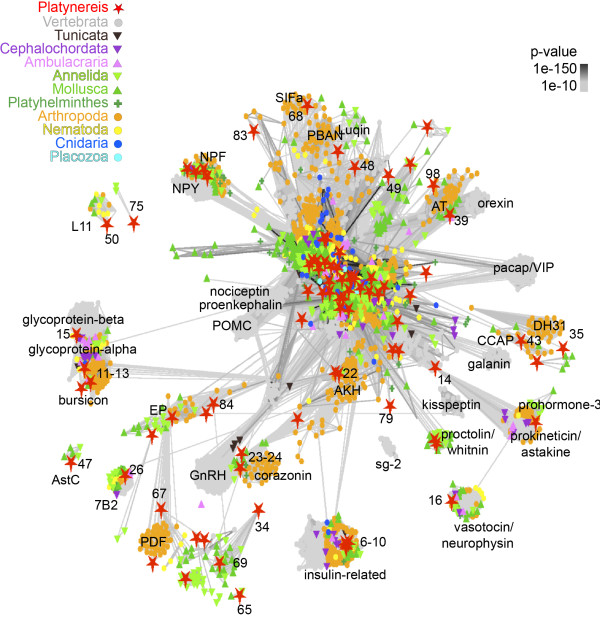
**PSI-BLAST cluster map of *Platynereis *pNPs and their metazoan pNP homologs.** Nodes are colored based on taxonomy. Edges correspond to BLAST connections of *P* value < 1e-10. *Platynereis* pNPs are highlighted as red stars. The identifier of *Platynereis* pNPs is provided in Figure 1 (CLANS-ID).

**Figure 3:**
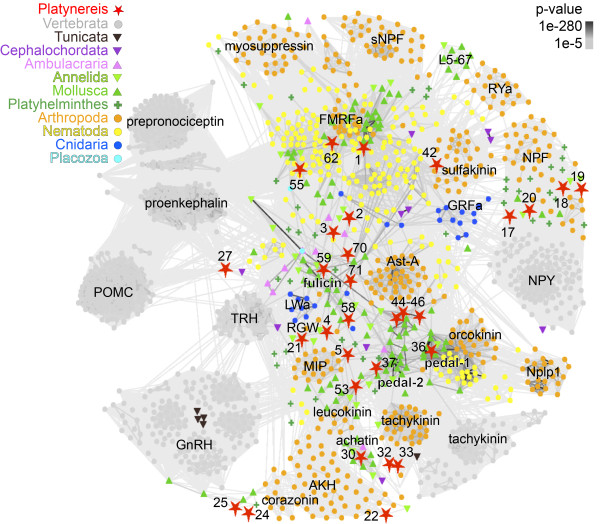
**Non-iterative BLAST cluster map of strongly connected pNPs.** The sequences that were strongly connected in the PSI-BLAST map (Figure 2) were re-clustered with non-iterative BLAST. Nodes are colored based on taxonomy. Edges correspond to BLAST connections of *P* value < 1e-5. The identifier of *Platynereis* pNPs is provided in Figure 1 (CLANS-ID).

For the phylogenetic classification, we performed a sequence-similarity-based (BLAST p-values) clustering approach [73]. We used a curated dataset of 6,225 pNPs from 10 phyla [19] combined with all *Platynereis* pNPs and their lophotrochozoan homologs collected from EST databases. We clustered this dataset using PSI-BLAST with 3 iterations. All *Platynereis* pNP sequences with no similarity to known metazoan pNPs were removed from the map. Metazoan pNPs that did not connect to any *Platynereis* pNP were also removed (e.g., parathyroid hormone, growth hormone) (Figure 2). Many of the repetitive pNPs formed a strongly connected cluster at the center of the map. These sequences were reanalyzed with clustering using non-iterative BLAST (Figure 3). The resulting maps were used to obtain an initial overview, which indicated a relationship of several *Platynereis* pNPs to known pNP families. The conservation of some *Platynereis* pNPs is limited to small stretches (the mature peptides) in the precursor and is difficult to identify using BLAST-based clustering (e.g., diuretic hormone 44 (DH44)). For this reason, we also performed motif searches and multiple-sequence alignments, which reinforced the family assignments, obtained by our clustering approach (Additional file 10).

Careful inspection of unassigned *Platynereis* pNPs for the presence of short conserved motifs using the motif discovery tool MEME [74] and known peptide profiles [20] led to the assignment of further *Platynereis* pNPs to known families (e.g., DH44, SIFa, pyrokinin/small cardio active peptide (sCAP); Additional file 10). We also discovered several *Platynereis* pNPs that belong to newly defined lophotrochozoan and annelid pNP families (Figure 1 and Additional file 10).

### *Platynereis* pNPs belonging to ancient eumetazoan families

Several *Platynereis* pNPs belong to ancient eumetazoan families [19] such as insulin-related peptide pNPs (IRPs) and the glycoprotein hormones bursicon A-1, A-2 and B (Figure 1).

*Platynereis* also has three pNPs that belong to the eumetazoan R[F/Y]a family, FMRFa, RYa, and YFa (Figure 1 and Figure 3 and Additional file 10). The FMRFa pNP represents the ortholog of other protostome FMRFa pNPs. RYa has orthologs in other lophotrochozoans, sharing a Pro-rich C-terminal peptide (Additional file 10). The *Platynereis* YFa pNP is also part of the FMRFa cluster (Figure 3), however, direct orthologs of YFa pNPs could not be identified outside *Platynereis.*

A member of the eumetazoan Wamide/MIP/GWamide family, the *Platynereis* MIP/allatostatin-B pNP, gives rise to peptides involved in the regulation of larval settlement [65]. *Platynereis* has another related pNP, yielding non-amidated W[I/L] peptides (Additional file 8 and Additional file 10). The two Trp residues that WI peptides share with MIPs (x-W-x_6-7_-W-[G/I/L]), and the position of the WI pNP in the Wamide/MIP/GWamide cluster (Figure 3) supports a close relationship between MIPs and WI pNPs. We only identified an orthologous WI pNP in the distantly related annelid *Capitella teleta*, suggesting that WI pNPs are annelid divergences of the MIP family.

*Platynereis* also has a pNP with a cysteine-rich prokineticin/colipase domain [75]. Directly after the SP, the *Platynereis* prokineticin pNP contains amidated LFVa peptides. A similar peptide could also be identified in the *C. teleta* prokineticin pNP (Additional file 10).

### *Platynereis* pNPs belonging to ancient bilaterian families

Recent comparative genomic analyses defined more than 25 pNP families that are ancestral to bilaterians [19,20]. We found *Platynereis* representatives of most of these families that could readily be classified as one-to-one or many-to-one orthologs. These include glycoprotein hormone beta, vasotocin-neurophysin, NPY, RGWa, adipokinetic hormone (AKH), GnRH, 7B2, proenkephalin, DH44, achatin, luqin, tachykinin, calcitonin, diuretic hormone 31 (DH31), pedal peptide, allatotropin, sCAP, sulfakinin, crustacean cardio-active peptide (CCAP), allatostatin-A, allatostatin-C, SIFamide, L11, EP, pigment dispersing factor (pdf), and leucokinin (Figures 1, 2, 3 and Additional file 10). Two of these families, calcitonin/DH31 and DH44, show interesting gene or peptide repeat duplications in their evolutionary histories and will be discussed in detail below.

**Figure 4:**
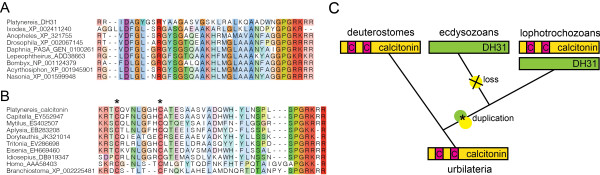
**Evolution of the calcitonin/DH31 pNP family. (A)** Truncated multiple sequence alignment of *Platynereis* DH31 with arthropod DH31 pNPs, all lacking cysteine residues. **(B)** Truncated multiple sequence alignment of *Platynereis* calcitonin with other lophotrochozoan and deuterostome calcitonin pNPs, all containing two conserved Cys residues. **(C)** Scenario of gene duplications and losses to account for the present distribution of calcitonin and DH31 pNPs in bilaterians.

*Platynereis* has two pNPs belonging to the calcitonin/DH31 family, calcitonin and DH31. The calcitonin pNP shows high sequence similarity to the Cys-containing calcitonin peptides from vertebrates (Figure 4). In *C. teleta*, a second calcitonin-like pNP has recently been identified that is lacking the Cys residues [20]. We also found an orthologous *Platynereis* sequence. These annelid pNPs are more similar to insect DH31 pNPs, also lacking the Cys residues, as shown by clustering (Figure 2) and sequence alignments (Figure 4). These results suggest that the ancestral bilaterian calcitonin peptide contained two Cys residues. The gene of this pNP duplicated in stem protostomes to give rise to DH31, followed by the loss of the Cys residues. Calcitonin has been retained in mollusks and annelids, but lost from the arthropod lineage. DH31 was lost from mollusks, and preserved in annelids and arthropods (Figure 4C).

**Figure 5:**
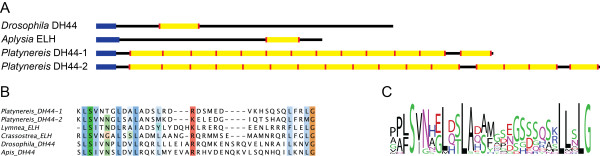
***Platynereis *has two highly repetitive DH44 pNPs. (A)** Precursor schemes of the *Drosophila* DH44, *Aplysia* ELH, and *Platynereis* DH44-1 and DH44-2 pNPs with the signal peptide (blue), cleavage sites (red) and predicted amidated peptides (yellow) indicated. **(B)** Multiple sequence alignment of protostome ELH and DH44 peptides. **(C)** Sequence logo generated from 16 different *Platynereis* DH44 peptides.

We also identified two members of the corticotropin releasing factor/DH44 pNP family. DH44 has been shown to be related to mollusk egg-laying hormone (ELH) [20]. We identified two pNPs in *Platynereis* that showed high sequence similarity on the level of mature peptides to mollusk ELH and also to insect DH44, with several highly conserved amino acid positions (Figure 5). The *Platynereis* DH44 pNPs are highly repetitive (13 and 16 copies) compared to their mollusk or insect counterparts that have only one peptide copy per pNP (Figure 5).

### *Platynereis* pNPs belonging to ancient protostome families

Several members of ancient protostome pNP families are present in *Platynereis,* including myomodulin, prohormone-3, and whitnin (Figure 1).

The *Platynereis* myomodulin pNP has two alternative transcripts, both yielding several peptides with an LRMa C-terminus, characteristic of myomodulins (Additional file 8 and Additional file 10). Comparison of the *Platynereis* myomodulin pNP with the *C. teleta* sequence revealed other highly conserved peptide-stretches, flanked by monobasic cleavage sites, which may potentially yield non-amidated peptides (PRXGX, Additional file 8 and Additional file 10).

### Lophotrochozoan-specific pNP families

We identified various pNPs belonging to lophotrochozoan pNP families including the fulicin related pNPs FLa and FVa/EFLGa, NKY, FVRIa, GNXQN, LXRX, CLCCY, CCWa, QSGa, CCRFa, and HFAa (Figure 1). Many of these could only be identified in annelids, mollusks, and sometimes also platyhelminths. We refer to these as lophotrochozoan families for simplicity. However, further sampling in other lophotrochozoan phyla will be needed to clarify their history.

**Figure 6:**
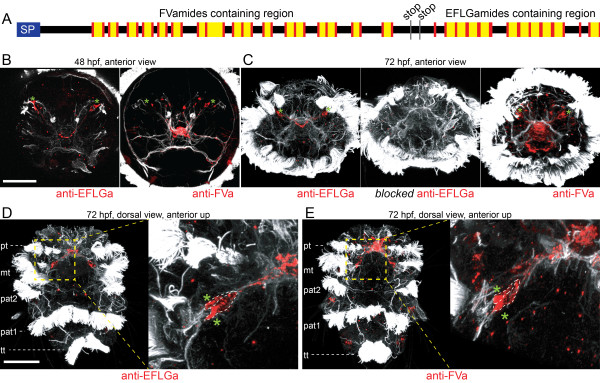
**Neuron-specific translational stop-codon read-through of the *Platynereis *FVa/EFLGa pNP gives rise to two types of neuropeptides. (A)** Precursor scheme of the *Platynereis* FVa/EFLGa pNP with the signal peptide (blue), cleavage sites (red), stop codons (grey) and predicted FVa and EFLGa peptides (yellow) indicated. **(B)** Anterior view of 48 hpf *Platynereis* larva stained with antibodies against EFLGa (left) and FVa (right). **(C)** Anterior view of 72 hpf *Platynereis* larva stained with an antibody against EFLGa (left), an antibody against EFLGa blocked with synthetic EFLGa peptide (middle) and an FVa antibody (right). **(D)** Dorsal view of 72 hpf *Platynereis* larvae stained for EFLGa (left) and a close-up of the EFLGa expressing neuron pair (left). **(E)** Dorsal view of 72 hpf *Platynereis* larva stained for FVa (left) and a close-up scan of a FVa expressing neuron pair (left). All samples were counterstained with a mouse acetylated-tubulin antibody (white). Green asterisks label neurons where FVa and EFLGa peptides co-occur. Scale bars: 50 μm. Abbreviations: pt: prototroch; mt: metatroch; pat1: first paratroch; pat2: second paratroch; tt: telotroch.

Annelid FLa and FVa pNPs are related to mollusk fulicins, forming a lophotrochozoan pNP family. The *Platynereis* FVa mRNA has an unusual structure. Following the stop codon at the end of the coding region containing the FVa peptides, a second putative coding region is present, potentially yielding fulicin-like EFLGa peptides with an extra Gly residue (Figure 6A). A similar structure has been described for the FVa/EFLGa pNP in *C. teleta*[40]. The region encoding the EFLGa peptides lacks a start Met and a SP, therefore it is not clear whether this region could be translated to yield mature peptides. The analysis of Illumina reads did not reveal any alternative transcripts with in-frame EFLGa peptides following a start site and a SP. The conservation of the EFLGa peptide-stretches across annelids (Additional file 10) suggests that these peptides are functional. Since MS evidence for EFLGa peptides was missing, we raised and affinity purified a specific antibody against EFLGa and performed immunostainings on *Platynereis* larvae. The EFLGa antibody labeled two neurons in the dorsal episphere of 48 h and two pairs of neurons in 72 h larva (Figure 6B and C), in the region where a subset of the precursor-expressing cells are found [28]. When we pre-incubated the antibody with 5 mM EFLGa peptide, we no longer observed cellular staining (Figure 6C). In agreement with previous studies [13,28], a cross-species reactive FVa antibody [13] labeled more neurons in the larval episphere at 48 h and 72 h (Figure 6B and C). EFLGa-positive neurons were a subset of these FVa expressing neurons as confirmed by their position, cell shape, and the presence of characteristic sensory dendrites abutting the cell bodies. (Figure 6D and E). These results suggest a translational stop-codon read-through mechanism [76] to yield bioactive EFLGa peptides, occurring in a subset of the precursor-expressing cells.

### Annelid- and *Platynereis*-specific pNP families

Some *Platynereis* pNPs have recognizable orthologs only in annelids, including the FVMa, DLa, SLRFa, QERAS, MNC, and LEQ pNPs (Figure 1 and Additional file 10).

For several pNPs we could not find orthologs outside *Platynereis*. Although we could not rely on pNP conservation as an indication of biological activity, we are confident that these pNPs produce bioactive peptides. In the case of WLD and SPY an effect on ciliary beating has previously been established [28]. Other pNPs harbor two or more copies of similar peptides, often confirmed by MS evidence, including HIGA, NGEW, AYNPY, YTL, PPLPa, PY, Qpeptin, THDa, FGa and SPRa (Additional file 8 and Additional file 9). Many pNPs contain peptides with hallmarks of biological activity including amidation (SPY, HIGA, AYNPY, PPLPa, THDa, YYQa, FGa, LPWa, GYa, SPRa, QRIa, QDNa, PCNa and CCAa), pyroglutamination (YTL, PPLPa, SLL-1, QRIa, QDNa) or various Cys residues that potentially form disulfide bridges (WLD, AYNPY, FGa, SLL-1, SLL-2, SHM, RNT, QRIa, QDNa, PCNa and CCAa) (Figure 1 and Additional file 8). Further sampling in annelids could lead to the identification of orthologous pNPs in closely related species.

### Stage-specific profile of *Platynereis* pNP expression

**Figure 7:**
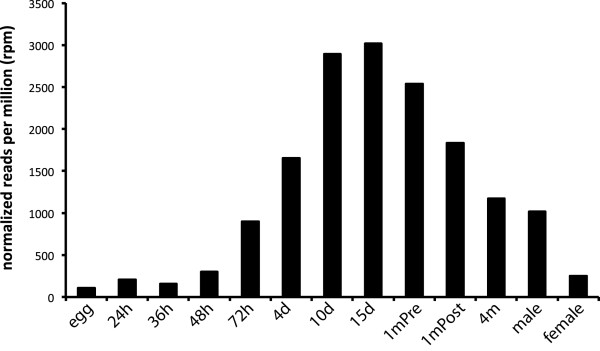
**Stage-specific transcriptional profile of pNP reads.** The sum of the total normalized reads per million (rpm) for the 98 *Platynereis* pNPs are shown for the indicated stage. The read counts for each pNP are normalized to the total amount of reads for each stage.

The *Platynereis* transcriptome dataset was acquired from various larval, juvenile and adult stages. To profile pNP expression, we mapped the obtained stage-specific Illumina paired-end reads to all pNP transcripts. The total number of pNP reads increases through larval development, peaks in 15-day-old juveniles, followed by a drop in adult stages. There are also large differences in pNP expression levels between sexually mature males and females (Figure 7).

**Figure 8:**
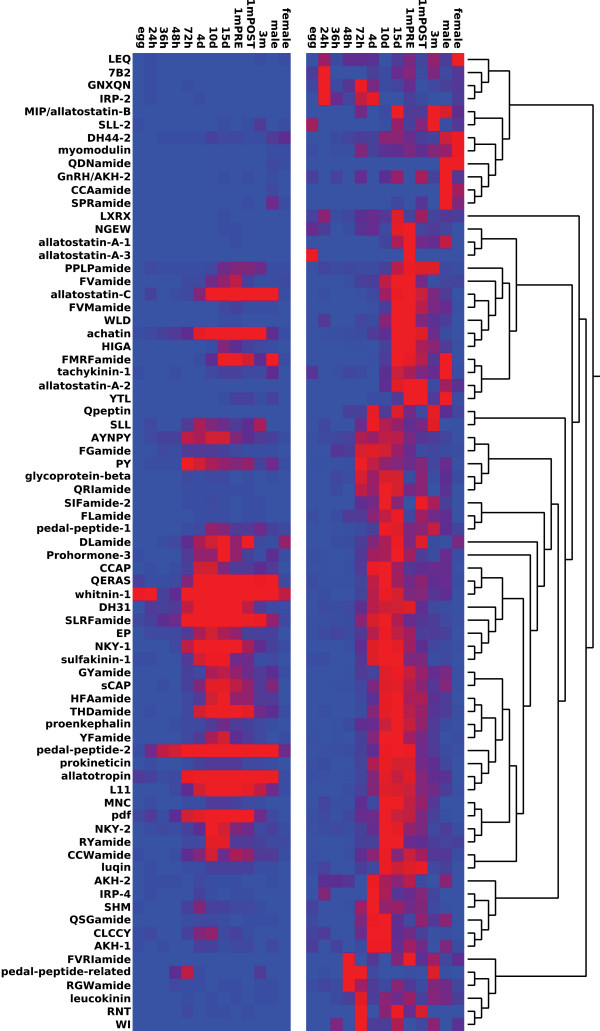
**Clustering of *Platynereis *pNPs based on stage-specific transcription profiles.** Pearson-clustering of *Platynereis* pNPs based on stage-specific transcription profiles, displayed as normalized reads for each pNP (left) or normalized reads transformed to 1 (right). red: high expression, blue: low expression.

We also performed a Pearson-clustering of pNPs based on their normalized stage-specific expression values. The *Platynereis* pNPs formed distinct clusters with expression peaking in different life cycle stages. We also found variable expression between 1-month pre- and 1-month post-cephalic-metamorphosis, and sexually mature male and female samples (Figure 8 and Additional file 11). For example, FMRFa, allatostatin-C and allatotropin were highly expressed in males, and lowly expressed in females.

## Discussion

### An integrative approach to obtain the *Platynereis* pNP repertoire

To obtain a broad complement of *Platynereis* pNPs*,* we used an integrative approach combining transcriptomics, peptidomics, and bioinformatics. Such an integrative methodology was indispensable to extend the *Platynereis* pNP repertoire to 98 sequences.

In particular, MS analysis was often necessary to reveal pNPs with more restricted phyletic distribution, lower sequence conservation, or non-repetitive precursor structure. For example, in the GYa and SLL pNPs only one short potential bioactive peptide occurs in the precursor sequence. Moreover, MS evidences combined with the conservation of the peptide in orthologous annelid and mollusk pNPs allowed us to conclude that these sequences represent bona fide pNPs with likely biological activity (e.g. GNXQN, LEQ).

### Expression profiling of *Platynereis* pNPs

The transcriptional profiling of pNP expression throughout the life cycle revealed that most of the pNPs are expressed at multiple stages. This indicates that the *Platynereis* nervous system is strongly peptidergic throughout the life cycle. The broad temporal expression of most pNPs also suggests that several of the *Platynereis* neuropeptides have pleiotropic functions. For example, we have recently shown that 12 different neuropeptides affect ciliary swimming and larval settlement in early larval stages [28,65]. Since larvae settle after a brief planktonic stage (approximately 6 days) and locomotor cilia disappear after cephalic metamorphosis (approximately 1 month), the later expression of these peptides implies functions unrelated to ciliary swimming. The other specific differences we uncovered between different stages may be related to changing physiology (e.g., initiation of feeding). The differences between males and females indicate the presence of sex-specific neuroendocrine regulation potentially related to sex-specific physiology or behavior (e.g., in pheromone production or during the nuptial dance of *Platynereis*[68]).

### *Platynereis,* a powerful marine invertebrate model for studying the function of ancient peptidergic signaling

Nervous systems evolved in a marine environment. Consequently, comparative studies of neuropeptide signaling in bilaterians would benefit from marine models with a rich set of conserved pNPs. Among the lophotrochozoans, a predominantly marine super-phylum, there are many emerging model species [77]. However, few studies used a comprehensive approach to identify pNPs in lophotrochozoans [20,25,40]. A recent study in the freshwater planarian *Schmidtea meditterranea* established the first broad pNP resource in a lophotrochozoan [34]. Compared to *S. meditterranea, Platynereis* has a richer repertoire of conserved pNPs. For example, *S. meditterranea* has no recognizable orthologs of achatin, luqin, AKH, allatotropin, CCAP, pdf, leucokinin, tachykinin, proenkephalin and whitnin [34,78], all present in *Platynereis*. Previous studies also highlight the potential of *Platynereis* as a powerful lophotrochozoan model for studying neuropeptides. Peptide functions can be easily investigated in *Platynereis* larvae by bath application of synthetic neuropeptides [28,65]. Recent technical advances now allow various genetic and other manipulations of peptidergic systems. For example, laser nanosurgery [65], transgenesis [79], morpholino-mediated gene knockdown [65], cellular resolution RNA *in situ* hybridization [80], complete neuronal reconstruction from TEM sections [65,81], and whole-body gene expression pattern registration [66,82] can be applied to explore the function of peptidergic systems in *Platynereis*.

## Conclusion

Here we used an integrative approach, combining transcriptomics, peptidomics and sequence homology searches, to obtain a broad pNP complement for the model annelid *Platynereis dumerilii*. Using homology-based sequence clustering and a comprehensive set of multiple sequence alignments and peptide-motif screens, we classified many *Platynereis* pNPs into conserved families. This work will serve as the foundation for further research of neuropeptide functions in *Platynereis* and for the study of conserved bilaterian peptidergic cells. Our pNP catalog will also provide a useful resource for the identification of pNPs in other annelids and mollusks (comprising more than 100,000 extant species), as well as understudied non-model marine invertebrates (e.g. bryozoans or brachiopods).

## Methods

### RNA extraction

*Platynereis* samples were obtained from an in-house culture at 18°C as previously described [83]. Samples were collected for RNA extraction at the following stages: fertilized egg, 24 hpf, 36 hpf, 48 hpf, 72 hpf, 4 days post fertilization (dpf), 10 dpf, 15 dpf, 1 month post fertilization with pre-cephalic metamorphosis morphology, 1 month fertilization with post-cephalic metamorphosis morphology, 3 month adult asexual (atokous) worm, male and female sexually mature (epitokous) worm. Total larval RNA was extracted from pooled batches of larvae (minimum 3 batches), consisting of hundreds of individuals. Total RNA for the 3-month adult stage was extracted from the combined tissue of 10 worms. Male and female epitoke RNA was extracted from the combined tissue of 4 epitokes for each sex.

Total RNA was extracted from all samples using TRIreagent (Sigma) according to the manufacturer’s protocol, with two additional phenol:chloroform:isoamyl alcohol (25:24:1) phase separations, followed by one additional 1-Bromo-3-chloropropane (BCP) phase separation step prior to isopropanol precipitation.

### Transcriptome sequencing, assembly and annotation

We used a combination of techniques to obtain a high-coverage *Platynereis dumerilii* transcriptome sequence. First, we generated a custom, normalized, full-length, mixed-stages cDNA library (with m^7^Gppp affinity purification to avoid bacterial RNA contamination; Invitrogen), cloned into the pCMV-Sport6 vector. After plating, we sequenced 83,152 randomly picked clones using the Sanger technology (ABI 3730) and the SP6 primer. 1,115 of the clones were also sequenced with the T7 primer. For base calling and vector trimming we used Phred [84,85] and Cross-match. Mitochondrial and ribosomal RNA sequences were removed using Ribopicker 0.4.3 [86] and a database of *Platynereis* mtDNA and rRNAs. The sequences were filtered with dustmasker, and every sequence with more than 20% low complexity regions was removed. The resulting 77,419 sequences were submitted to dbEST [GenBank: JZ391525 - JZ468943]. Second, we performed 454 sequencing (GS FLX, Roche/454) on the PCR-amplified cDNA library, following concatenation and fragmentation. We obtained 2,757,258 reads following adaptor trimming, quality (0.05), and length filtering (50 bp cutoff), using the software package CLC Genomics Workbench 4.5.1. Third, we performed Illumina sequencing on total RNA isolated from different developmental stages. Construction of sample-specific cDNA libraries from 5 μg total RNA, and paired-end transcriptome sequencing with an Illumina Hiseq 2000, was performed by GATC Biotech (Konstanz, Germany). Transcriptome Sequencing data was analysed using CLC Genomics Workbench 4.5.1 and 5.5.1 (CLC Bio). The raw read data for each stage-specific library was first filtered to remove Illumina adapter sequences, low quality sequences (Quality Limit 0.05) and short fragments (less than 30 base pairs).

All filtered 454 and Illumina reads were assembled using CLC Genomics Workbench 4.5.1. The resulting contigs and singletons were joined with all EST sequences, and passed to the CAP3 assembler with default parameters [87]. Transcript sequences were searched for the longest ORF and translated.

### Transcriptome mapping

The stage-specific filtered libraries were mapped to a subset of the assembled transcriptome of *Platynereis* (including only those sequences that had a BLASTX hit with an e-value < 1e-5 to the SwissProt database, and the 98 pNPs, a total of 52,631 transcripts) using the RNA-Seq Analysis function, with the following mapping parameters: paired distance 250 – 350 base pairs, minimum length fraction 0.8, minimum similarity fraction 0.9, maximum number of mismatches 2.

The total number of reads mapped to each gene in each stage-specific sample was normalized for total library size (reads per million (rpm)). For the subsequent analysis we focused only on the 98 pNP genes. To view the global pattern of neuropeptide expression throughout the *Platynereis* life cycle, we plotted the total sum of normalized expression values for all 98 pNPs in each stage-specific library.

The pNP genes were filtered based on their normalized expression values using the EdgeR RNA-Seq analysis package (Bioconductor) [88] in R version 3.0.1 to retain only those genes with at least 2 rpm in at least one developmental stage. The expression values were also further normalized for gene length (reads per kilobase million (RPKM)). The transcriptional profiles of the remaining 79 pNPs were clustered using hierarchical clustering with a Pearson correlation distance measure. The expression values were plotted using both the normalized expression values and the same values transformed to a fraction of 1.

### Peptide extraction

*Platynereis* larval and juvenile stages from 2 days post fertilization until 15 days post fertilization were collected in a small net of 100 μm mesh size (approximately 500–1000 animals in total). To remove contamination, animals were washed several times in sterile-filtered natural seawater. Excessive salts were washed off by rinsing animals 1–2 seconds in distilled water. Specimens were immediately transferred to ice-cold extraction solution (methanol : glacial acetic acid : distilled water, 90:9:1). Samples were centrifuged for 1 h at 4°C. The supernatant that contained the dissolved peptides was evaporated completely. Peptides were dissolved in 100 μl double distilled H_2_O and used for mass-spectrometry.

### Mass spectrometry: peptide sample preparation and LC-MS analysis

Neuropeptide mixtures were either directly desalted with C_18_ StageTips [89] or reduced and alkylated as described before [90]. LC-MS analyses were performed on an EasyLC nano-HPLC (Proxeon Biosystems) coupled to an LTQ Orbitrap Elite mass spectrometer (Thermo Scientific). Separations of the peptide mixtures were done as described elsewhere [91] with slight modifications: Peptides were eluted with a 87-min segmented gradient of 5–33-90% HPLC solvent B (80% ACN in 0.5% acetic acid).

The mass spectrometer was operated in the positive ion mode. Precursor ions were recorded in the Orbitrap mass analyzer at a resolution of 120,000. The target value for the Orbitrap was 10^6^ charges and the maximum allowed fill time was 100 ms. The 15 most intense precursor ions were sequentially fragmented in each scan cycle. High-resolution HCD MS/MS spectra were acquired with a resolution of 15,000 and a target value of 40,000. The normalized collision energy was set to 35, activation time to 0.1 ms and the first mass to 120 thomson. A minimum of 5000 counts were required to trigger MS/MS fragmentation and the maximum allowed fill time was 150 ms. The isolation window for MS/MS fragmentation was set to 2 thomson. Precursor ions were excluded from sequencing for 60 s after MS/MS. In one of the measurements MS/MS on singly charged precursor ions was enabled.

### MS data processing, bioinformatic analysis and validation

In total three MS measurements were performed. The acquired MS raw files were processed separately using the MaxQuant software (v1.2.2.9) [92]. Extracted peak lists were submitted to database search using the Andromeda search engine [93] to query a target-decoy [94] database consisting of the predicted *Platynereis* proteome (51,767 entries), the predicted secretome (11,075 entries), predicted neuropeptides (347 entries), commonly observed lab contaminants (248 entries), and the reversed complements of a those sequences (130,831 entries). We required full tryptic specificity allowing up to three missed cleavages and set the minimal peptide length to five amino acids. The initial precursor mass tolerance was set to 6 ppm, for fragment ions we used a mass tolerance of 20 ppm. For reduced and alkylated samples we defined carbamidomethylation of cysteins as fixed modification in the database search.

In order to screen for potential peptide modifications we performed a pre-run on one of the measurements by defining a fixed set of commonly observed (oxidation of methionine, acetylation of the protein N-terminus) and expected variable modifications (amidation of the peptide C-terminus) and iteratively included the following modifications in the database search: amidation of glycine; methylation of lysine and arginine; sulfation of tyrosine; acetylation of serine, threonine, alanine, glycine; pyroglutamic acid. Based on the results of this analysis we chose to include the following variable modifications in the actual database search: oxidation of methionine, sulfation of tyrosine, acetylation of the protein N-terminus, amidation of the peptide C-terminus, pyroglumatic acid.

Identified peptide spectrum matches (PSM) were statistically scored by MaxQuant software by calculation of posterior error probabilities (PEP) for each PSM. We considered all PSMs having a PEP below 0.01 for further analysis. The complete list of all identified peptide evidences including the respective spectra can be found in Additional file 9.

### Antibody purification, immunohistology and imaging

A specific rabbit antibody against CEFLGa was raised and affinity-purified following a previously described protocol [13]. The specificity of the obtained EFLGa antibody was also confirmed by pre-absorbing the antibody in 5 mM EFLGa for 2 h before applied in immunostainings, which completely blocked the antibody signal. The specificity of the cross-species reactive rabbit FVa antibody has been documented previously [13]. All samples were counterstained with a mouse acetylated tubulin antibody (Sigma, Saint Louis, USA). Immunostaining on 48 hpf and 72 hpf *Platynereis* larvae as well as image acquisition was performed as described [13].

## Abbreviations

AKH: adipokinetic hormone; BLAST: Basic Local Alignment Search Tool; CCAP: Crustacean cardio active peptide; conoCAP: Cardio active peptide of the conus snail; CRF: Corticotropin releasing factor; DH31: Diuretic hormone 31; DH44: Diuretic hormone 44; dpf: Days post fertilization; ELH: Egg laying hormone; EP: Annelid excitatory peptide; EST: Expressed sequence tag; GnRH: Gonadotropin releasing hormone; hpf: Hours post fertilization; IRP: Insulin related peptide; LC-MS/MS: Liquid chromatography - tandem mass spectrometry; L11: Abdominal ganglion neuropeptide L11 of *A. californica*; MIP: Myoinhibitory peptide; MS: Mass spectrometry; mt: Metatroch; NKY: Mollusk neuropeptide KY; NMB: Neuromedin B; nmed-U: Neuromedin U; NPF: Neuropeptide F; NPFF: Neuropeptide FF; NPS: Neuropeptide S; NPY: Neuropeptide Y; ORF: Open reading frame; pat1: First paratroch; pat2: Second paratroch; PBAN: Pheromone biosynthesis activating neuropeptide; pdf: Pigment dispersing factor; PEP: Posterior error probabilities; pNP: Proneuropeptide; PSM: Peptide spectrum matches; pt: Prototroch; RPCH: Red pigment concentrating hormone; sCAP: Small cardio active peptide; SP: Signal peptide.

## Competing interests

The authors declare that they have no competing interests.

## Authors’ contributions

MC extracted peptide samples, analyzed the transcriptome and mass-spectrometry data, performed immunoassays, participated in the design of the study and drafted the manuscript. EAW generated the transcriptome resource, performed the stage-specific transcription profiling and helped to draft the manuscript. KK performed the processing, bioinformatic analysis and validation of the MS-data and helped to draft the manuscript. MFW processed and analyzed mass-spectrometry peptide samples. BM designed and coordinated the mass-spectrometry analysis and helped to draft the manuscript. GJ designed the study, generated the transcriptome resource, performed sequence analysis and sequence clustering and drafted the manuscript. All authors read and approved the final manuscript.

## Supplementary Material

Additional file 1 ***Platynereis *** **transcriptome assembly, part 1 in GenBank format (txt).** The file was compressed with tar -jcvf archive_name.tar.bz2 file_to_compress, use tar –jxvf to uncompress it. Contains 85,000 sequences. Click here for file

Additional file 2 ***Platynereis *** **transcriptome assembly, part 2 in GenBank format (txt).** The file was compressed with tar -jcvf archive_name.tar.bz2 file_to_compress, use tar –jxvf to uncompress it. Contains 85,000 sequences. Click here for file

Additional file 3 ***Platynereis *** **transcriptome assembly, part 3 in GenBank format (txt).** The file was compressed with tar -jcvf archive_name.tar.bz2 file_to_compress, use tar –jxvf to uncompress it. Contains 85,000 sequences. Click here for file

Additional file 4 ***Platynereis *** **transcriptome assembly, part 4 in GenBank format (txt).** The file was compressed with tar -jcvf archive_name.tar.bz2 file_to_compress, use tar –jxvf to uncompress it. Contains 96,626 sequences. Click here for file

Additional file 5 ***Platynereis *** **predicted proteins in FASTA format (txt).** The file was compressed with tar -jcvf archive_name.tar.bz2 file_to_compress, use tar –jxvf to uncompress it. Contains 51,767 sequences. Click here for file

Additional file 6 ***Platynereis *** **predicted secreted proteins in FASTA format (txt).** The SP was removed from the sequences to facilitate the matching of MS hits. Contains 11,075 sequences. Click here for file

Additional file 7 ***Platynereis *** **pNPs in FASTA format (txt).** Contains 104 sequences. Click here for file

Additional file 8 **Precursor structures and peptide logos for repetitive motifs for all *Platynereis *** **pNPs, in portable document format.** Click here for file

Additional file 9 **Mass-spectrometry hits obtained by the analysis of *Platynereis *** **peptide extracts with the corresponding spectra for each hit, in portable document format.** Click here for file

Additional file 10 **Comprehensive list of multiple sequence alignments of *Platynereis *** **pNPs with homologous sequences, in portable document format.** Click here for file

Additional file 11 **Normalized Illumina RNA-sequencing read counts for 98 *Platynereis *** **pNPs in 13 different life-cycle stages, as excel spreadsheet.** Click here for file
