# Supplementary material for: The neuropeptide complement of the marine annelid Platynereis dumerilii
Source: BMC Genomics. 2013 Dec 20;14:906. doi: 10.1186/1471-2164-14-906 (PMC3890597; doi:10.1186/1471-2164-14-906)
Supplement: Additional file 8 — Precursor structures and peptide logos for repetitive motifs for all Platynereis pNPs, in portable document format. [file 1471-2164-14-906-S8.pdf]

Additional file 8: Structures of *Platynere*is pNPs and identified repepetive peptide motifs.

Structures of *Platynereis* pro-neuropeptides (pNP) as indicated, with the predicted signal peptide (blue), predicted amidated peptides (yellow) and predicted cleavage sites (red), cystein residues (pink) and potential pyroglutamination sites (green). Stop codons are indicated by asterisks (*). The peptides that are confirmed by mass-spectrometry are highlighted in **bold**, alternative MS-hits are underlined. For identification of conserved peptide motifs we used MEME [58] and manual inspection. For generating peptide logos, we generated multiple sequence alignements with MUSCLE and ClustalOmega and created peptide motifs using WebLogo. We provide these sequence logos if they are not available in previous publications [19, 28, 49].

Platynereis pNPs belonging to eumetazoan pNPs families:

>FMRFamide [GenBank:JF811326]

MRDQWLHLGALFFLAHAHSVISVTLESLCQSANEIHNEKLHIFCNAFKAYIEDLTESEDSSWDDGLAEKRGLKFGR
R**SDGNLGPMYFAIRH**RR**SFDPSLYLQMRQG**RGGGYIRFGR**SVHLASDPSQAYLASFGNVD**KRAGGHYMRFGRSVPN
SESTSVVSVPAAEADAVVSQLTDESDKEKRFMRFGK**SDPEEELHE**KDKKFMRFGRGDADDLEEAKRFMRFGRGGDE
EKRFMRFGRGDEEAAKRFMRFGRGEKGFMRFGRDPQEMSDDKRFMRFGRDGADEDEVEKRFMRFGKRFMRFGRDPL
KKRFMRFGKRDDDEDLAEEKRFMRFGKR**DGENGFMRFG**KRGDEEKRFMRFGKREDMDEEKRFMRFGKRESEDELDD
EEQKRFMRFGKRDSEVMDEQKRFMRFGKRDGGEEEKRFMRFGKRGDGEEEEKRFMRFGKKDGGEEEKRFMRFGKKS
ETDDKRFMRFGKKDNVEDAMDKRFMRFGKKDGAEDSADKRFMRFGRDPESKSEDDKRFMRFGKKDEVSE*

>RYamide [GenBank:JF811330]

MSIDSRTLILALAVFVTLAFAEDKDDQSTSVAADEDDKR**GTLLRYG**RGSLMRYGRGSLMRYGKRGTLMRYGKRDDE
EFYDDDDVDAIKRVFRYGKRGDDDYKRLFRWGKRSDDYVPEDGLDEDKRGTLMRYGKRVFRYGKRSDDDSASEDMA
KRVFRYGKR**NSVDLDDLIEEA**KRRIFRYGR**DIRAPQAPHVPFRFGEE***

>YFamide [GenBank:JF811332]

MNQVGLILTVLSLSSSLLIHATQDEALNGELQDRSSRVARSPDVDSEKRYPNTVLFGKRAPLFKFGKRRQFMFGKR
QDEEDAMDEGYDTDMMAEKR**YFGKMPVGSLYKGD**KRYYPTDQRHVTLEDLMERENRKFYFGKRTDDAAAADIRGDR
KFFLGKRDDLDEEKRKMVYFGKRMEDPEMDKRRMVYFGKREPNADMMDEEKRKMVYFGKRDPTEEKRKMVYFGKRE
PGQTEEEKRKMVYFGKRTDGDKSSR*

>MIP/allatostatin-B [GenBank:JX513877]

MDRVTITCFSLCLASVLIPLVHSEENVDLDEDKRAWMKNNIAWGKRGWKQGASYSWGKRDSEGDGLMSDEEKRAWN
KNNMRVWGKRSDEDDKRGWKDSSMRVWGKRAGEDDNNKRWGKNNLRVWGKRADDLEVLEDKRAWGDNNMRVWGKRS
DLEDDKRAWNKNSMRVWGKRDMEEDEDNKRAWKGQSARVWGKRADEDDKRGWNGNSMRVWGKRGWHGNGVRQWGKR
LHLDDVEPILDDEESKRAWAKNNMRVWGKRSTDNVRNMKAVVAEPAEVAADAESAKSS*

>WI [GenBank:KF515919]

MASMQWPIQLLAVLLALALTISSTHGLCEQQSPESSATAPSLLATGPVRSVLCKADRKAGEEMVKVAGRVFDHDCK
PLVHADVIVWQLKHNSLQGSRNPKEELDCHGRLVTGPSGEYTFSTVAGRSQPIHLQVHLPDGSHFTTDINIDDGKS
LQHSFDVVTKAKSHSRQTR**NVHDYELYNDDD**KRTWGDSGMNWLKKRSWGNANMDWLKR**NSMSDDSSRDWL**KRAWGN
SNMNWLKRKNWDEANTKWLKKRTWGDADMAWLKKKRTWGDANTNWLKRTWGDSGMSWLKKRAWAHPDTDWLKRTWQ
DNEDIMKWLKRSWGDANMNWIKR**DGLADEDEDQMVEVENPYEHV**RRRR**DVSSAGPEHEAQQVVAGEAEQGHVD**KKS
WGDSGMAWIKRDPHEKEEDKKSWGDSGMAWIKRDPEKKSWGDSGMAWIKRDDEKDKKSWGDSGMAWIKRDPEKKSW
GDSGMAWIKRDPEKKSWGDSGMAWIKRDPEKKSWGDSGMAWIKRDGDKRTWGDSGMAWIKKNADKRTWGDSGMAWI
KRHPGDKKSWGDADMAWIKKSWNDANVAWLKRNADKKSWGDSGMAWIKRNPDKKSWGDSGMAWIKRNPDKKSWGDS
GMSWIKRDGDKRTWGDSGMSWIKRNPDKKSWGDSGMAWIKRDPSKKSWGDSGMAWIKRDGEKDKKSWGDSGMAWIK
KR**DAEQSNLVSQQQPAEKESTGH***

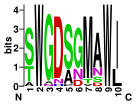

Peptide logo generated from 26 peptides of the WI peptide pNP.

>IRP-1

MWSVLVTITLVMAVSCQAETQGRRFCSSTLADMLSLVCGGRFYSGSDKRSQNDLVIRSGIEPGRKKRSVVEACCYR
TCSLEEMESYCEEGSSWRNLGSLMMERPTQDSKAPLGGDRDAQLSGGRSPSAASSRQVYDVLINGKPFFFLVPQAK
RNSDLQNLRVQNKPHMAAFEVKQADRR*

>IRP-2 [GenBank:KF515920]

MYTVYRLNWYVLLCILAASLSSTLGELTRTCHSDMMQKRGFCGSQLYNTLRLVCHPHGYHWQKRSVGNDPEFLEDS
PFLEKRLASSILHTNNRQKRGIICECCKHRCSYWELKEYCKAKKRSILPDADSEEPSQDQVTSNSIDDVASGLDAG
VASSSEWGSMREGVDSKTGFLPSAVMHDKANAKTDSKIGRMVRLLLDKSSTNNAP*

>IRP-3 [GenBank:KF515921]
MGTAHRFSWHLLLCLFATSSHGGLTRTCLGDTLDKRGLCGPGLSSALQIVCPNGYHYGKRSAGTEISPQVSAESPF
MDKRLAMSILHNRGKRGIICECCKHKCSFSELKEYCHVPVKRSFLSTDKPKLSQDQMNLIDVKWGLDDEGATSRLL
NIGDIEDDSDVNNKYLPSELTQDERNTRKDSQIGQLVRLLLDKSSAHNNI*

>IRP-4
(…)YGKRSAGTEISPQVLGKSPFMDKRLAMSVLHNRGKRGIICECCKHKCSFKELKEYCKTPG
KRGSLLLTAQPKPSQDQMTSNLIDVNWGLNNEGASSRLANMEDIEDDFDVTNQYLP(…)

>IRP-5
(…)CHNDHLVKRGLCGTGLSTALKKLCGMKGYHHGKRSAVTDTSLQDLTKSPFVDKRLAMSVLHNRGKRGSQGITC
ECCRHPCSVKELMEYCKSPFKRSLLLTAQPEPSQDQMTSNLIDVNWSFEDESAASRLDSMEGDLDVKNNYLPSKPN
HDEINSRQIGQLVRLLLNMTSAHDTL*

>bursicon-A
MTGAAAKRSFVCGFICCLICSVASEGNTEVDLPTGIVVPACRIRWIVHRIELPHCTQKRLLSLACKGQCESYTQYS
TDTNDIERVCSCCQPHGRKLRRIRMRCRNPKTFMPEIHFVQVYIPNSCMCRPCSVSIDNVPDVVNPLEAMSDNPPM
EFMLLRHK*

>bursicon-A-2
MRGVASKRSFVCGVVCCLICSILGGSTEVNLPTGIAVPSCKTHFIMHRIELPNCTRKRMLSLACKGECESYTQYST
DTNDIERVCSCCQPHGR(…)

>bursicon-B
MTTEWGTSLLLLVPAILILAHCPLSSASCNTDFTEIFLNKEKPYEYNGRPARLTCYRKVTIRKCEGTCTSEVSPSV
VRFPGFKKDCKCCRESRLDEKSYLLPCYYGNALIPGETVRLHIKEPMDCQCSECAM*

>prokineticin
MANIFHVCLLLMVFATIQGIPSKRGRSRPLFVGKRYPPYMPYRRTDYHGKRGRLQLLFVGKRQPTYEMSCRTDLDC
PSSHCCLADLENSSGTCTPLRHMEQICTTSSFYDGISYPMCPCASGLSCAISPEKRFGICTSSWDEEEGEEQYNY*

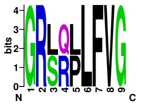

Peptide logo generated from 2 amidated peptides of the prokineticin pNP.

Platynereis pNPs belonging to bilaterian pNPs families:

>glycoprotein-beta
MNKGGGHKGQLLWLLSIMVIAMAVMTSSGHPWTGDSRAKQKRNERFNGNSVTSCGLYHHWKIAVNRDYLAENGTII
PCSAIVEVSICAGGCDTSEIPDYKVPFKIINHPVCTYGEVRPRTVRICGDNHPAPFAKVFDAVSCVCQPCSRSNAS
CESL*

>vasotocin-neurophysin [GenBank:EF544399]
MQFSRPTFTLQYGSAVLLTLVVCCSACFVRNCPPGGKRSMDLPQIHSTRQCMRCGPQGLGQCFGPNICCGPSIGCY
INTLESEECSKENDVRTPCDITAEICGVDGQGRCGADGVCCTDEKCTLDSSCDKLDMDKPRFSPDILRMLKKIFDR
RSYPGRRK*

>NPY-1
MTQILKLSLLFLILLGDKLLTCGKVLEEMPTLQQIPLKPVRPNRFRNKDELHSYLQSLRDYYSVIGRPRFGKRLSS
FLQRSPDSFGISESDNKGLGKTSDFSIEESPRLSFRKILLQSMRH*

>NPY-2
MLKFYCYLTALCILLTLVFSADMRGPPLRPAVFHNPKELRSYLKSLNEYFAIVGRPRFGRKRSPMPNFEEKRTLIT
PEVFMVMDSDGDGCIDQTELATFTSL*

>NPY-3
MRWTYLVLGLDLLFAVFLTFKLTSAQNMEGPPPRPAIFRTPQELRDYLSDLNEYFMIVGRPRFGRSTRHHLIDTVY
DKLGLPKSEKSS*

>NPY-4
MNLLFMWSTLVALLLSANLIISYPLTFTGPFEEDGLRLKRDPSFISSGPPVRPSSFKSPEELMEYLQKVRAYYNVM
SRPRFGKR**TIAEWYEGMDH**KR*

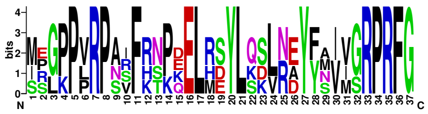

Peptide logo generated from the C-termini of potential peptides yielded by the four *Platynereis* NPY pNPs.

>RGWamide (JX412226)
MKLQGVVASWLFFGLVILEATYAADSGTSQIDKRRGWGKRDSEGEVDKRRGWGKR**GALDPELESEAD**KRRGWGKRS
SLEEMEKRRGWGKRTSVEEEKRRGWGKRDFEEEEEMDKRRGWGKRSLEEAEEKRRGWGKR**LTIDALEDGID**KRRGW
GKRSQMEDEEEKRRGWGKRSWPKDSAEACSEMRQAVVYYINSAIETEGMRVKQCGGDPIVTP*

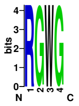

Peptide logo generated from several identical peptides of the RGWamide pNP.

>AKH-1 [GenBank:KF515922]
MRAWLVLLVACIVIAYQAHTSDA**QFSFSLPGKWGNG**KRAALGWGKRGECGDFDPDAIFNVYRAIQAEALRINECMQ
QKLEEESKKH*

>AKH-2 [GenBank:KF515936]
MMRLWVVYLTVCVMMLFAFFPHVCHSQLTQSLGWGSAGSSGKRSVKSPIYYGDDDDYDYDVRQVRKNRLTLSEALC
SQDEKDAISKIKKLIQREVVRQRYCNRKK*

>GnRH-1
MQNCSWVSCLLALATAWLTLSALLTPVSSQAYHFSNGWMPGKRSSLPLTNLRQVSRQLSQMTSSSPSSSDPRSDLS
FCRTQPQVFELIMTIIQAEMDRLQLLCSSETQDSTDDMETKKNSLLAWLKGKQNFGYED*

>GnRH-2
MRFCVIALVLVAMTALTSAQHAHFSTGWTPGFGIGKRMEGEPCNTKALESLQKLQAFTNVRFIILSSRILNRDFRI
MI*

>7B2 [GenBank:KF515944]
MRTWAMTGLAGLMLVLLVANVRADYYDDYLVQDLYKRLSQLDDYLAEDNEAYGGNSDWLDDRIPLDSRDGTTDIRD
HEYLEHSASKGGFQYISGGAGEGNQHLTPEGTQNNTHEVKSDEALPFYCHPPNPCPKGFTEEDGCQLDVKDTAEDQ
KKWISKMMASGQCSCDEEHMFSCPKDRSTMNADKGHEGRDDLDNVLDSLLAGKMDNPYSVGNKREKMVAKKGHPGN
LNLGNPFLDGQVVHTVAKKGGVGSVMMK*

>proenkephalin [GenBank:KC708483]
MKILVVFLLLQYHALAFASSEVEAVDKSEKEESSEESTSAFAESSQVDMCDSNKDINAVCKICDVLNGPQKGNCCS
DPDALLWCHLTVDEVMRTEGGQGEESAEDSGEGDHSEDSEESNEMDKRYGSLFSNWRWYSHRNDRSANANAEEAKR
GVEKR**YGNLLGGLFSNSNYGKD**KR**YGDLFSNSNYGKD**KR**YGGLFSNSHYGKD**KR**YGGSLFGRLFSNKG**KR**YGNLLG**
HVFGRSVDDKRDGQAEKRYGSLFTPMFGGKKDKR**YGSLFTPMFGGKD**KR**YGSLFTPMFGGKD**KR**YGSLFTPMFGGK**
DKRYGSLFTPMFGGKDKRYGSLFTPMFGGKKDKR**YGSLFTPMFGQKG**KR**YGGLLAQMYGRSEPTSD**KRSGEMQKRY
GHLLGQLFSNKSGKR*

>DH44-1
MPSTPRLGLLTLCIYLAIFTLTIATNDDRPDSSESDLDIHKRTRKLSVNTGLDALADSLRDRDSMEDVKHSQSQLF
RLGKRPAFSVNQDLSSLADAYGHGNAEESRARLLSLGKRVPLSVNSGLQSLADSMSDSSQNRLLALGKRPAFSVNH
GLDTLAEAMGMNGHNRLMSLGKRVPLSINHGLQSLADSMADDDRQSRLQMLGKRGPLSINNELHSLADAYGNQDSP
LLQLGKRGPLSVNAELHSLASSFAPGSHASLLNLGKRMPFSVNAELQSLAHSFKDGDSQSQLLRLGKRAPLSVGAE
LQSLADSYDGGDSRSRLLSLGKRIPFSVNQDMQSLADSMNGNRGSESNSFLQSLGKRAPLSIGQEMQSLSDAHMSG
YNPLLNLGKRVAFSVNQELQTLGDAMRQGDDRLLQLGKRSAKSHEANSRSKRVPGARRFAFNSELWSLAEMARHSR
SQQERKDVQDLLDNLGKRSV*

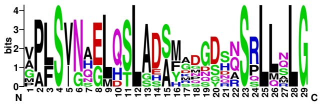

Peptide logo generated from the 8 different peptides of the DH44-1 pNP.

>DH44-2 [GenBank:KF515942]
MPTPLIQGLLSVCVLIALISLSLA**LNDDSAEFESSEGLH**KRAVKLSVNNGLSALANSMKDKELEDGIQTSHAQLFR
MGKRPSFSFNQDLKSLADAHGMHSAENSRARLLNLGKRVMFSVNGGLQSLADSMNEGPSHGNLMALGKRPAFSVNH
GLDTLAQAMGSDGSSQSKLLSLGKRPAFSVNHGLDTLAQAMGSEGSSSQSKLLSLGKRPAFSVNHGLDTLAQAMGS
EGSSSQSKLLSLGKRPAFSVNHGLDTLAQAMGSEGSSSQSKLLSLGKRPAFSVNHGLDTLAQAMGSEGSSSQSKLL
SLGKRMQLSFNQGLQSLADSMSNDGGRSRLMNLGKRGQLSINNELQSLAHSFKPNGDSHMSLLNLGKRAPLSVNAE
LHSLAHSFRPNSQASLLDLGKR**APLSINAELHSLAHSFNGDGEGRARLLNLG**KRVPLSINAELQSLAHSYQPNSQM
RLMSLAKRVPLSVNQGLVSLASSMRGDRAEASRNFLQSLGKRAPLSIGQEMQSLSDAYNSHSGNPLLNLGKRMALS
VNHELRTLGDAMRHGQAAPSLLSLGKRFDNQNDHEHVRIMKRSPGARRLSINSELWSLAQMARDARSHEDRKNAQA
LLDHLGKRSV*

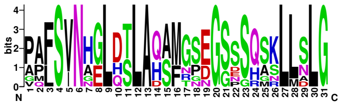

Peptide logo generated from the 8 different peptides of the DH44-2 pNP.

>achatin [GenBank:KF515943]
(…)STLFPYTTLFRSGKLTLVQVVSFALLVLVIANDENLLDSRELEENEIYDEIRDFGNKRGFGDKRGFGDKRGFG
DKRGFGDKRGFGDKRSAEAEIYNDKVLEEGIKERINAMFDSLPAAYKRGFGDKRGFGDKRGFGDKRAADKRGFGDK
RGHEEAFNSQELNDYLENLNNQLKFRMFAHHVFDKRGFGDKRGFGDKRGFGDKRGFGDKRGFGDKRGFGDK*

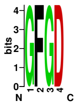

Peptide logo generated from several identical peptides of the achatin pNP.

>luqin [GenBank:KF515941]
MLCKYLAVATILAVLAPQLAQSWRPQGRFGKRTLDSNSLSEVFYGNKGLDADYGDDLSDSEDMPNAFRHNNLVCLR
KSQSGLYKCIRLRGAQDVPLDLHTA*

>tachykinin-1
MTSTIVLTLGLLSLIGHVTWAKLVLNDVYDQEDNNDLFEYEDFANSQKRSDDNLIEEEDRVDTYPFKRMDSNQFFH
MRGRRNGGESLTVDDQDPETLLKLARYFRYLQAKRSLRSNVPSLSAKRQPPKGFHAVRGKRSFHH*

>tachykinin-2
MTSAMLLTVGLLSLIGHLSWAKIVIPDVYDQDDNSDLFEFEDFANTQQKRRDTLIEDPEDEQVDSYPFKRMDSNQF
FHMRGRRNGQSEVMVDEQDPEALLKLARYFRYLQAKRSLRNDSPSGLSAKRQPPQGFHAVRGKRSVLH*

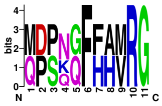

Peptide logo generated from four peptides of the tachykinin-1 and tachkinin-2 pNPs.

>calcitonin
MNWRSVSALICVIYVSVCIAENYEEDEKIETALRMIEQMAETRKGLSALKQVIDSIDGDLQKTHVEKRKVKDLTTK
RKGLEVLKRILKEMDSDLIMEQKRTCQVNLGGHCATESAASVADQWHYLNSPLSPGRKRRDTSLYQKLVGGKLLQ*

>DH31
MQATLATVVVLCLSSMLLLSHITSSHAMPALRQRRAADSDLTLALLEELVDELRADNVIQKRRIDAGYGSRYAAGA
SVGSKLRALKQAADWNGPGRKRRNAEEFDEE*

>pedal-peptide-1_mRNA1[GenBank:JF811329]
MVLWKLLLPLLATVLLVNAAPEKSHMKRSIVDDDDENSIYAGKGQSARQLDSLGGAEIPLYGRQLDSLGGAEIPLY
GRQLDSLGGAEIPLYGRQLDSLGGAQIPLYGRQLDTLGGGQIPAEDKRAFDSIGHHSAFGGLKRAFDSIGHHSAFG
GLKRSFDSIGHSSNFAGLDKRPFDSIGHSSSFAGLKKRPFDSIGHSSSFAGLKRAFDSIGHHSAFSGLRKRRSAEK
RSFDSIGHSSNFAGLDKKSFDSIGHSSNFAGLDKKSFDSIGHSSNFAGLDKRPFDSIGQSSNFAGLDKKSFDSIGH
SSNFAGLDKRSFDSIGHSSNFAGLDKRPFDSIGQSSNFAGLDKRSFDSIGHSSNFAGLDKRAFDSIGHSSNFAGLD
KKSFDSIGHSSNFAGLDKKSFDSIGHSSNFAGLDKRSFDSIGHSSNFAGLDKRPFDSIGHSSNFAGLDKKSFNSIG
HASNFAGLKKRSEQVNPEEEDKN*
>pedal-peptide-1_mRNA2
MVLWKLLLPLLATVLLVNAAPEKSHMKRSIVDDDDENSIYAGKGQSARQLDSLGGAEIPLYGRQLDSLGGAEIPLY
GRQLDSLGGAEIPLYGRQLDSLGGAQIPLYGRQLDTLGGGQIPAEDKRAFDSIGHHSAFGGLKRAFDSIGHHSAFG
GLKRSFDSIGHSSNFAGLDKRPFDSIGHSSSFAGLKKRPFDSIGHSSSFAGLKRAFDSIGHHSAFSGLRKRRSAEK
RSFDSIGHSSNFAGLDKKSFDSIGHSSNFAGLDKKSFDSIGHSSNFAGLDKRPFDSIGQSSNFAGLDKKSFDSIGH
SSNFAGLDKRSFDSIGHSSNFAGLDKRPFDSIGQSSNFAGLDKRSFDSIGHSSNFAGLDKRAFDSIGHSSNFAGLD
KRPFDSIGQSSNFAGLDKRSFDSIGHSSNFAGLDKRAFDSIGHSSNFAGLDKRAFDSIGHSSNFAGLDKRAFDSIG
HSSNFAGLDKRAFDSIGHSSNFAGLDKRAFDSIGHSSNFAGLDKKSFDSIGHSSNFAGLDKKSFDSIGHSSNFAGL
D*

>pedal-peptide-2 [GenBank:KF515945]
MIRSDVTLAFLFVCLAVRSFAAREKLEEQQFMIGSEAEDGLHKRMLDQVGSSLLKKRMSPQEDYLMKSQGQKRMLD
EVGSSLLKKRMLDEVGSSLLKKRMLDEVGSSLLKKRMLDEVGSSLLKKRMLDEVGSSLLRKRVVDPIGSALKKRML
DEVGSSLLRKRYLDRLGSSLIKKDAEEMMEEGDRVAYAANNPKRSYSREEVEELLGKEYAENNVHYPRQLDETAEG
LIG*

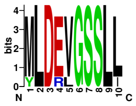

Peptide logo generated from 7 peptides of the pedal peptides from the pedal peptide-2 pNP
peptide pNP.

>pedal-peptide-related
MKPITSMLVLLSLMAFAGAMYNNPFTWAMHRFYQYRGISNQDRYNQDRHGNSDNSYGNSGSYGSRFQERYVLQGYP
YYQQQRSMGRRHQEQCRFEQCWDEFFMRRWGQGVTGESSQNGGQSNGFQNYEQCSEGVSRLRQCLQQQLNNNGGCG
CEYRDMMWAVRTARDSDGMATYAFCDDFYNEYTERQCKDDQYRRCVQTIQRLSFAENRYRCDLRDEVSFAKQCINK
YRSQPQPPSGCGNFNWFRYMEQDMEKVWMAEINGCPVYDIVKRCMNPMYEYFACSNQKDRVMTEYDQCIQKFANQD
GRCSPMREARKAMHQFMKFMLEQHCQSDRQVRERFQQCCHHEEDKKGFMSGSSSNNDNNRNGHQNNSNGYEEKKEK
KTEEKKEVKKEEKKEEEKK**FTMNGNGHEGNYEE**KKDVEKKRNGNQQHDQDRNQYDSNRNNQNGFDKKNYKQNGFDK
KDFNNQNGFDKKDYNNQNGFDKKHYNQNGQSNTNNHNDGNRSDYEDKKSYNNNFDKKNSNDNQSSNFKKSEEKKSY
NQQEDRKTKGGRHGNKNNKFSERGNNYDSYKPQEGEGRLAYLARVIKSWRDSQS*

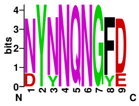

Peptide logo generated from 6 peptides of the pedal-peptide-related pNP.

>allatotropin [GenBank:KF515935]
MKVVLCLFVVVFVVSVNGMDLRAPRAKRGFRTGAYDRFSHGFGKRGELTNEDDGLMSVEDMAELITNTPKLALSFV
KRYMDRNDDGVISKEELLFVPEQ*

>sCAP
MKSWMKLSVCVLFVVILLHTVTS**LPPDFFRNG**RAGDLPGAKRTTGVGMVEEAACRYCGMYNPTLGFSCRSDIGDSR
RICLGDFATQLNSALEKHNLK*

>sulfakinin-1
(…)DLTPAEVAKR**QQGAWDMDYGWGGGRFG**KRAPLGKR**YDMYGIGGRFG**RDVSRAQQAAARQADLQ*

>sulfakinin-2
MNLNLALSCMIAVMWLFCGRCKAAPGLITGIHRNLGNHPHIRGYAVEGLVKDLMEDLVGEDEDNLRLSKRQQLDDY
GHMRFGKRSNNDDEEYGHLRFGRNIE(…)

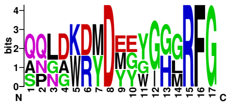

Peptide logo generated from four peptides from the sulfakinin-1 and sulfakinin-2 pNP.

>CCAP
MNQSTCLSVVLTFITLSVCLYQSSASRDSLTSQTFHQQKRIFCNFFGCGSKRSSSLPVGGQETHDPRLTELLQEAK
RSGALYQDDLPLVARSGSSSNSIYDDIFDDDTKKMAARSSLSDFSSESVDPEALLKKASLLSLLRSQIRRHSQLKG
SK*

>allatostatin-A-1
(…)KTKNCLESFECGKGSNDNLMISDSFTFPRFKSGEDGSIKLSQLPNVTINDLTNISKYLGLRKMIDYGLKFHGN
GRQTDNTLKFYGPGKRINEILGYSGSGKRTDNGFKLPRRPNNIFKFSGLVKRTDRARSGIKMRLSGKRVDDISKFS
GLGKR**DDNIFRFSELG**KR**VNNALKFSGLG**KRIDDGLRSSGSEKRTNDALEFSGLGKRGNDALQFSGLGKRGNDA*

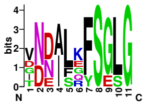

Peptide logo generated from 6 peptides of the allatostatin-A-1 pNP.

>allatostatin-A-2
(…)RK**ANNALKFSGLG**KRNDALKFSGLGKRANDALKFSGLGKREIDTLKFSELGKRNDALKFSGLGKRTNDALEFP
GSVKRADDHLIFSRLEKKEDDTLNFSGQGKRTEVGLRFSGLGKRNDDIMNFSGLGKRADDSLGERVNDVEQFSGLE
KRANDVMTFSGLGKRMDETLKFSGVGKKADDVLAFAELKKRVDNILKFSRIGQREDGGFRFSELEEKPDDILRAGE
KNLFRLKKKSDNGLKFSGLGKSHASKDSRMGKSTDGIMELPGLVKRANEIFRLSGLGKKIGNGLIFTGPERHIEEK
GTDNSILHRGEKYNSLNLSEFAKYMPQCTKSINNSFLFSKFRKGMIDNAILSVLSHRIDNGKTTPAPSPRKRRVNS
MHKSGPGKEVNQNNVLTVSGSDKFKDNDMMWIHPTEKINGYHHSRGISAVNQQADLYEPNSKSIPSSLKVGNPKHR
KDRSVTLPDWKNHNKTRIPPGRIFRESKNNLLPHFTPSRLQEIRIEGLNDGIPFKLKQKVVSRNSKKSVRRSKRRS
LVDEISAITNGKRLGHAYHSYVRRLSVDPGISLIGLGK*

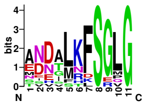

Peptide logo generated from 14 peptides of the allatostatin-A-2 pNP.

>allatostatin-A-3
(…)RDPGISGLGKRGNDALQFSGLGKRGNDALQFSGLGKRGNDALQFSGLGKRGNDALEFSGLGKRGNDALQFSGL
GKRGNDALQFSGLGKRANDALKFSGLGKRGNDALQFSGLGKRGNEALEFSGLGKRGNDALQFSGLGKR**GNDALKFS**
GLGKRGNDALEFSGLGKR**GNDALKFSGLG**KRGNDALQFSGLGKR**GNDALKFSGLG**KRGNDALKFSGLERK**ANNALK**
FSGLGKRNDALKFSGLGKRANDPLKFSGLGKREIDTLKFSELGKRNDALKFSGLGKRTNDALEFPGSVKR*

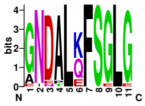

Peptide logo generated from 21 peptides of the allatostatin-A-3 pNP.

>allatostatin-C [GenBank:KF515923]
MVDFTAHTLAIVLIINCICIAFAEPAPSELGSVGDYENEHYKSLQGGMLERRLR**EDIEREMTDV**KKLEHQLMGHLN
LIQEKKRQLEIKKRQPVQCLVNIVSCWKRK*

>SIFamide-1
MEARLAVVLMATIFSVVLIARHASCEPLEDQLPDTTGLFFGKR**ASHPNMNNLLFG**RRSGVYDTYNGNQKFDLVQAR
RVCRSFRQTCASWGLDDEN*

>SIFamide-2
MASSKCFLTIFISVCLVIQVSSEPLEDQLPDTSGLFFGKRTRSHPNQNNLLFGKRNPAASQNAQIVCREVLQNCAA
LGLY*

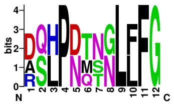

Peptide logo generated from 4 peptide motifs of the SIFamide-1 and SIFamide-2 pNPs.

>L11 [GenBank:JF811323]
MQKMCKVCLAFLVILILFSESSEARRRPDCTRFVFHPSCRGVAAKR**SESEEFVLDPR**SSINQEGGADHYISNGADE
DFIEFSEDDFAENKR**QADQASSYDLGLAAKLWPHI**KKALLGRTNRGSQDVEAFAPQKRNNEAKLEKWSKVLRHLLD
QQE*

>EP [GenBank:KF515924]
MDRTGLWYLLLVSVLYIWTGQKVSGKCSGQWAIHACAGGNGKRSEGALSSIGDRERDSRMSLQRILRGFGEYSNEV
EDQNRFASEPELSNYEDVLSQPQPDLTYLQPDLTYLQKENRKQKSVLRKLFTILKLRQNRQNLQN*

>pdf [GenBank:AEJ87224]
MGSSKTVQVAVVVCLVSMFVMQVVCYPTQRSNLRNSLTDADRQEILRYAAKIARIAMGDNVDFKAGPNKRNPGTLD
AVLDMPDLMSLGRK*

>leucokinin
MSSAKSTLYSSNGFLHFLLLLCTHLLHSIDATDYPSLLRTGGGSARLTDSLTHAQYRRGAFNPWKGKRSFSPWAGK
RDLSEVDADDKRAFSPWSGKRSELEDDKRAFSPWSGKRSELENDKRAFSPWSGKRSELENDKRAFSPWSGKRSELD
NDKRAFSPWSGKRSELDNDKRAFSPWSGKRSELEDDKRAFSPWSGKRSELEDDKRAFSPWSGKRSELENDKRAFSP
WSGKRSELDADKRAFSPWSGKRSDLDAEKRKFTPWAGKRSDNNDEVVDKRKFTPWAGKRSDDSSLEEKRKFTPWAG
KR**ISELVEEKE**KRKFNPWSGKR**SHALDDIEED**KRTFSPWAGKRAFQAWAGKRAFNSWAGKRDGLDTSDLEKLSEEQ
DEEKRGFNPWSGKR**SIGGLTEEDLKAVLHGLT**KRSVDSEDDSLSDKEQNGQPKKERMAVRHAPGKDSSGYHDSKHF
NSLNELHQHKPEGEVVDPTHIENPHHQIHKRSTGTNQKKFNPWA(…)

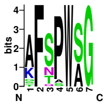

Peptide logo generated from 19 peptides of the leucokinin pNP.

Platynereis pNPs belonging to protostome pNPs families:

>prohormone-3_mRNA1 [GenBank:KF515946]
MKSSTFLAVFATLLATALGWSKYYEDSFDELPARRSYCVNSGGKCFTSSECCKGYVCAAFDDLFAGYCILEKSLKP
CAKDVDCAEDARCVSLGRKDERYCIQRADSPNDVIYRTAGPKAKLGQECTTNADCEKYGKDGNELCCQKIRRFRQK
AKTICDRVNHMSACIPNNADDWLSSKILTAEFVEDRNLANPELFLFSALMTLCWCGVNRYSVGFSPNDIPPSVLPS
INLIGWLSRLIVDRQI*
>prohormone-3_mRNA2
MKSSTFLAVFATLLATALGWSKYYEDSFDELPARRSYCVNSGGKCFTSSECCKGYVCAAFDDLFGRHVAKRIEPGY
CILEKSLKPCAKDVDCAEDARCVSLGRKDERYCIQRADSPNDVIYRTAGPKAKLGQECTTNADCEKYGKDGNELCC
QKIRRFRQKAKTICDRVNHMSACIPN*

>prohormone-3_mRNA3
MKSSTFLAVFATLLATALGWSKYYEDSFDELPARRSYCVNSGGKCFTSSECCKGYVCAAFDDLFAGYCILEKSLKP
CAKDVDCAEDARCVSLGRKDERYCIQRADSPNDVIYRTAGPKAKLGQECTTNADCEKYGKDGNELCCQKIRRFRQK
AKTICDRVNHMSACIPN*

>myomodulin_mRNA1
MTNCFALCLLVSCLCALSSAENDREKRAIRMLRMGKRGFGMLRLGRSAPYDQYESRQLFDVPRMGKDLVDIMEPSK
RHPPIPRVGNLDKVIDEYRRMVADEATSFDELPRFGRFVRSAEEVANEVEGSHKEKREVSGGVLPRLGLRDVENEI
RAAPLPRLGYRAIPRPRVGYRDLETLPRLGLRELEYQRAAPLPRLGLRENFADEDDKEERAVPLPRLGLRDLDKKA
VSMLRMGRSEETDTEHEMDKRAMSMLRMGKRGMSMLRMGKREEEIEPEVDEKRAMGMLRMGRAMSMLRMGKKDAIE
QYKRKMSMLRMGKRGMSMLRMGKRDDEQQEFTEEGKRKMNMLRMGKRAMSMLRMGKRDLDEYREQKRAMGMLRMGK
REFNDSPDEKRAMGMLRMGKRDSEDELQEKRAMGMLRMGKREYENLDEKRAMGML*
>myomodulin_mRNA2 [GenBank:submitted]
MTNCFALCLLVSCLCALSSAENDREKRAIRMLRMGKRGFGMLRLGRSAPYDQYESRQLFDVPRMGKDLVDIMEPSK
RHPPIPRVGNLDKVIDEYRRMVADEATSFDELPRFGRFVRSAEEVANEVEGSHKEKREVSGGVLPRLGLRDVENEI
RAAPLPRLGYRAIPRPRVGYRDLETLPRLGLRELEYQRAAPLPRLGLRENFADEDDKEERAVPLPRLGLRDLDKKA
K*

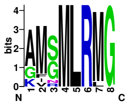

Peptide logo generated from 14 different peptides from the myomodulin pNP.

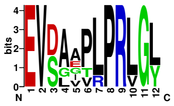

Peptide logo generated from 6 different peptide motifs from the myomodulin pNP.

>whitnin-1 [GenBank:KF515926]
MESKIIMMSLAVVLAALCLGASAMSLPSDEVKHNQVSESGLDEKERATWLDTRDLGDLEDQFKELVYLAVKELQNE
GRISPGVVSERKTQQKRGRFQGFCFRRTRSGRFLPYICWKGNDSRKQAKI*

>whitnin-2
MESKTIVMSLAVTLAALCLGASAMSLPSDDVKHNQVSESALDQKERATWLDTRDLEDQFKELVYLAIKELENEGRL
TTGIVAEDEPRQKRGRFQGFCFRRTRSGRFLPYICWKGGNSRK*

Platynereis pNPs belonging to lophotrochozoan pNPs families:

>FLamide [GenBank:JF811325]
MSTPVEPCMLLALFVLLATGSSHSAQSANVGSDVASCDQERLTLCKACGTPDLLDKCCLDDTTYLICLKEVSKADA
PYWEGELQDDDFEEDEKRAKYFLGKRAKYFLGKRPRNNFLGKRDSSLDFEDQYEGYDKRSPSPFLGKRGRSPFLGK
RAKYFLGKRENEYDSAPMSDNYFEEGDKRAKYFLGKRAKYFLGKRASWEELLKRAKYFLGKRSEDKRQKYFLGKRS
VDAVQQQEQQENSQ*

>FVamide/EFLGamide [GenBank:JF811327]
MHMSVGGWPLLLLIIAHGSSLQAQDADSENMRQLLQSMMLEDALSANENSLGGQNAAPGYIGDDKRAAHRFVGKRD
SEKRRLFVGKREDDEDEEKRAHMFVGKREDDKRAHRFVGKREEEKRRLFVGKRDDDKRNRMFVGKRSDFDDNDFEY
DEKRRYFIGKRDGISRDIRRLWIGKRGDGYDYKRPHNFVGKRGHMFVGKRDDFDLAEDKRRMFIGKRMYDDLEDYD
DKRARMFIGKRAHMFVGKRYMDDLDDEESKRRMFIGKRNRMFVGKRDDELDQDALSLDKRRQLYIGKKFAVEPAEE
TRDLEEGEEKRNRMFIGKRSAEVRQPADASVSSQ*SDASLRAQ*AIPVIPEIPGDYKKHFANYVAKRFSEFVGGKR
FSEFLGGKKRFSEFLGGKKRSAEFLGGKKRSEEEYPLEEIPDKRFSEFLGGKKRFSEFLGGKRSAEFLGGKKRFSE
FLGGKKRFSEFLGGKRSSGIFDSVPRSERSVDAILKRDFDFLGGR*

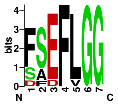

Peptide logo generated from 10 different peptide motifs from the EFLGamide containing stretch of the FVamide/EFLGamide pNP.

>NKY-1 [GenBank:KF515927]
MKQFILLVLSVSAYITLCVCEDSFSDWLQRGHVARRSAPESRLFGGKPKSKRDSNYAIKMLMEELLNELNESEERH
KETQMNQMKNLLEKEMSKRKAFWQPMMGGPLPVETRLASFGSRIEPDRTEPGSGPNGIKAMRYGRR*

>NKY-2
MNAIYSAALVLTYISTILGASLHSQQFAVAQPNDSAENYDFAAKASSQNSYNNWMRYLLSNAKHIVPDTDQKRFTD
FAMPYLKYKVSPVDKRNNGIWIWMPAQGYVSVPHQQEGGAADEGKPGKIMRYGK

>FVRIamide_mRNA_1 [GenBank:FJ358432]
MKGAPYVIPLTLVAVTSWLVCACVAANFHENDAEQQAEEIAELENDPYPKRRVSGFVRIGRDPSKK**ASSFVRIG**RG
SSSFVRIGRNPWGDKR**VSSFVRIG**KKSNDDVPAYLPYDDALVEDQEKRARSGFVRIGKSVGDEEKR**ASSFVRIG**KS
VDEPNYIEDFGHEYEEESPEKRASAFVRIGRPSSFVRIGRDPYKR**ASSFVRIG**KRSADSSEMEDSKR**ASSFVRIG**K
SVDEEKRNHFVRIGKSSEEDLDKR**VSSFVRIG**KSLSDEEKRARESFVRIGKALEDEEEKRGIREGFVRIGKAKSGF
VRIGKAKSGFVRIGKSMDDEKR**ASSFVRIG**KKDVGDEEKRASSFVRIGNPLGEKRPSGGFVRIGKNYEGDEVLEEA
KRPSSGFVRIGKSMDIVQEDGLAEPVDEAQKRASNFVRIGKRSADSMSDMTEEH*
>FVRIamide_mRNA_2
MKGAPYVIPLTLVAVTSWLVCACVAANFHEVCSHFCDSYNKVTSKESCVLVCENFIIKHLQNDAEQQAEEIAELEN
DPYPKRRVSGFVRIGRDPSKK**ASSFVRIG**RGSSSFVRIGRNPWGDKR**VSSFVRIG**KKSNDDVPAYLPYDDALVEDQ
EKRARSGFVRIGKSVGDEEKR**ASSFVRIG**KSVDEPNYIEDFGHEYEEESPEKRASAFVRIGRPSSFVRIGRDPYKR
ASSFVRIGKRSADSSEMEDSKR**ASSFVRIG**KSVDEEKRNHFVRIGKSSEEDLDKR**VSSFVRIG**KSLSDEEKRARES
FVRIGKALEDEEEKRGIREGFVRIGKAKSGFVRIGKAKSGFVRIGKSMDDEKR**ASSFVRIG**KKDVGDEEKRASSFV
RIGNPLGEKRPSGGFVRIGKNYEGDEVLEEAKRPSSGFVRIGKSMDIVQEDGLAEPVDEAQKRASNFVRIGKRSAD
SMSDMTEEH*

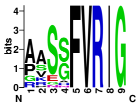

Peptide logo generated from 20 different peptide motifs from the FVRIamide pNPs.

>GNXQN [GenBank:KF515928]
MRVLLLLAGLLLCVYSAPLQQHSKRLSRAKR**AAEQVIFGNHQNNARV**KKSGPGLATPLEPLGQPVKGDEQAIDKKA
LVTSKVTSSEDVQVPAEVEGQEDLSDDLETDTKGHLAEEEEADKLAPLAEDSDASSQDATAADMKDEETEAGSSSQ
EDATEQSLEQLLNENEKIPSQAQQQGFPLDLFGKNQQQGQEYMDYPTYDYQYNPYLRRKRSLTPHKARAVNAEDMM
QGSAGIANSGRRSKRDLGQERYMWNDLDYPEVERRGYEEEEGPELDEEQQEALKEMLRDEILKYNPEMAEEVDNEI
GEEERPYEPEQEAQDLALINYLYRNGYAEAPEQPDYEEQGPEPWEDMGPAAEVALPEEEEPYFNPYGQYASEDNVM
SAPYSLAKRQYLSFVPGMRKRGNDFYPYSYGPDARWNAMVTEDVTEKRAEERMYERLLRLAAALRDRR**DELEAQRF**
YAEDFPEKK*

>LXRX
MKELTCLLVVVATIQLALSAAAPSQNKVVRMKRNYEQPYDLQFHQPDETALVRIKRQRRSAMEPTLVRVKRDEQEL
KRYKREKRDVEEELQRVKRQKRDTEEELHRFKRQKRDTEAELEEYKKRHSGEVEDPELARTKRFKREAEPPLQRYK
REKRDAEAEIHRFKRQKRDTEQELERYKR**DRDLEEPELSRT**KREKR**DVESSPTLAKE**KKGLKEKKSRAAGDRRRNQ
KALKREKKTNMNHRWEKHQKRNGNKHHISPRMKKNVLRKERRSDLLANHALRNVHLVSQRNKLGSKLAKHRVKRTQ
KIHQGHHYNKLEADVAKLGRLRRDIALNLHKANLKNAAKRRVKRTHAKKNKKIAAKKH*

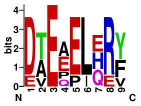

Peptide logo generated from 6 different peptide motifs from the LXRX peptide pNP.

>CLCCY [GenBank:KF515938]
MSSIYKMALLVLCALLLIALASS**RPSATNQEEEVDQANVARL**KRRTKCGFQLEACDPYENSCCPGMKCANLFGGYC
LSGIEKCMCLPTSYGV*

>CCWamide [GenBank:KF515929]
MRWIIFCFLIVLFSHTVLCWRIQDFANTDNEADDDDTSARYRQERRLLDYLRDVKRRHVKDRQVQKPPYFLTCSAW
QEPCDPWTNNVHTQCCDEDGLVCKCNFWAQNCKCVSRLWGR*

>QSGamide [GenBank:KF515940]
MPNSQVLIATVIFCVAVSCVCSKSIQVFNRHNERLKRQIGDLQIAHHQAQIGLKHWPKGCVEIGCGLFDIAASGKR
KRSYSNNVEDEFLNNENELRRESNRLVLLQRIADELMRES*

>CCRFamide
MQVKIIQLTTYSLIIALVQWEVIKALPTFPDMSACQAFCKANFQGEQHIACLTDICRSMYRGRISRFGKRVSHVME
DDLQEPLEPTALKDRLYAAYMKVLQHDQDRGSKRLPGEQQEKRSWPFALSLD*

>HFAamide
MTRDVTTAVTLALSVILVALLLTVQETNSFEYNGYCQKMCRWGRGGNLCKCNAVHFAGKRTSATPPPDVLDLPDDV
TNEVESSRAAEQLRKWFTRAALAVQSSKDGQAQENSASEDLSRAKQLLLMALKKLSDNSQGNSNPWSDITEDRLFD
NI*

Platynereis pNPs belonging to annelid pNPs families:

>FVMamide_mRNA1
MSLLWVHVAAFSLVATAVVLAYSDDKRFRKPEYQDFVLGKRDRPSYIDFLMGSKRSRRIYPEFVDFLLGSSKRPSY
SDFVLGKRSRPSFNDFYMGRRAERPSFSEFFMGKKRNDAFHDFYMGKRDAPNYLDFMTLDDQHVRKRRSANEEQTS
ESHNEANDAAVDKKDNDYGKFVMGKKNSQQQDFQAFSLGKRQEEKQNYDSFVMGKKKDKDYSKFVMGKKSDKDYSN
FVMGKKNGDDYSKFVMGKKDNKDYSNFVMGKKNAKDYSNFVMGKKDGQDYSKFVMGKKNDGDYSKFVMGKKNDGDY
SKFVMGKKNDDYSKFVMGKKNDDDYSKFVMGKKNDKDYSNFVMGKKNDG(…)
>FVMamide_mRNA2 [GenBank:JF811328]
(…)NADDYSKFVMGKKNADDYSKFVMGKKNDGDYSKFVMGKKNNGDYSKFVMGKKNVDDYSKFVMGKKNDGDYSKF
VMGKKNADDYSKFVMGKKNANEYSKFVMGKKNDGDYSKFVMGKKDDNDYSNFVMGKKNDGDYSNFVMGKKNDGDYS
KFVMGKKNDKDYSNFVMGKKNGEDYSKFVMGKKNDGDYSKFVMGKKNDGDYSKFVMGKRSIPEYDAFVMGKRNPKD
LDSFYMGKKEQKPDYQSFLLGKKSHGPQNYENFVMEKKNGEDYSHFVMGKRKNYQDFVMGKRGSADYQDFVFGKKA
SPNYQDFVLGKRSPNYQDFVLGKRNSQNYQDFVLGKRAAGNYQDFVMGKRGAPQYQDFVMGKRGAPHYQDFVLGKR
ASPNYQDFVLGKKASANYQDFVLGKKAEPNYQDFVLGKKSEGTQ*

>DLamide [GenBank:JF811324]
MAAGRQCLLTALVIFCACSQLTHALNENSVPLEKEDLLLTGDDEKRYAFNADLGKRSQLEELYEDEDADDKRSYGF
RSDLGKRRMGFNADLGKRFAAFNTDLGKRYYGFNNDLGKRYYGFNNDLGKRYSGFRADLGKRYMGFNADLGKRFSS
FRADLGKRLRDLDESHKRYSSFRADLGKRSVPSEDLDKRFMFRQDLGKRYSSFRADLGKRAMFRGDLGKRFSDSSD
DA*

>SLRFamide
MLSVSRWLQVLVVCVLGVVCTCALPSLAGYPDDGLHYRDEEDSFASSDPDKPVRDDGNGLGSLLLKNLRFRDLSNE
YHRLRPALLKSLALKPHYQEAQETKEFNKRGDMSVNELAQILNHLKARNVDGGVKLQSLRFGRRRR

>QERAS [GenBank:KF515937]
MEKIAVFLLVATIALAFSASINQADALYLPTNRVMKRSAAAAAANNHVKISSPRQKRYTDYQERASAFCTGLCMYE
ERKSYSECFDLCNYYW*

>MNC
MRSFLLVILALVLSALLVSSQSDDFSSQYSQWQLESEQPKQTEPDSISDFARDVKRLLLHLRRRKQPNSASPSSRS
ASKRFQLGQRINKRHCWDVADVCCMWNVC*

>LEQ [GenBank:KF515939]
MWIPLAAAVLFATVVSNIGAPLNPLPQKIHRESTKEILGLEQELKKAAGENKKPRPPAEEGPVPAEALADEELVKE
LAAHDEAVKDDKPGQSKETDEDSTEEELQQEKAVAEKAAELKAKQEEQEDLKDVEKEPESEPDVAQEEEEAQDQEE
PGNEEVHDEEELQNLQAPSNINDLYEDYLNEQRQAYYDAKRSYGAALDNLAEQENENKDEFVSSLLETIREHPELL
DEMRTGEVEEEEEQQPSYNYNPPSNDQEEALRAEYLAATRGLQEPQQQFESYASNPPYQGSPYEENEIRPPMENFQ
NYPSDKMAKVVAKKWGYSAPEEYYQQEENQAPLMEESAQEKKDHHEESLDQPQHLEPIM*

Platynereis specific pNPs:

>WLD [GenBank:JF811332]
MTSSGVLATLILGYLTNAVLTQEVVFPSVQRNLLRDHNTAQQTNCHKCKSLEGAAKLKCVARACGFGAILNKRWLD
NSQFIEDKRSHKVTRIAPWSKKARANHIRELLASRIGDLKNAKRWLDNSHFNEDKKWLDSMQFNEDKKWLDHSQFK
DEDKKWLDNSQFRDEKKWLDNSHFNEDKKWLDNSHFNEDKKWLDNSQFKDEDKKWLDNSQFREDKKWLDNSQFKDD
DKKWLDHSQFKDEDKKWLDHSQFKDEDKKWLDHSQFREDKKWLDNSQFKDEDKKWLDNSHFNEDKKWLDNSQFRED
KKWLDNSQFKDEDKKWIDASQFADDKRWVDMMHFNDEKRSAQQGLDSAEDMEESMKKKWVDPLNYGLDRKRSAYAK
LSMCFHQHCAHLAKMNYQNCVQARCMPLITSGETEGEATKA*

>SPY [GenBank:JF811331]
MGPRKMPCTSVVQLLFALFVVLCLISAAAPEQSDVENAAEQDVPDNAENKRSSSMESDKKAVKKDVNPSDELIGTK
QDKRSPIARLMKSNDDDKRSPYAGFMGPNKRSPYAKFMGSSEDKRSPYASFMGSNDEEKRSPIAKLMGSNDKRSPY
AKFMGSSEDKRSPYAKFMTDDERSPYANFMGSNDDEEKRSPIAKLMGSKDDKRLAYAVHPAAFHKFRAGKRSPYAS
FMGSNDEEKRSPIAKLMGSSDDKRLAYAVHPAAFHKFRGGKRSPYASFMGSNEEKRSPYANFMSDSSDDKRSPYAG
FLASNSEDKRSPYAGFMTGDKRSPYAKFMTGDEEKRSPYAKFMGSSEDKRSPYAKFMGSNEEKRSPYAQFMGSNEE
KRSPYAKFMGSSEDKRSPYANFMAGDKRSPYAKFLNEDKRSPYGAYMGMMSDDKRSPYAKYMYNENDKRSPGFRTM
RLGSNPRSIYDVQRDDTANIARGLRGPSMEQLSDNGLVQTIDGLKETDKLSDKRIPTDEDVLLKSQEDPINLPPAM
MQALLKPGENEVKKDEQNHGRQKRSALVYDEDKRTPYAGFREIMARNRELPVSRLNPAPGFKRTFETLNDISDRMG
SDGMLWSDSYSIPRFTGSFRKRRSTDEVHREKRSPGFRSMKPNWPDFDDMDKRSPGFRFMKWQSGQDDEDMGAKRS
PGFRHMRLRDNGSNSRYRNSYFLDALANELEEDKRGAGFRSMRIQPDNFEELDSDDMIDNDITKRYFGGSNRFLHP
AAFHRQLAFSHRRYRG*

>HIGA [GenBank:KF515947]
MIGILFFYCSLLVQLFCSASSQLADDAGSDVTKR**HLGSAIRWVDANRGRPGLE**KRHLGPALNVGYGGQFWFRDEPF
RLSRYNSRNDMYKRLATSLSYLKGQIKNSDADDVDDEQPSSNEVWRKPHHAFLNAGNSGRLPFWKDDRWSRRYKRD
AEVSVEDAHKEEDNEVKDRSKRHIGAAMNLAQLKEAENKRHIGAAMRLRPSKFEDKRHLGAALNLVRQANKVDKRH
LASAMKLVGRLRMPLSQIDSDVRDQMSEMNDDKRHIGADMRFAEATDNSDMDKRHLGAAMRLVQQPEDSDKMDKRH
LGAAMRLVQPKENDAKRHLGAAMRLVKSNNDNDKDKRHLGAAMRLVGTKENDEKRHIGAAMNLIGLKDDNENKDKR
HMGAAMNLVQTKDGDEKRHIGAAMRLVQPKETNEMAKR**HIGAAMNLAQADNENMD**KRHLGAAMRLVQPKDDNMEKR
HIGAAMNLVGLKEDNENMDKRHLGAAMRLVQTKDDDEKRHLGAAMRLVQPKSDGNMDKRHLGAAMRLVQPKQSEEK
RHLGAAMNLVGLKQDNSQSMDKRHLAAAMRLVQAKGDDDKRHLGAAMRLVQPNDDREKRHIGSAMNLIGPKSDEMD
KRHLGAAMALLQPKSDDKRHMGAAMNWRNSKNFNDDDRFEKR**HIGAAMNLVSPNNDNKDAE**KRHIGAAMNWRNSKN
FNDDDRFEKRHIGAAMNLVSSADDDENIDEEKRHMGAAMNWRNSKNFNDNDEGFEKRHIGAALNLVSPDDDNIDEE
KRHMGAAMNWRNSKNFNDDDDAFEKRHIGAAMALRPANIDDMGMNSMDKRHIGAAMALRPADSDDMAMDGMGKRHI
GAAMRLRAPSYSDRYSGAVESALVPLPAPPQAKEQKRTPPIFTSTVNIYASTYTQNGSNYSRKKMGTGPPPSKGQP
FASKILPRVKKGGFWPPTPPRTRPNFFSPESVEIFFKKKKTNPPPPPPPPFFGGGALRGGPTPAFFF*

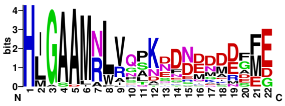

Peptide logo generated from 32 different peptide motifs from the HIGA peptide pNP.

>NGEW [GenBank:KF515948]
MISTGMLYTILFITLFAGSALCAEGVKKSEDSKRSAENYVEKDDDPRRYAGSQYDKKDSWKRFLKDAAKR**GDEWKY**
VENQNNRQQEWKRFMDEQNDLKKRLAEAGQETDNDKRGKVWSMVGDHHSNGEWKRDDERDLESLNEKRGKVWQWVS
DTHHGGQWKKAVDSEHPVGSKTDKRGKVWSIVGDHHSNGEWKRNDVNDETDKRGKVWSMVGDQHSNGEWKRNEENS
ETDKRGKVWSMVGDHHSNGEWKRNDVIDETDKRGKVWSMVGDHHSNGEWKRNDVSETDKRGKVWSMVGDHHSNGEW
KMNDVVDETDKRGKVWSKVDNHHRNEDWKRNDPSQIDKR**GKVWNMVGQSEPEGE**KRGKAWQWVSDTHHGGQWKKSV
DKRTSTKALAN*

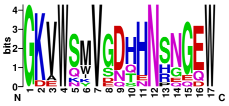

Peptide logo generated from 11 different peptide motifs from the NGEW peptide pNP.

>AYNPY [GenBank:KF515949]
MVSLRMAPVLWSALLVTLIGLTEVTGEESVSDASLLSDETEDDMEKRAGSRACQRCLHDSDDWGSCLACFSRPGPA
PYYGEKKRAVDPYAWDVEKKAVDPYDWDVKR**AYNPYDWRSFSAADLE**KRAYNPYEFEKR**AYNPYDWRSFDSTDFE**K
RAYNPYDMEVKKRGLSSCRCCMKIRLTSCCNKCSYAPYFTKRAYRTSYRPQFGENCACCRKDRFNYGCCLNCAGKR
K*

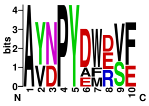

Peptide logo generated from 6 different peptide motifs from the AYNPY peptide pNP.

>YTL
MVEMRIIVLLAFLLPIQLICAEEVVHTKRQFGGPILSSAYTGNYRYKHRNGEERIQRPGAPQPNKNDKR**SSNDFDI**
DPYKRGLLGALEKIANSKR**YTLGTSYGGSYNNDNSDGYPH**KRYTLGTSYGGSYNNDNSGGYPHKR**YTLGTSYGGSY**
NNDNSDGYPHKRYTLGTSYGGS(…)

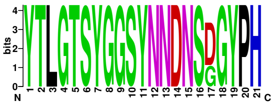

Peptide logo generated from 3 different peptide motifs from the YTL peptide pNP

>PPLPamide [GenBank:KF515950]
MVYKTIIPLVVILLFGHIHSIPKRKTLITLNDEDLREKKAEDAAAYPPFIPPLPGKKAGAASNNELALELALIDKK
GKQPSKNEIALQDLNEDEVISVGKKAQPDVKEELVDRLLEILKEDTVSGIKREEPPLPGKRETPPLPGKRETPPLP
GKRETPPLPGKREEPPLPNKREEPPLPNKRETPPLPNKREEPPLPNKRDEVISVGKKVLQDDNEELVDRLLEILKE
DTVSGNKREEPPLPNKRETPTLPNKRDEVISVGKKAQQDANNEQLVDRLFEILKEDNVLGEFDTPPVIPLPRNKIE
AIDKEVQQDDNMELIDRLLKYLTKK*

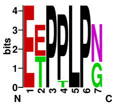

Peptide logo generated from 10 different peptide motifs from the PPLPamide pNP.

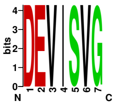

Peptide logo generated from 3 different peptide motifs from the PPLPamide pNP.

>PY [GenBank:KF515930]
MDLKTGIICSLSLVLLICTVCSDAFAIRRPENEELVRALARLDR**ALGEMISSPDDPHELSEPM**KRNEDGAVPYKRN
EDGSTPYKRNEGSSRKRLAVDGPVPYKRFSDESLDRVLRYLLDMEERLHERNTEKR**DHDIPFPFAPT**K*

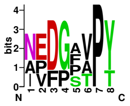

Peptide logo generated from 4 different peptide motifs from the PY peptide pNP.

>Qpeptin [GenBank:KF515931]
MGHFSTLLVLLATASVSFA**FYIRDDDAALSEA**RR**SDEERDELGAYDPFALFN**KRDEDKEKRMAELFDRSISEAVRS
LADFGYPEKR**DAEAEEEFAEAVAAEALEEVADAEQ**KRDQVKRYLDDFTDLLYMQRRGVGEAEEELADAVAAEALEE
VADAEQKRDQVKRYLDDFTDLFSMQRRGLGETEEELADAVAAEALEEVADAEQKRDQVKRYLDDFTDLFSMQRRGL
GEAEEELADAVAGEALEEVADAEQKRDQIKRSFDGLTEFLGMQER**NAEEEFAEAVAGEALDTVAEAEQ**KRDQIKKS
LDGVTEFLGMQRK**NAEENFTEDAEERDLEDM**KRYTHLLSSNQGYNRNNGGNRLGYNHNRMGYNRKGFKRDFTEEQV
EQMRELRDLFVDHVKRMTED*

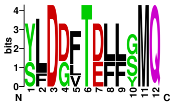

Peptide logo generated from 5 different peptide motifs from the Qpeptin pNP.

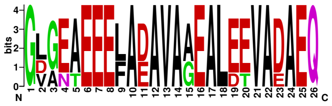

Peptide logo generated from 5 different peptide motifs from the Qpeptin pNP.

>THDamide
MNANQFIAVCLAVWFCLVAVAVAYPRAPAQWNWQVADDDDAAFLRSAK**ARGGPIAAPLWFLKTHDG**KRGSPVAAPL
WFLQTGGKRDAEAVNEDSGLWMVGDKAVKAPGYRTKREAIDTEDDMP*

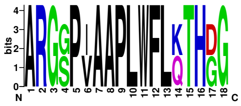

Peptide logo generated from 2 different peptides from the THDamide pNP.

>YYQamide
MNWKAVLLLSLFILAVTVFVTDGSIPRGGGKIRSRFKARVWNKKPGYRKPSDRKPDNEVSVPWKSQKKKPWVTLED
YYYQGKR**RTLTDPADELVYIE***

>FGamide
MAATSRSSLSIQLLSSCLLLTFSFESKSLTSA**KQLGPQQPSPW**KRLPTNGGKMRNVNTDNQIPSGNALNSTIVEEK
PVDMEIEVSKKFTVMRNNLANGFGGRHCLESAIHICDLRNFATSVTPPVTPCSLLRYFQNCMEDETNLCLEPDVIL
LKTSLKSLMSGFRANGKCHQMPLAFHEAWLNSGEGSAEECKPEATWACALQLVSDLKGLHDKTCDAILQFRNCIAM
HTHTCGYKGARLLVDGTQGLVWAYKKAAFCK*

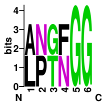

Peptide logo generated from the C-termini of two different peptides from the FGamide pNP.

>LPWamide
MKLQTTLAILVIAAIATTHSLPWGR**DLGGVEKVALEEALDKAL**RNTDFEPQEERRRRFPESFFNSYRDHYNALN*

>GYamide [GenBank:submitted]
MDISACAFCAIFLFASLLHTGSARPLDEYLIDQEIGKLQDTDRMEMLEKLLYRLIPSEDGENLRNGEDLEDDRSLQ
NYYDKHLKDLHVSTRSHSSSPVRYSNVFGTRNNYEGSRMPLRIAGSKR**NFDRNGWGGGYG**KK*

>SLL-1
MSMLGLQVMILVAVAVVLASAQGQEPPNPMAPKIRTIRDVLHEFVRR**NSVKSPNSASLL**KRLAYDNKDSKRFIPGA
FSSNCGSFGCNSQQSFGSNGFGSQGFGSQGFPSSSYDDDDEYYDDDEYYDDEYYDDE*

>SLL-2
MLGLQVMILAAVAVVLATGQGTGQQPPNPMAPKIRTIRDVFREFVRDNSVRSPNSASLLKRLGYYKKDSERFIPGS
FGTNCGPLGCNNQLSQDSHGFGSRGFEDQGLDTNSDENDDEYYDEEYRDRS*

>SHM [GenBank:KF515933]
MKNCVVLALFVLIGLVCLTLADETERDAAFHPRKTYERSSR**AIEVNSVEEYESHM**KRGAKKCSRNSVCYGVCCGLF
YQRCCKDGWRCTFEPSIRTYYCAPSHY*

>RNT [GenBank:KF515934]
(…)GHSLTMKQAVNLVVFLILPVSLAAVTLLGQDDEPFYLPNEIATSSQQETANALHQALAHVYSQLATDNRDDSD
IEDSKKIPPGLRSTSMEKRDRCYCDLDLFCFGCRRRKR**PDIRSLLAASMFRNT**RKRRDSQFTPNLHEISLNPALFD
NTVMDSLHAKRSKLSI*

>SPRamide
MMEKVFVFLIVVALAVALQPAAGGGLERRKSSTNDIFLDTTRGDESPRGRSKSKERPRSSSPSKGKRSRSRSKSRN
RDVDIPIVS(…)

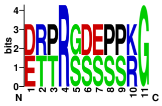

Peptide logo generated from the C-termini of two different peptides from the SPRamide pNP.

>QRIamide
MPSDHGNLSLLTRCLLLLTVISLATAQCPCMHCGPPMCRAVCAHDCYKCCECCVFFENRIGKRNSEIQSQPPKEDK
QESLCLFKRQLQLASAKQKEEDENVIPLDDVSDIAEAMSVRLSILRASEDRLNPDSSLADTSSPTGLDRFLAFLQQ
AAKSRET*

>QDNamide
(…)GSTTMRSPGLILLLGSAALAAGQLHYTAHHRVCCLDNGKRRGPEAIVLEKRFDPLTSFVISLNSLNYSRSDGC
IEYAGLRRGNLRILEELEEWPDNNDLHREQDHDIVFSSRPERNIDPGTAMNCLEVRISSQAEEHIKRRLTRIPFFT
HSDLKSMFPPKLDEKDTTMMLLAGTVIRPDEEETVYFKITDSQVINGVELYPGSVQFLSAIDRARIVTAEFVMGFA
KYKASSEEEEGGEEPDLPQRVLDTLRFLQYGTSRPIVSLKEADAFFFANQLSDPTLREDIAELNIKAVLGQLNEDF
SYISGKKMLAVNFGQTEKPPTTGTLSKFAKRL(…)

>PCNamide
MFMLLGLLLLSFEVQESAGLYVGPSGDSKGILQYLQQMILGRKKRDLTRNAESAPFCIPCNGK*

>CCAamide
MKTYVSHQREKFARHRMLSMLSLVASLLKMSAEPGGIDAKERERLETCAADSIKRDETEMIQQIELRFCQLLCAGK
RPKWTPGMRDVPRWGESINDNSTARFRDELKKFYDNIDQQISKEDEASEKSRLEA(…)
