# Supplementary material for: The neuropeptide complement of the marine annelid Platynereis dumerilii
Source: BMC Genomics. 2013 Dec 20;14:906. doi: 10.1186/1471-2164-14-906 (PMC3890597; doi:10.1186/1471-2164-14-906)

Additional file 9: Obtained MS-evidences for *Platynereis* pNPs.
For each MS-hit, peptide sequnece, spectral ID (ID), mass, posterior error probabilities (PEP) and number of total evidences (ev) are given. Modifications are indicated as follows: oxidation (*), amidation (am), pyro-glumatic acid (gl).
For each hit, spectra are provided following the ID numbering in the table.

precursor	peptide sequence	ID	mass	PEP	ev
***Platynereis* pNPs belonging to ancient eumetazonan families**					
FMRFamide	SDGNLGPMYFAIRH	1	1576.746	8.07E-03	1
FMRFamide	SDGNLGPM*YFAIRH	2	1592.741	6.70E-06	3
FMRFamide	SFDPSLYLQMRQam	3	1482.729	2.78E-09	2
FMRFamide	SFDPSLYLQM*RQam	4	1498.724	2.99E-04	2
FMRFamide	SVHLASDPSQAYLASFGNVD	5	2076.975	1.13E-06	2
FMRFamide	SDPEEELHE	6	1083.436	8.58E-03	1
FMRFamide	DGENGFM*RFam	7	1086.455	8.02E-03	1
RYamide	GTLLRYam	8	720.428	9.72E-03	1
RYamide	NSVDLDDLIEEA	9	1331.609	1.59E-03	1
RYamide	DIRAPQAPHVPFRFGEE	10	1964.986	1.21E-04	4
RYamide	APQAPHVPFRFGEE	11	1580.774	1.43E-04	1
YFamide	YFGKMPVGSLYKGD	12	1560.765	9.28E-03	1
MIP/allatostatin-B	LHLDDVEPILDDEES	13	1737.795	1.40E-06	2
WI	NVHDYELYNDDD	14	1510.585	6.09E-04	1
WI	NSMSDDSSRDWL	15	1411.567	4.14E-04	2
WI	NSM*SDDSSRDWL	16	1427.562	6.35E-04	1
WI	DGLADEDEDQM*VEVENPYEHV	17	2447.991	7.15E-07	3
WI	DVSSAGPEHEAQQVVAGEAEQGHVD	18	2545.132	3.59E-13	1
WI	DAEQSNLVSQQQPAEKESTGH	19	2282.041	7.10E-07	4
***Platynereis* pNPs belonging to ancient bilaterian families**					
NPY-4	TIAEWYEGM*DH	20	1366.550	5.73E-03	1
RGWamide	GALDPELESEAD	21	1244.541	8.74E-03	1
RGWamide	LTIDALEDGIDK	22	1301.672	1.18E-11	2
GnRH/AKH-1	(gl)QFSFSLPGKWGNam	23	1348.656	7.22E-05	2
proenkephalin	YGNLLGGLFSNSNYGKD	24	1817.858	3.47E-04	2
proenkephalin	YGDLFSNSNYGKD	25	1478.631	9.30E-05	3
proenkephalin	YGGLFSNSHYGKD	26	1443.642	1.89E-03	1
proenkephalin	YGGSLFGRLFSNKam	27	1443.762	1.81E-08	1
proenkephalin	YGNLLGHVFGRSVDD	28	1647.801	3.59E-04	1
proenkephalin	YGSLFTPMFGGKDK	29	1546.749	5.80E-04	1
Proenkephalin	YGSLFTPM*FDGKDK	30	1620.749	5.15E-03	1
proenkephalin	YGSLFTPM*FGGKD	31	1434.649	1.11E-04	3
proenkephalin	YGSLFTPMFGGKD	32	1418.654	1.11E-03	1

proenkephalin	YGSLFTPMFGQKam	33	1373.680	3.62E-09	1
proenkephalin	YGSLFTPM*FGQKam	34	1389.675	8.36E-05	4
proenkephalin	YGGLLAQM*YGRSEPTSD	35	1859.836	8.79E-03	1
DH44-2	LNDDSAEFESSEGLH	36	1648.685	6.49E-29	1
DH44-2	APLSINAELHSLAHSFNGDGEGRARLLNLam	37	3070.606	3.15E-10	2
tachykinin-1	SDDNLIEEEDRVDTYPF	38	2055.891	5.33E-10	2
tachykinin-1	(gl)QPPKGFHAVRam	39	1117.615	6.90E-04	1
tachykinin-1/2	MDSNQFFHMRam	40	1310.565	2.95E-12	1
pedal-peptide-1	AFDSIGHHSAFGGL	41	1414.663	2.18E-16	2
pedal-peptide-1	SFDSIGHSSNFAGLD	42	1552.679	5.48E-10	3
pedal-peptide-1	SFDSIGHSSNFAGL	43	1437.653	3.13E-04	3
pedal-peptide-1	PFDSIGHSSSFAGL	44	1420.662	3.87E-07	2
pedal-peptide-1	AFDSIGHHSAFSGL	45	1444.674	1.76E-14	3
pedal-peptide-1	AFDSIGHSSNFAGLD	46	1536.685	4.86E-10	2
pedal-peptide-1	SFNSIGHASNFAGL	47	1420.674	8.31E-07	1
pedal-peptide-2	MSPQEDYLM*KSQGQ	48	1656.712	2.91E-03	1
pedal-peptide-2	M*SPQEDYLM*KSQGQ	49	1672.707	4.21E-07	3
pedal-peptide-2	M*LDEVGSSLL	50	1078.522	4.07E-36	1
pedal-peptide-2	YLDRLGSSLI	51	1135.624	1.25E-06	4
pedal-peptide-2	DAEEM*MEEGDRVAYAANNPK	52	2254.947	2.86E-12	2
pedal-peptide-2	DAEEM*M*EEGDRVAYAANNPK	53	2270.942	4.87E-07	1
pedal-peptide-rel.	FTMNGNGHEGNYEE	54	1597.610	6.06E-03	1
sCAP	LPPDFFRNam	55	1003.524	7.76E-126	2
sulfakinin	QQGAWDM*DYGWGGGRFam	56	1844.769	2.57E-04	1
sulfakinin	(gl)QQGAWDM*DYGWGGGRFam	57	1827.742	5.76E-04	2
sulfakinin	YDMYGIGGRFam	58	1176.539	5.79E-03	1
sulfakinin	YDMYGIGGRF	59	1177.523	1.43E-21	1
sulfakinin	YDM*YGIGGRFam	60	1192.534	3.68E-03	1
sulfakinin	YDM*YGIGGRF	61	1193.518	1.27E-32	2
allatostatin-A-1	DDNIFRFSELam	62	1253.604	3.72E-04	1
allatostatin-A-1	VNNALKFSGLam	63	1060.603	6.17E-03	1
allatostatin-A-2/3	ANNALKFSGLam	64	1032.572	9.88E-03	1
allatostatin-A-3	GNDALKFSGLam	65	1019.540	5.00E-05	2
allatostatin-C	EDIEREMTDV	66	1235.534	1.00E-12	1
SIFamide-1	ASHPNM*NNLLFam	67	1271.608	1.20E-03	2
L11	SESEEFVLDPR	68	1306.604	5.58E-06	2
L11	(gl)QADQASSYDLGLAAKLWPHI	69	2166.075	1.33E-04	1
leucokinin	ISELVEEKE	70	1074.545	9.87E-17	3
leucokinin	SHALDDIEED	71	1142.473	1.13E-03	1
leucokinin	SIGGLTEEDLKAVLHGLT	72	1851.994	5.94E-05	1

***Platynereis* pNPs belonging to ancient protostome families**

myomodulin	(gl)QLFDVPRM*GKDLVDIM*EPS	73	2204.049	7.35E-03	1
myomodulin	M*VADEATSFDELPR	74	1595.714	5.09E-11	2
myomodulin	SAEEVANEVEGSHKE	75	1613.717	6.21E-11	2
myomodulin	SAEEVANEVEGSH	76	1356.579	4.57E-09	3
myomodulin	AMSMLRM*am	77	853.397	1.67E-07	1
myomodulin	AMGM*LRMam	78	823.387	7.62E-03	1
myomodulin	EEEIEPEVDE	79	1216.498	9.48E-03	1

Lophotrochozoan specific pNPs

FVamide/EFLGa	FAVEPAEETRDLEEGEE	80	1948.854	3.27E-10	4
FVamide/EFLGa	FAVEPAEET	81	991.450	3.99E-03	1
FVamide/EFLGa	SAEVRQPADASVSSQ	82	1530.727	5.96E-26	1
NKY-1	EDSFSDWLQRGHVA	83	1645.749	9.80E-08	2
NKY-2	FTDFAM*PYLKYKVSPVD	84	2035.997	3.45E-03	2
FVRIamide	VSSFVRIam	85	805.481	4.61E-10	1
FVRIamide	ASSFVRIam	86	777.450	3.01E-06	1
GNXQN	AAEQVIFGNHQNNARV	87	1766.881	3.26E-07	5
GNXQN	DELEAQRFYAEDFPE	88	1857.806	4.71E-04	2
LXRX	DRDLEEPELSRT	89	1458.695	6.68E-03	1
LXRX	DVESSPTLAKE	90	1174.572	2.57E-04	1
CLCCY	RPSATNQEEEVDQANVARL	91	2126.035	7.59E-15	2

Annelid specific pNPs

FVMamide N-term.	DRPSYIDFLM*GS	92	1415.639	9.55E-04	1
FVMamide N-term.	AERPSFSEFFM*am	93	1361.607	1.36E-03	1
FVMamide N-term.	DAPNYLDFM*TLDDQHV	94	1908.820	1.36E-06	1
FVMamide N-term.	(gl)QEEKQNYDSFVM*am	95	1514.635	4.34E-04	2
FVMamide N-term.	QEEKQNYDSFVM*am	96	1531.661	4.70E-03	1
FVMamide N-term.	SDKDYSNFVM*am	97	1219.518	8.19E-04	1
FVMamide N-term.	NAKDYSNFVM*am	98	1202.539	5.24E-04	1
FVMamide	NADDYSKFVM*am	99	1203.523	1.16E-03	2
FVMamide	NDGDYSKFVMam	100	1173.513	1.72E-06	1
FVMamide	NDGDYSKFVM*am	101	1189.507	2.09E-03	1
FVMamide	NDKDYSNFVM*am	102	1246.529	4.72E-08	2
FVMamide	SHGPQNYENFVM*E	103	1566.641	3.29E-04	1
FVMamide	KNYQDFVM*am	104	1058.486	1.65E-05	1
DLamide	YSSFRADLam	105	956.472	7.38E-04	2
SLRFamide	DDGNGLGSLLLK	106	1200.635	1.18E-04	1
QUERAS	SAAAAAANNHVKISSPRQ	107	1791.934	6.57E-112	7
MNC	FQLGQRIN	108	974.530	8.27E-04	4
LEQ	ESTKEILGLEQEL	109	1487.772	1.84E-04	2

***Platynereis* specific pNPs**

WLD	WLDNSQFKDED	110	1395.594	9.02E-03	1
WLD	WLDNSQFRED	111	1308.574	4.03E-03	1
SPY	DVNPSDELIGTKQD	112	1529.721	1.04E-07	2
SPY	SPYAKFM*GSSED	113	1333.550	1.40E-04	1
SPY	SPYAKFMGSSED	114	1317.555	3.85E-05	2
SPY	SPYASFM*GSNDEE	115	1448.540	1.29E-03	1
SPY	SPIAKLMGSND	116	1131.559	6.42E-03	1
SPY	SPIAKLM*GSND	117	1147.554	1.19E-03	2
SPY	SPIAKLM*GSKDD	118	1276.633	1.29E-03	3
SPY	SPIAKLMGSSDD	119	1219.576	1.50E-05	1
SPY	SPIAKLM*GSSDD	120	1235.570	6.23E-05	3
SPY	SPYAKFMTGDEE	121	1373.581	2.55E-04	1
SPY	SPYAKFM*TGDEE	122	1389.576	5.57E-05	2
SPY	SPYAKFMGSNEE	123	1358.581	4.12E-03	1
SPY	SPYAKFLNED	124	1182.556	3.29E-03	1
HIGA	HLGSAIRWVDANRGRPGLE	125	2103.109	6.32E-03	1
HIGA	HIGAAM*NLAQADNENM*D	126	1845.762	9.46E-05	1
HIGA	HIGAAM*NLVGLKEDNENM*D	127	2101.941	6.84E-03	1
HIGA	HIGAAM*NLVSPNNDNKDAE	128	2024.922	4.67E-04	1
AYNPY	AYNPYDWRSFSAADLE	129	1903.838	1.13E-05	3
AYNPY	AYNPYDWRSFDSTDFE	130	2011.822	7.37E-04	2
YTL	SSNDFDIDPY	131	1171.467	8.54E-03	1
YTL	YTLGTSYGGSYNNDNSDGYPH	132	2280.920	8.44E-06	2
Qpeptin	FYIRDDDAALSEA	133	1484.678	2.02E-04	1
Qpeptin	SDEERDELGAYDPFALFN	134	2086.912	2.21E-17	3
Qpeptin	DAEAEEEFAEAVAAEALEEVADAEQ	135	2635.130	7.75E-05	2
Qpeptin	NAEEEFAEAVAGEALDTVAEAEQ	136	2392.055	9.18E-07	1
Qpeptin	NAEENFTEDAEERDLEDM*	137	2171.844	8.98E-03	1
THDamide	ARGGPIAAPLWFLKTHDam	138	1848.016	1.68E-04	4
YYQamide	RTLTDPADELVYIE	139	1633.820	8.28E-06	1
FGamide	KQLGPQQPSPW	140	1264.656	6.57E-03	1
LPWamide	DLGGVEKVALEEALDK	141	1684.888	1.55E-10	2
GYamide	NFDRNGWGGGYam	142	1240.537	2.29E-05	1
SLL	NSVKSPNSASLL	143	1215.646	1.94E-05	2
SHM	AIEVNSVEEYESHM	144	1635.709	1.66E-06	1
SHM	AIEVNSVEEYESHM*	145	1651.704	1.44E-03	2
RNT	PDIRSLLAASMFRNT	146	1690.883	9.23E-03	1

peptide ID 1

Source:	20120515_CO_0340GaJe_R01
Scannumber:	15756
Protein:	pep_180; pep_secretome_488
Peptide Score:	71.15
Method:	FTMS; HCD; 1

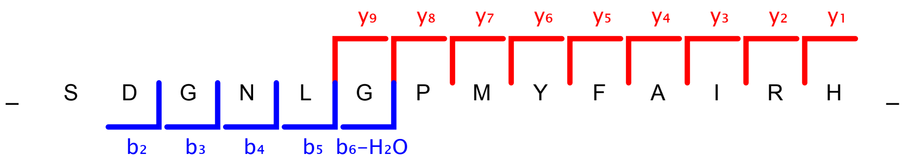

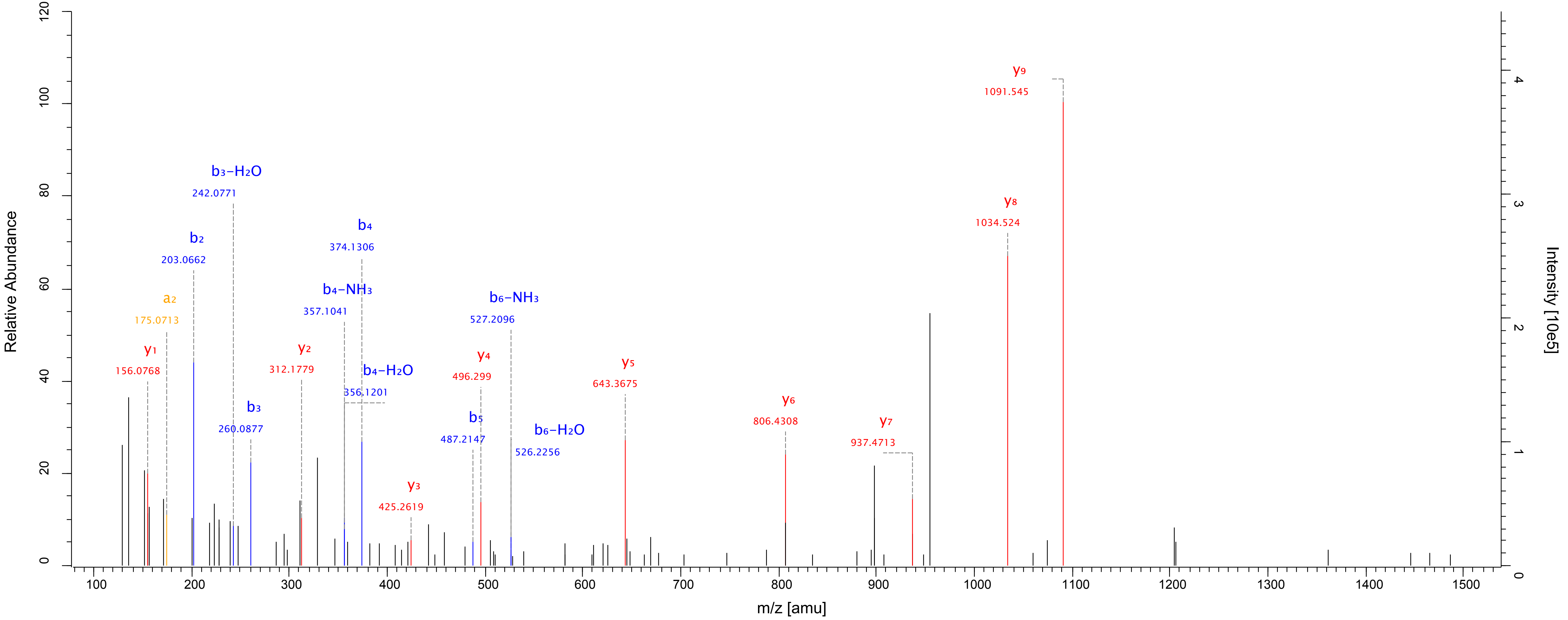

peptide ID 2

Source: 20120816_CO_0340GaJe_R02
Scannumber: 11042
Protein: pep_180; pep_secretome_488
Peptide Score: 89.4
Method: FTMS; HCD; 1

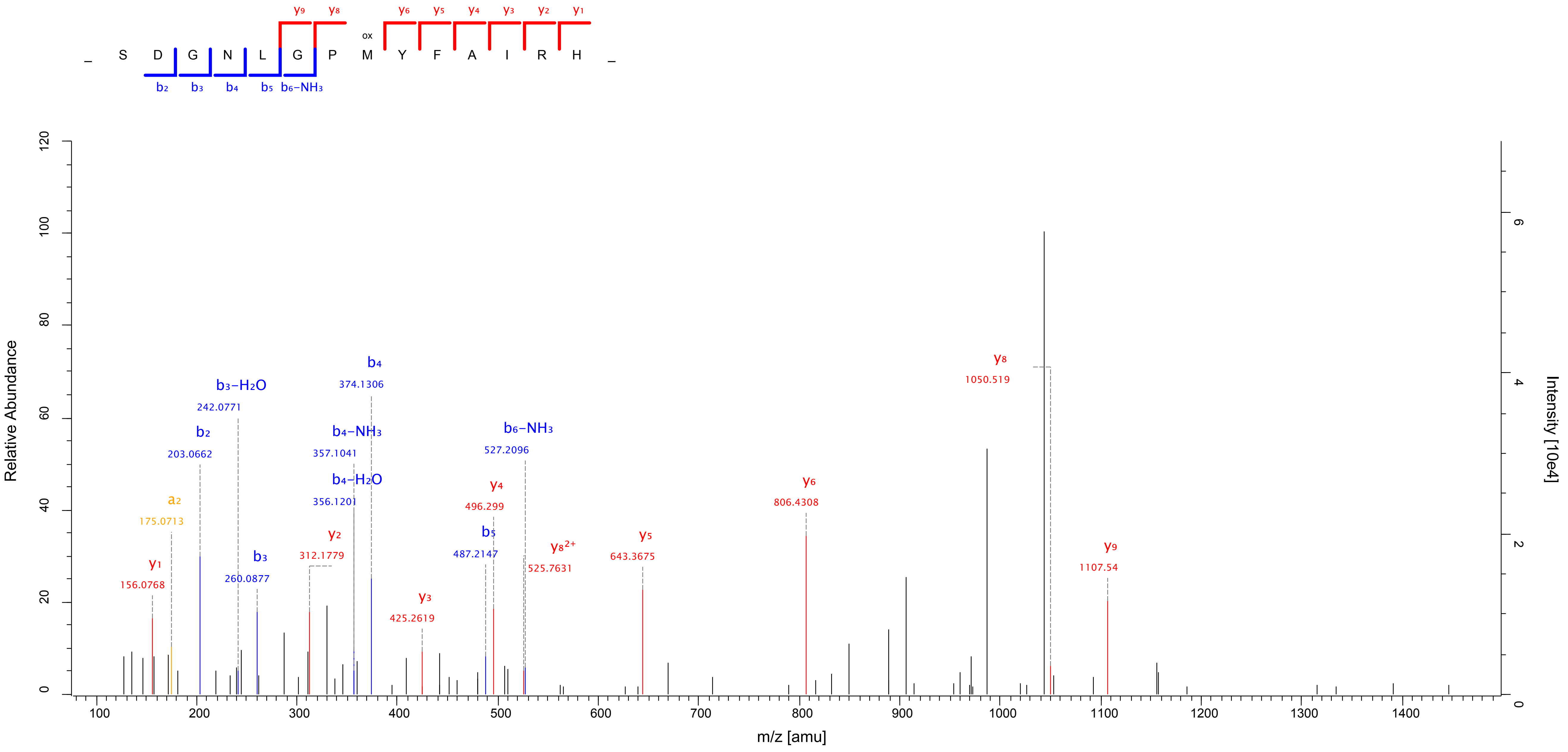

peptide ID 3

Source: 20120515_CO_0340GaJe_R01

Scannumber: 17844

Protein: SinglePep35

Peptide Score: 128.17

Method: FTMS; HCD; 1

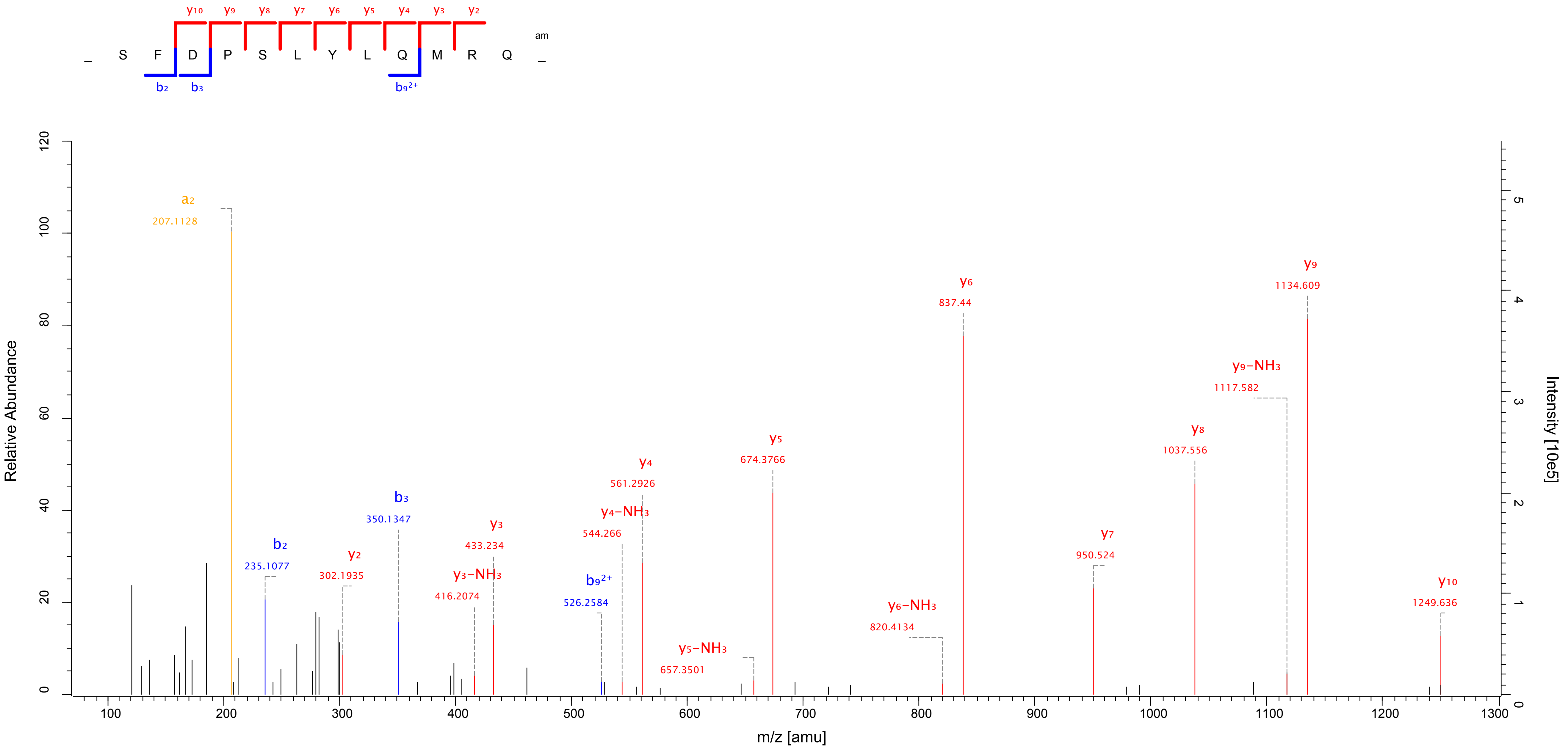

peptide ID 4

Source:	20120816_CO_0340GaJe_R02
Scannumber:	12493
Protein:	SinglePep35
Peptide Score:	82.17
Method:	FTMS; HCD; 1

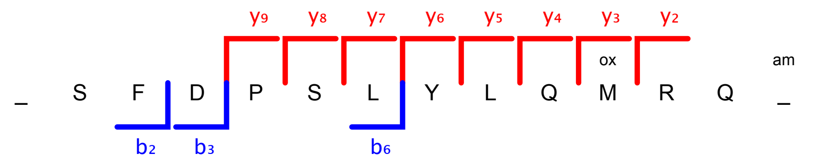

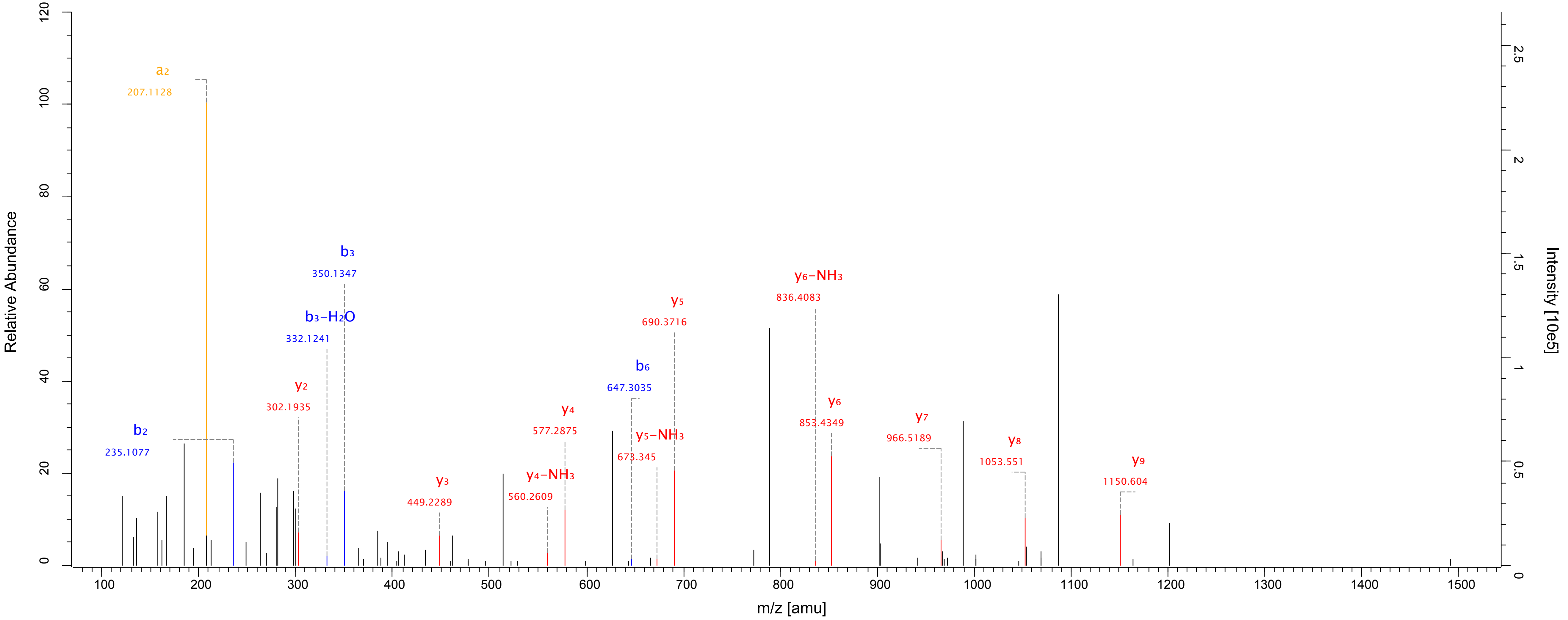

peptide ID 5

Source: 20120816_CO_0340GaJe_R02
Scannumber: 14399
Protein: pep_181; pep_secretome_489; pep_secretome_23365
Peptide Score: 76.71
Method: FTMS; HCD; 1

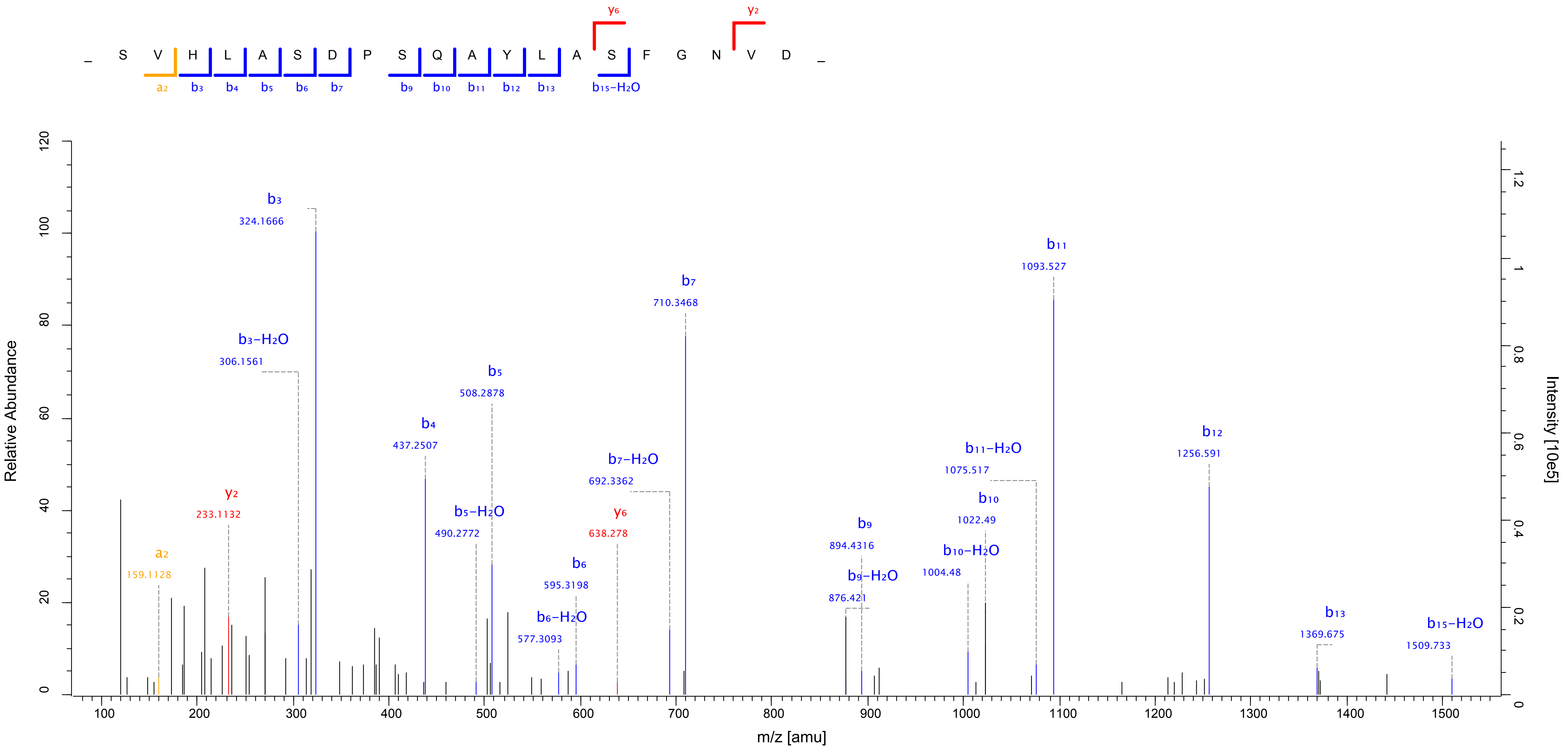

peptide ID 6

Source: 20120816_CO_0340GaJe_R02
Scannumber: 5888
Protein: pep_secretome_23368
Peptide Score: 105.14
Method: FTMS; HCD; 1

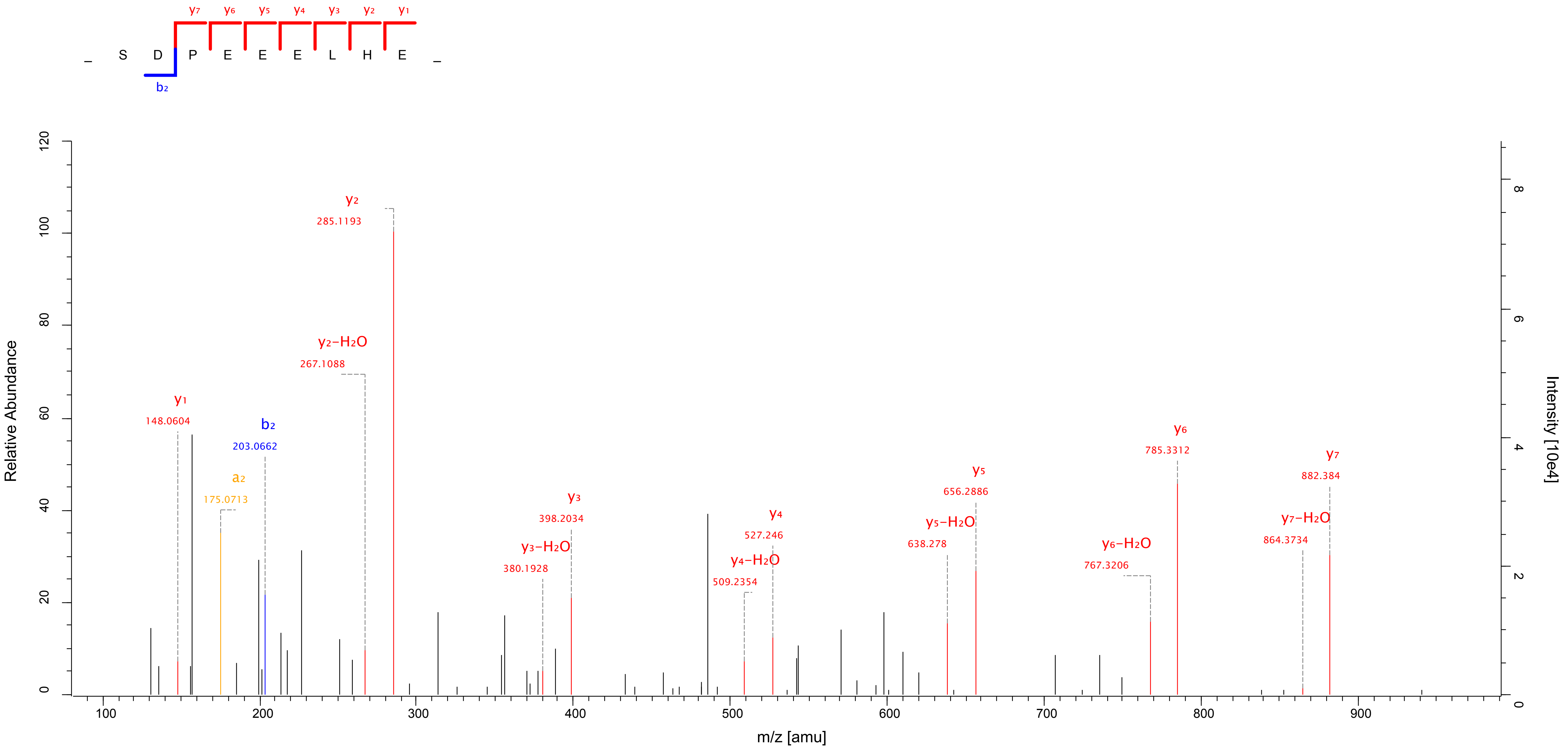

peptide ID 7

Source:	20120816_CO_0340GaJe_R02
Scannumber:	8800
Protein:	pep_secretome_502
Peptide Score:	77.67
Method:	FTMS; HCD; 1

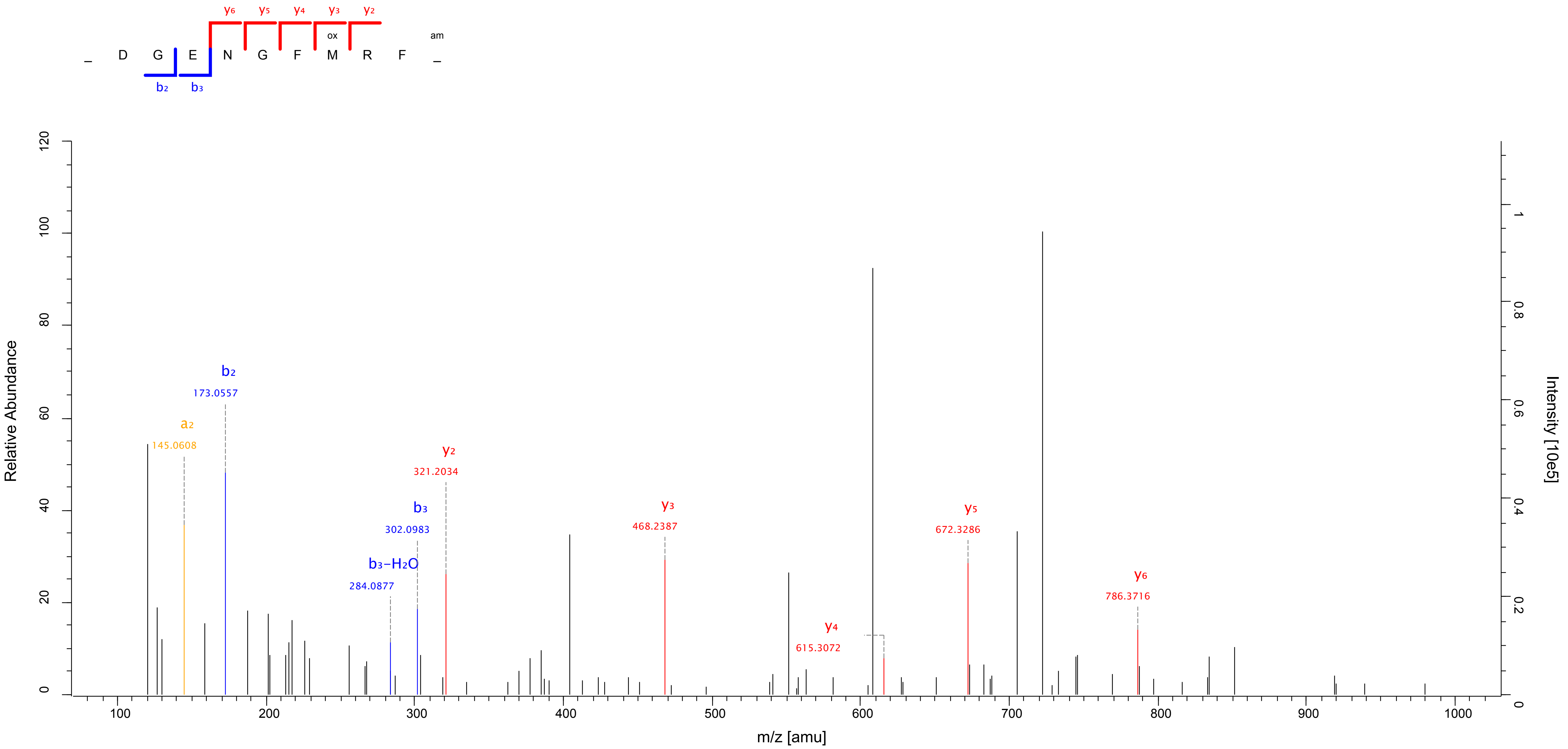

peptide ID 8

Source: 20120816_CO_0340GaJe_R02
Scannumber: 7730
Protein: SinglePep25
Peptide Score: 130.51
Method: FTMS; HCD; 1

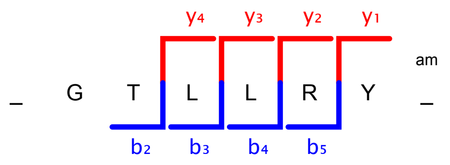

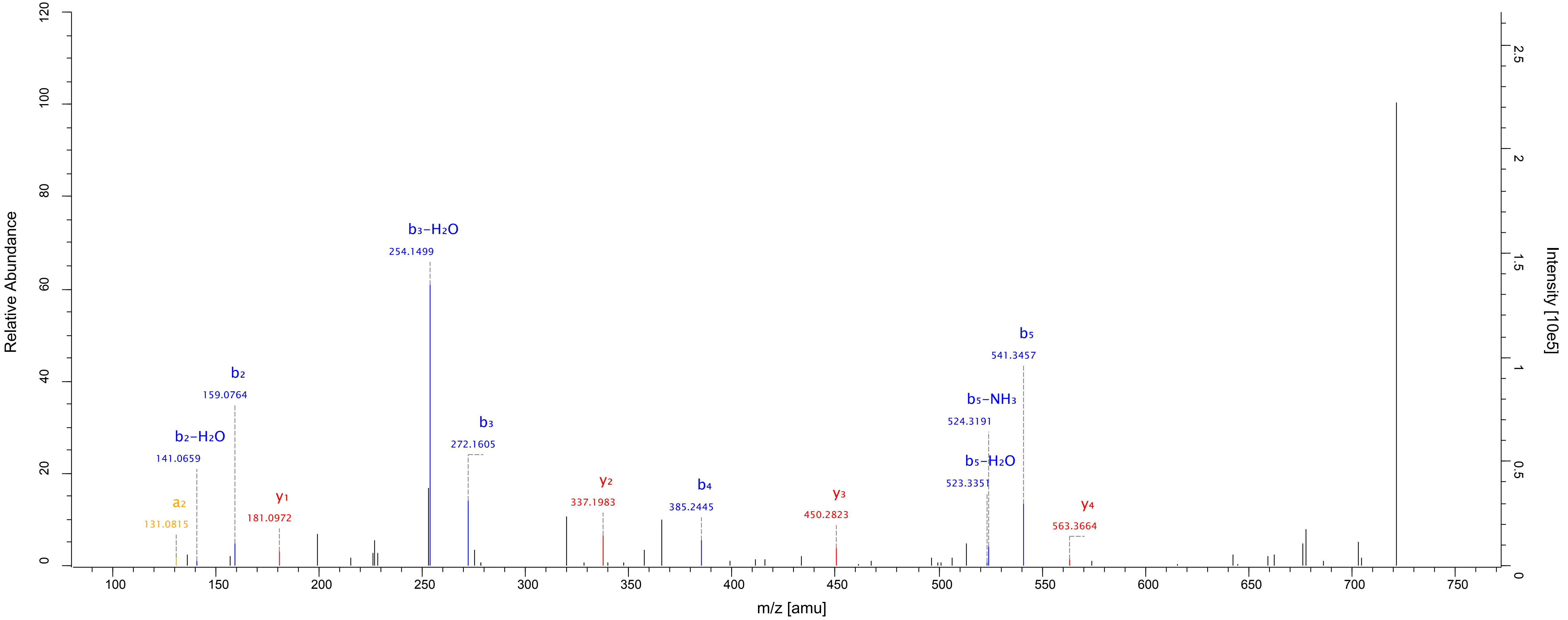

peptide ID 9

Source: 20120816_CO_0340GaJe_R02
Scannumber: 16365
Protein: pep_174; pep_secretome_23345; pep_secretome_479
Peptide Score: 78.69
Method: FTMS; HCD; 1

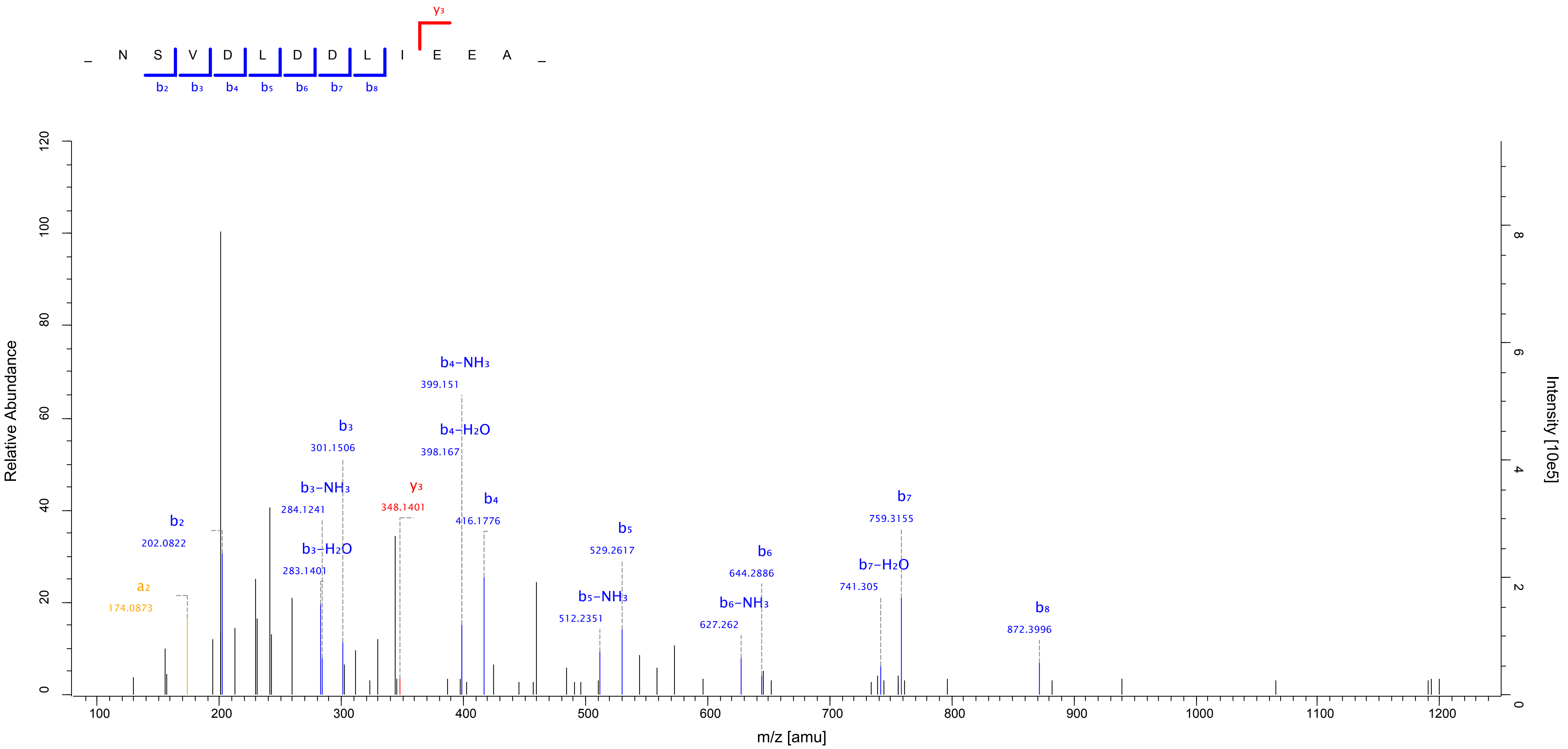

Source: 20120816_CO_0340GaJe_R02
Scannumber: 14110
Protein: orf_11768; orf_7289; pep_secretome_480
Peptide Score: 70.2
Method: FTMS; HCD; 1

peptide ID 10

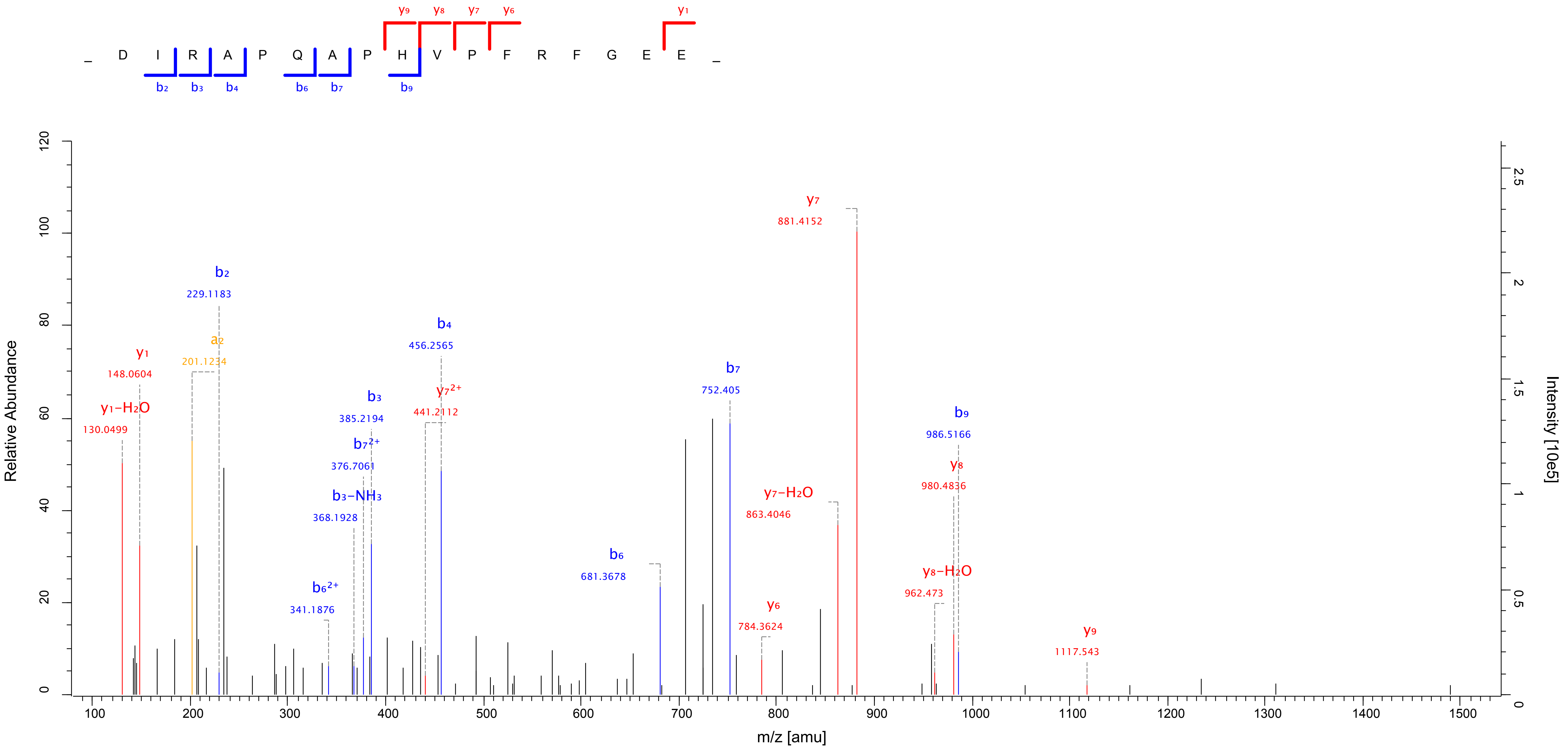

peptide ID 11

Source: 20120816_CO_0340GaJe_R02
Scannumber: 12502
Protein: orf_11768; orf_7289; pep_secretome_480
Peptide Score: 72.5
Method: FTMS; HCD; 1

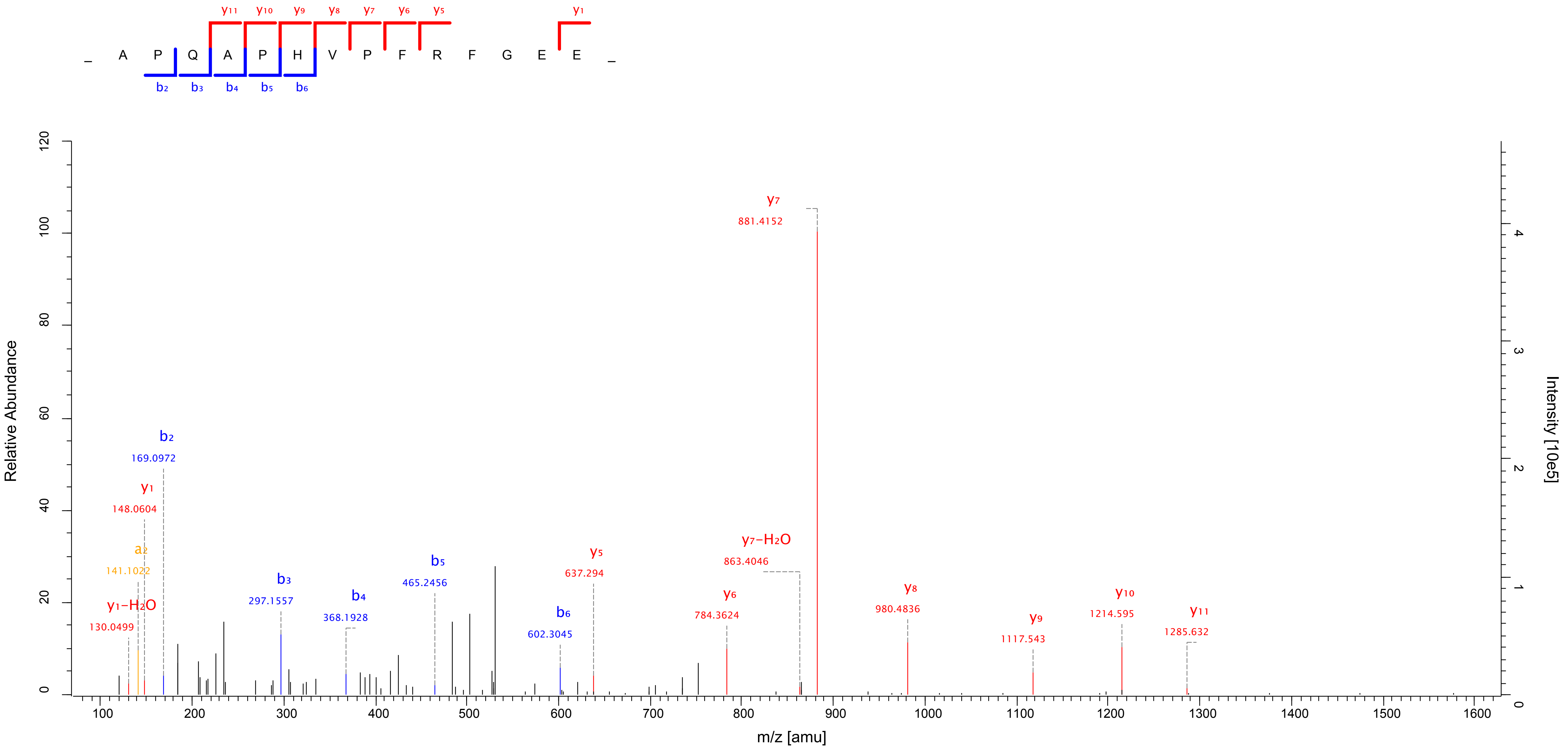

peptide ID 12

Source: 20120515_CO_0340GaJe_R01
Scannumber: 13383
Protein: pep_166; pep_secretome_451
Peptide Score: 73.47
Method: FTMS; HCD; 1

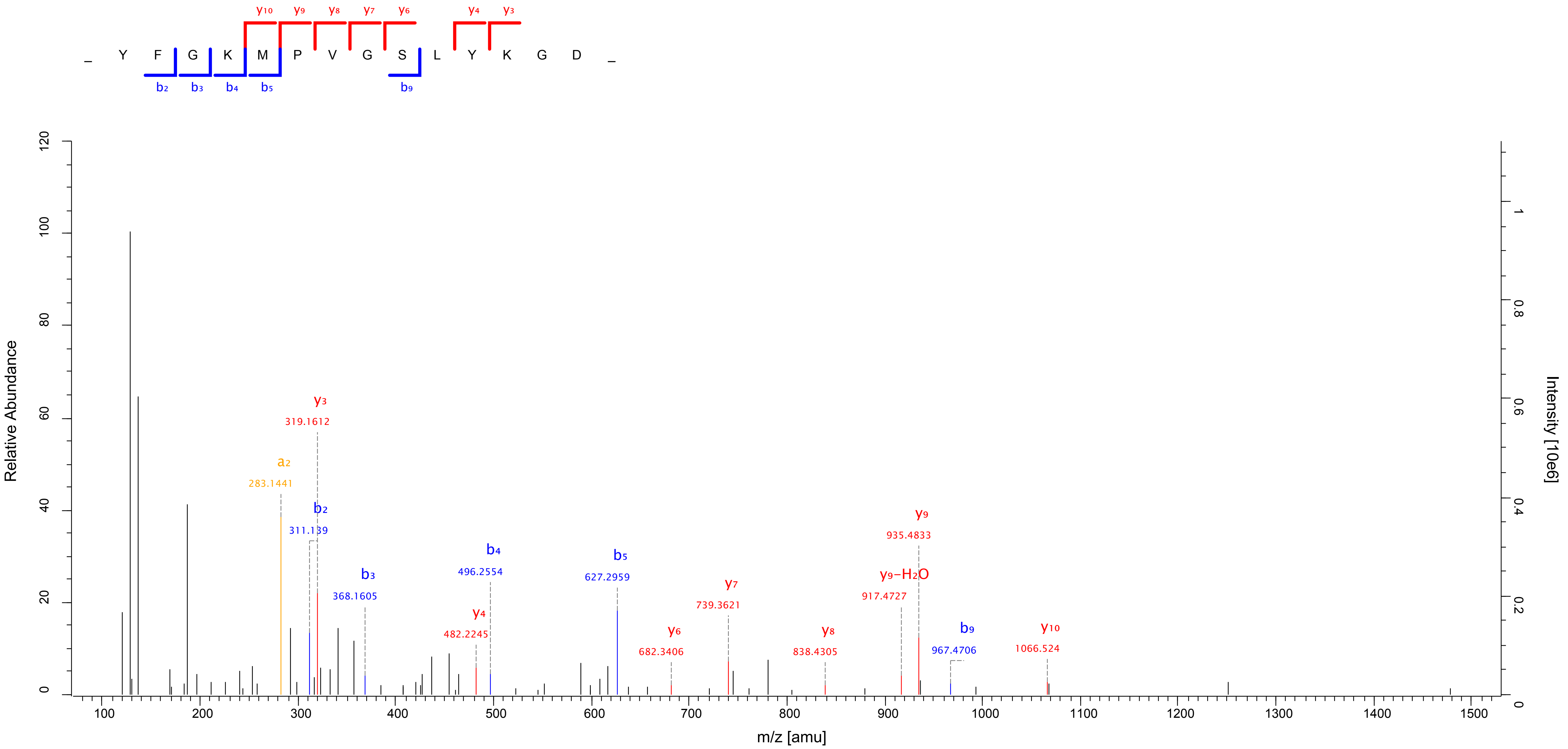

Source: 20121106_CO_0340GaJe_R02_2
Scannumber: 13490
Protein: pep_86; pep_secretome_205; pep_secretome_22883
Peptide Score: 90.73
Method: FTMS; HCD; 1

peptide ID 13

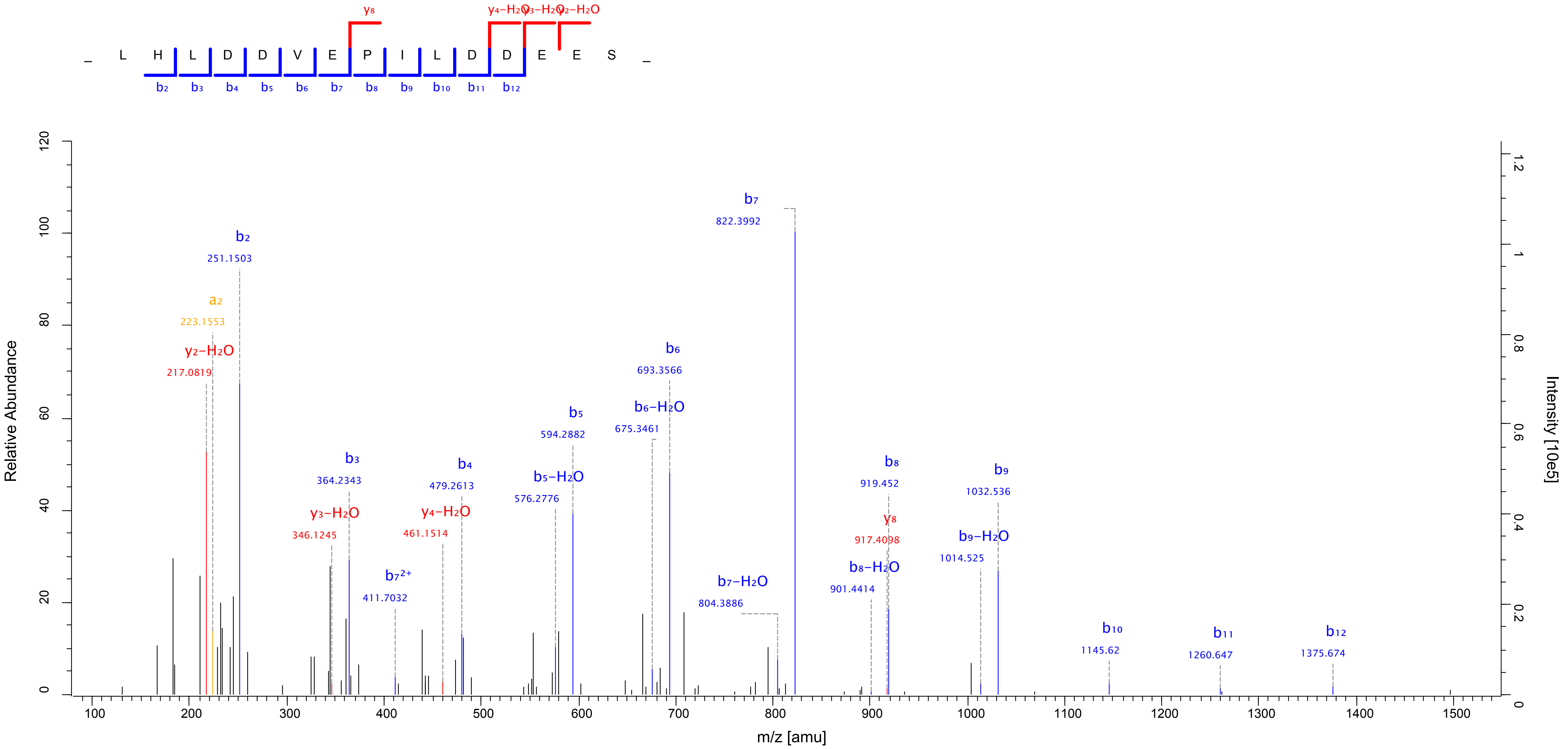

peptide ID 14

Source: 20120816_CO_0340GaJe_R02
Scannumber: 8498
Protein: pep_93; pep_secretome_213; pep_secretome_22908
Peptide Score: 85.81
Method: FTMS; HCD; 1

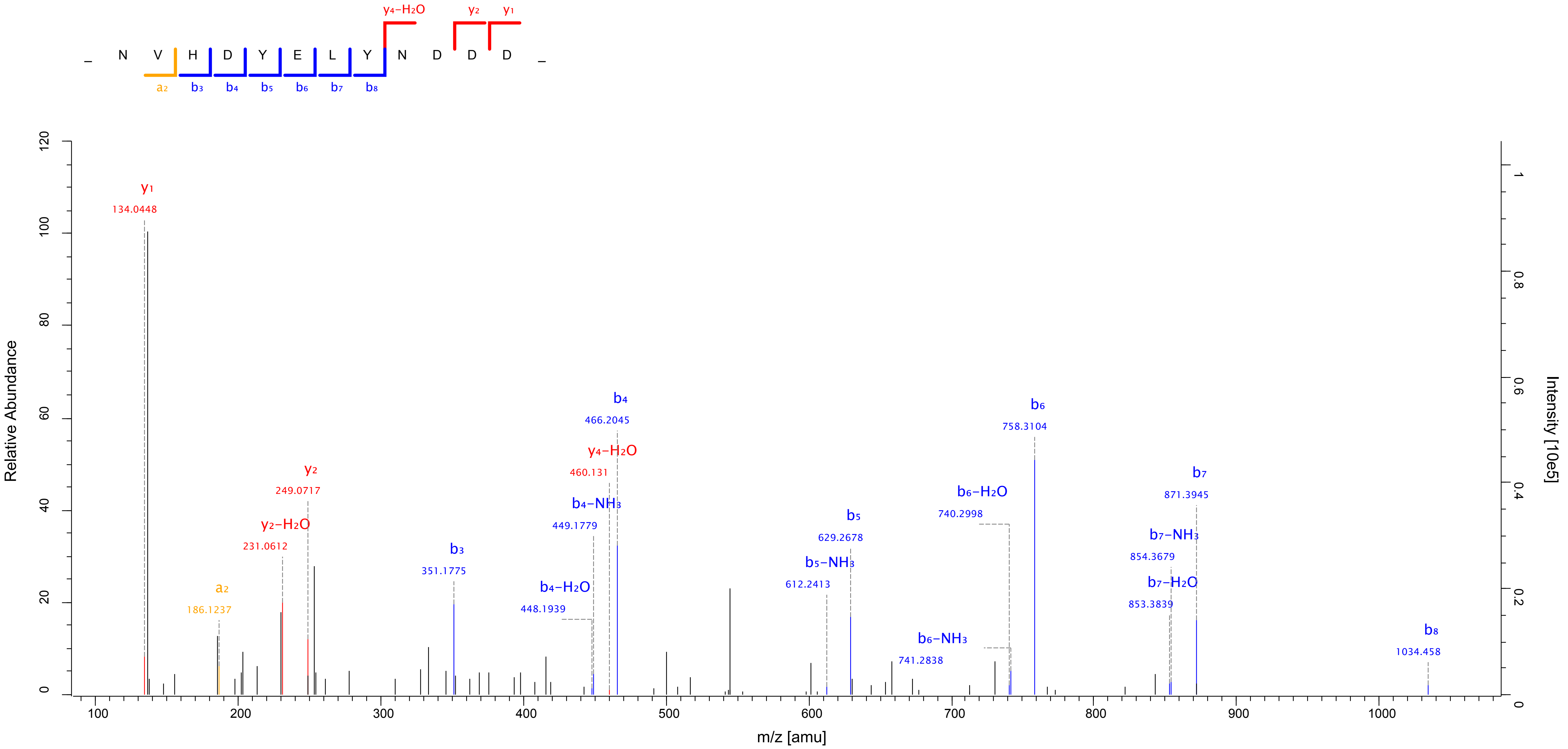

peptide ID 15

Source: 20121106_CO_0340GaJe_R02_2
Scannumber: 10723
Protein: pep_95; pep_secretome_216
Peptide Score: 85.38
Method: FTMS; HCD; 1

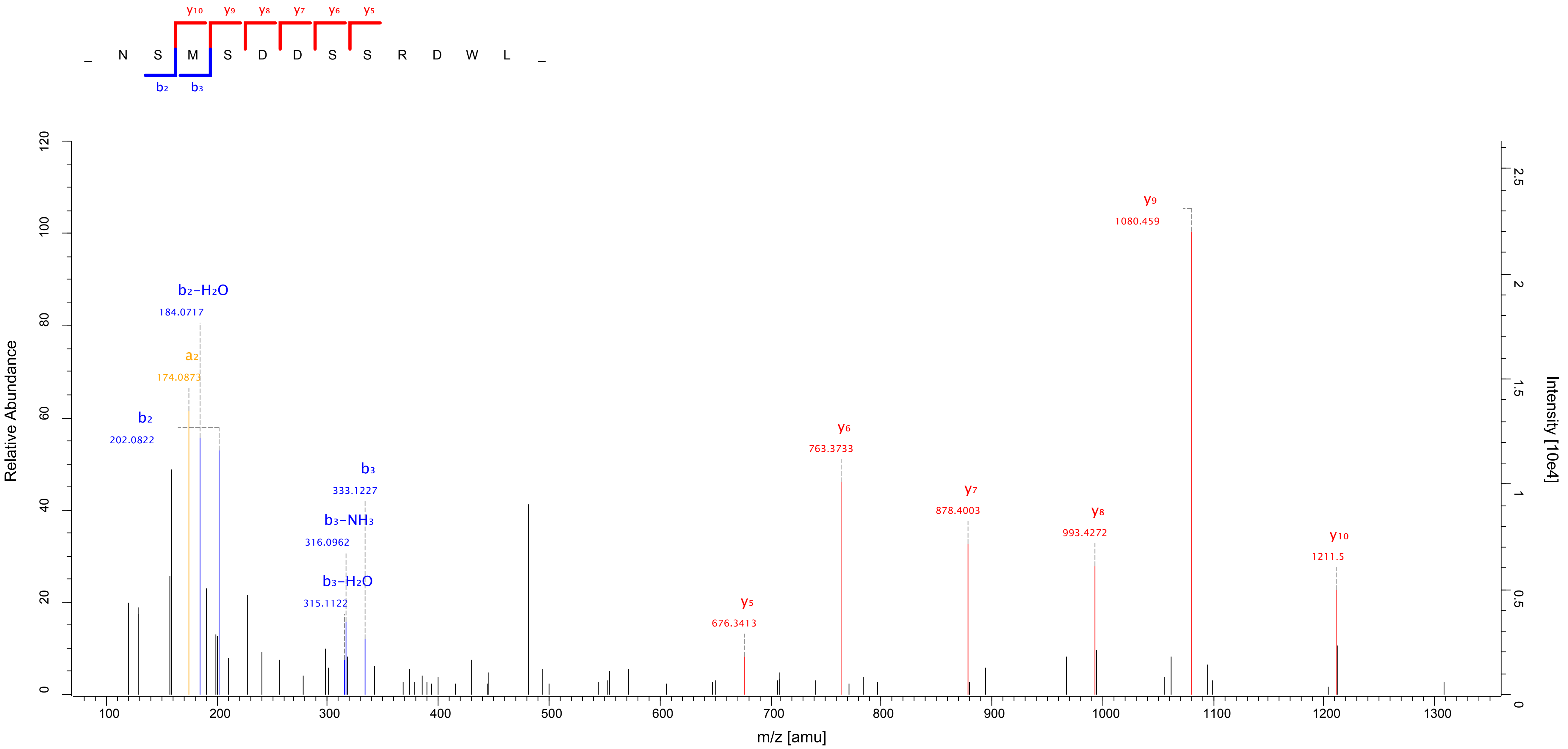

peptide ID 16

Source: 20120816_CO_0340GaJe_R02
Scannumber: 9950
Protein: pep_95; pep_secretome_216
Peptide Score: 79.97
Method: FTMS; HCD; 1

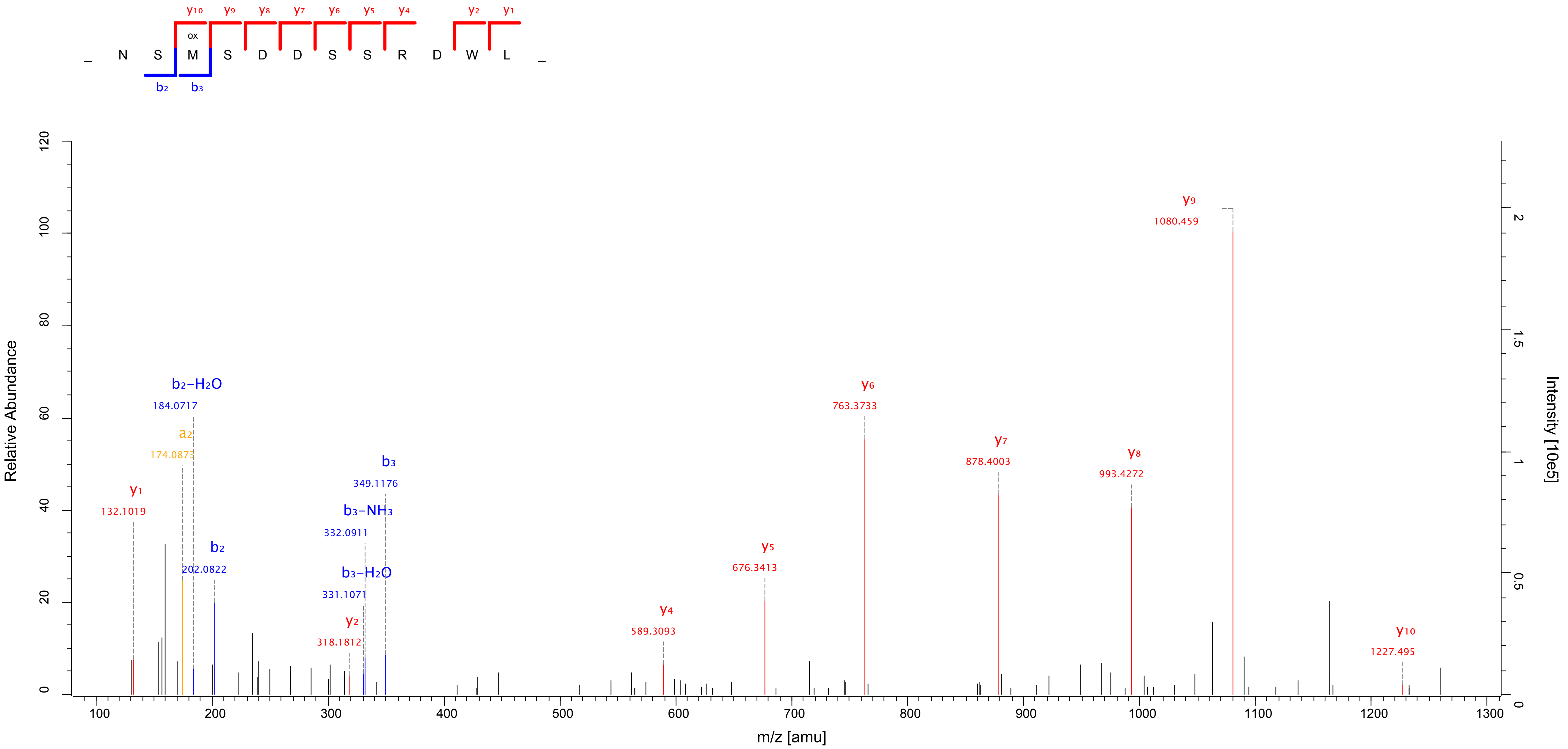

peptide ID 17

Source:	20120816_CO_0340GaJe_R02
Scannumber:	11791
Protein:	pep_100; pep_secretome_225; pep_secretome_22921
Peptide Score:	70.96
Method:	FTMS; HCD; 1

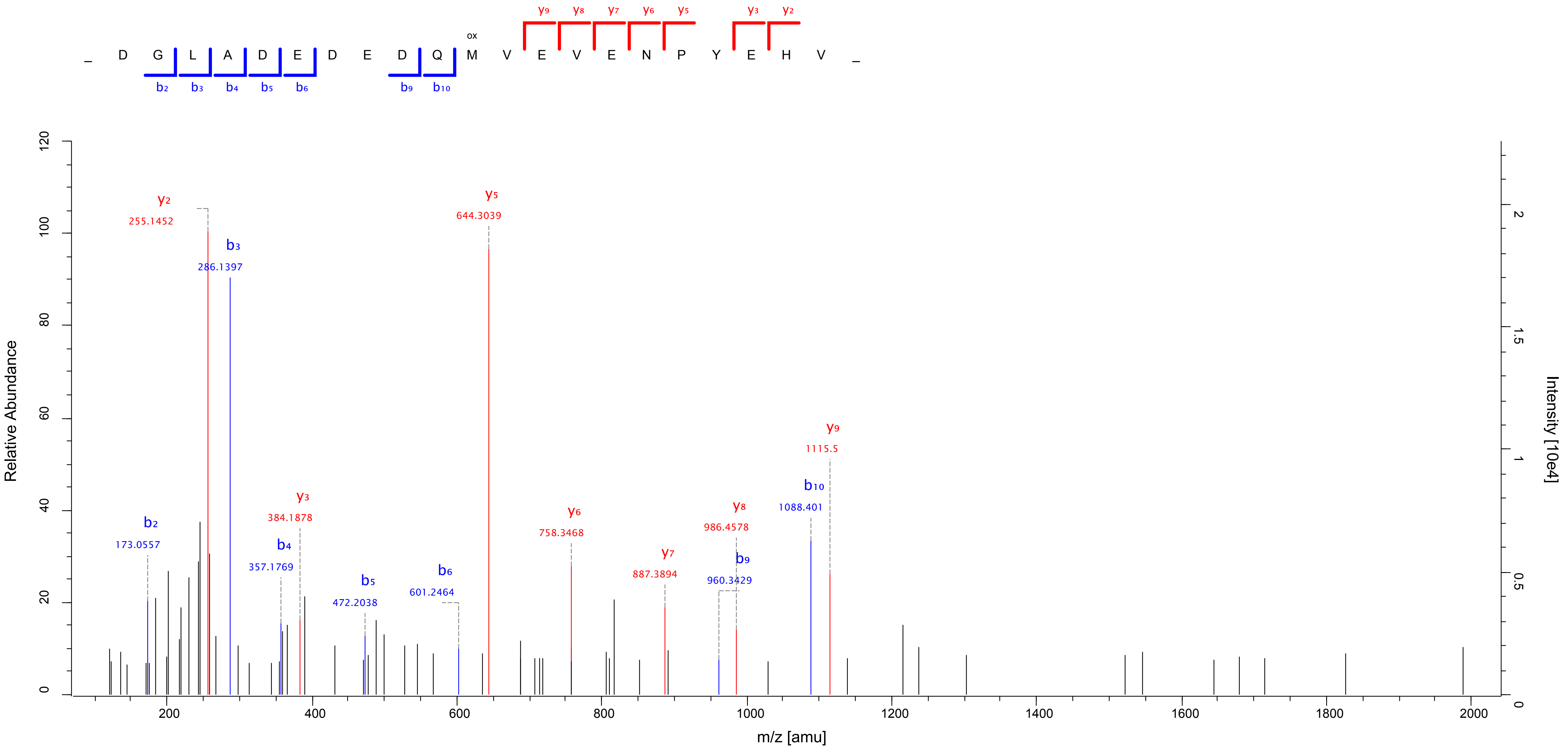

Source: 20120816_CO_0340GaJe_R02
Scannumber: 10815
Protein: pep_101; pep_secretome_226; pep_secretome_22922
Peptide Score: 87.77
Method: FTMS; HCD; 1

peptide ID 18

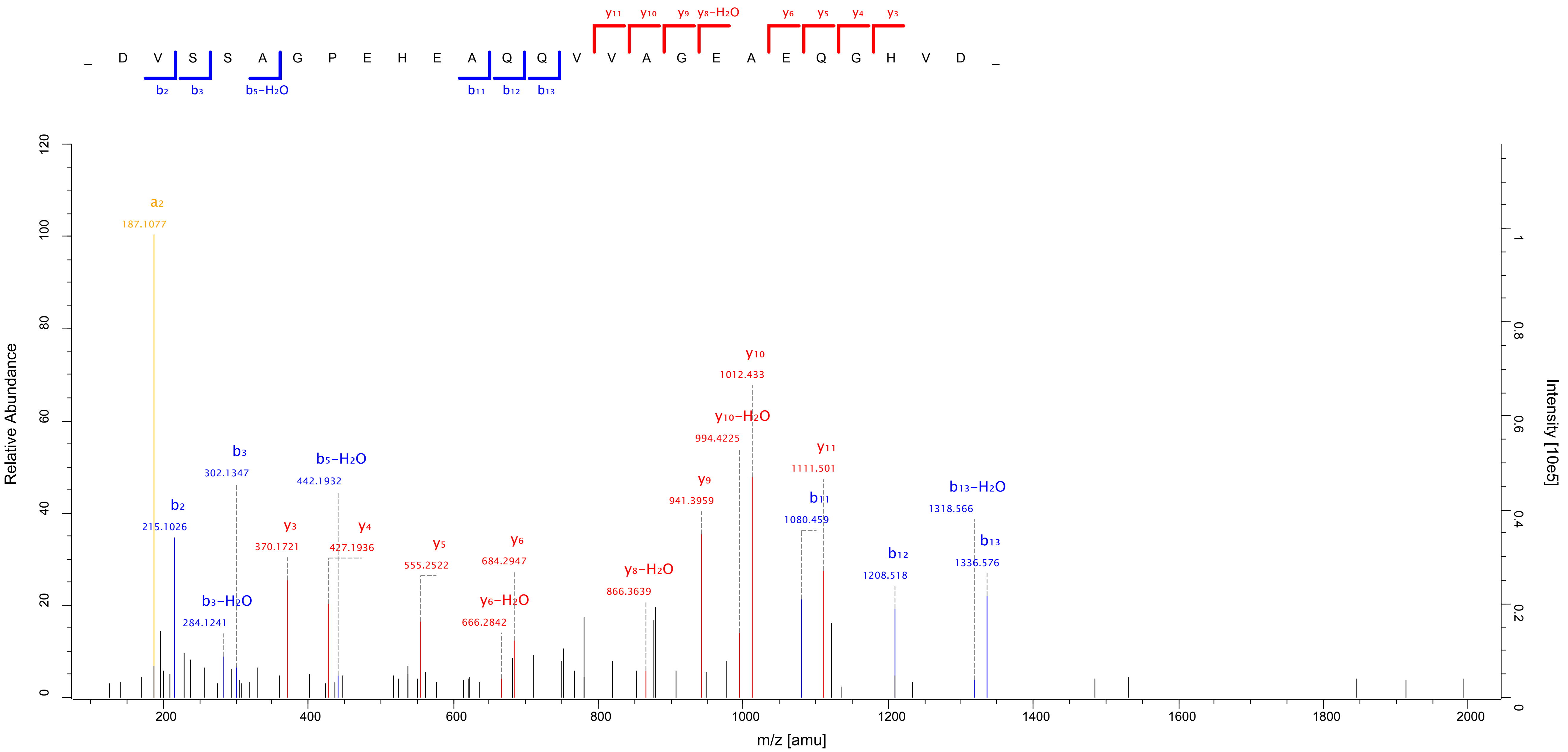

peptide ID 19

Source:	20120816_CO_0340GaJe_R02
Scannumber:	5682
Protein:	orf_3404; orf_7292; pep_secretome_261
Peptide Score:	65.72
Method:	FTMS; HCD; 1

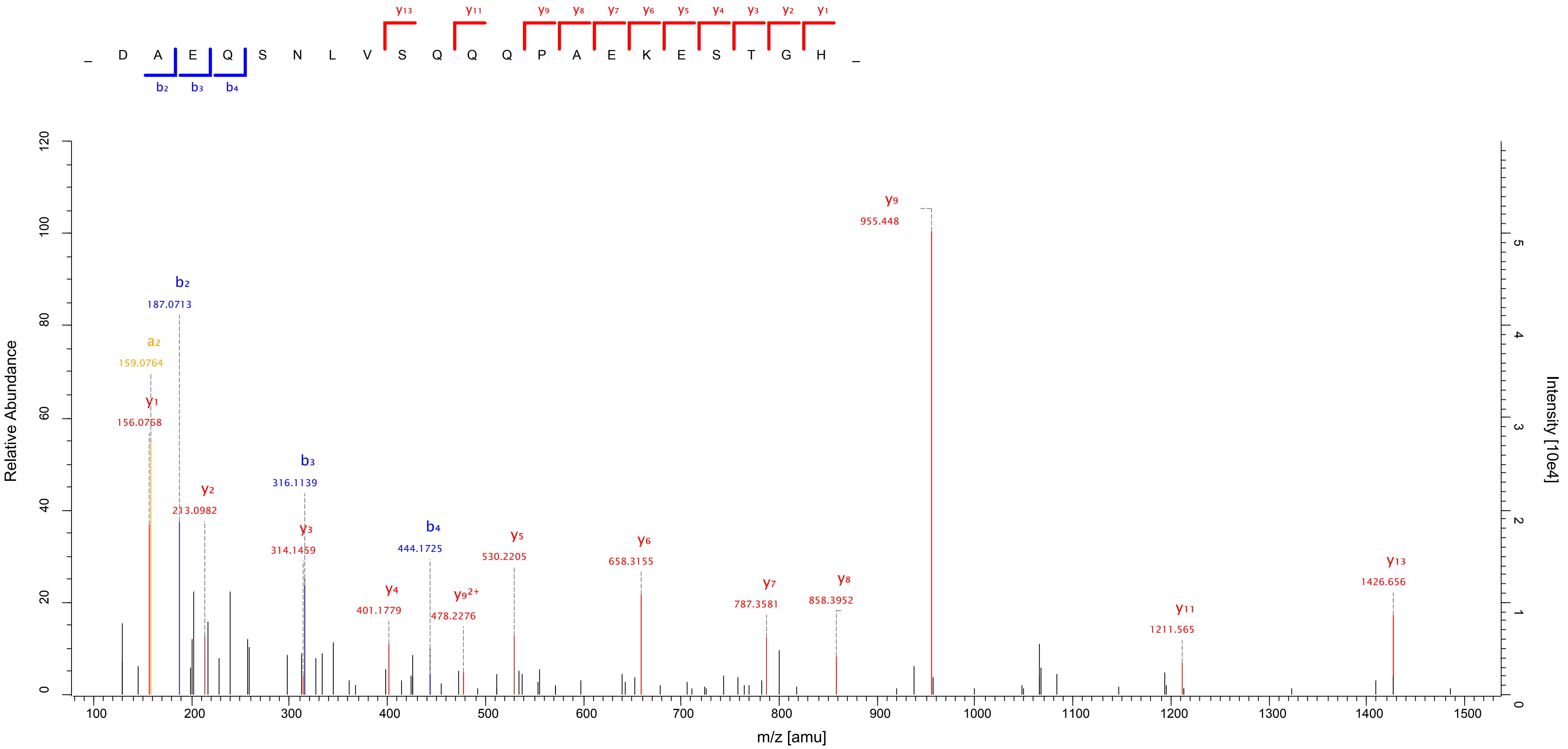

peptide ID 20

Source:	20120816_CO_0340GaJe_R02
Scannumber:	10708
Protein:	pep_128; pep_secretome_23090; pep_secretome_338
Peptide Score:	78.93
Method:	FTMS; HCD; 1

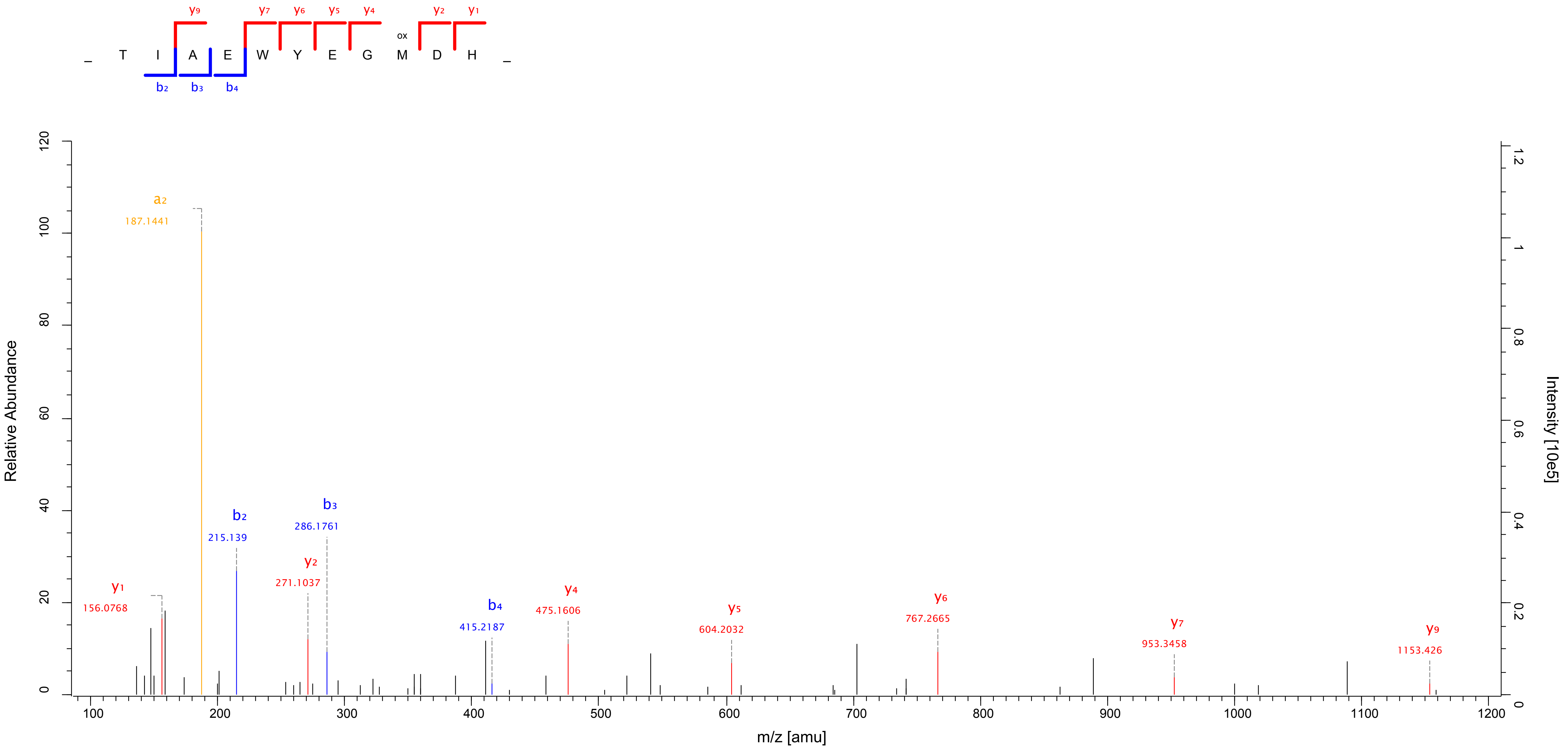

peptide ID 21

Source:	20120816_CO_0340GaJe_R02
Scannumber:	9645
Protein:	pep_83; pep_secretome_172; pep_secretome_22851
Peptide Score:	54.47
Method:	FTMS; HCD; 1

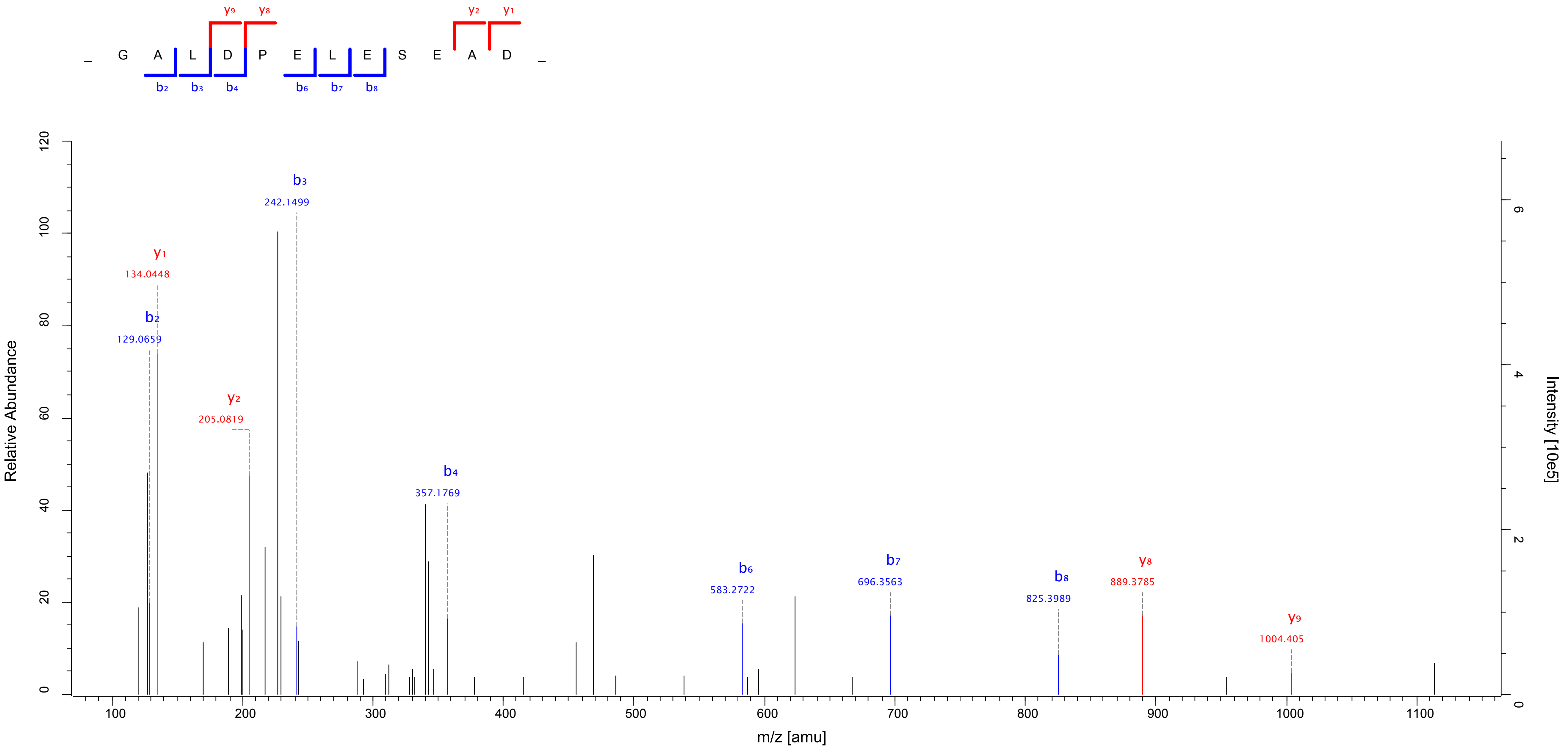

peptide ID 22

Source:	20121106_CO_0340GaJe_R02_2
Scannumber:	12611
Protein:	orf_11796; orf_7288
Peptide Score:	68.07
Method:	FTMS; HCD; 1

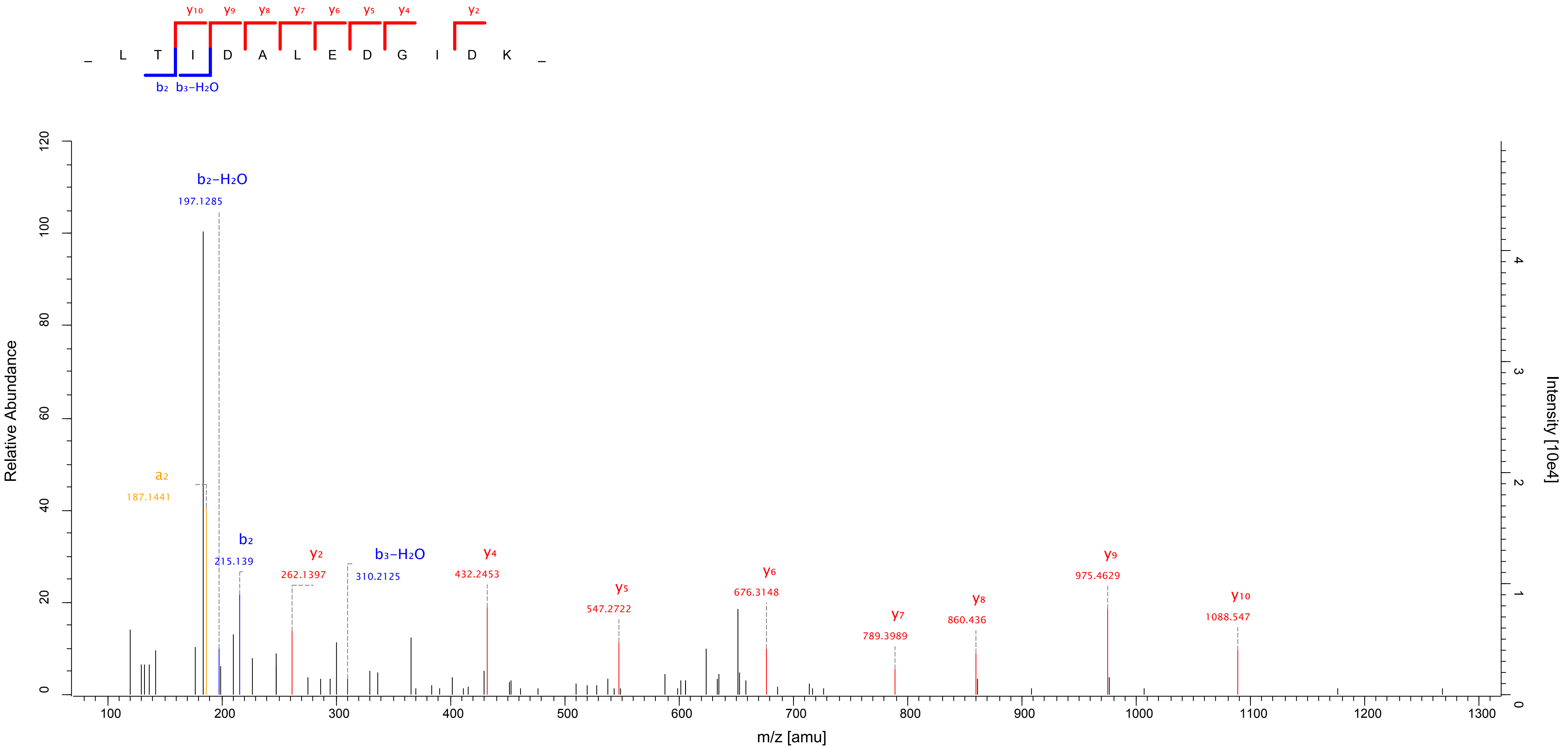

peptide ID 23

Source:	20120816_CO_0340GaJe_R02
Scannumber:	16701
Protein:	pep_8; pep_secretome_12; SinglePep63
Peptide Score:	70.73
Method:	FTMS; HCD; 1

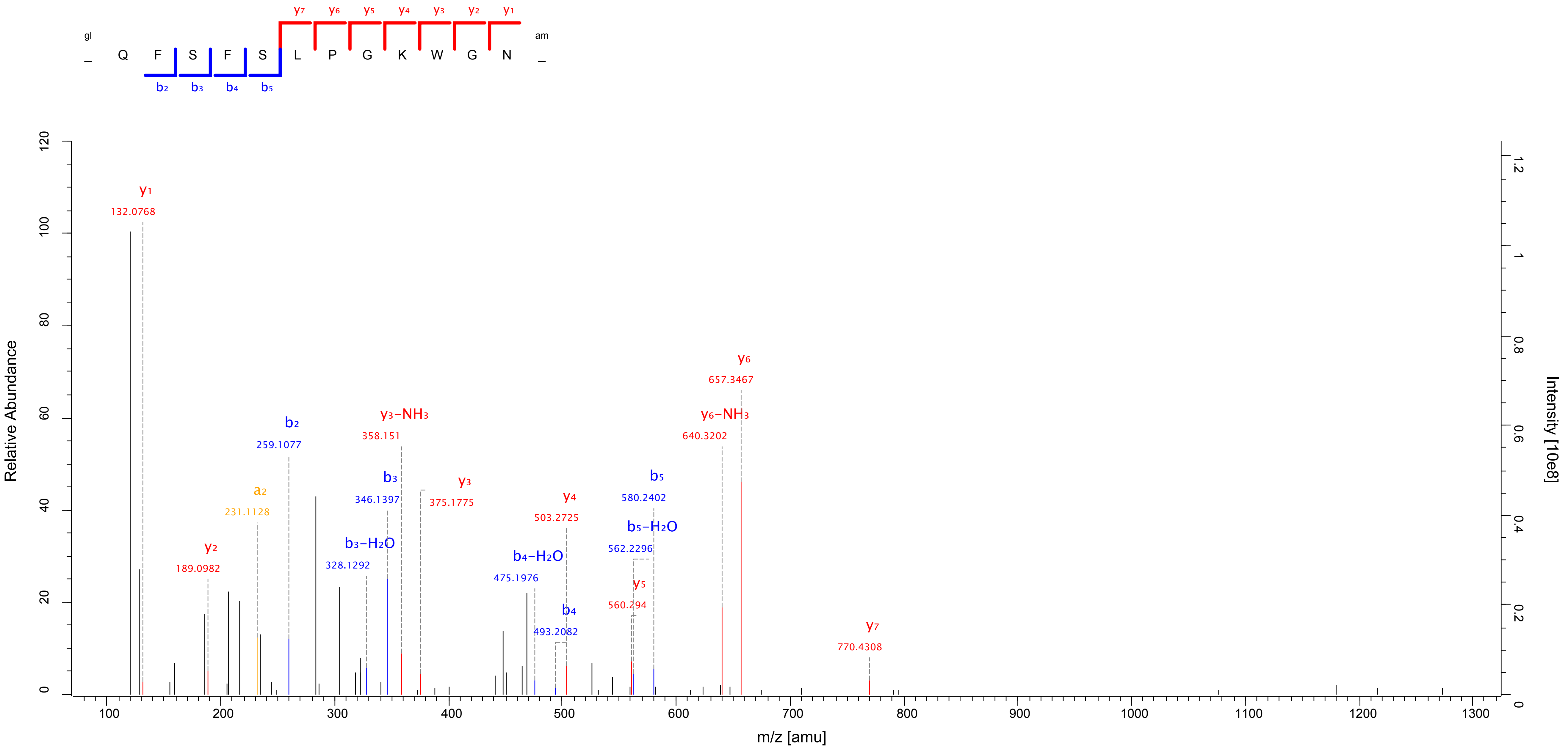

peptide ID 24

Source:	20120515_CO_0340GaJe_R01
Scannumber:	21023
Protein:	pep_74; pep_secretome_152
Peptide Score:	71.34
Method:	FTMS; HCD; 1

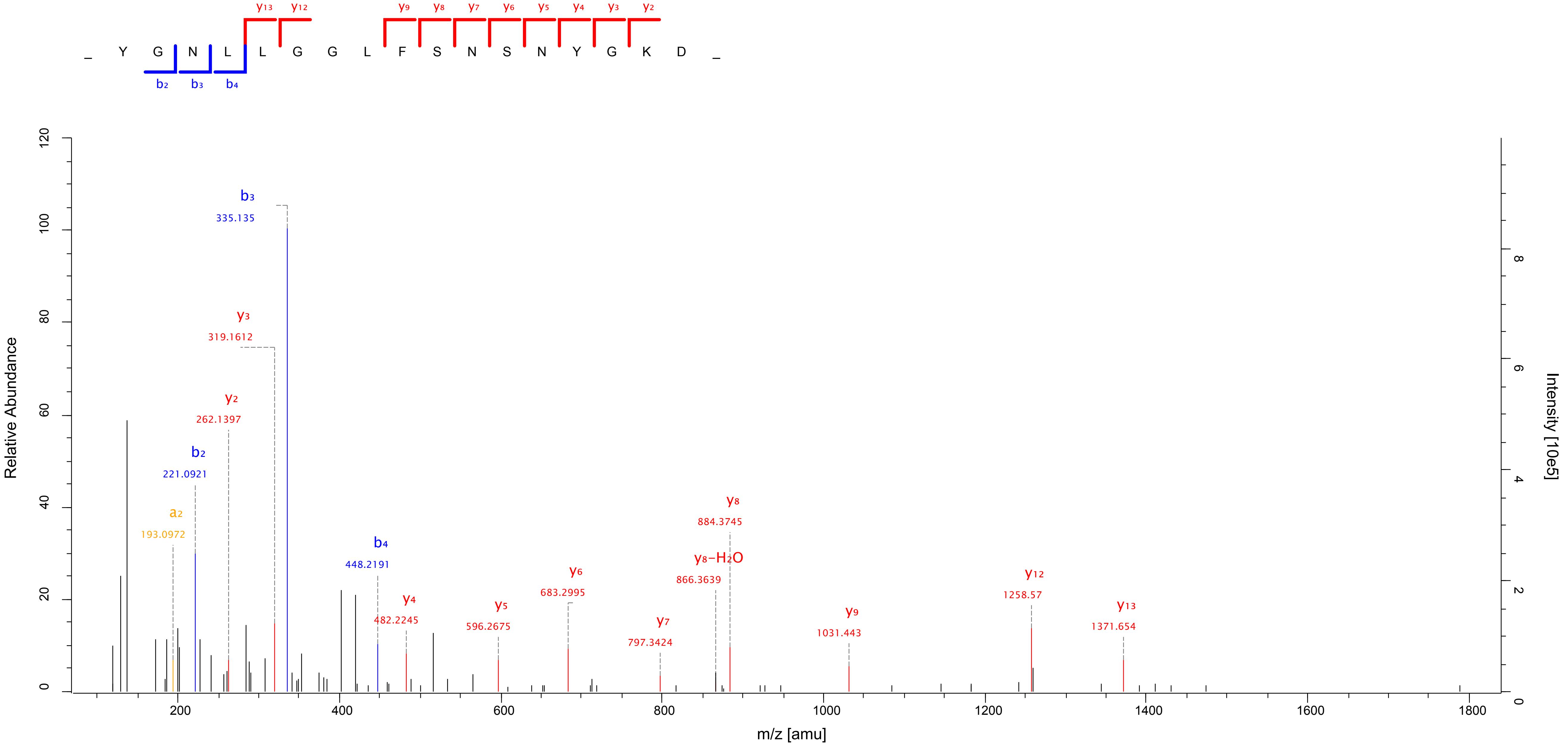

peptide ID 25

Source: 20121106_CO_0340GaJe_R02_2
Scannumber: 10053
Protein: pep_75; pep_secretome_153
Peptide Score: 92.19
Method: FTMS; HCD; 1

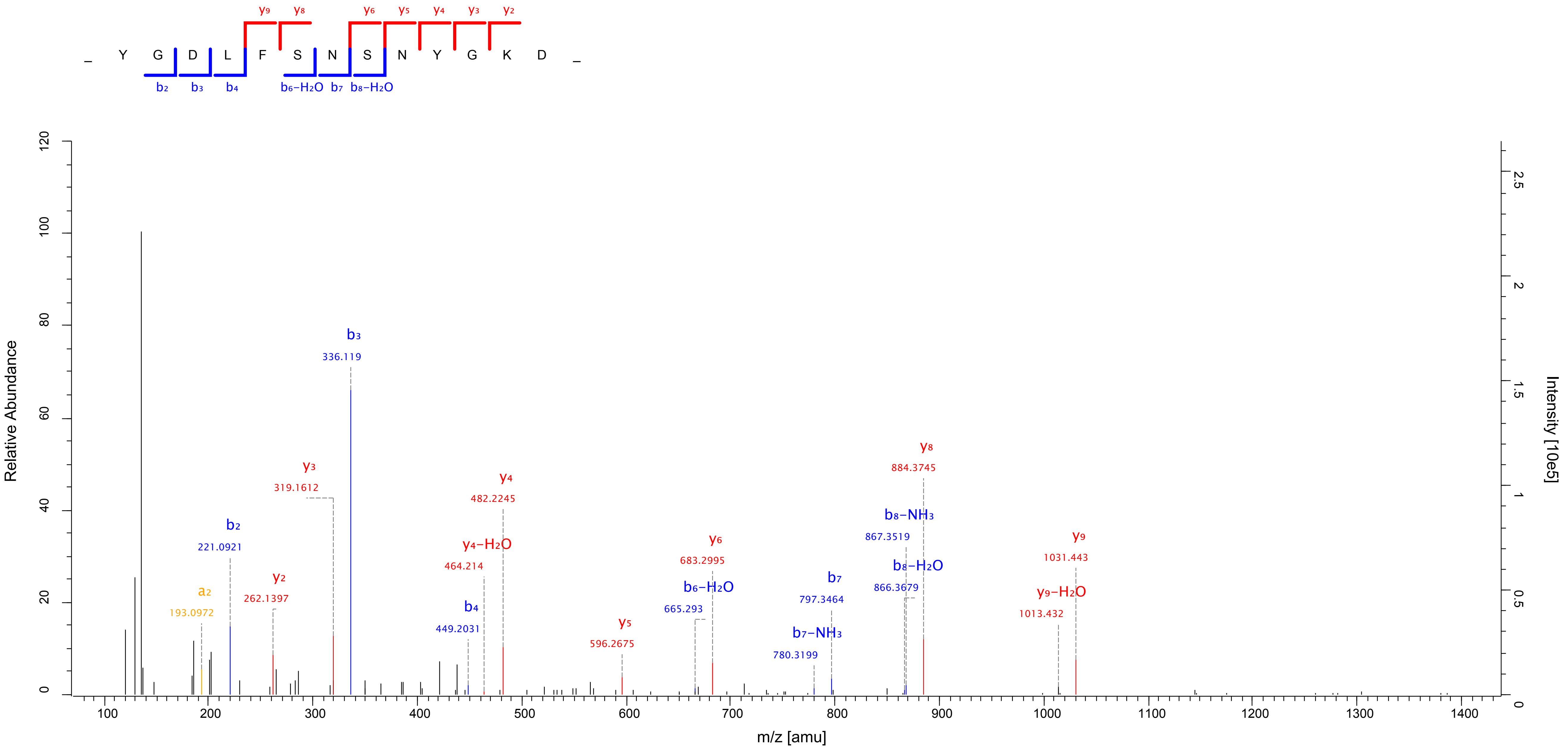

peptide ID 26

Source: 20121106_CO_0340GaJe_R02_2
Scannumber: 8619
Protein: pep_76; pep_secretome_154
Peptide Score: 78.69
Method: FTMS; HCD; 1

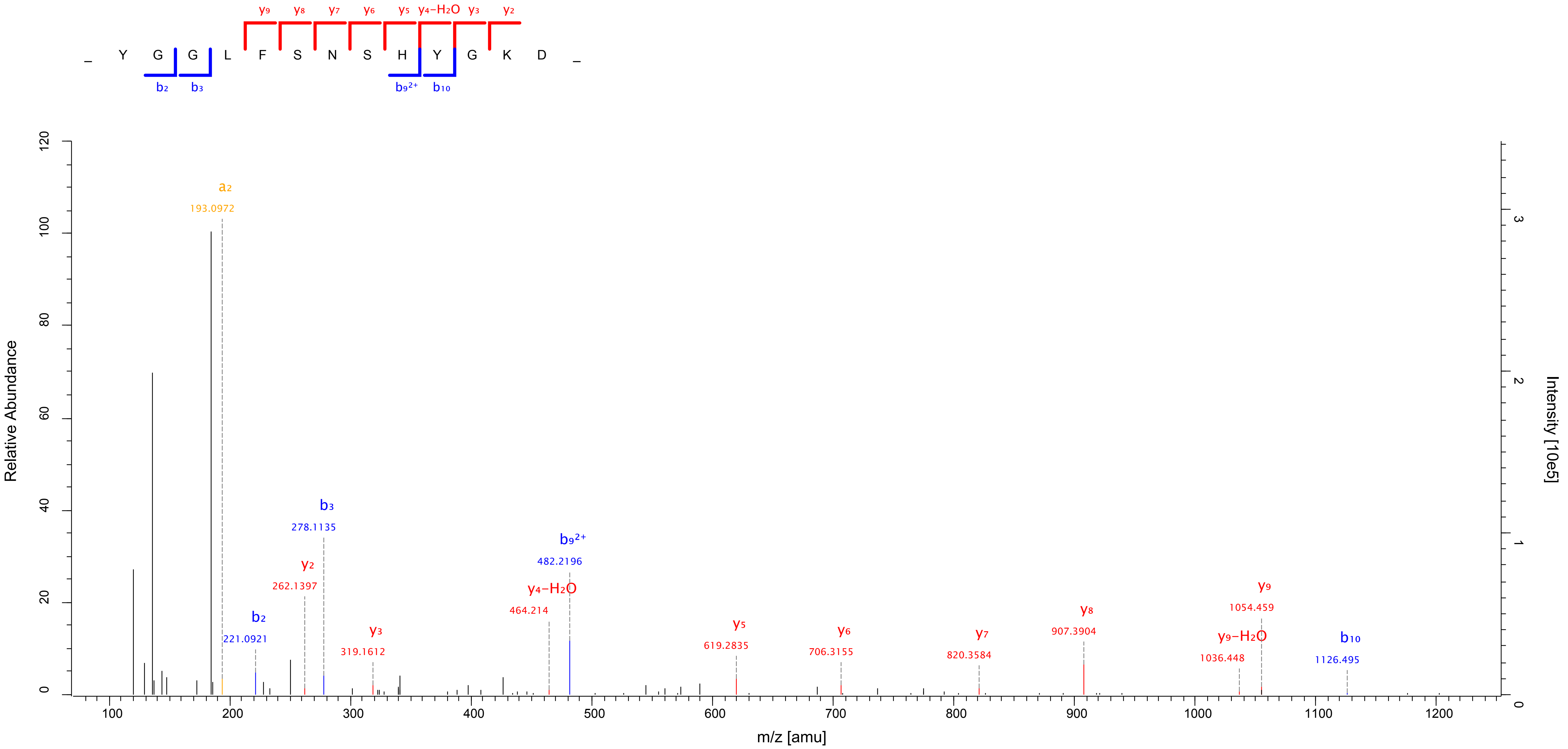

peptide ID 27

Source:	20120816_CO_0340GaJe_R02
Scannumber:	16470
Protein:	orf_7287; orf_6583; orf_1494; pep_77; pep_secretome_155
Peptide Score:	113.63
Method:	FTMS; HCD; 1

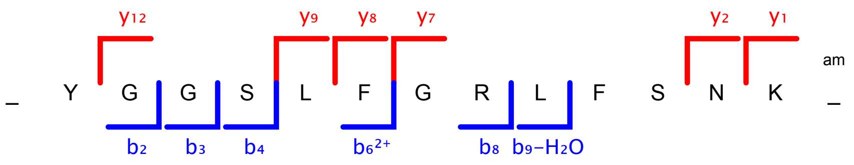

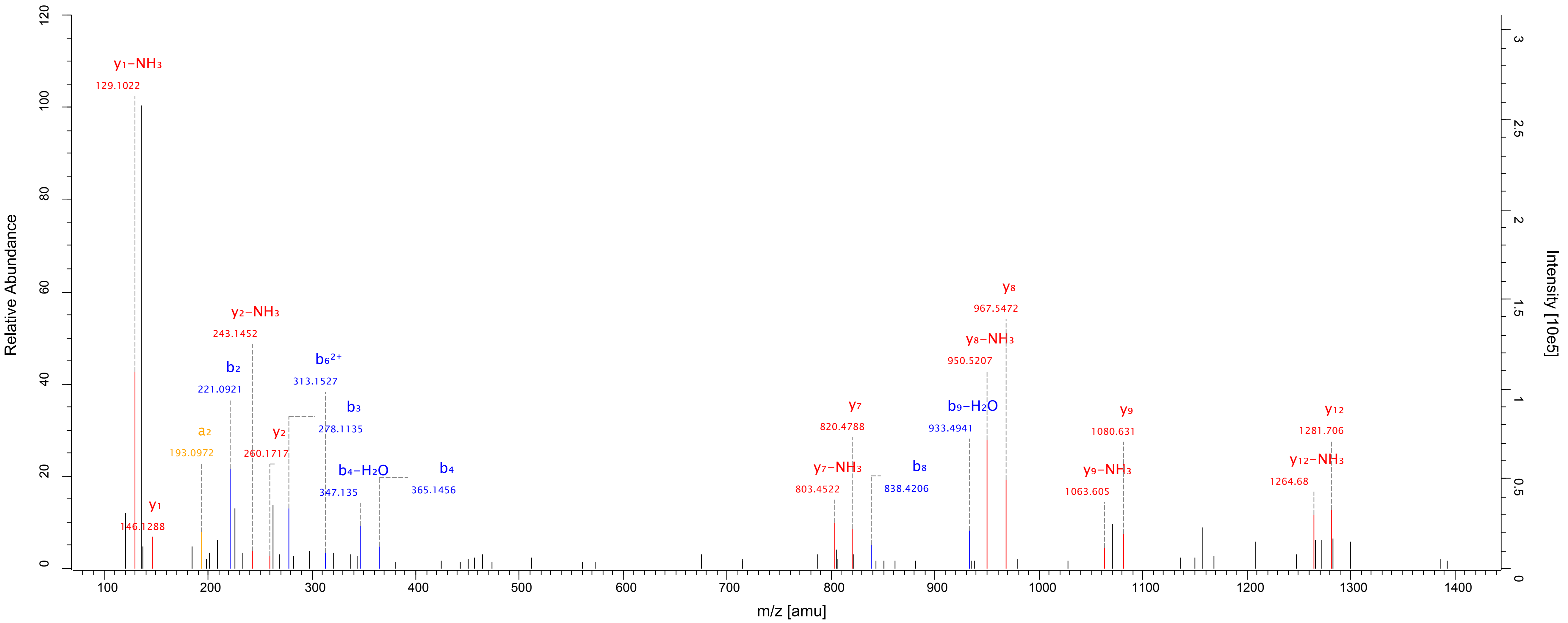

peptide ID 28

Source: 20120816_CO_0340GaJe_R02
Scannumber: 15205
Protein: pep_78; pep_secretome_156
Peptide Score: 70.62
Method: FTMS; HCD; 1

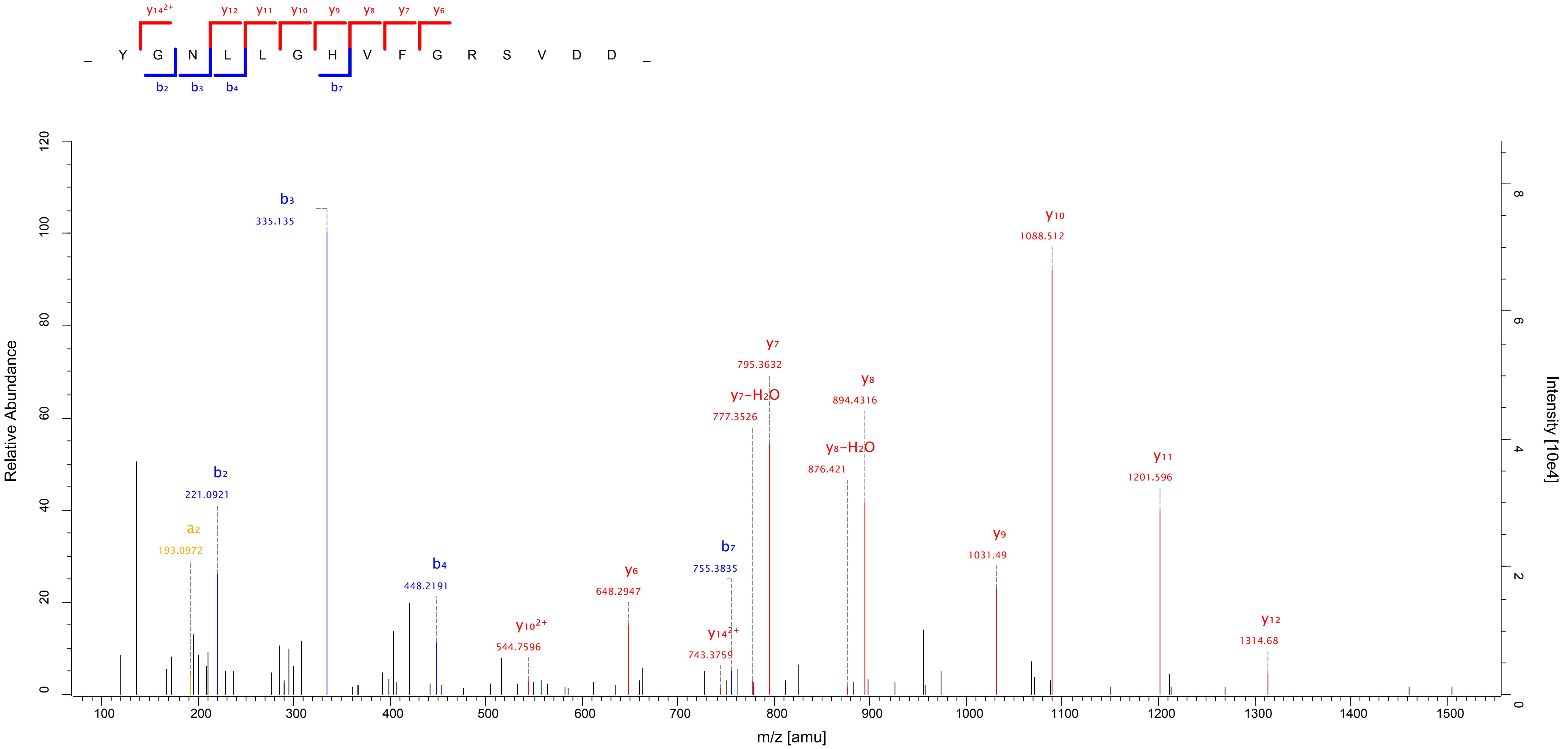

peptide ID 29

Source: 20120515_CO_0340GaJe_R01
Scannumber: 16424
Protein: orf_7287; orf_6583; orf_8467; orf_1494; orf_31538
Peptide Score: 81.43
Method: FTMS; HCD; 1

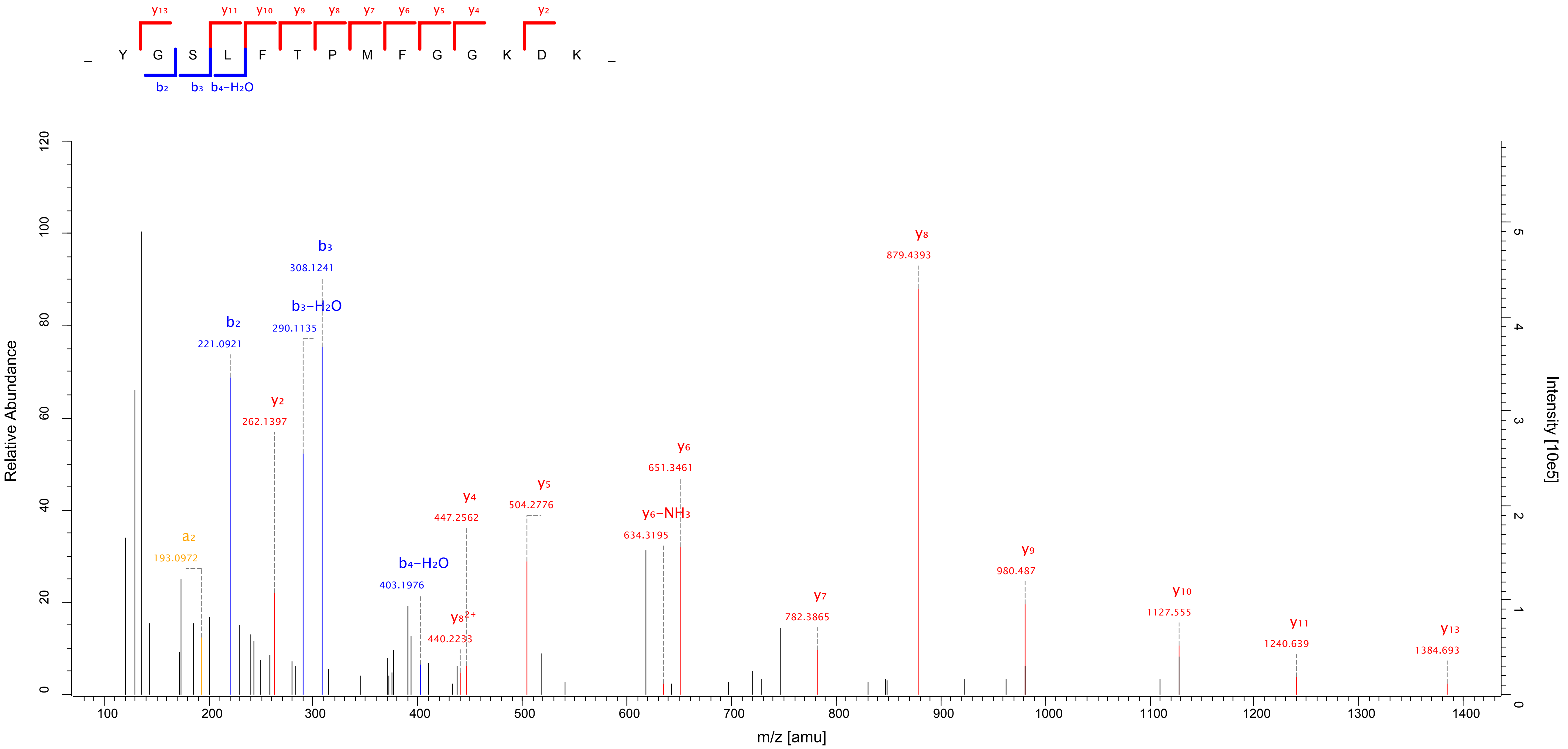

peptide ID 30

Source: 20120816_CO_0340GaJe_R02
Scannumber: 12518
Protein: orf_31538
Peptide Score: 61.38
Method: FTMS; HCD; 1

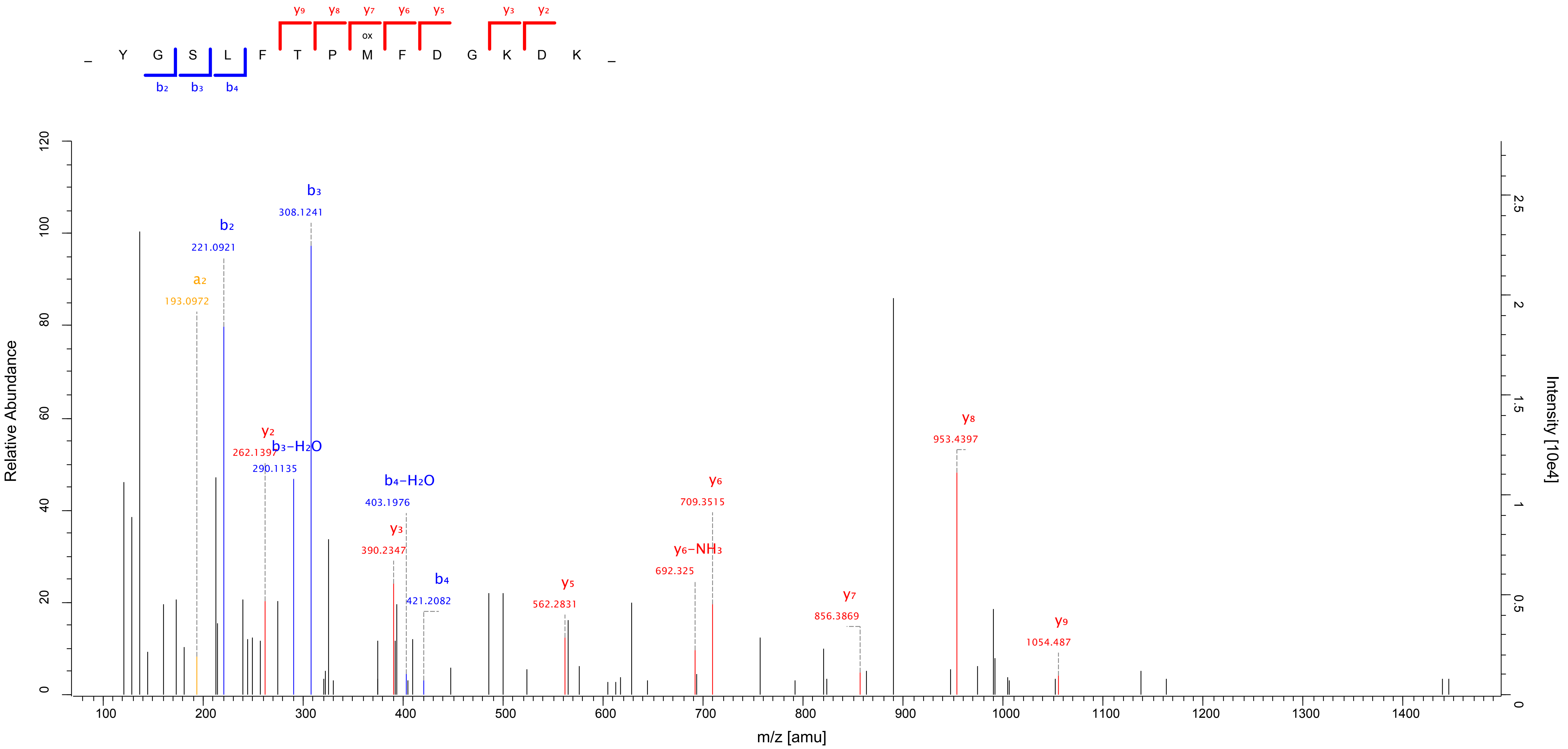

Source: 20121106_CO_0340GaJe_R02_2
Scannumber: 12572
Protein: pep_79; pep_secretome_159; pep_secretome_160; pep_secretome_161; pep_secretome_162
Peptide Score: 91.31
Method: FTMS; HCD; 1

peptide ID 31

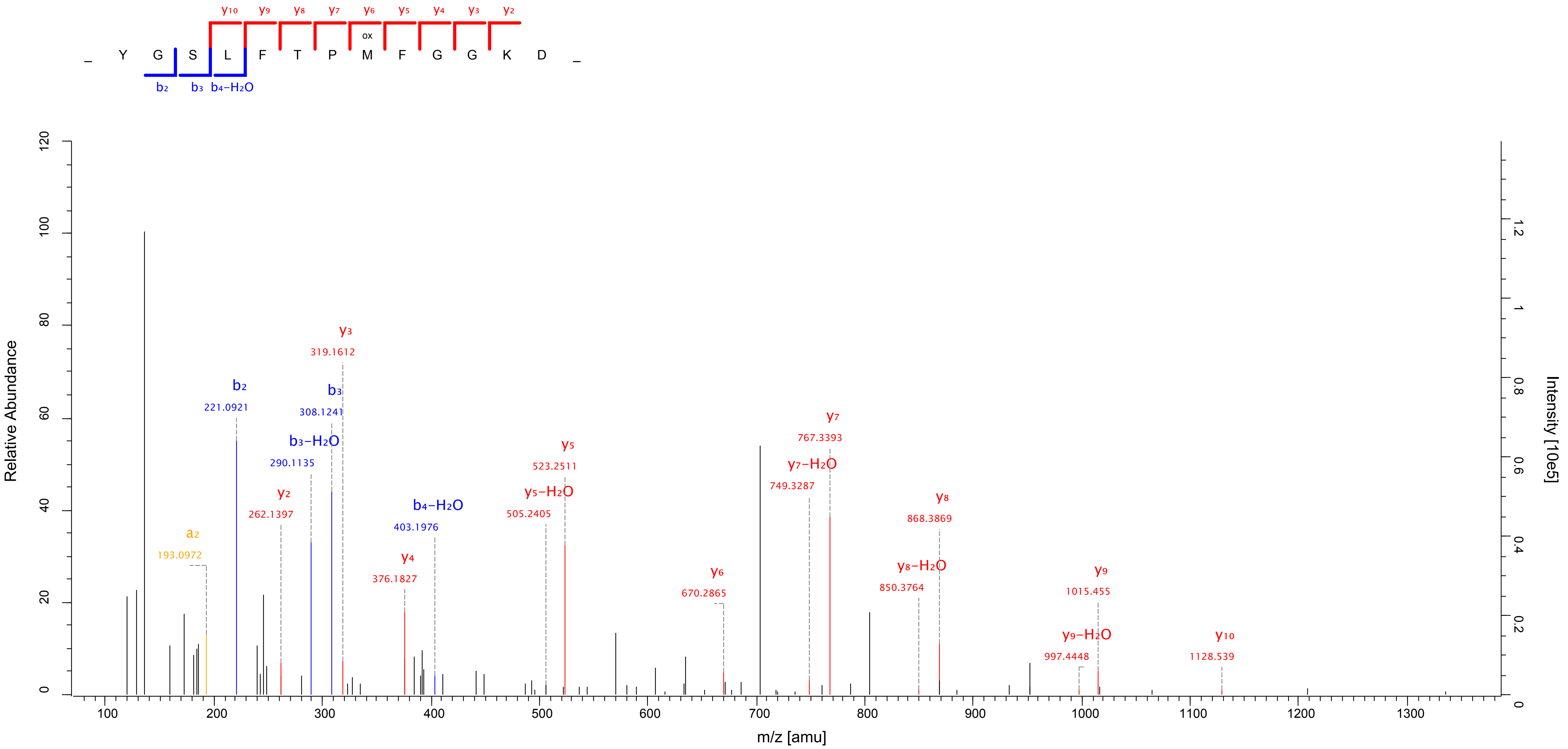

peptide ID 32

Source: 20121106_CO_0340GaJe_R02_2
Scannumber: 14976
Protein: pep_79; pep_secretome_159; pep_secretome_160; pep_secretome_161; pep_secretome_162
Peptide Score: 79.82
Method: FTMS; HCD; 1

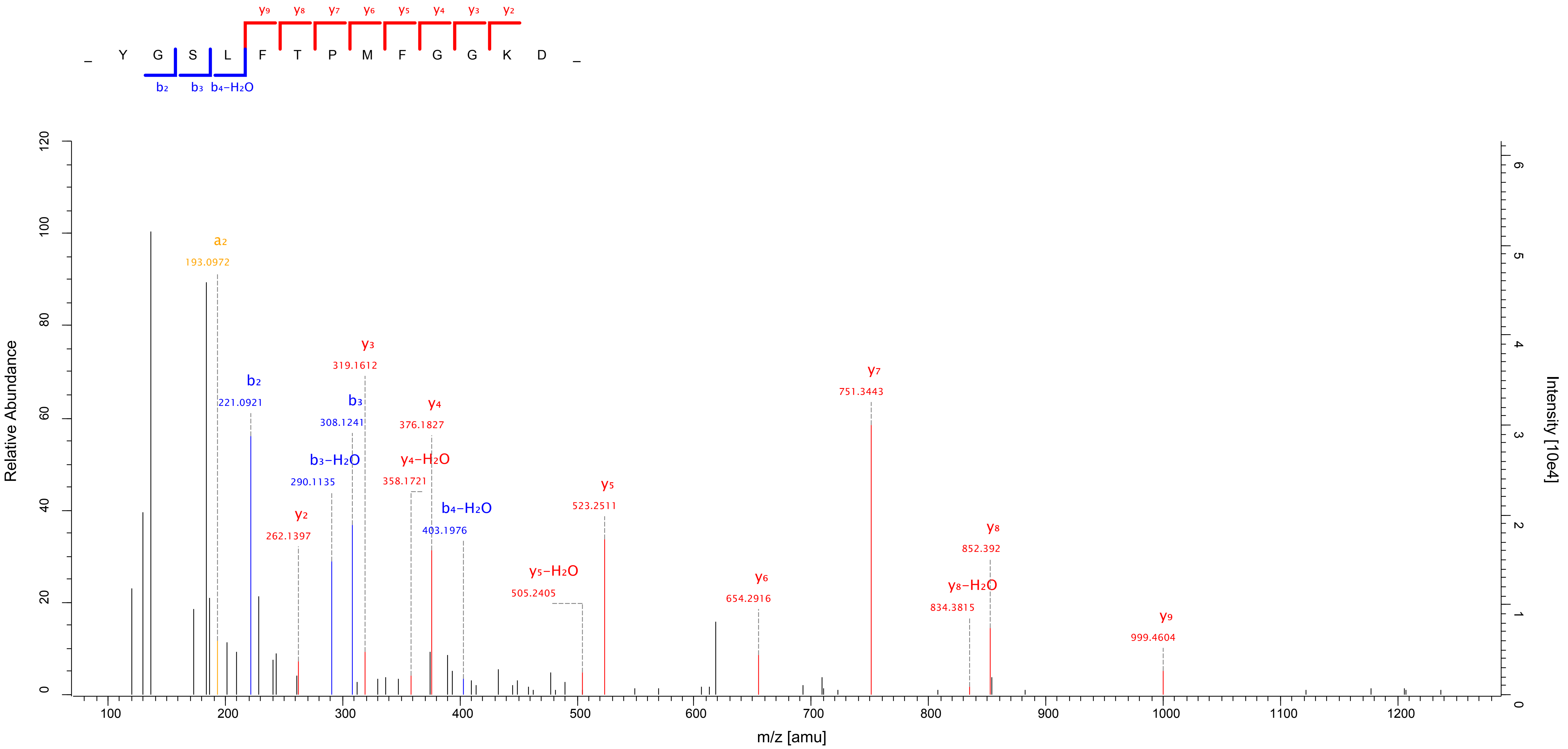

peptide ID 33

Source:	20120515_CO_0340GaJe_R01
Scannumber:	17895
Protein:	orf_7287; pep_80; pep_secretome_164
Peptide Score:	128.81
Method:	FTMS; HCD; 1

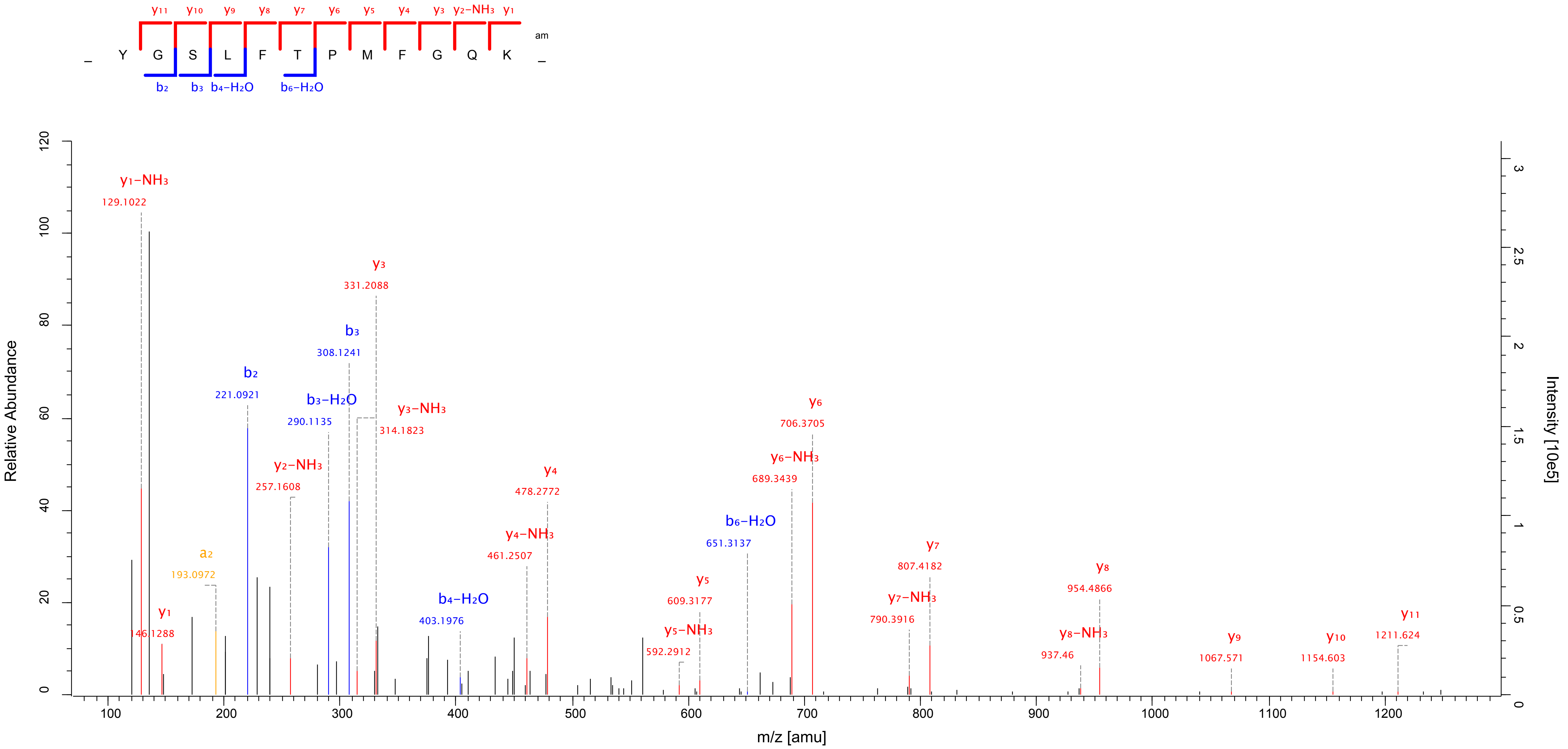

peptide ID 34

Source: 20120816_CO_0340GaJe_R02
Scannumber: 13419
Protein: orf_7287; pep_80; pep_secretome_164
Peptide Score: 66.92
Method: FTMS; HCD; 1

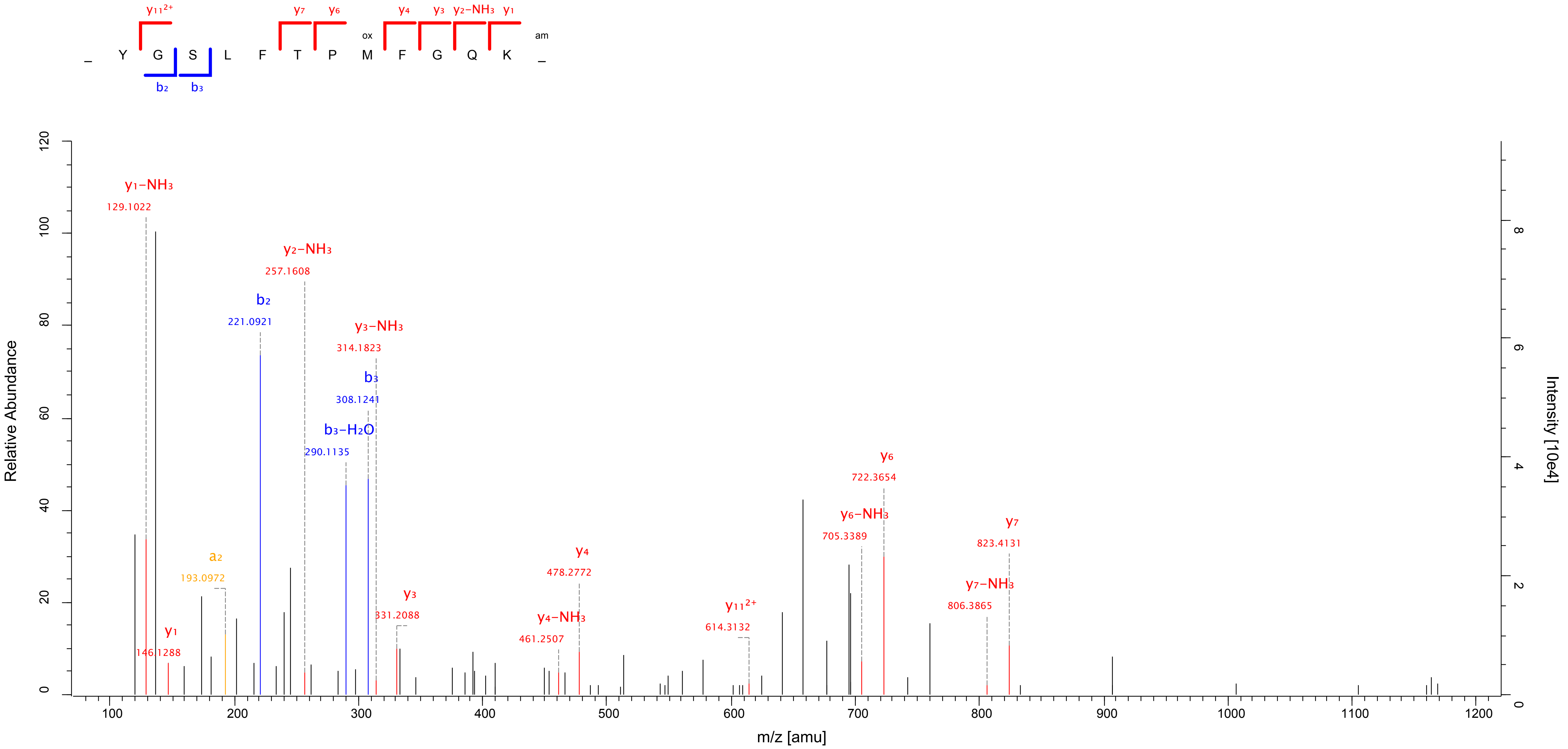

Source: 20120816_CO_0340GaJe_R02
Scannumber: 10065
Protein: pep_81; pep_secretome_165
Peptide Score: 40.8
Method: FTMS; HCD; 1

peptide ID 35

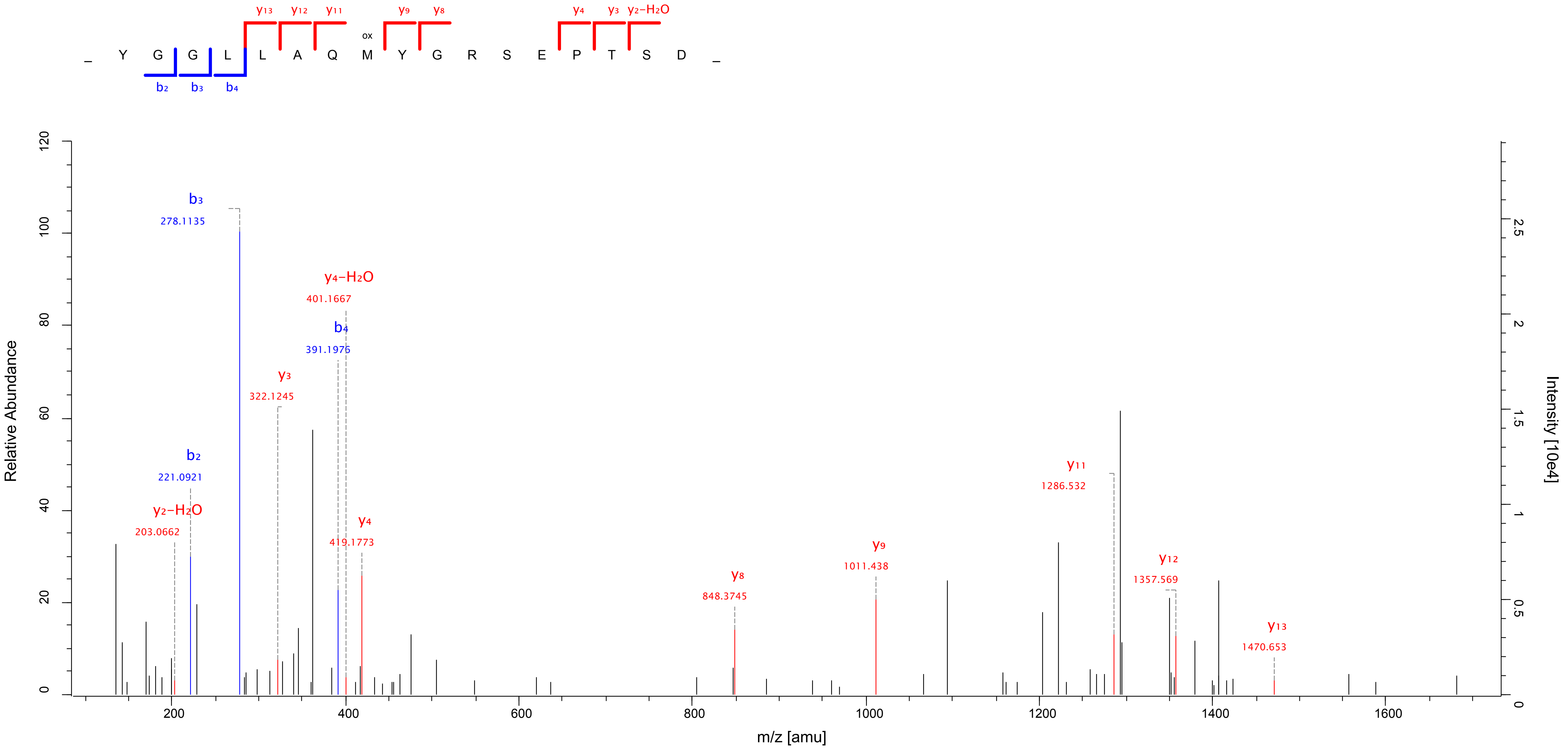

peptide ID 36

Source:	20120816_CO_0340GaJe_R02
Scannumber:	8501
Protein:	pep_secretome_15637; pep_secretome_83592
Peptide Score:	90.61
Method:	FTMS; HCD; 1

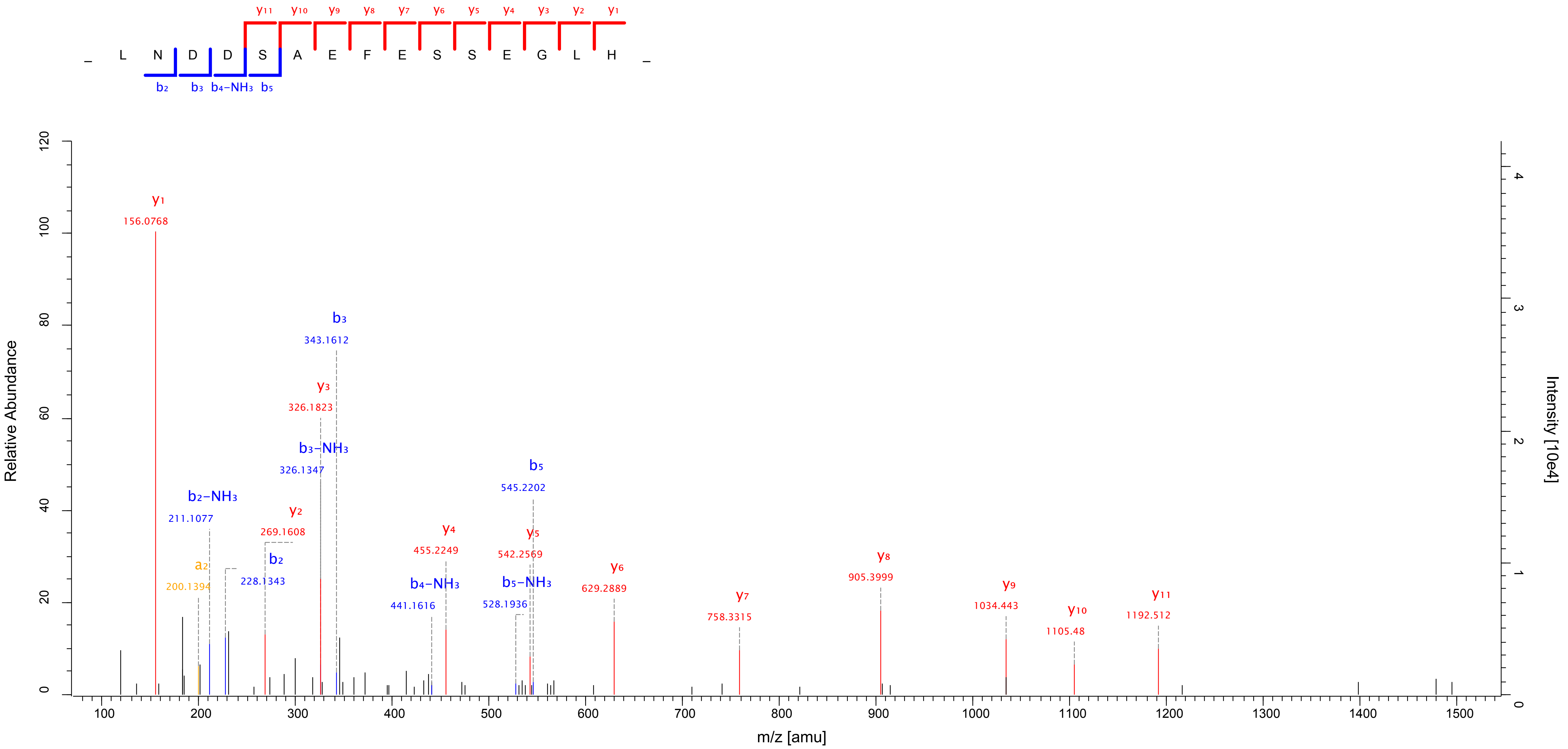

peptide ID 37

Source: 20120515_CO_0340GaJe_R01
Scannumber: 20753
Protein: pep_secretome_15649
Peptide Score: 67.86
Method: FTMS; HCD; 1

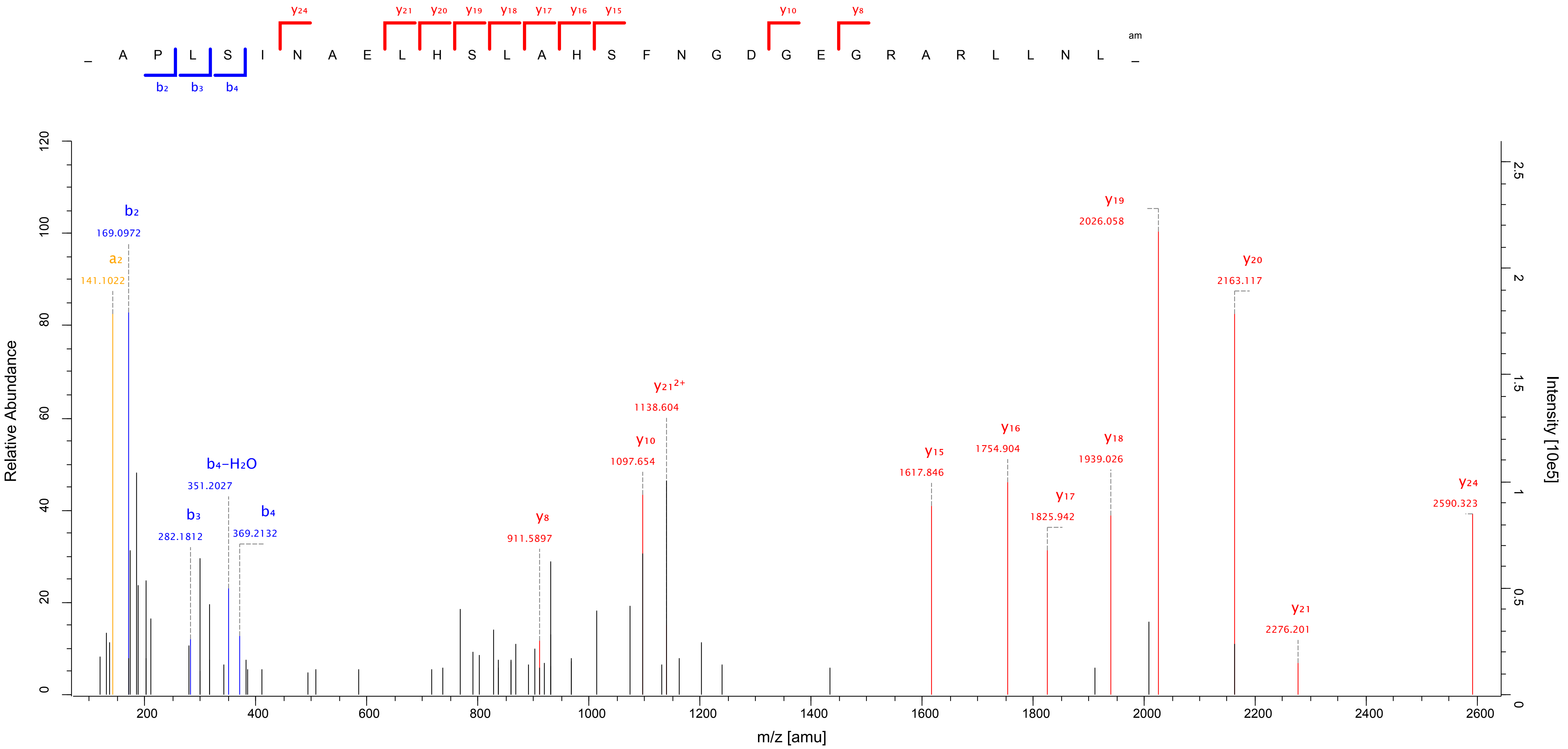

peptide ID 38

Source: 20120816_CO_0340GaJe_R02
Scannumber: 15198
Protein: pep_secretome_5102
Peptide Score: 106.58
Method: FTMS; HCD; 1

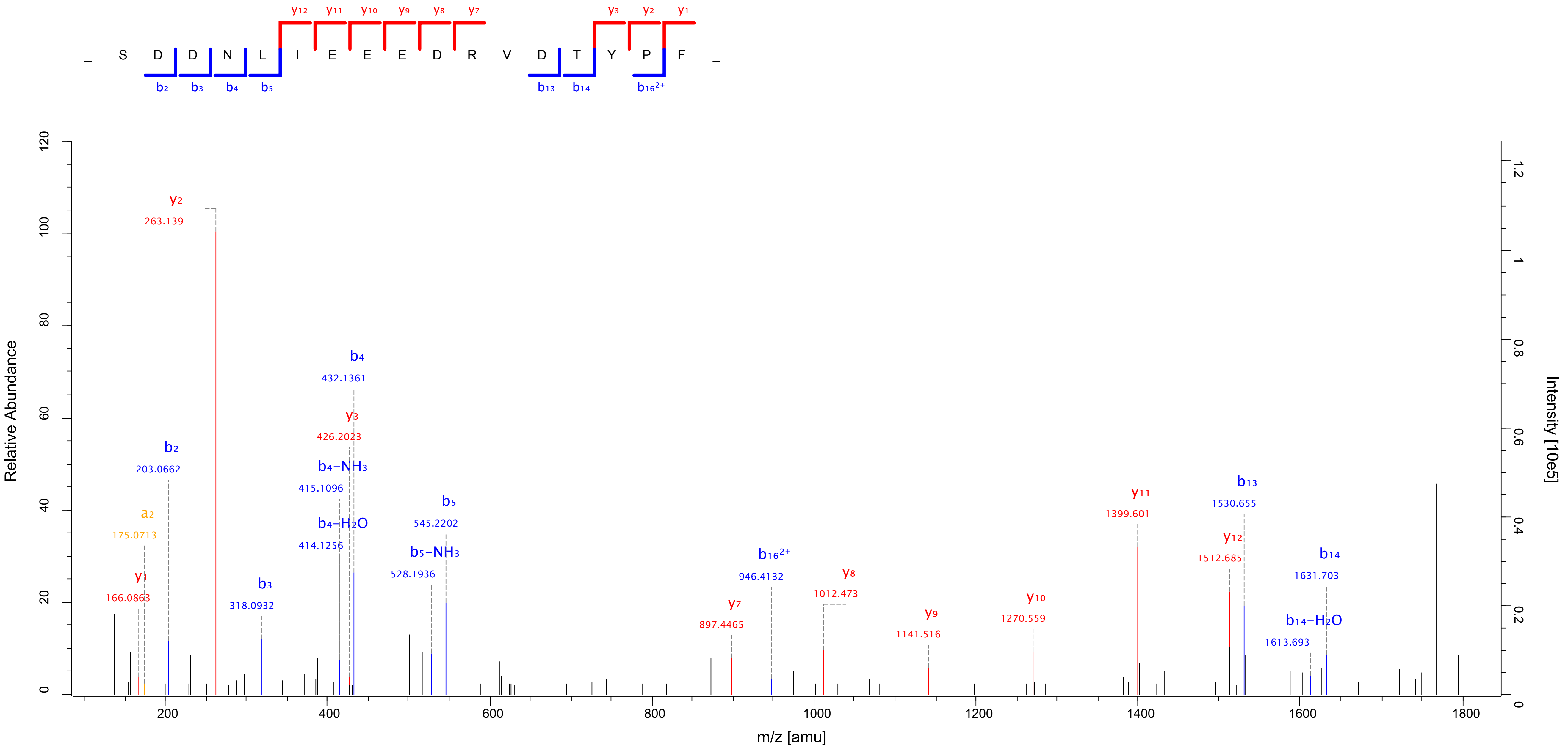

peptide ID 39

Source: 20120816_CO_0340GaJe_R02
Scannumber: 6737
Protein: SinglePep88
Peptide Score: 107.15
Method: FTMS; HCD; 1

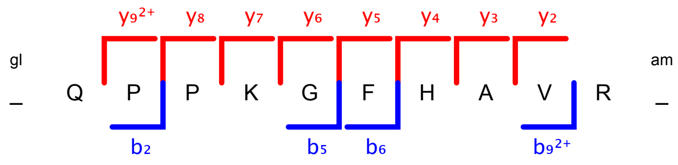

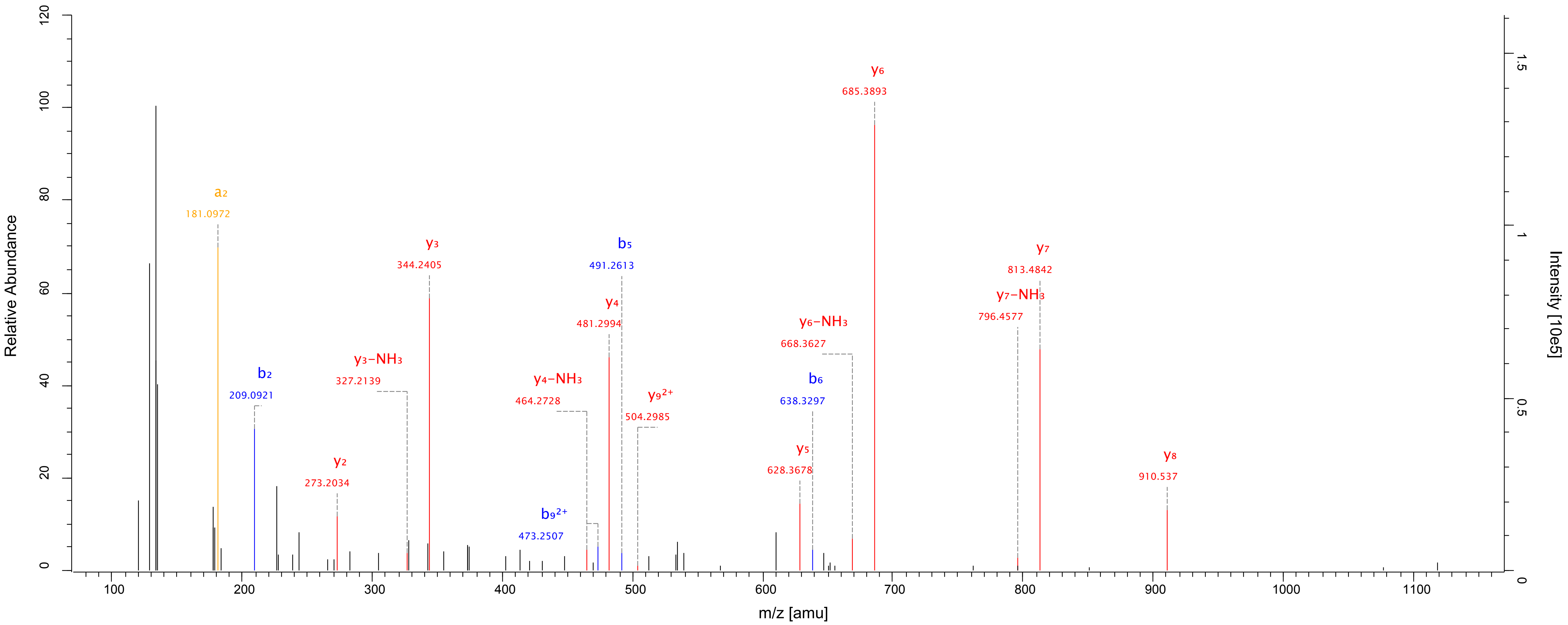

Source: 20120515_CO_0340GaJe_R01
Scannumber: 11864
Protein: SinglePep87
Peptide Score: 147.2
Method: FTMS; HCD; 1

peptide ID 40

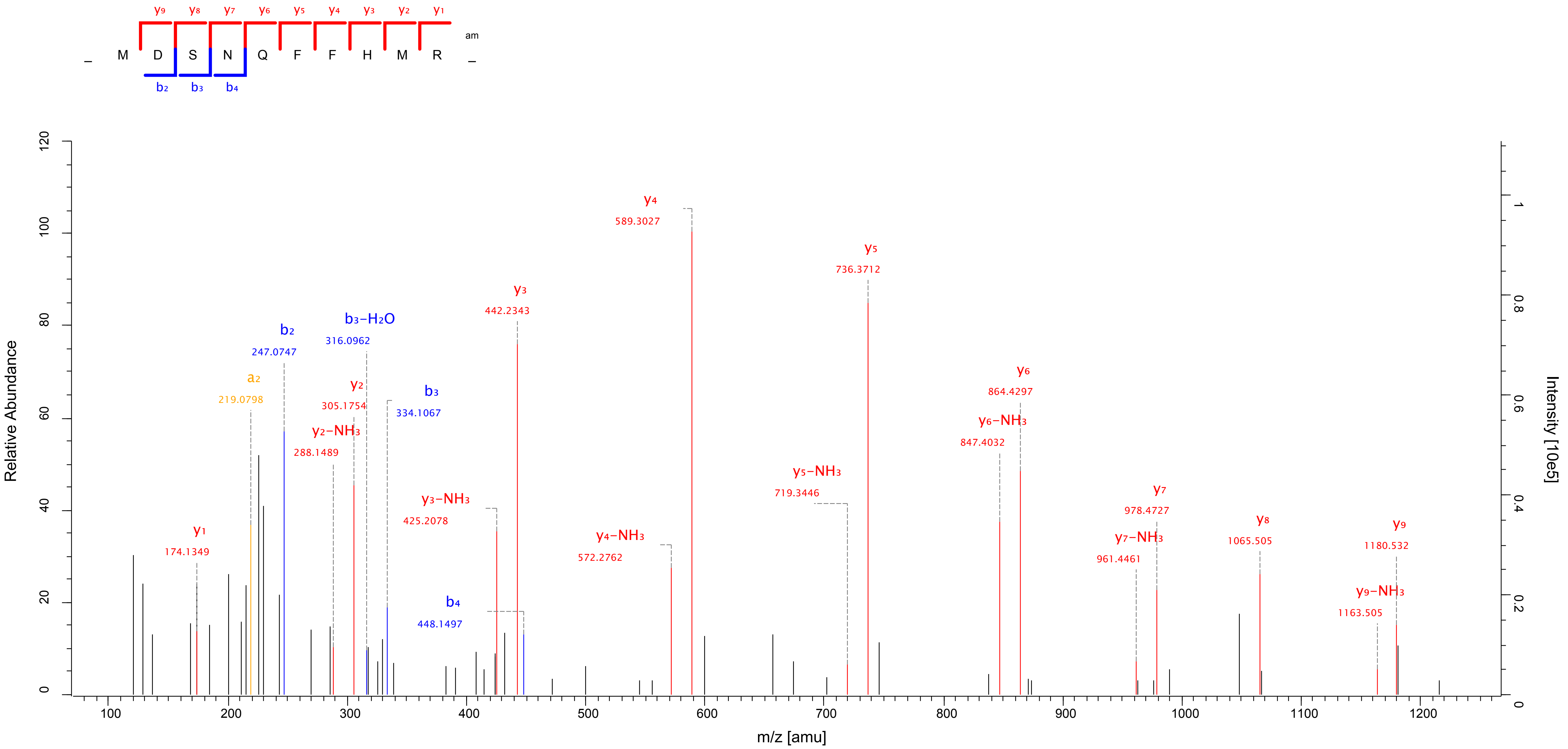

peptide ID 41

Source: 20120515_CO_0340GaJe_R01
Scannumber: 14110
Protein: pep_207; pep_secretome_10076; pep_secretome_10077; pep_secretome_63153; pep_secretome_63154
Peptide Score: 137.89
Method: FTMS; HCD; 1

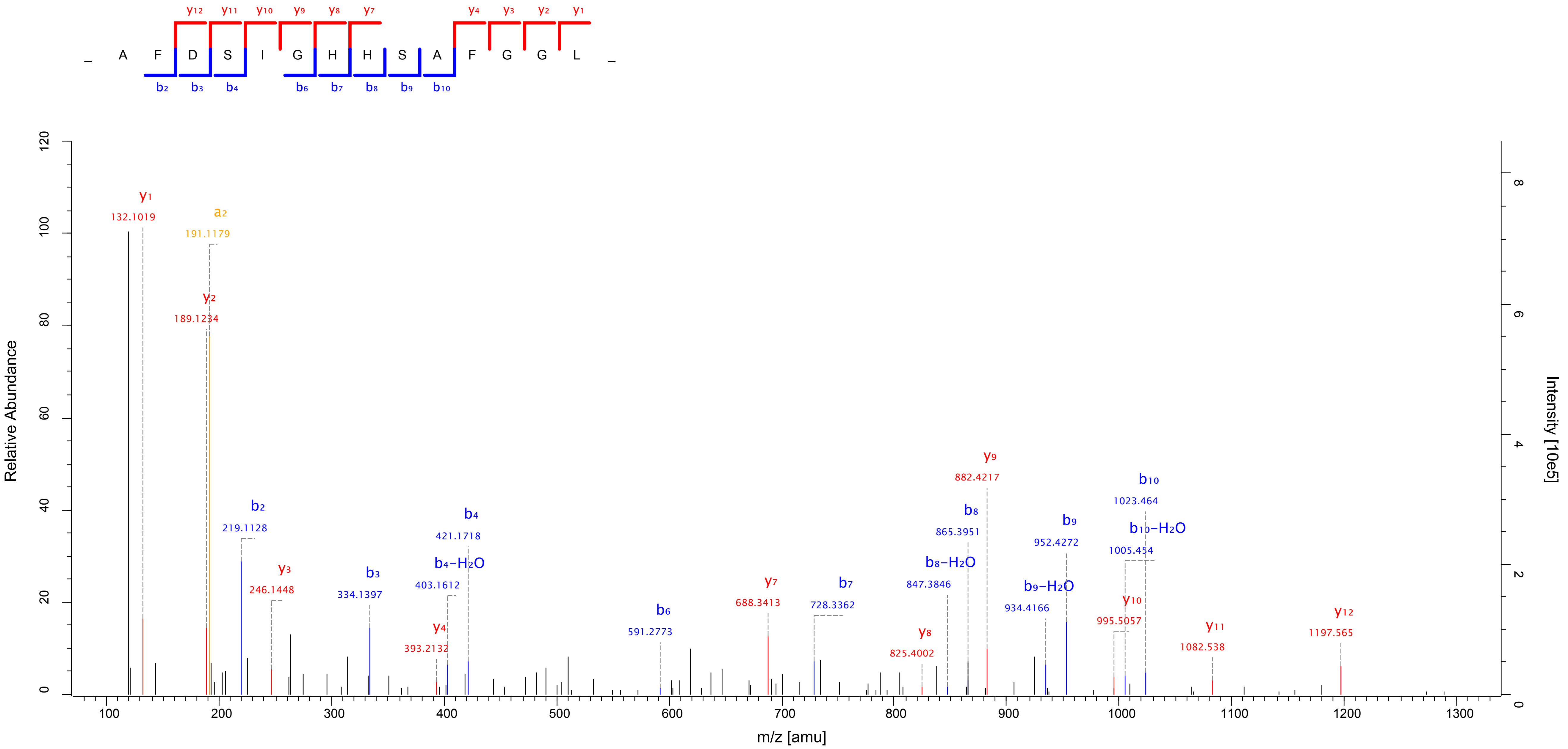

peptide ID 42

Source: 20120816_CO_0340GaJe_R02
Scannumber: 12365
Protein: orf_9630; pep_208; pep_secretome_10078; pep_secretome_10083; pep_secretome_10084; pep_secretome_10085; pep_secretome_10087; pep_secretome_10088; pep_secretome_10090; pep_secretome_10093; pep_secretome_10099; pep_secretome_101[illegible]
Peptide Score: 103.76
Method: FTMS; HCD; 1

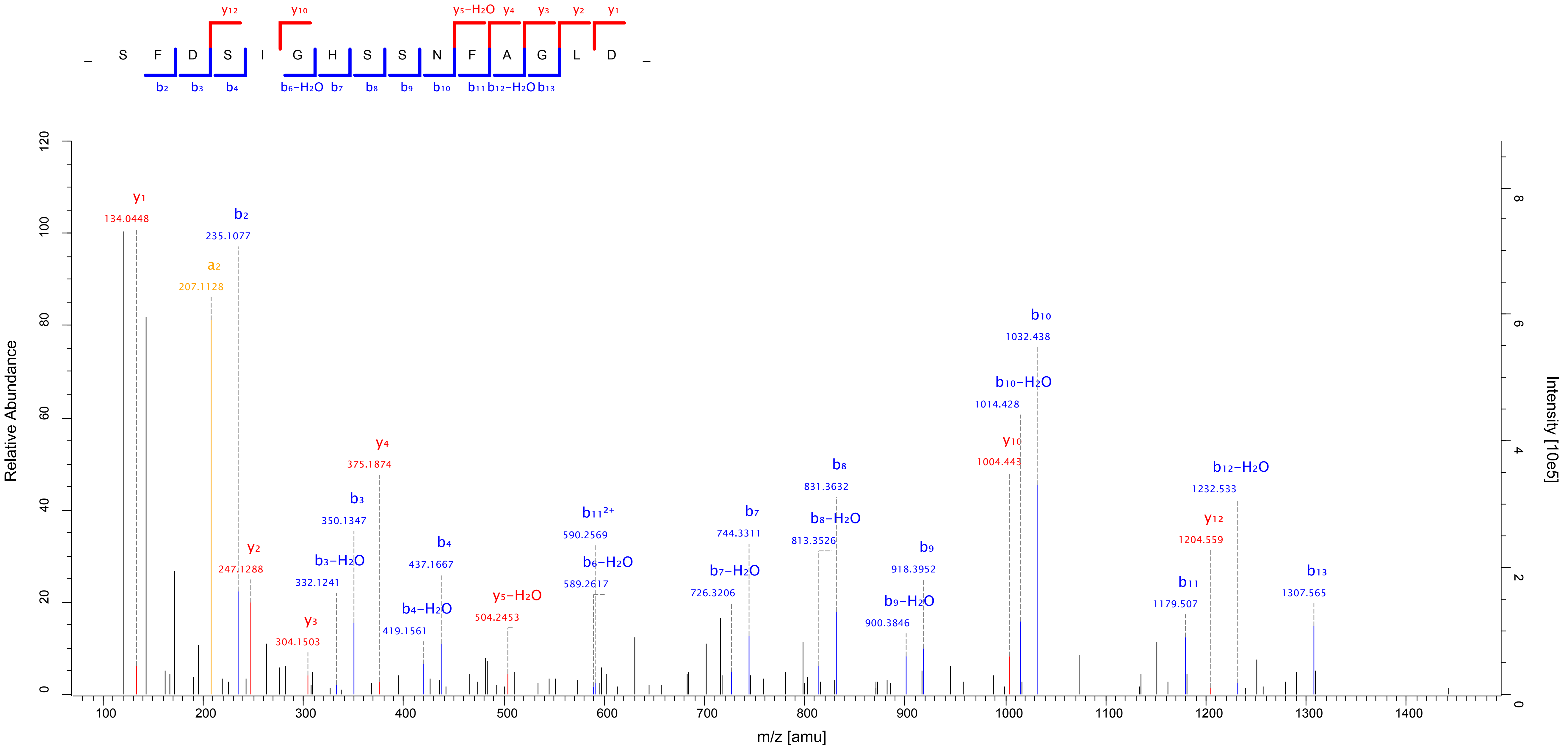

peptide ID 43

Source: 20120515_CO_0340GaJe_R01
Scannumber: 16566
Protein: orf_46468
Peptide Score: 83.14
Method: FTMS; HCD; 1

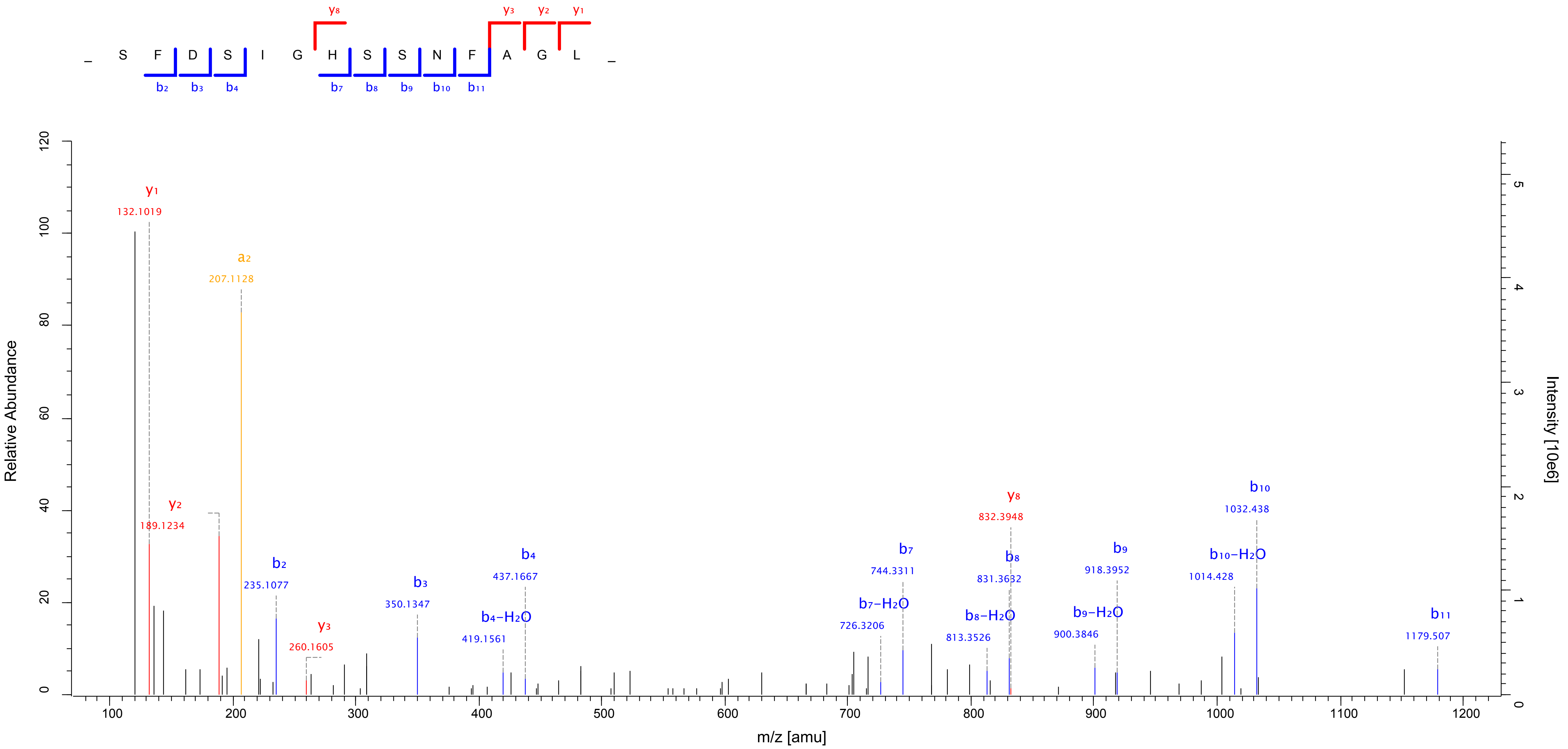

peptide ID 44

Source: 20121106_CO_0340GaJe_R02_2
Scannumber: 12920
Protein: pep_209; pep_secretome_10080; pep_secretome_10079; pep_secretome_63156; pep_secretome_63157
Peptide Score: 97.21
Method: FTMS; HCD; 1

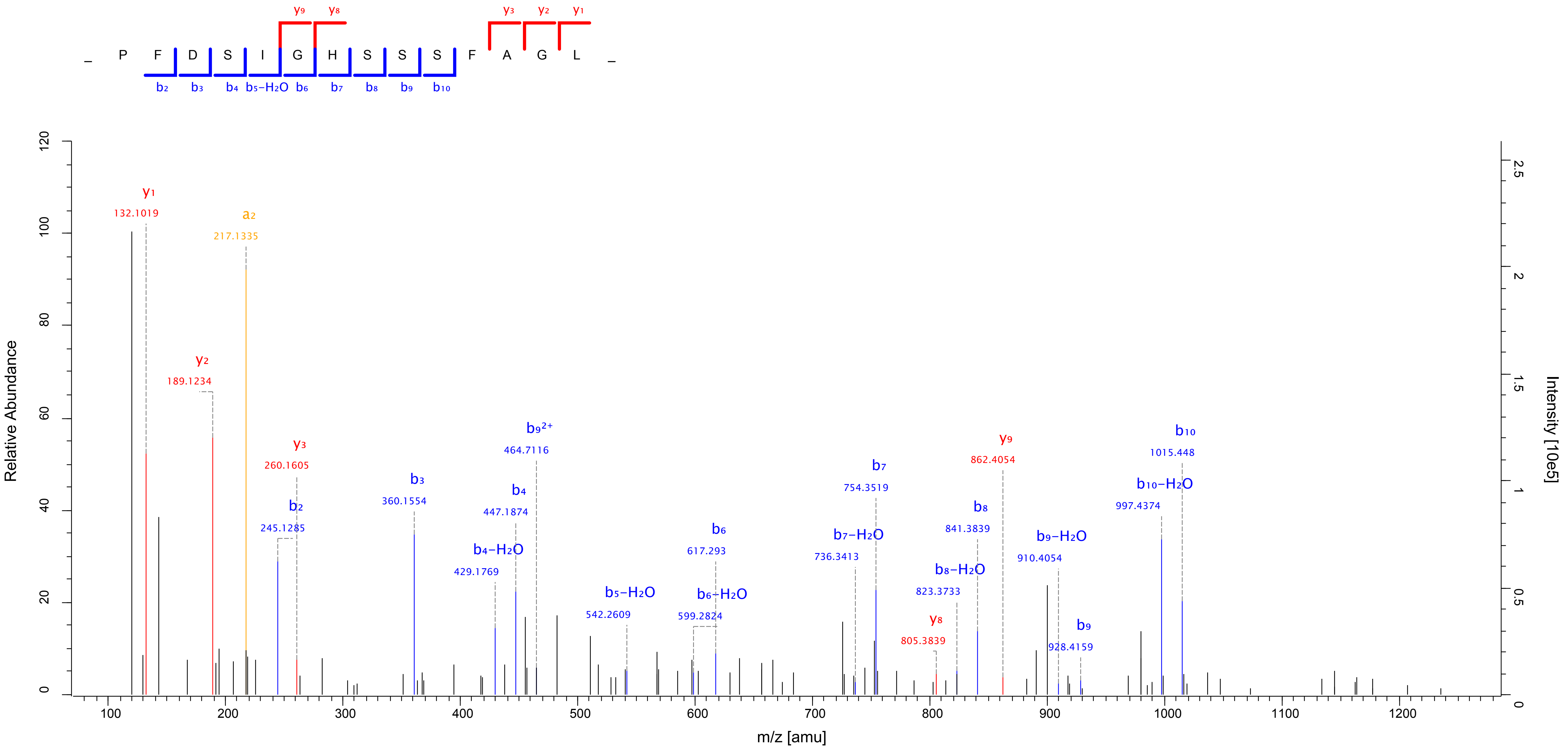

Source: 20120816_CO_0340GaJe_R02
Scannumber: 13787
Protein: pep_210; pep_secretome_10081; pep_secretome_63158
Peptide Score: 147.91
Method: FTMS; HCD; 1

peptide ID 45

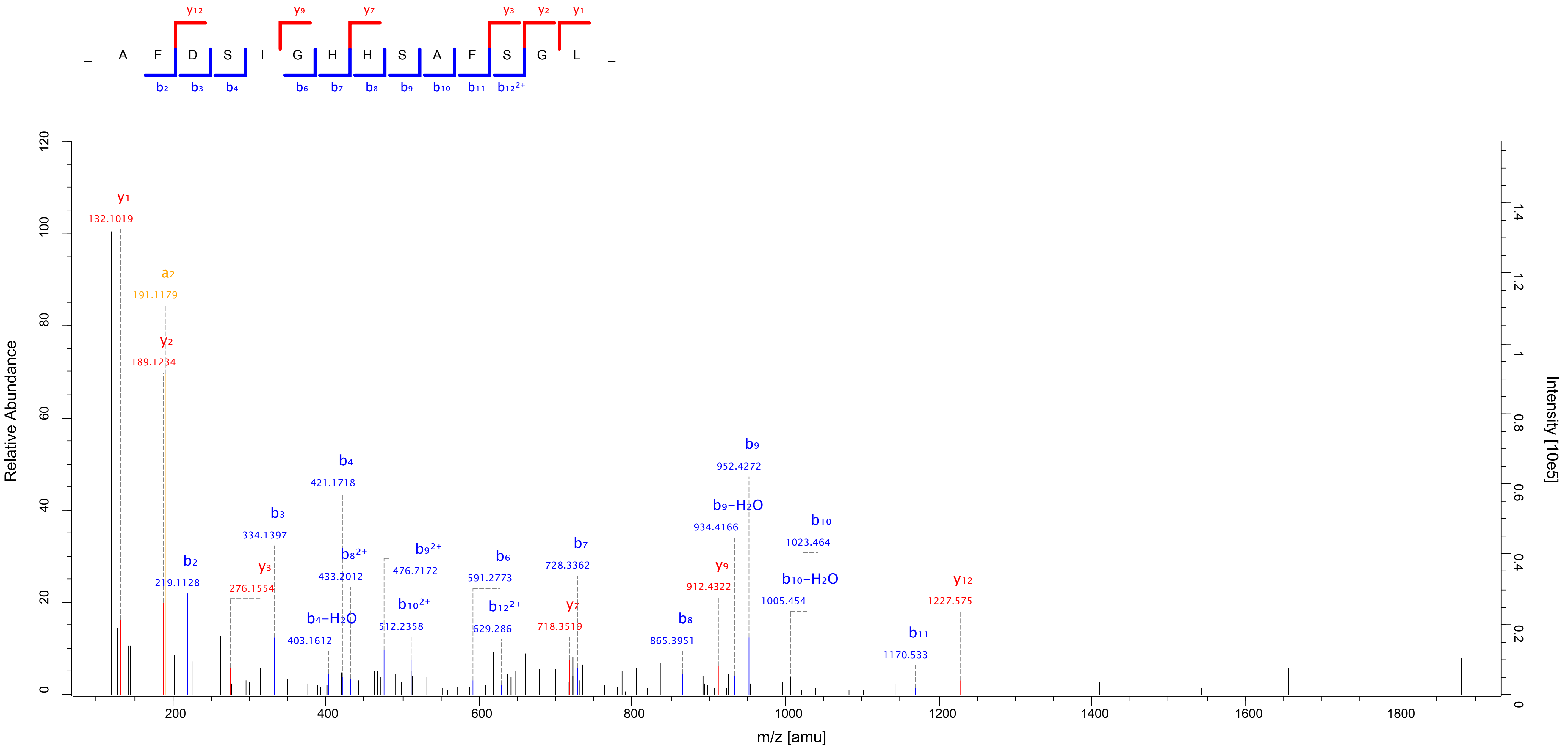

peptide ID 46

Source:	20120816_CO_0340GaJe_R02
Scannumber:	12369
Protein:	pep_212; pep_secretome_10091; pep_secretome_10094; pep_secretome_10095; pep_secretome_10096; pep_secretome_10097; pep_secretome_10098; pep_secretome_63168; pep_secretome_63171; pep_secretome_63172; pep_secretome_63173; pep_se
Peptide Score:	104.7
Method:	FTMS; HCD; 1

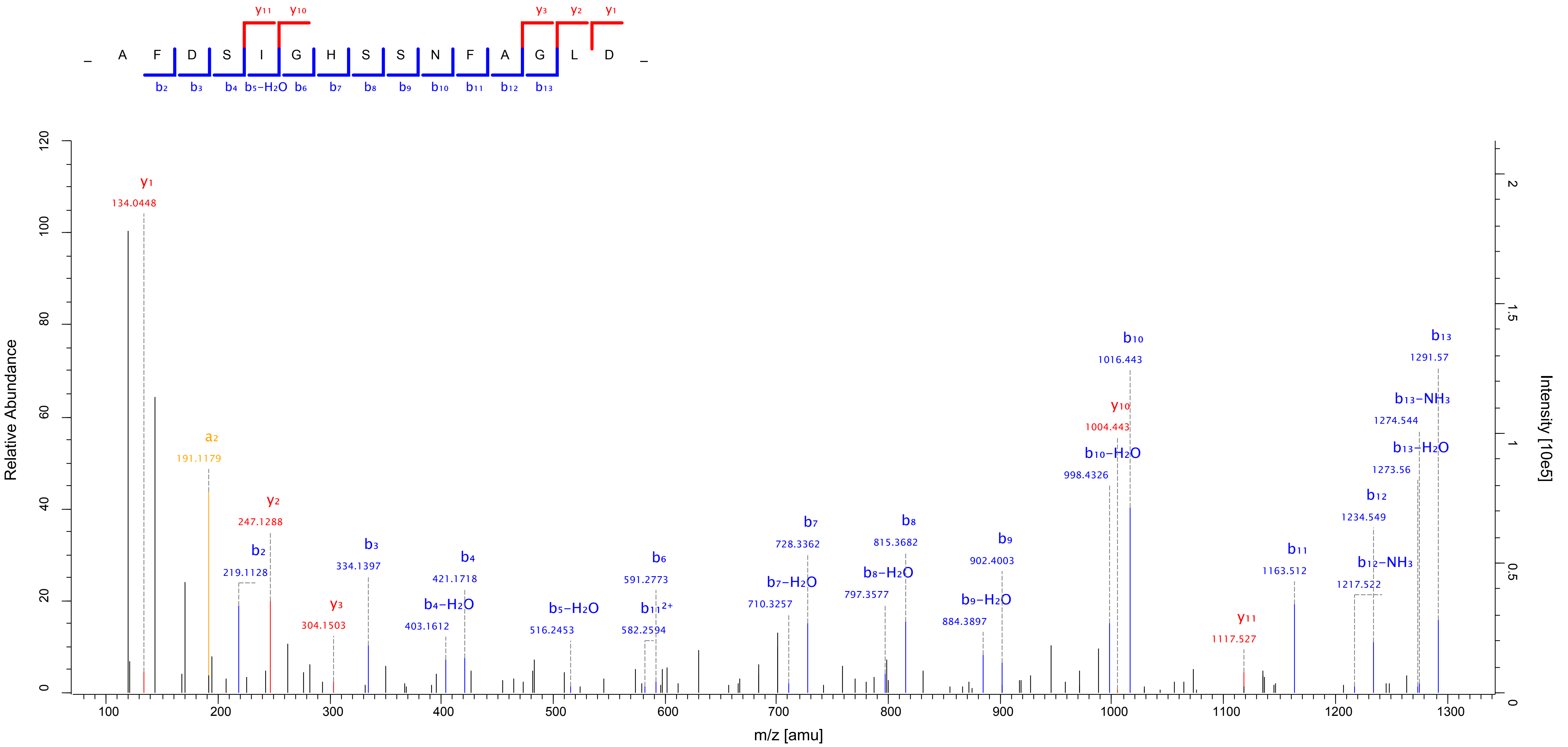

peptide ID 47

Source:	20120816_CO_0340GaJe_R02
Scannumber:	13077
Protein:	pep_214
Peptide Score:	100.19
Method:	FTMS; HCD; 1

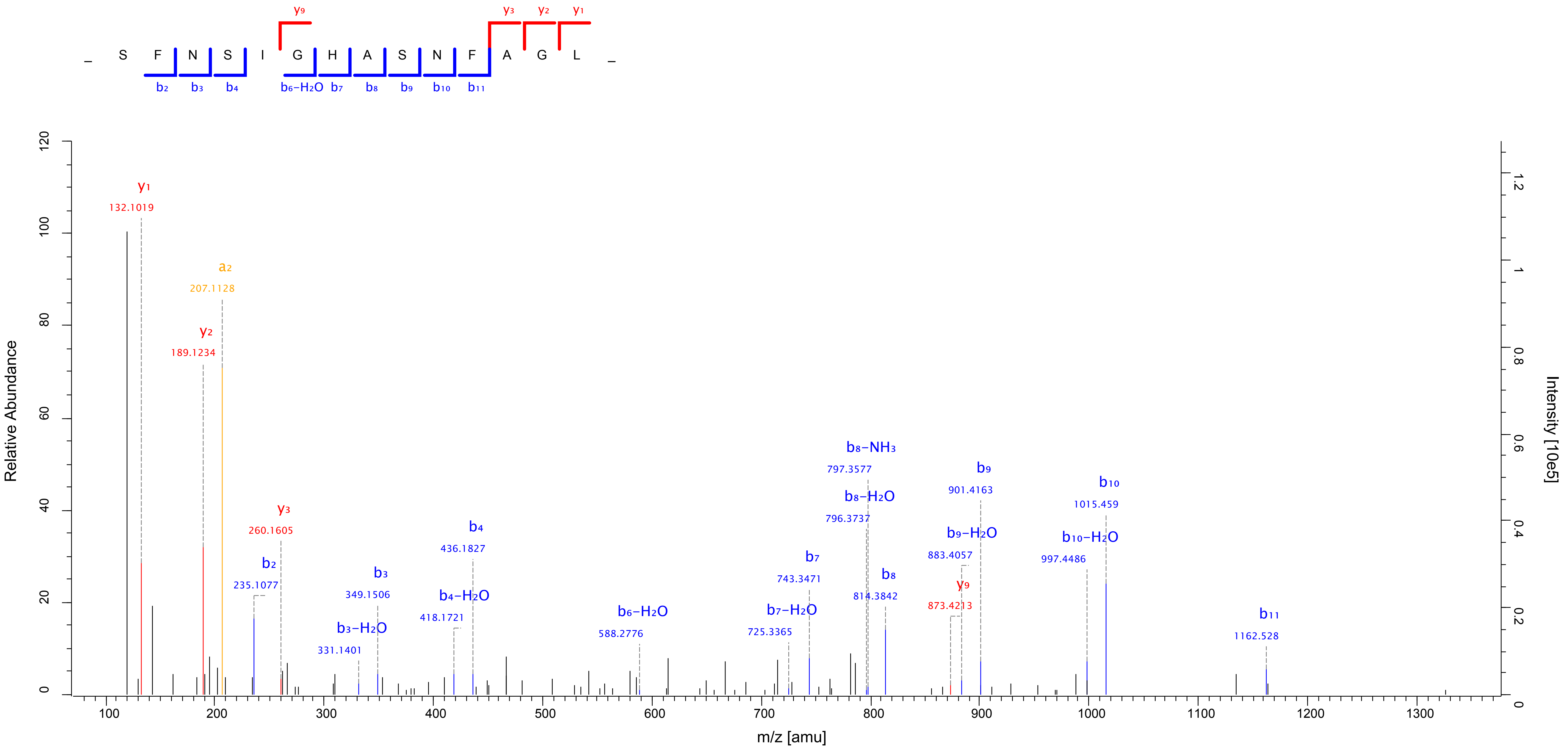

peptide ID 48

Source: 20120816_CO_0340GaJe_R02
Scannumber: 7364
Protein: pep_62; pep_secretome_113
Peptide Score: 64.94
Method: FTMS; HCD; 1

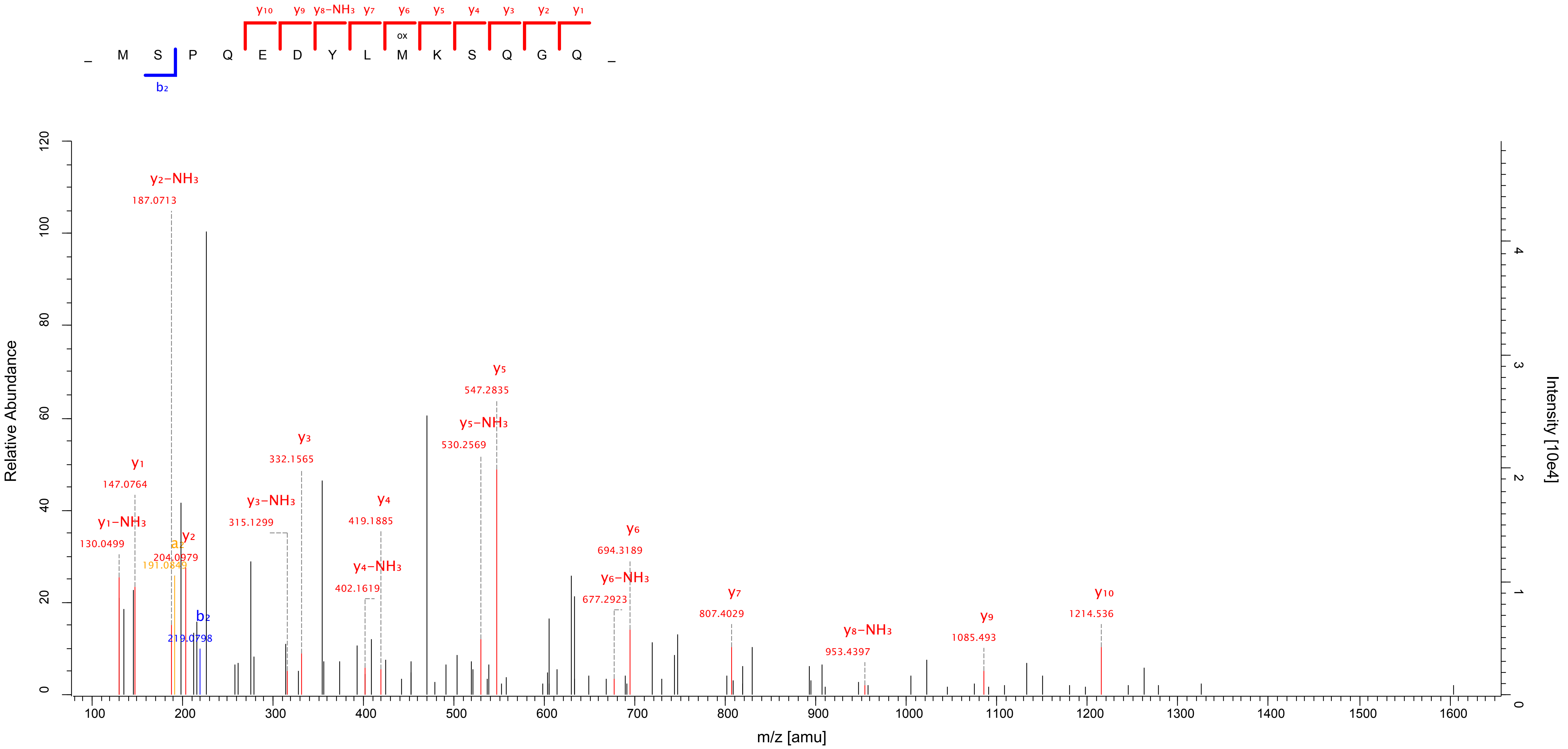

peptide ID 49

Source:	20121106_CO_0340GaJe_R02_2
Scannumber:	6387
Protein:	pep_62; pep_secretome_113
Peptide Score:	93.77
Method:	FTMS; HCD; 1

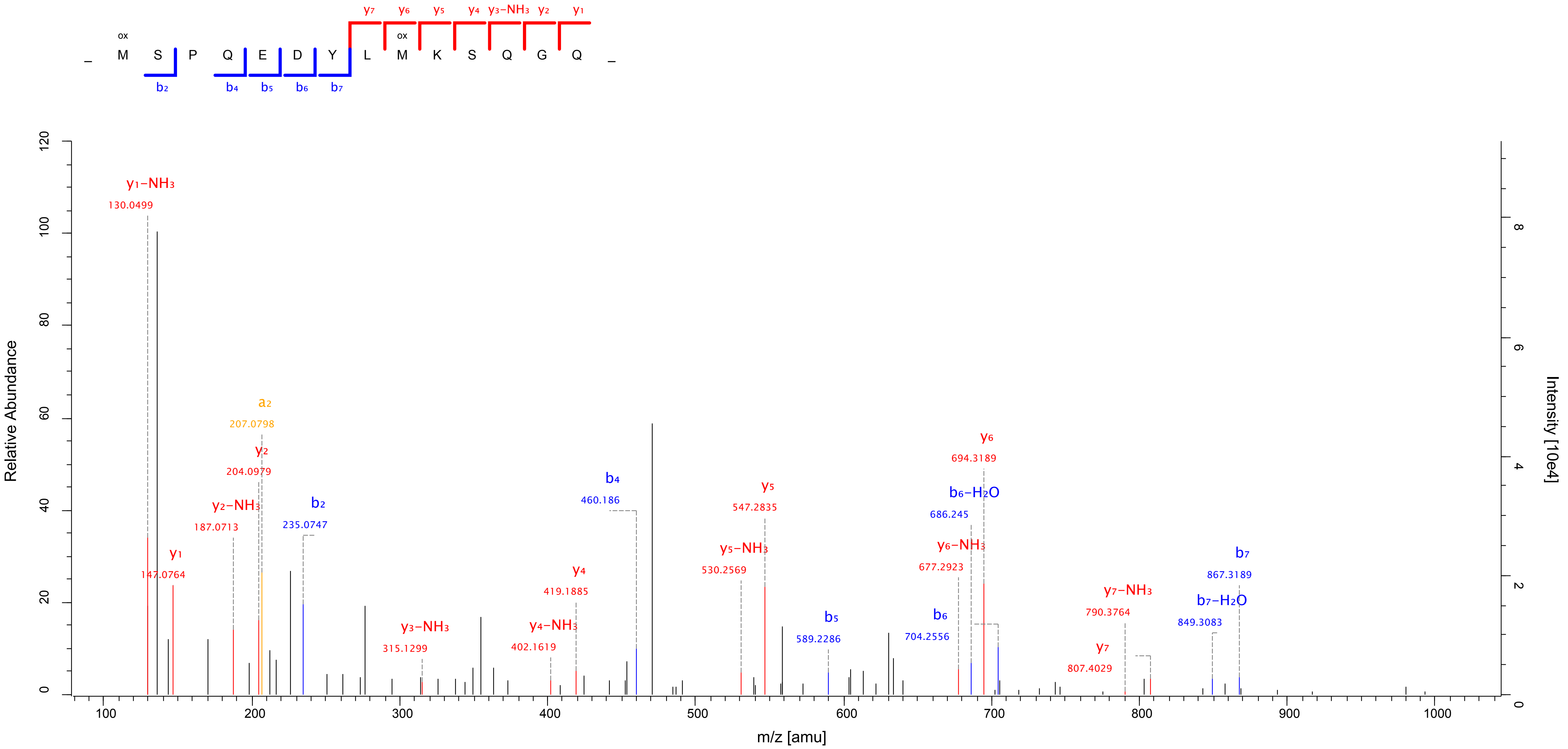

Source: 20120515_CO_0340GaJe_R01
Scannumber: 17484
Protein: pep_63; pep_secretome_115; pep_secretome_116; pep_secretome_117; pep_secretome_118; pep_secretome_120; pep_secretome_114; pep_secretome_22758; pep_secretome_22759; pep_secretome_22760; pep_secretome_22761; pep_secretome_22762;
Peptide Score: 178.84
Method: FTMS; HCD; 1

peptide ID 50

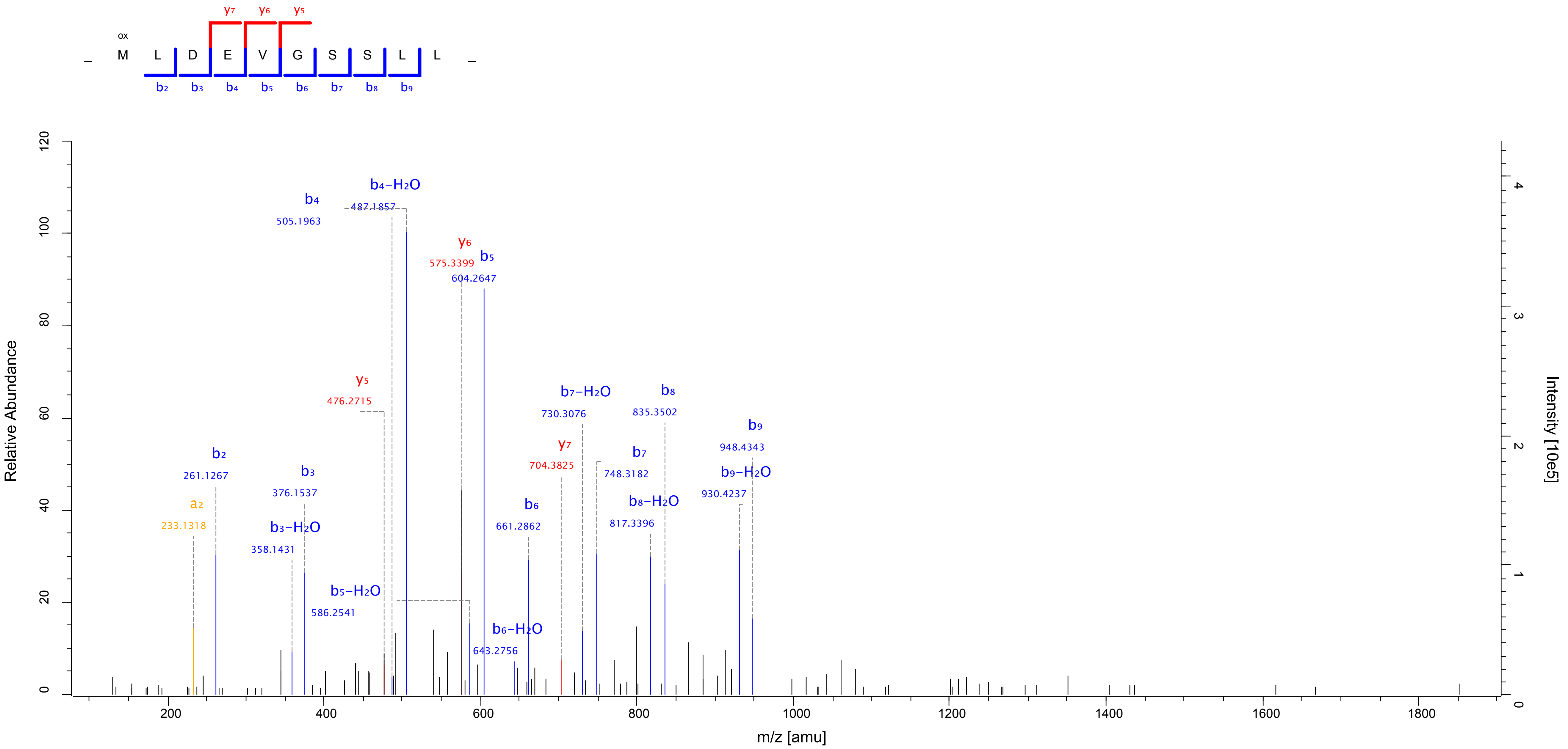

peptide ID 51

Source:	20120816_CO_0340GaJe_R02
Scannumber:	13703
Protein:	pep_64; pep_secretome_121
Peptide Score:	134.15
Method:	FTMS; HCD; 1

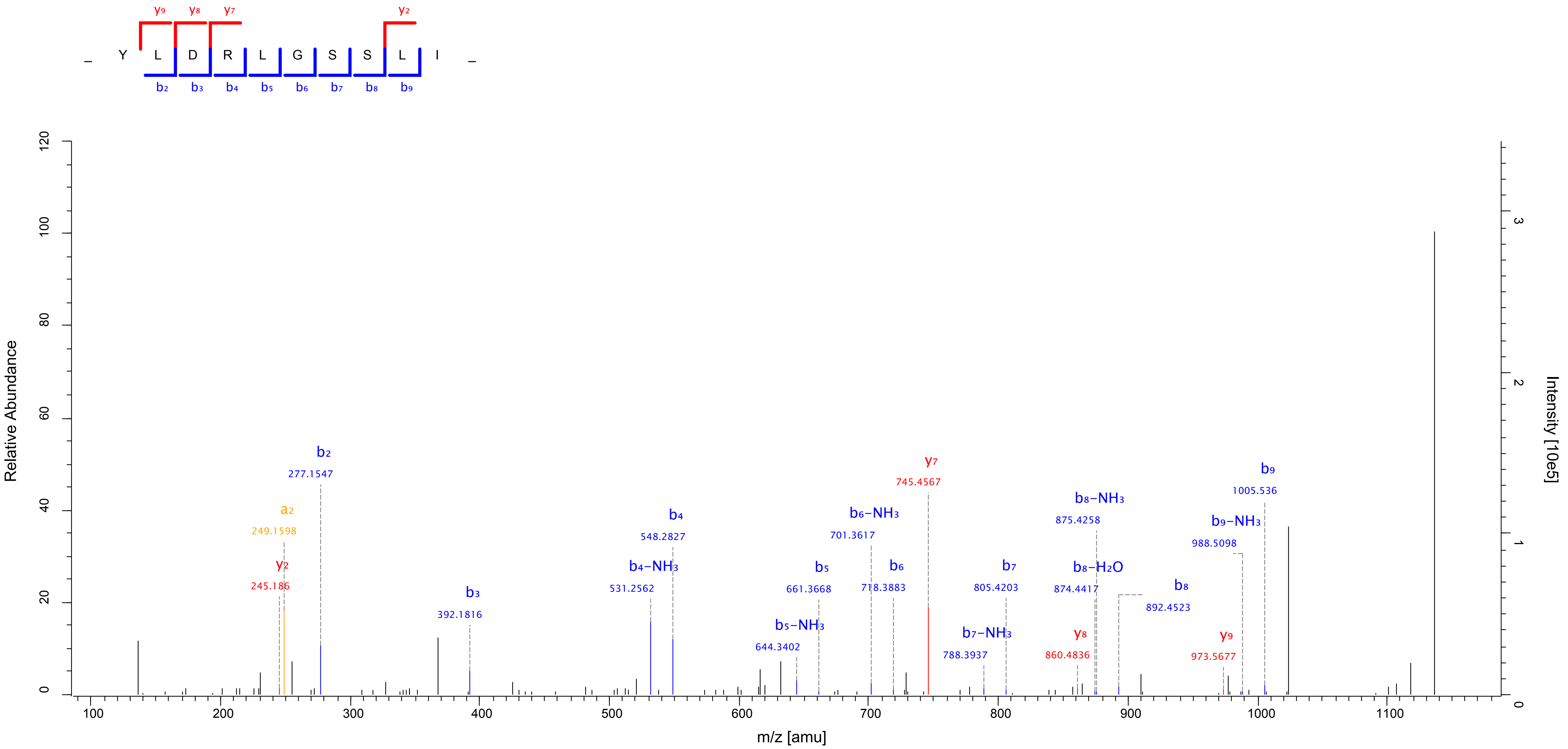

Source: 20120816_CO_0340GaJe_R02
Scannumber: 8454
Protein: orf_3472; orf_7284
Peptide Score: 95.77
Method: FTMS; HCD; 1

peptide ID 52

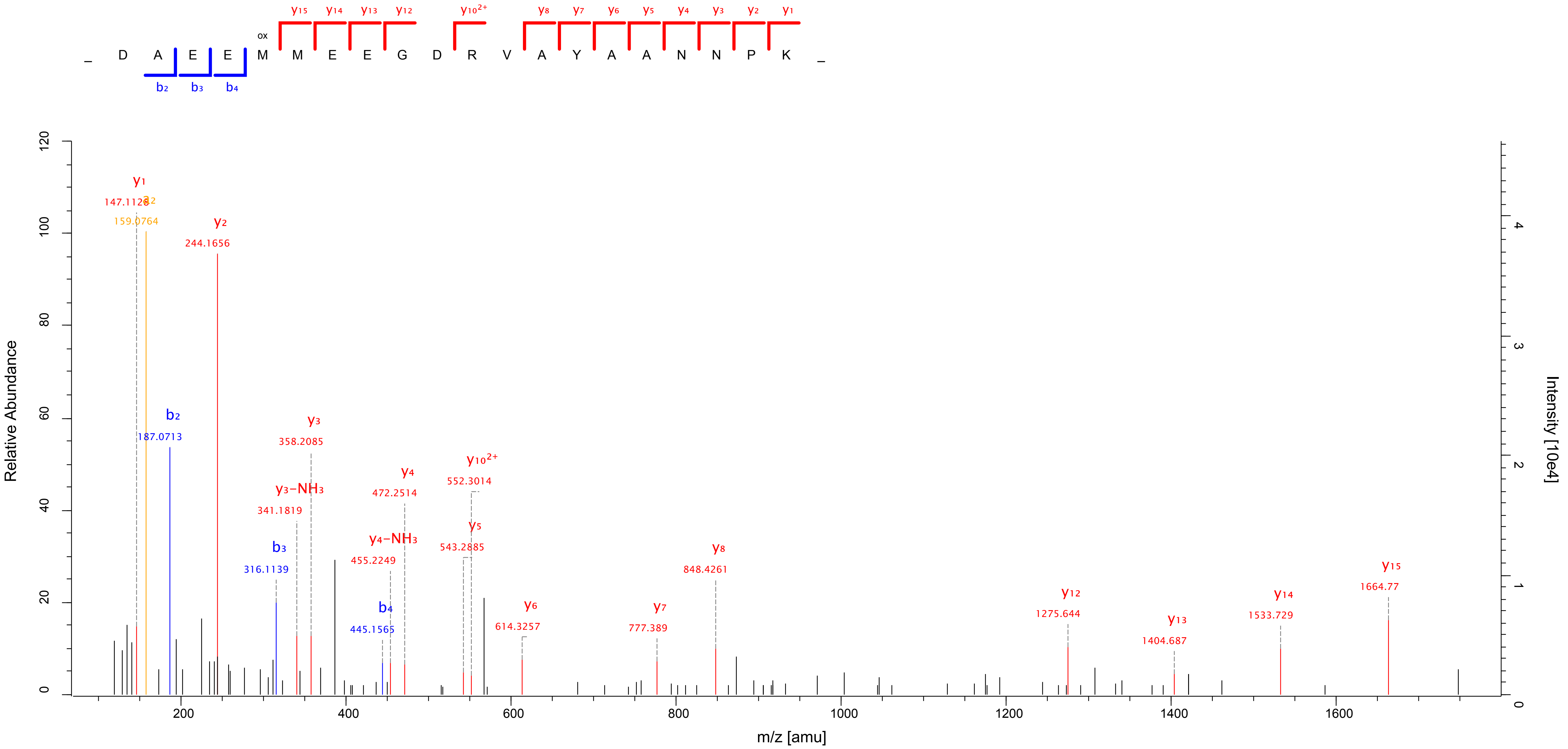

Source: 20120816_CO_0340GaJe_R02
Scannumber: 6595
Protein: orf_3472; orf_7284
Peptide Score: 74.12
Method: FTMS; HCD; 1

peptide ID 53

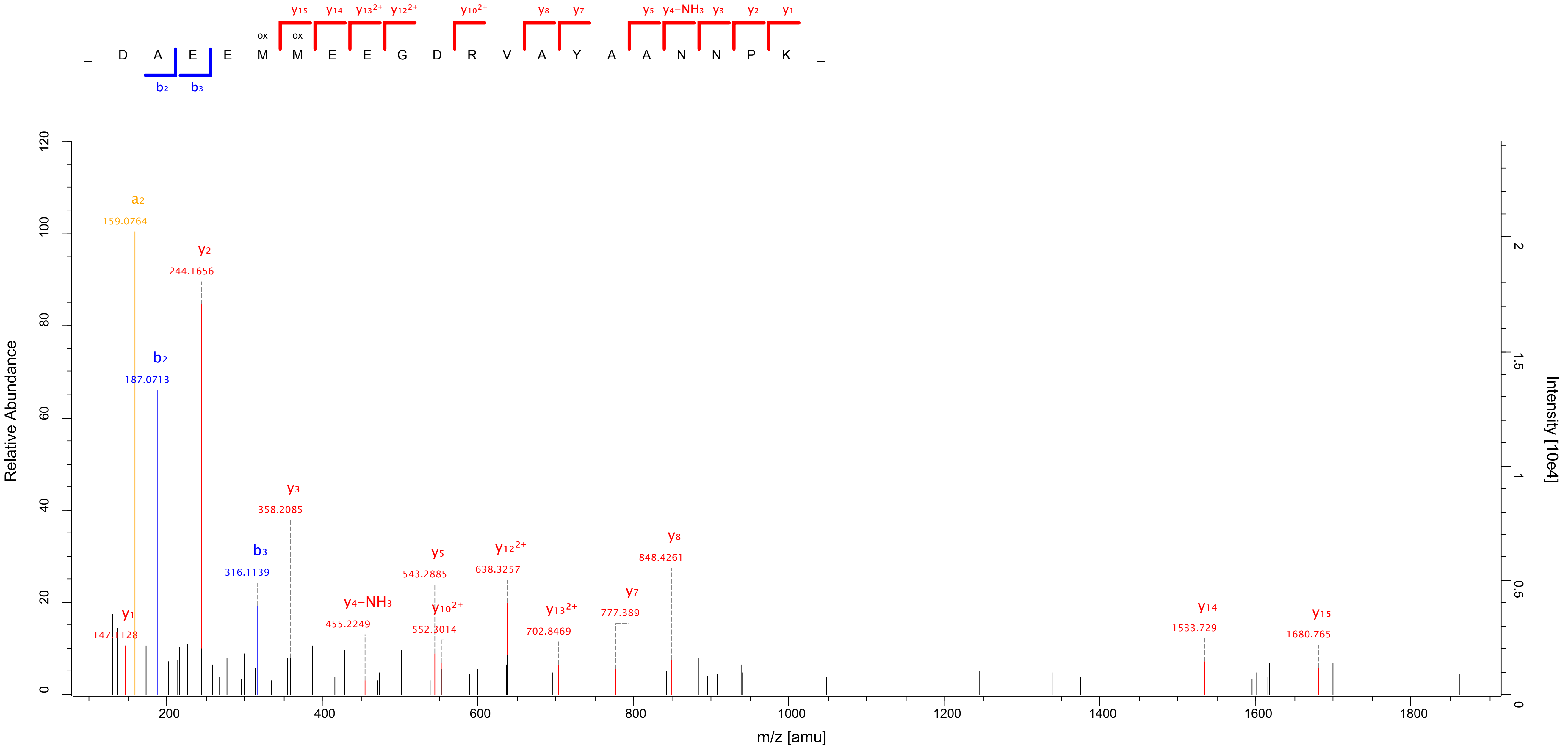

peptide ID 54

Source: 20120816_CO_0340GaJe_R02
Scannumber: 7087
Protein: pep_secretome_57816; pep_secretome_9016
Peptide Score: 64.8
Method: FTMS; HCD; 1

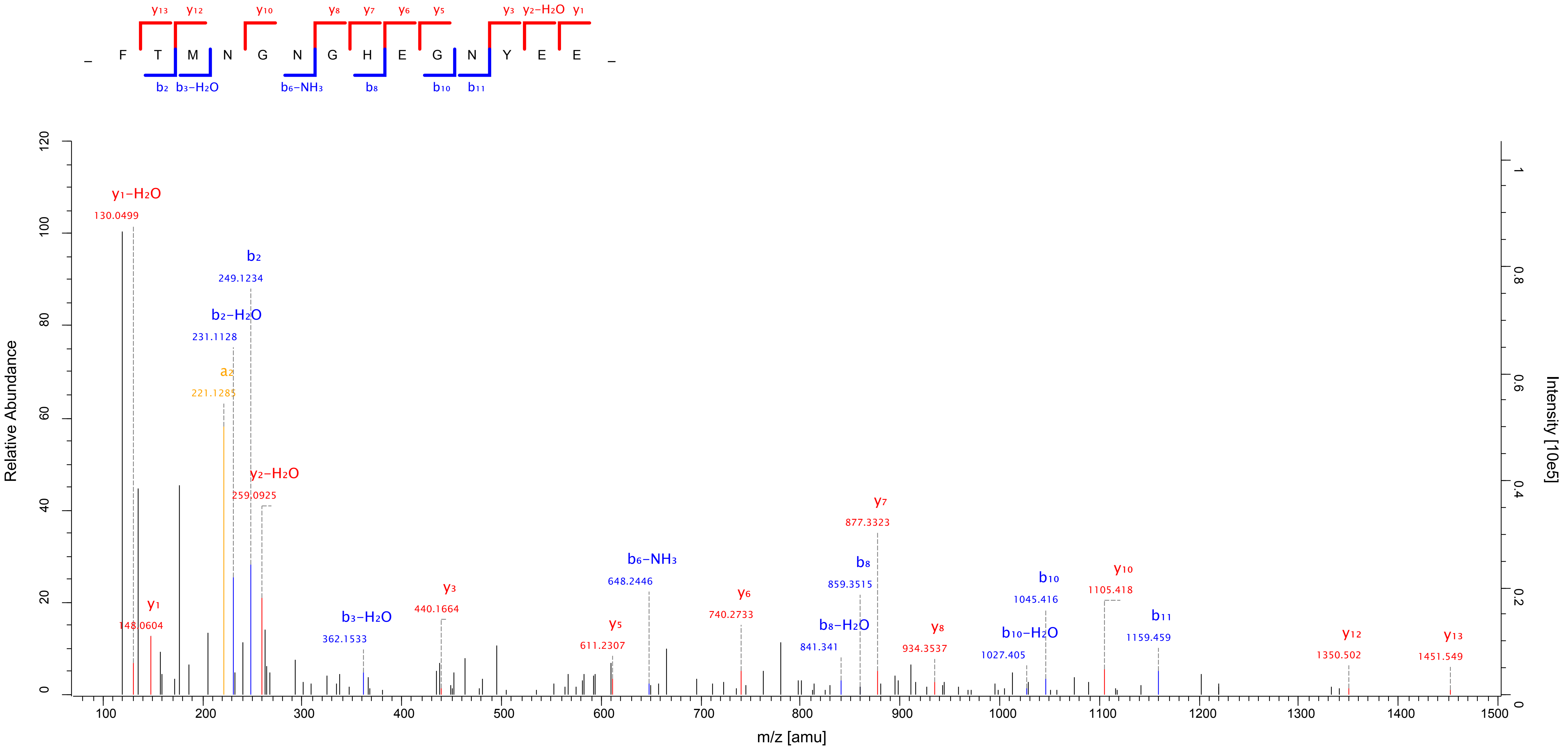

peptide ID 55

Source:	20120816_CO_0340GaJe_R02
Scannumber:	13663
Protein:	SinglePep86
Peptide Score:	134.44
Method:	FTMS; HCD; 1

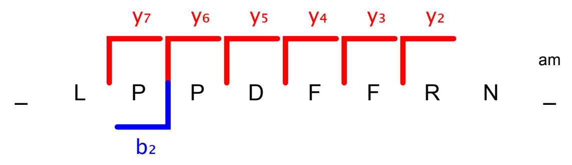

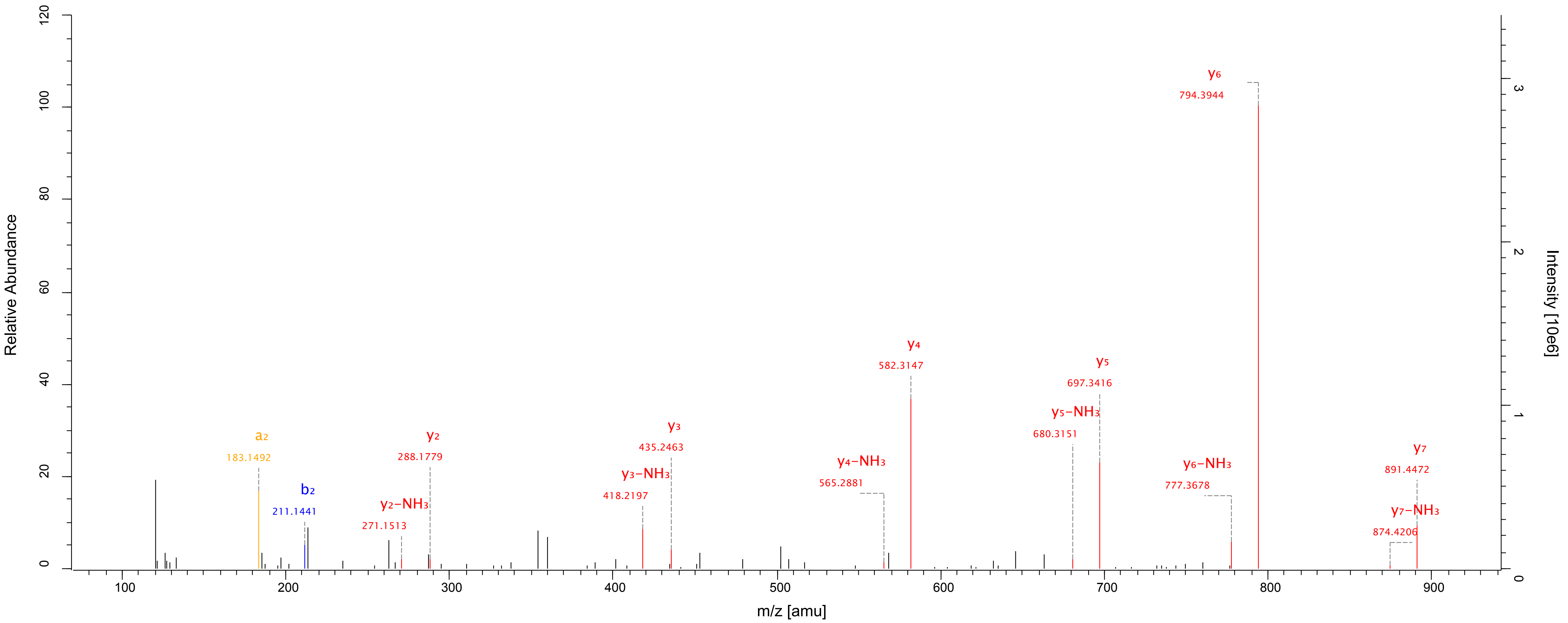

peptide ID 56

Source: 20120816_CO_0340GaJe_R02
Scannumber: 13679
Protein: SinglePep79
Peptide Score: 70.41
Method: FTMS; HCD; 1

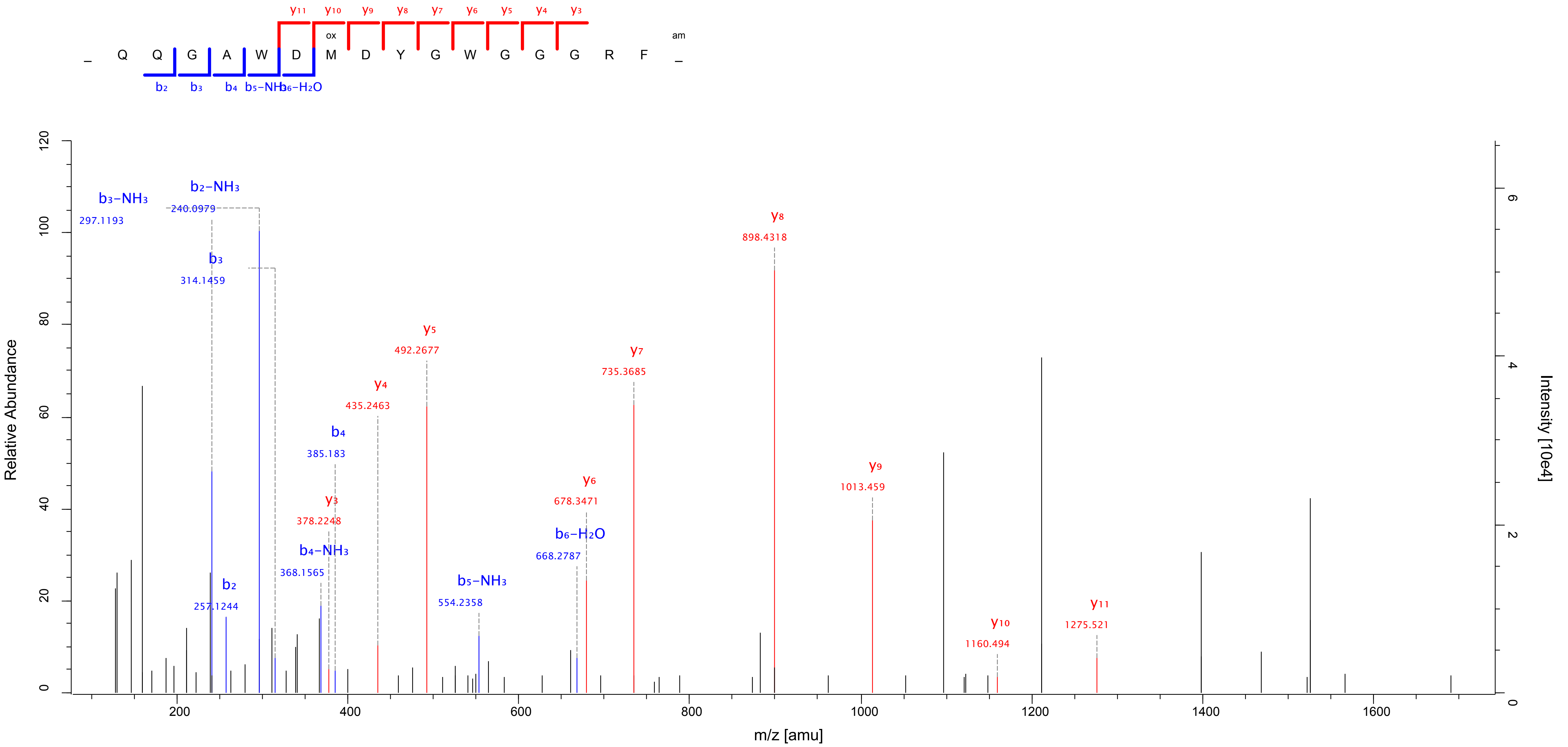

Source: 20121106_CO_0340GaJe_R02_2
Scannumber: 14735
Protein: SinglePep79
Peptide Score: 70.62
Method: FTMS; HCD; 1

peptide ID 57

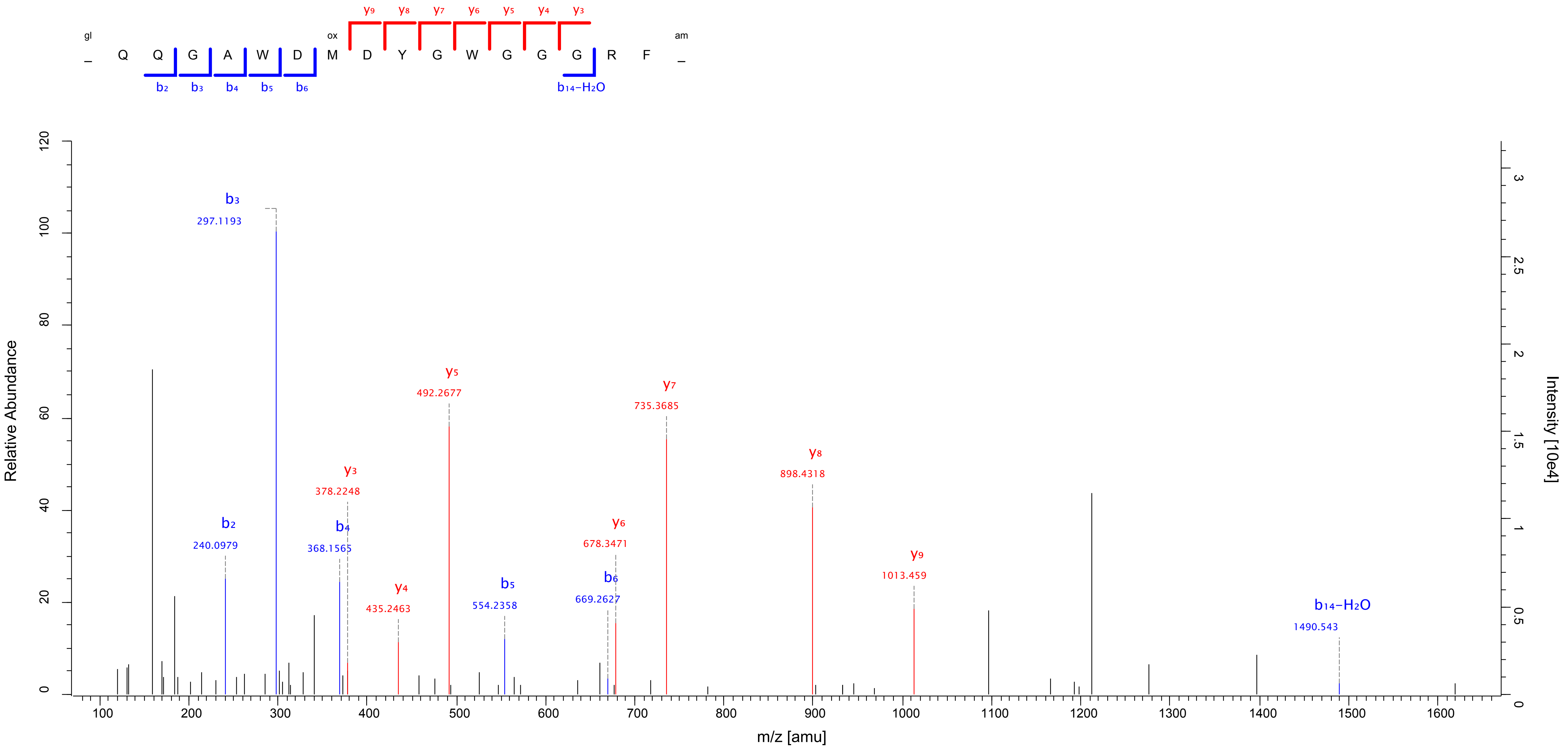

peptide ID 58

Source:	20120816_CO_0340GaJe_R02
Scannumber:	12435
Protein:	SinglePep78
Peptide Score:	86.01
Method:	FTMS; HCD; 1

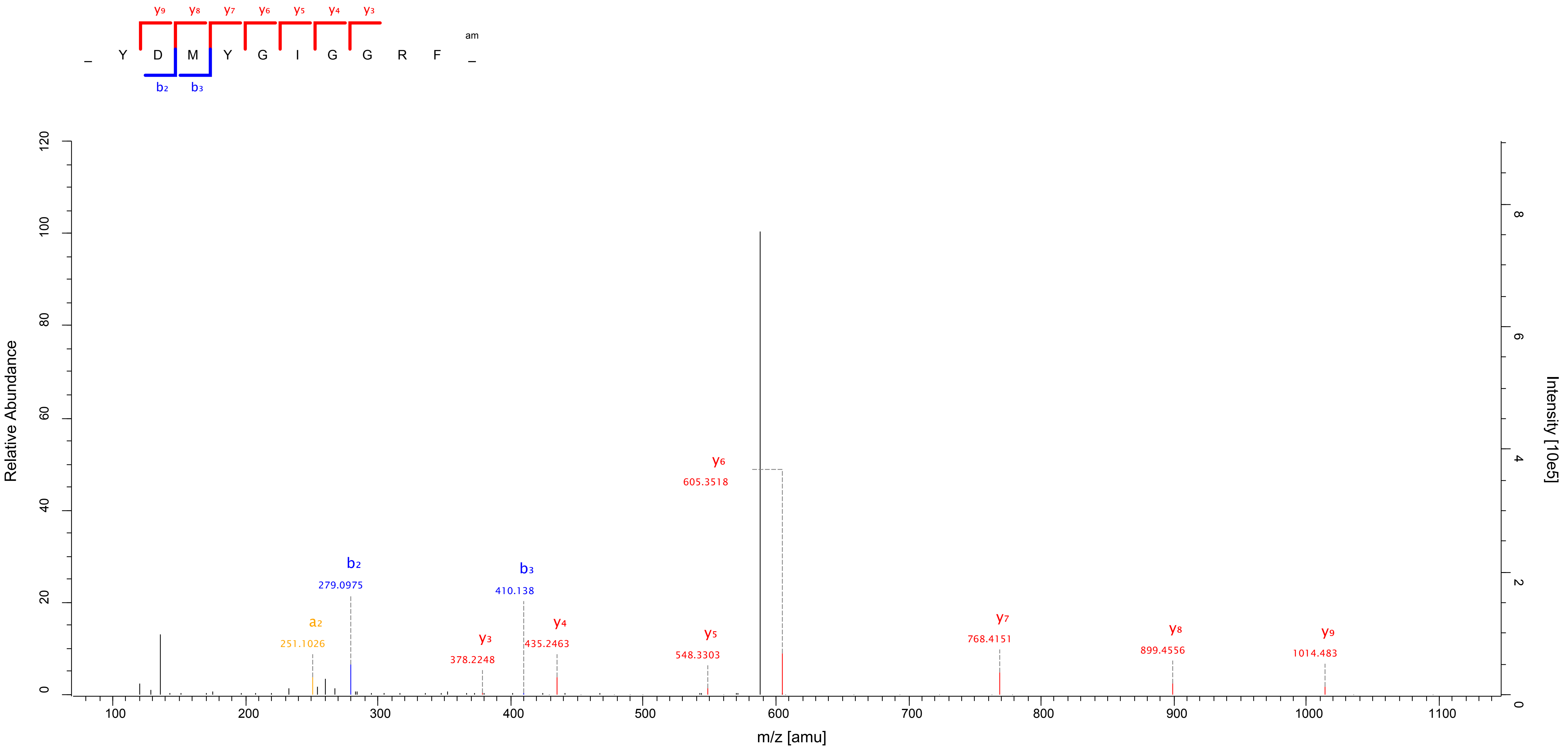

Source: 20120515_CO_0340GaJe_R01
Scannumber: 16351
Protein: SinglePep78
Peptide Score: 72.55
Method: FTMS; HCD; 1

peptide ID 59

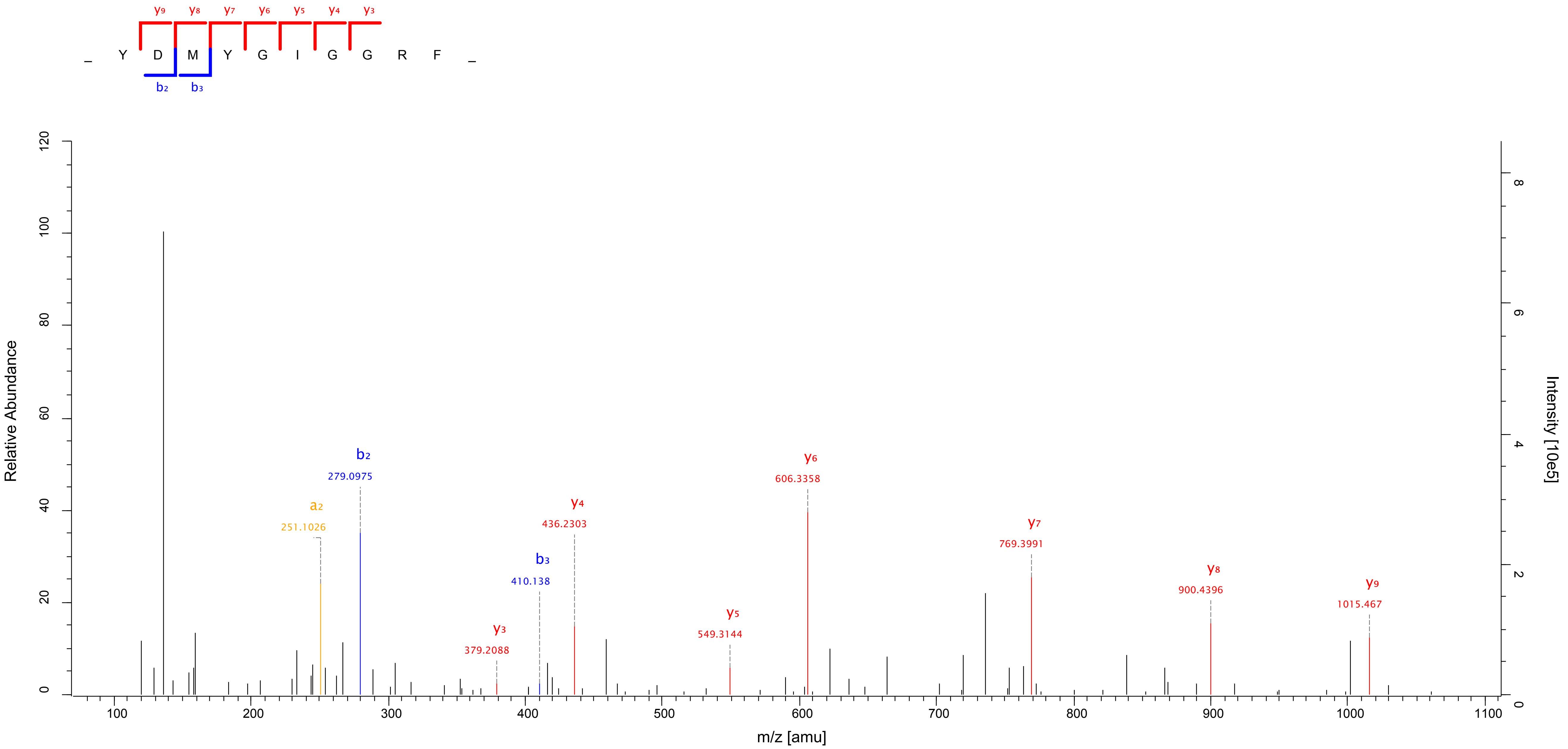

peptide ID 60

Source: 20120816_CO_0340GaJe_R02
Scannumber: 10191
Protein: SinglePep78
Peptide Score: 83.26
Method: FTMS; HCD; 1

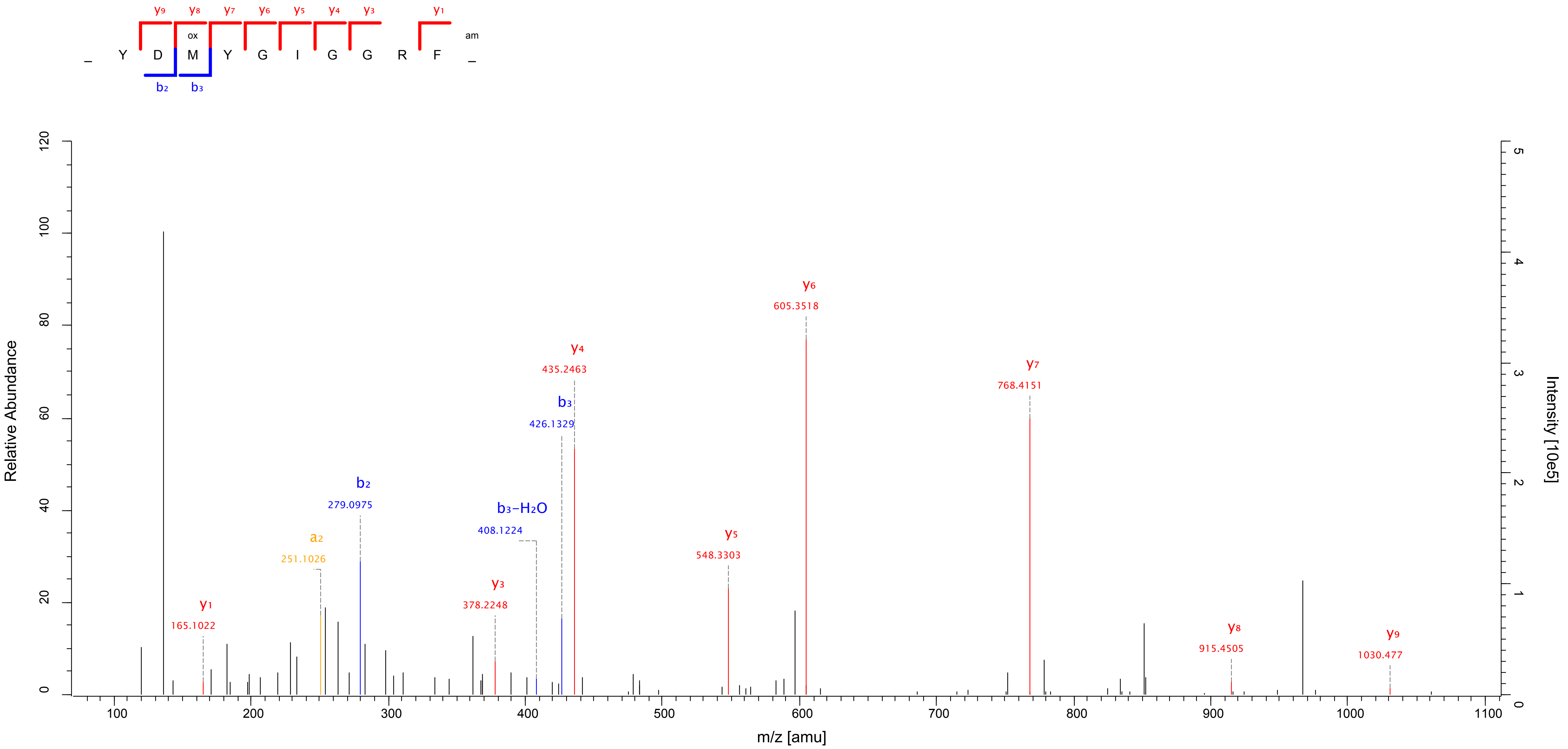

peptide ID 61

Source:	20121106_CO_0340GaJe_R02_2
Scannumber:	10423
Protein:	SinglePep78
Peptide Score:	86.01
Method:	FTMS; HCD; 1

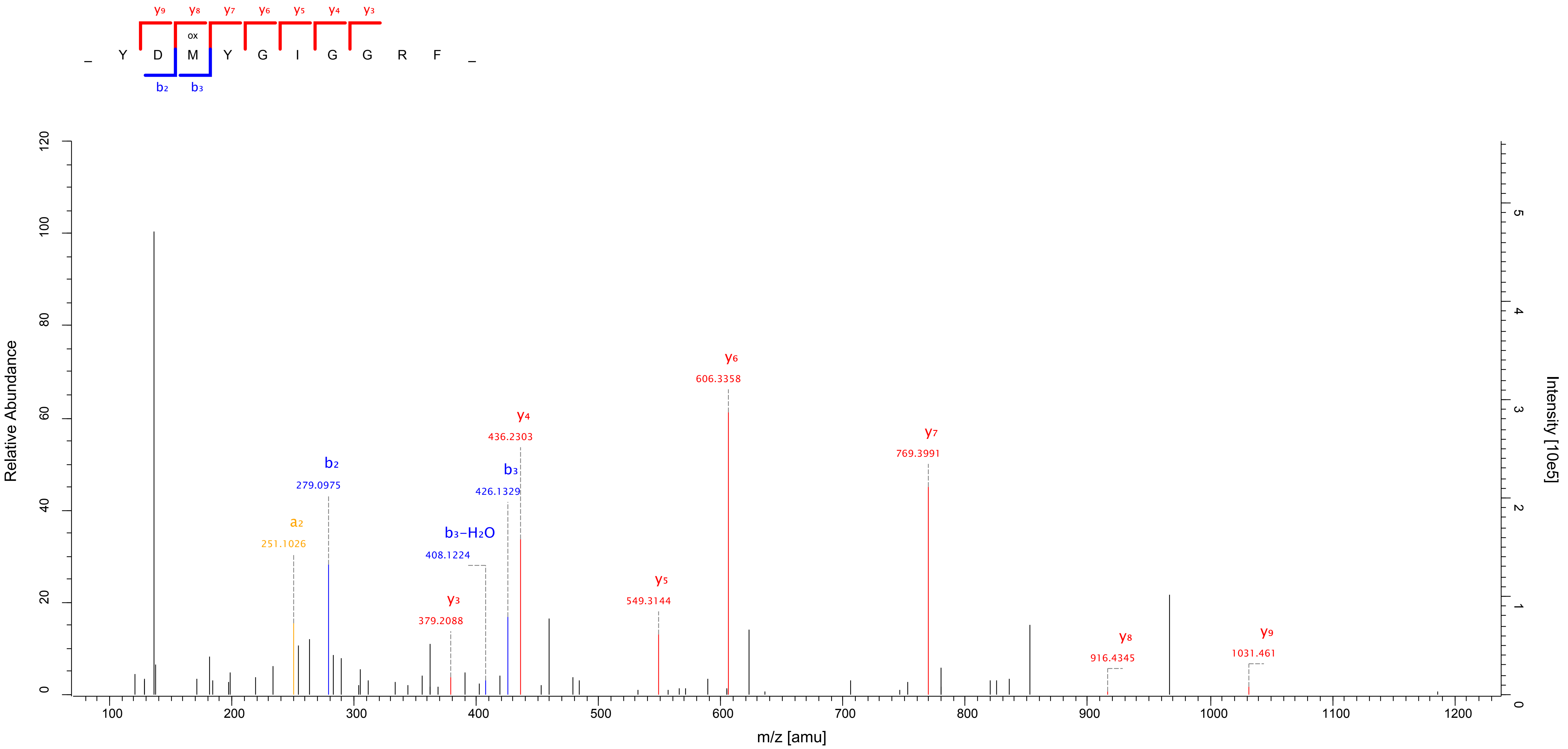

peptide ID 62

Source: 20120816_CO_0340GaJe_R02
Scannumber: 16130
Protein: pep_secretome_293
Peptide Score: 118.71
Method: FTMS; HCD; 1

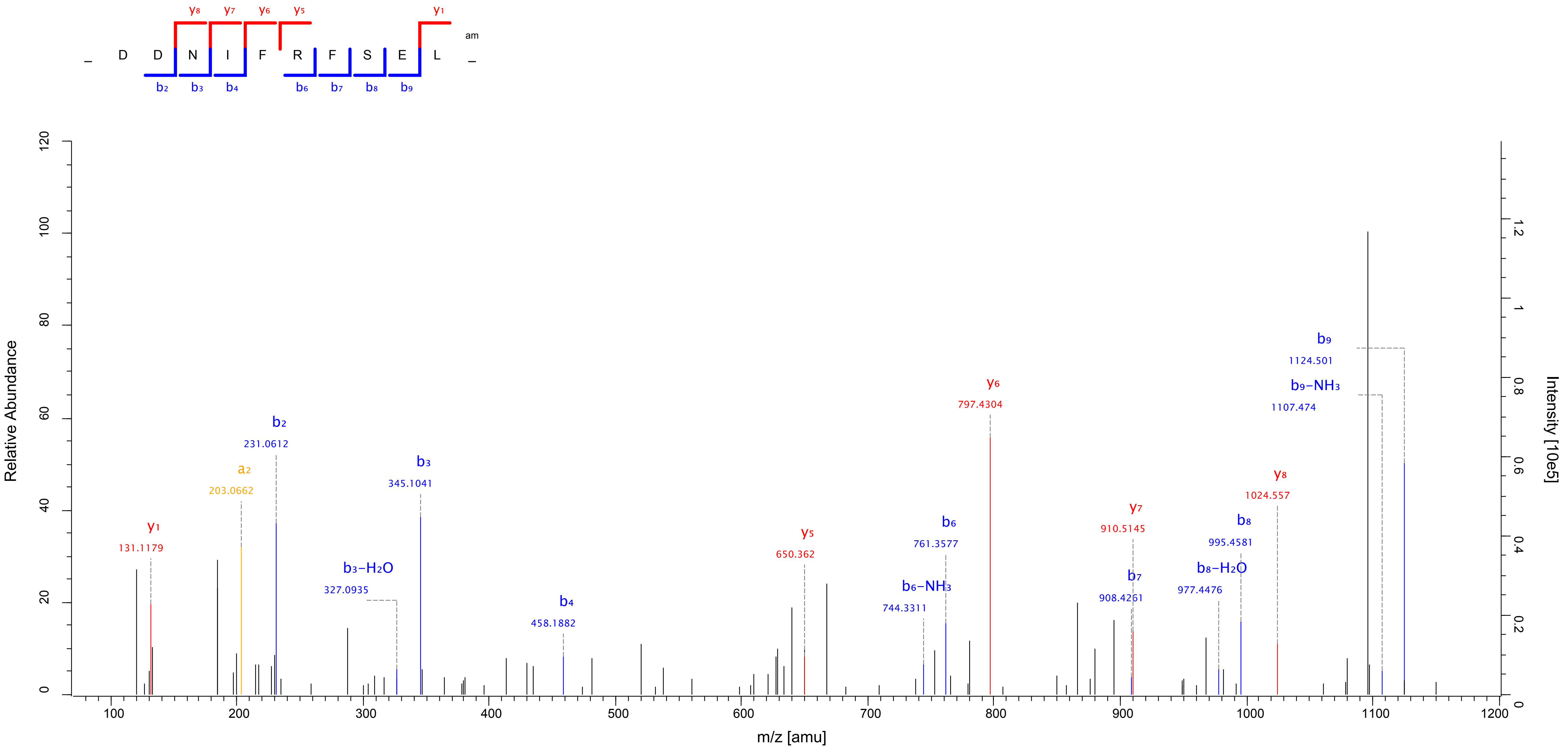

peptide ID 63

Source: 20120816_CO_0340GaJe_R02
Scannumber: 10960
Protein: pep_secretome_294
Peptide Score: 80.22
Method: FTMS; HCD; 1

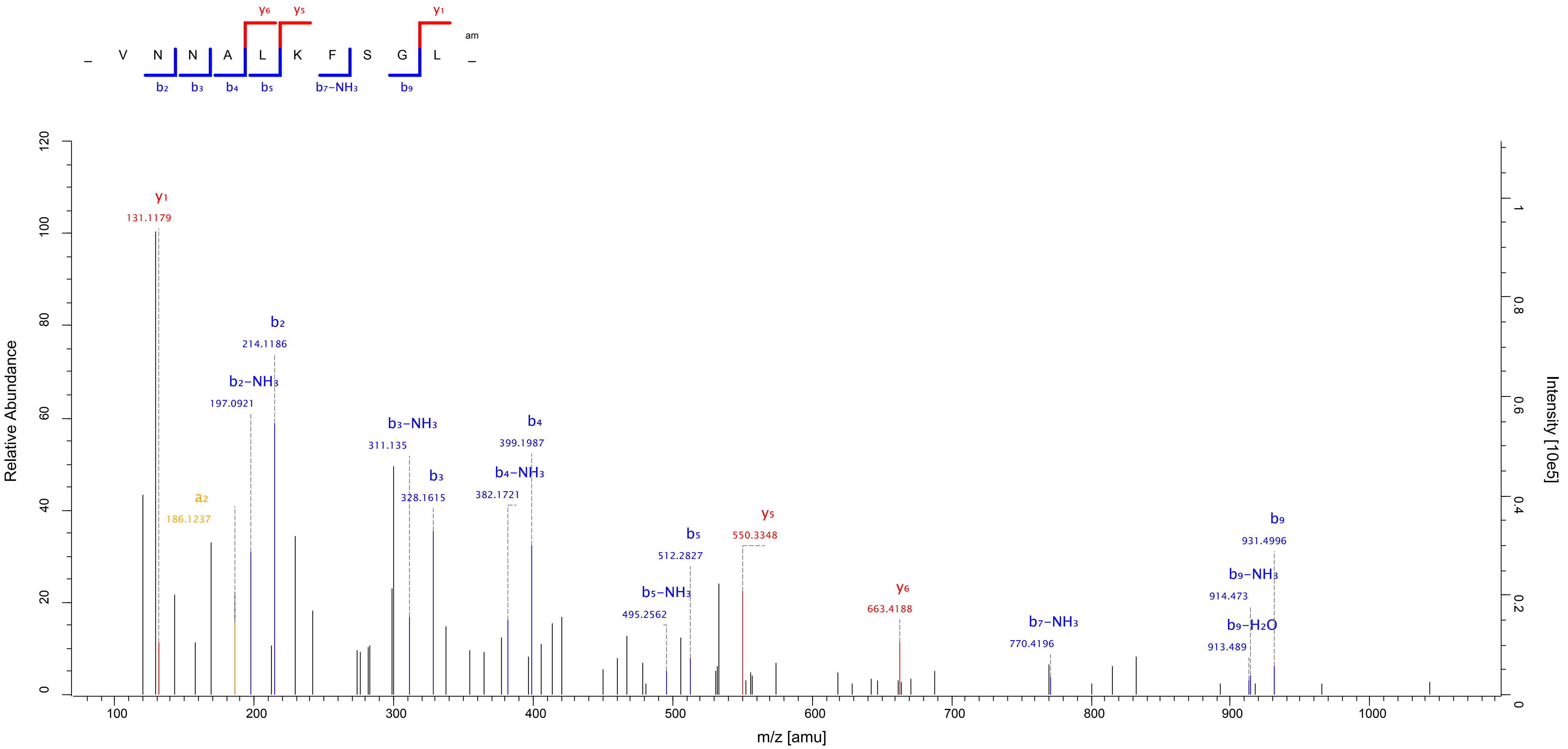

peptide ID 64

Source:	20120816_CO_0340GaJe_R02
Scannumber:	10500
Protein:	pep_secretome_32
Peptide Score:	74.46
Method:	FTMS; HCD; 1

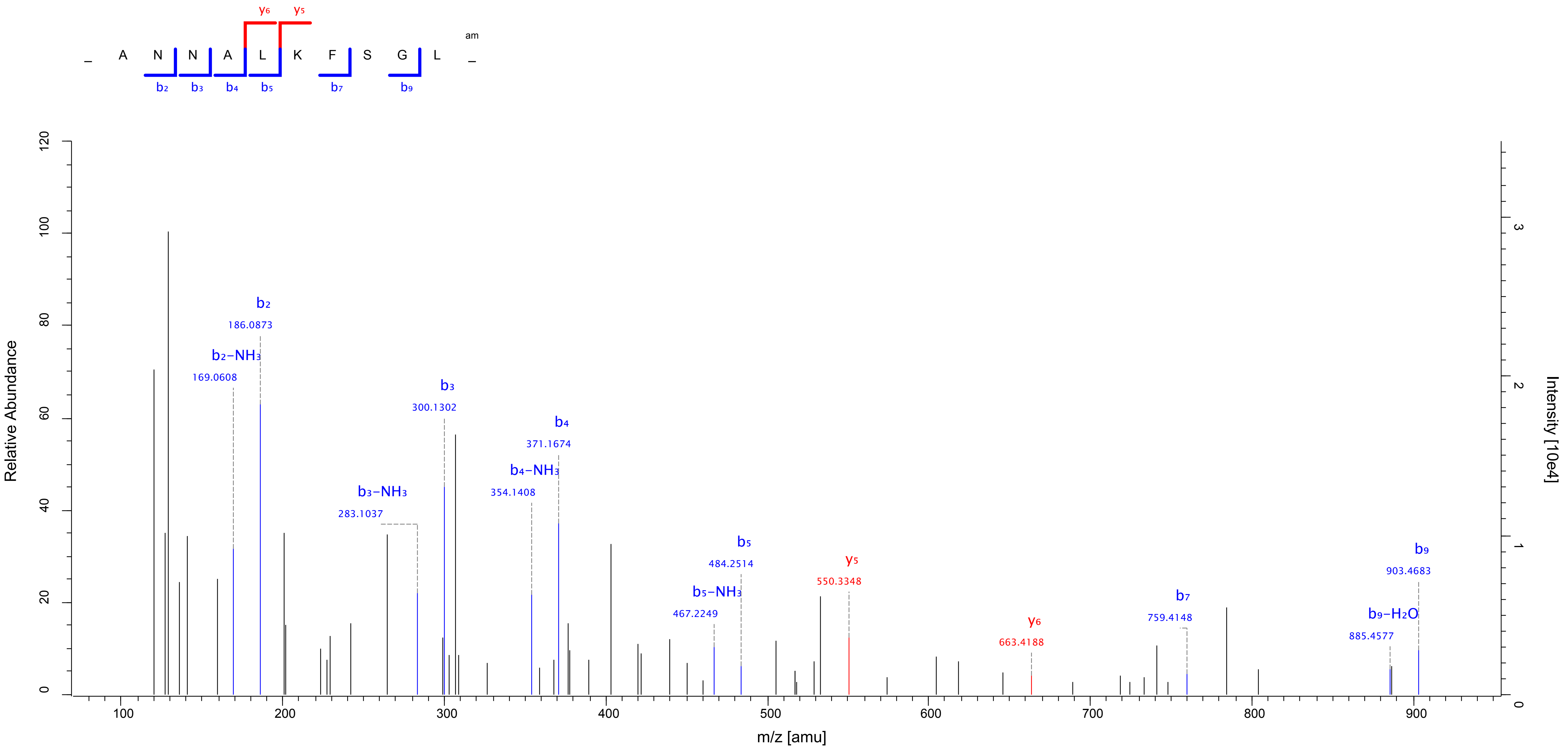

peptide ID 65

Source:	20121106_CO_0340GaJe_R02_2
Scannumber:	10221
Protein:	pep_secretome_26; pep_secretome_28; pep_secretome_30
Peptide Score:	121.73
Method:	FTMS; HCD; 1

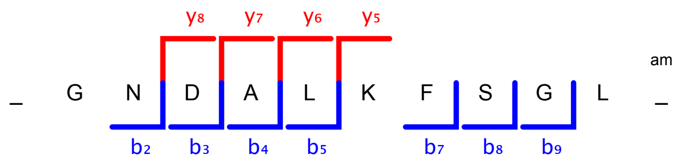

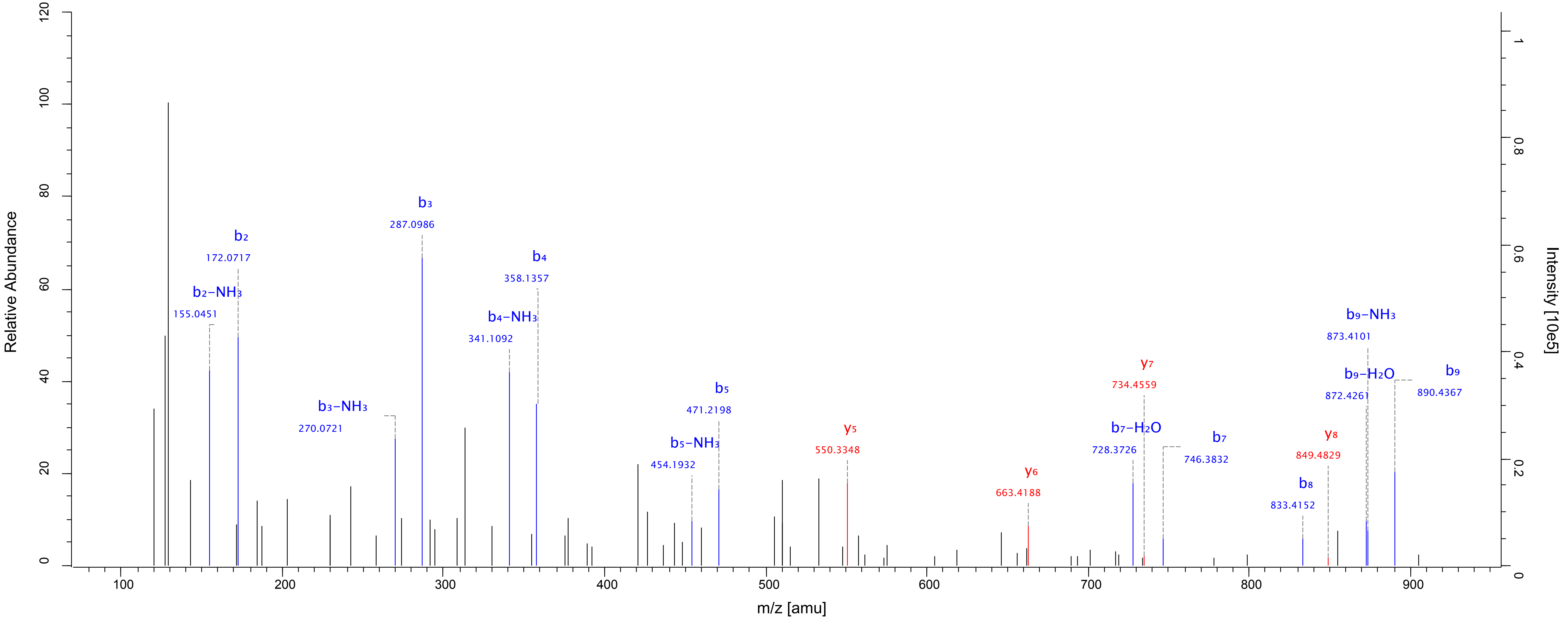

peptide ID 66

Source:	20121106_CO_0340GaJe_R02_2
Scannumber:	9929
Protein:	pep_5; pep_secretome_579
Peptide Score:	72.61
Method:	FTMS; HCD; 1

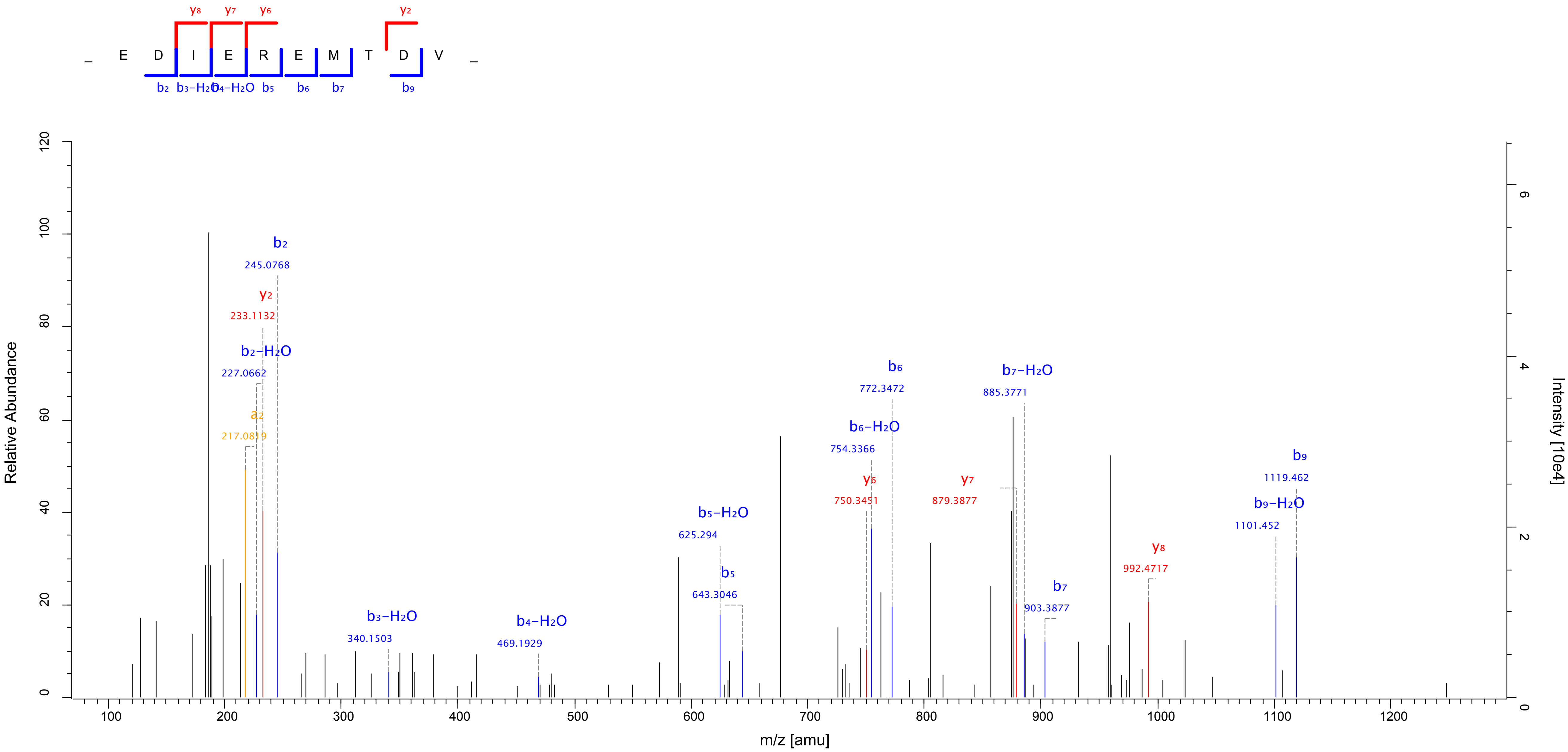

peptide ID 67

Source: 20120816_CO_0340GaJe_R02
Scannumber: 10799
Protein: SinglePep76
Peptide Score: 88.06
Method: FTMS; HCD; 1

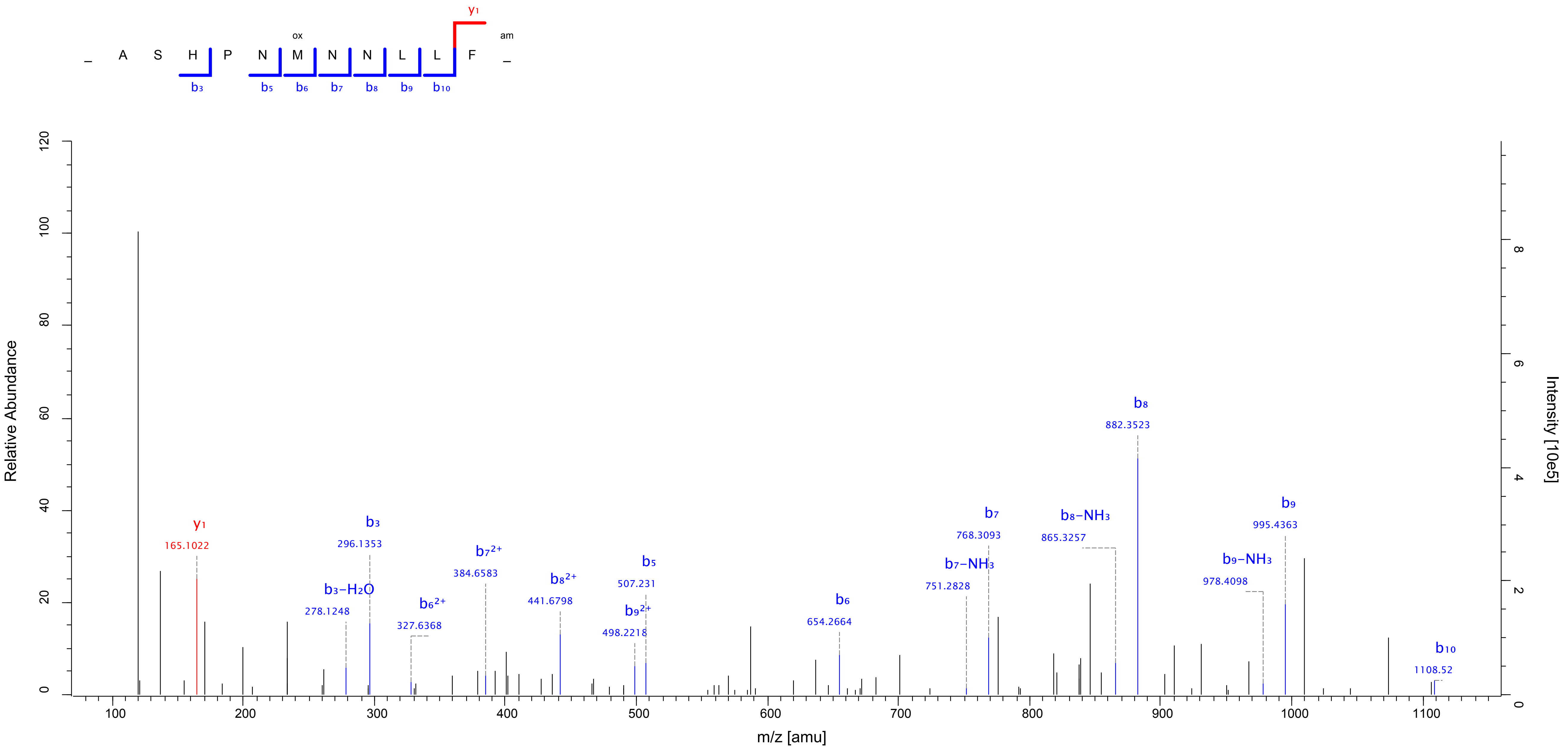

peptide ID 68

Source: 20120816_CO_0340GaJe_R02
Scannumber: 10511
Protein: orf_6877; orf_7269; pep_176; pep_secretome_482
Peptide Score: 121.83
Method: FTMS; HCD; 1

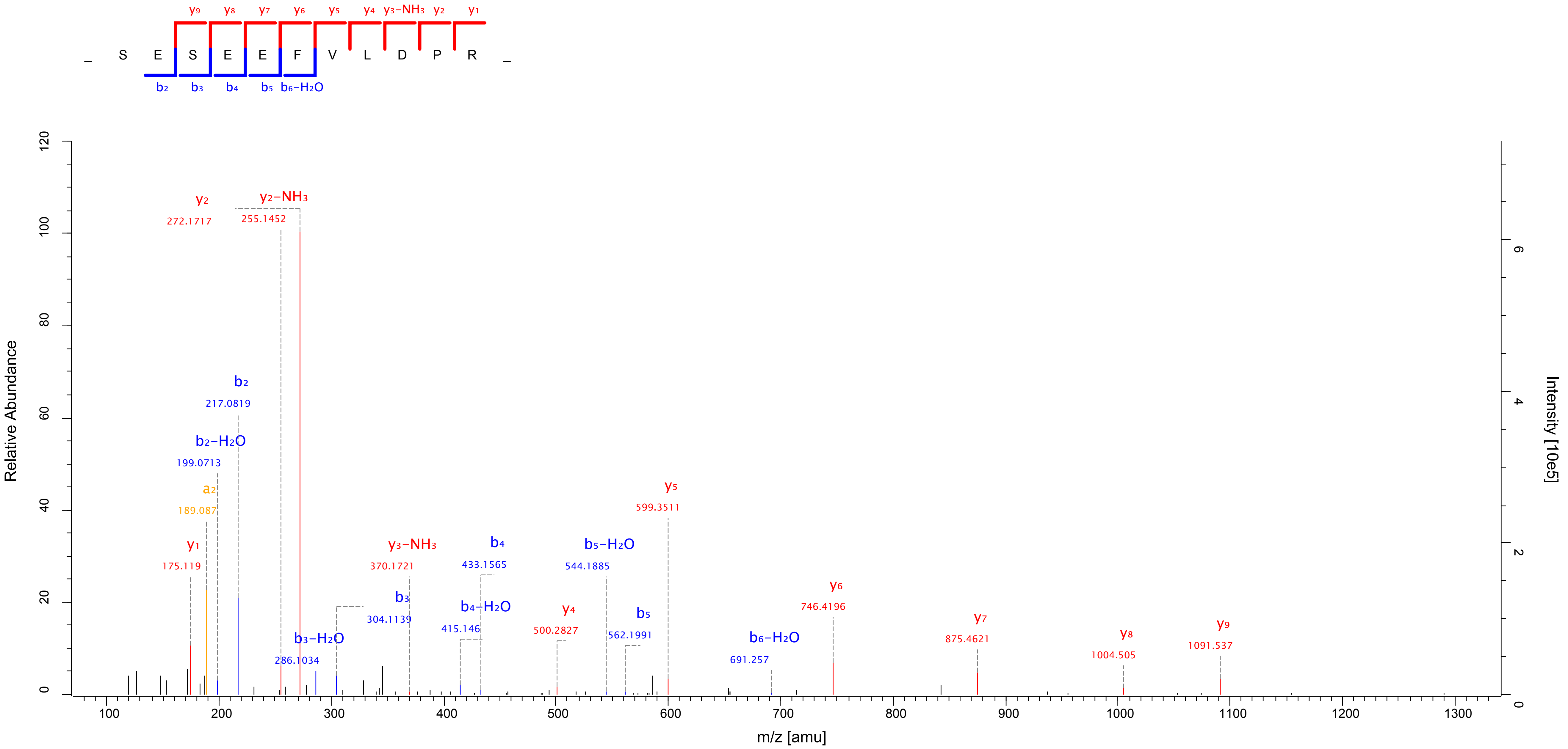

peptide ID 69

Source:	20121106_CO_0340GaJe_R02_2
Scannumber:	16832
Protein:	pep_177; pep_secretome_483
Peptide Score:	56.96
Method:	FTMS; HCD; 1

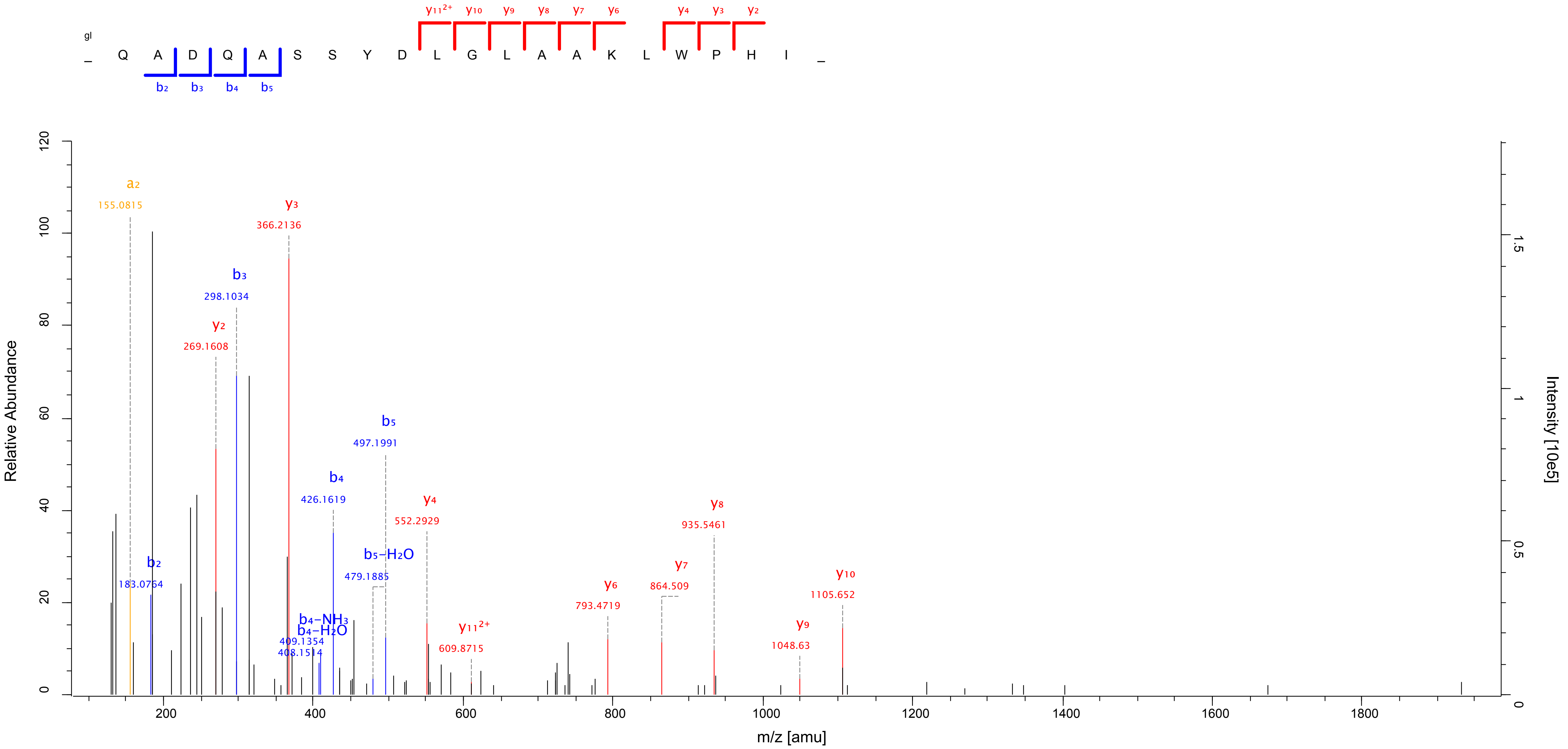

Source: 20120816_CO_0340GaJe_R02
Scannumber: 6558
Protein: pep_secretome_1211
Peptide Score: 167.24
Method: FTMS; HCD; 1

peptide ID 70

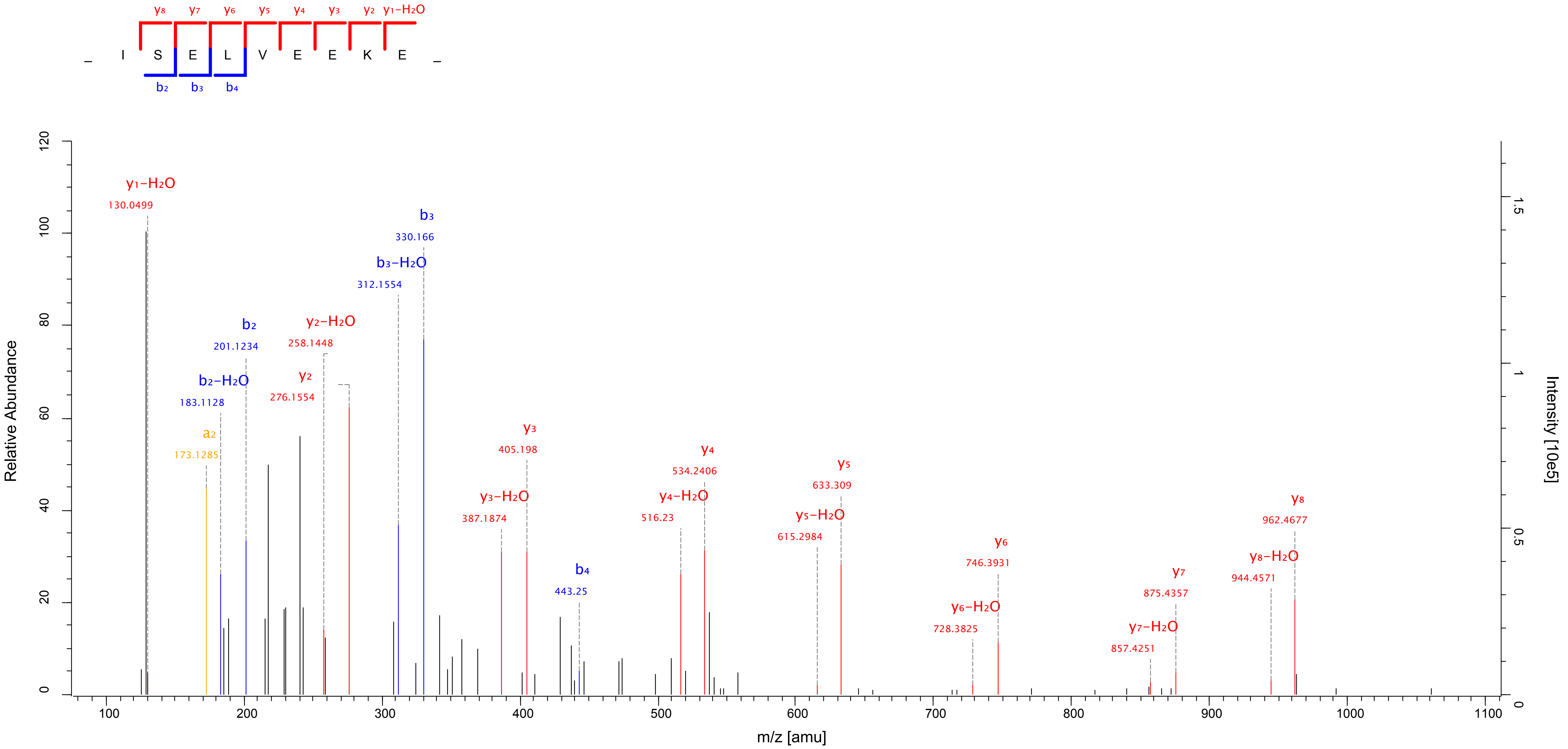

peptide ID 71

Source: 20121106_CO_0340GaJe_R02_2
Scannumber: 7135
Protein: pep_secretome_1213; pep_secretome_26648
Peptide Score: 108.98
Method: FTMS; HCD; 1

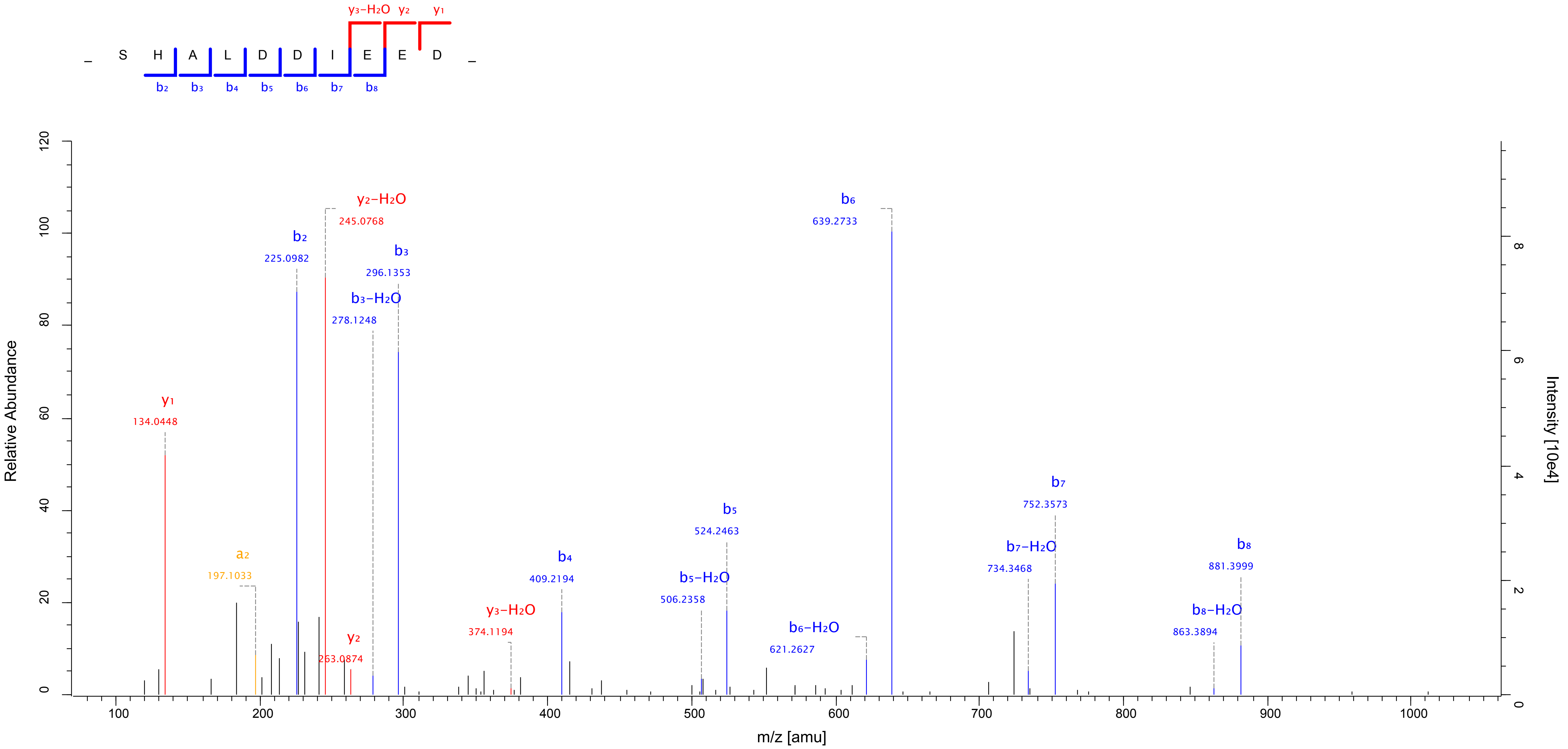

peptide ID 72

Source: 20120816_CO_0340GaJe_R02
Scannumber: 16767
Protein: pep_secretome_1219
Peptide Score: 66.96
Method: FTMS; HCD; 1

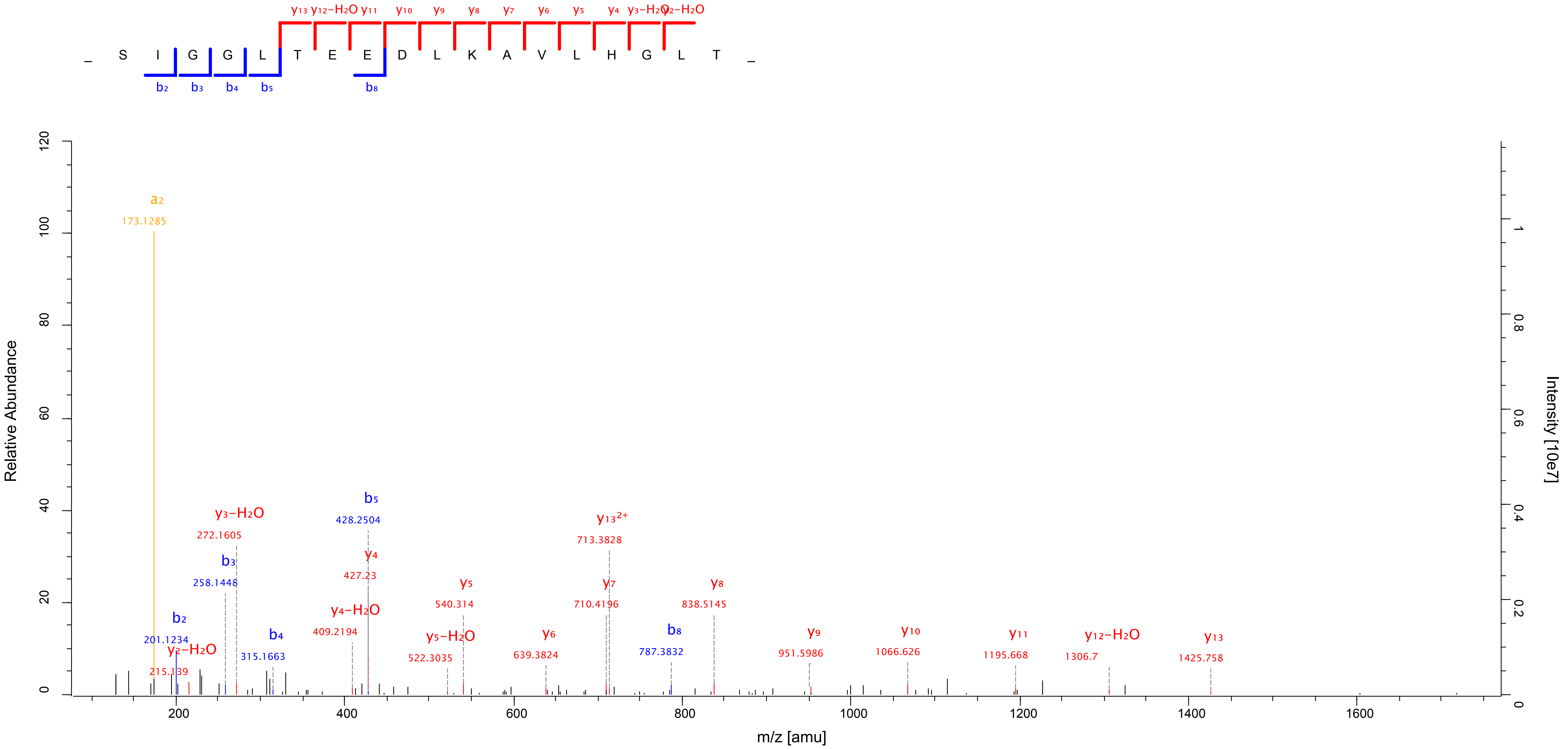

peptide ID 73

Source:	20121106_CO_0340GaJe_R02_2
Scannumber:	15246
Protein:	pep_66; pep_secretome_126; pep_secretome_19643
Peptide Score:	34.5
Method:	FTMS; HCD; 1

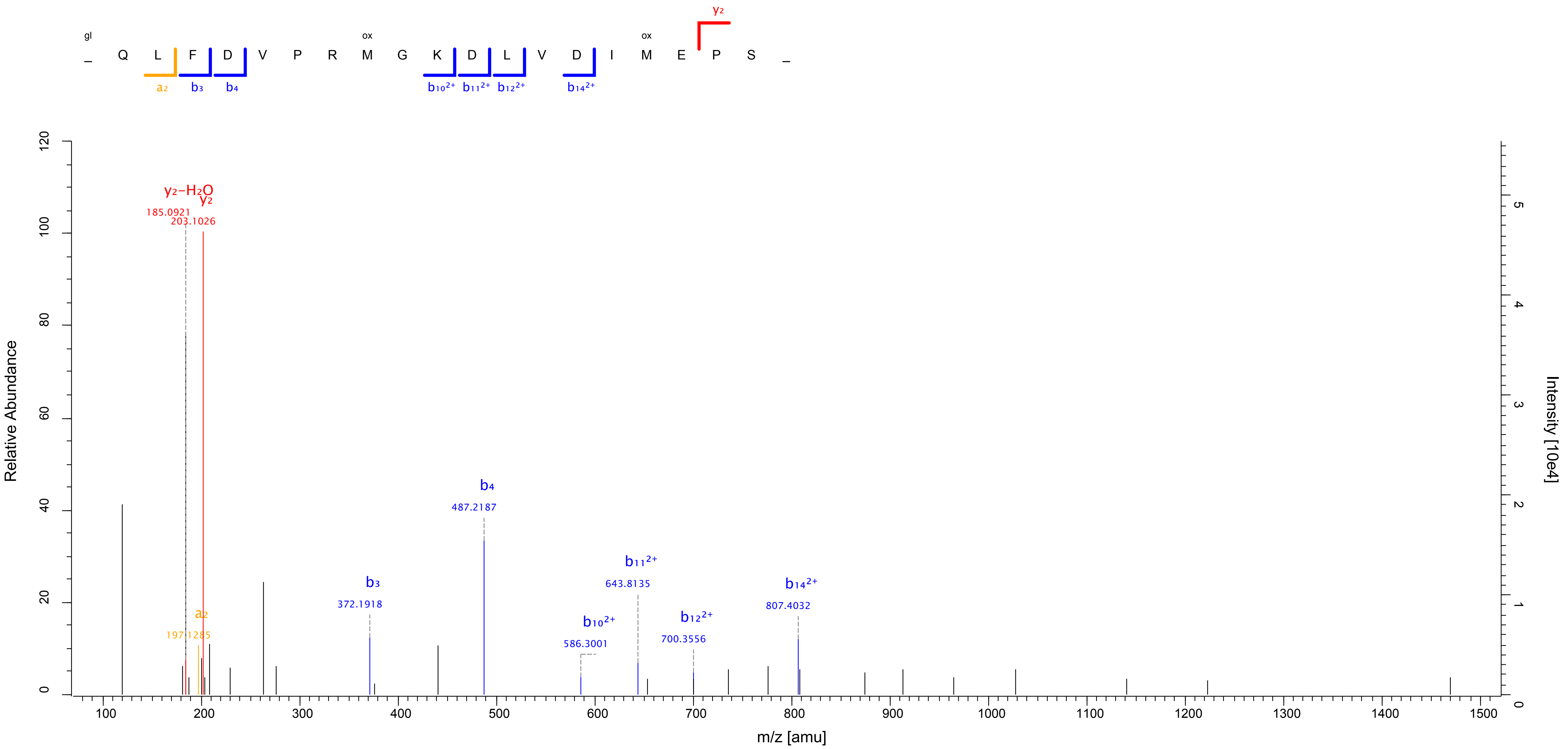

peptide ID 74

Source: 20120816_CO_0340GaJe_R02
Scannumber: 10975
Protein: orf_18496; pep_68; pep_secretome_128
Peptide Score: 134.08
Method: FTMS; HCD; 1

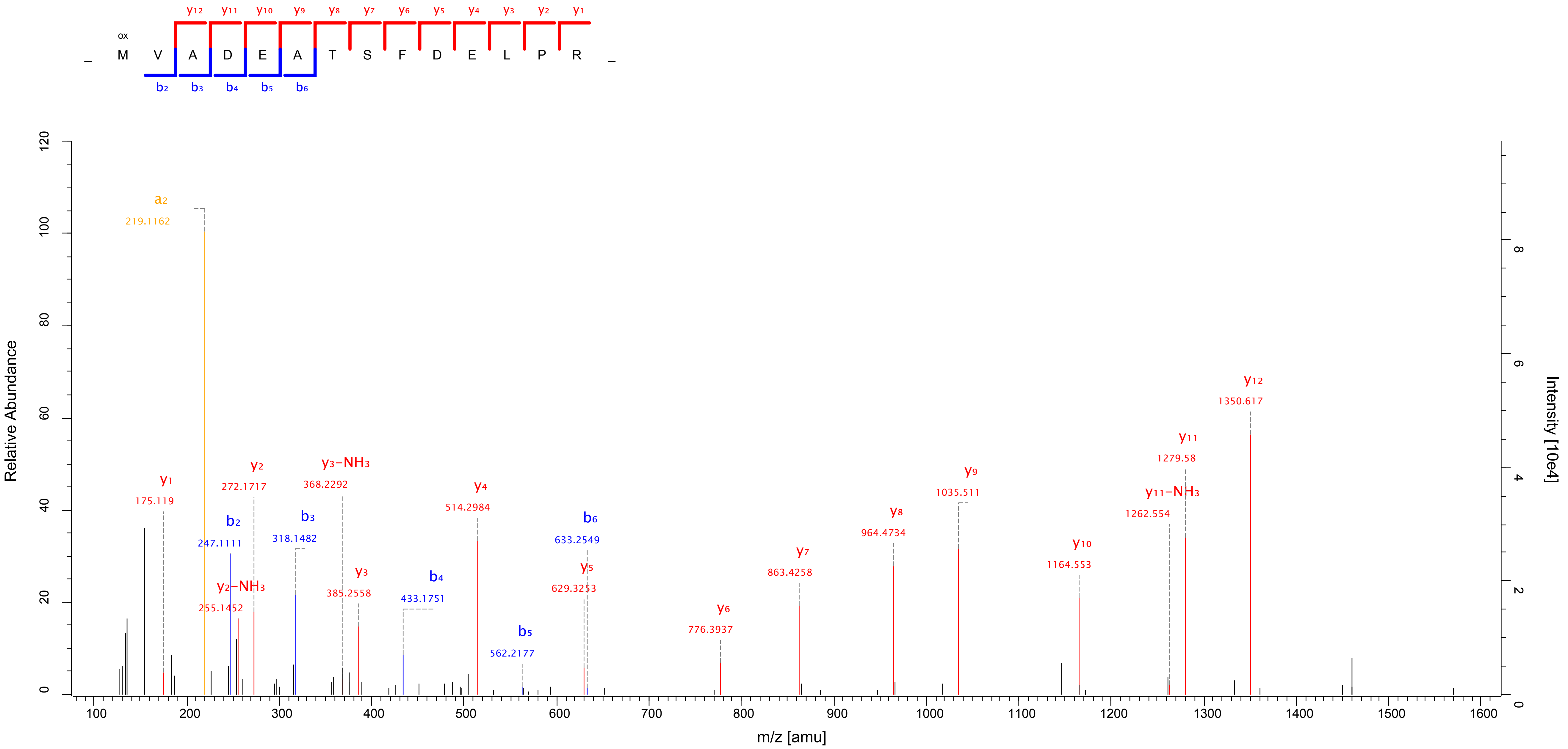

peptide ID 75

Source: 20120816_CO_0340GaJe_R02
Scannumber: 7055
Protein: orf_18496; pep_68; pep_secretome_128
Peptide Score: 109.84
Method: FTMS; HCD; 1

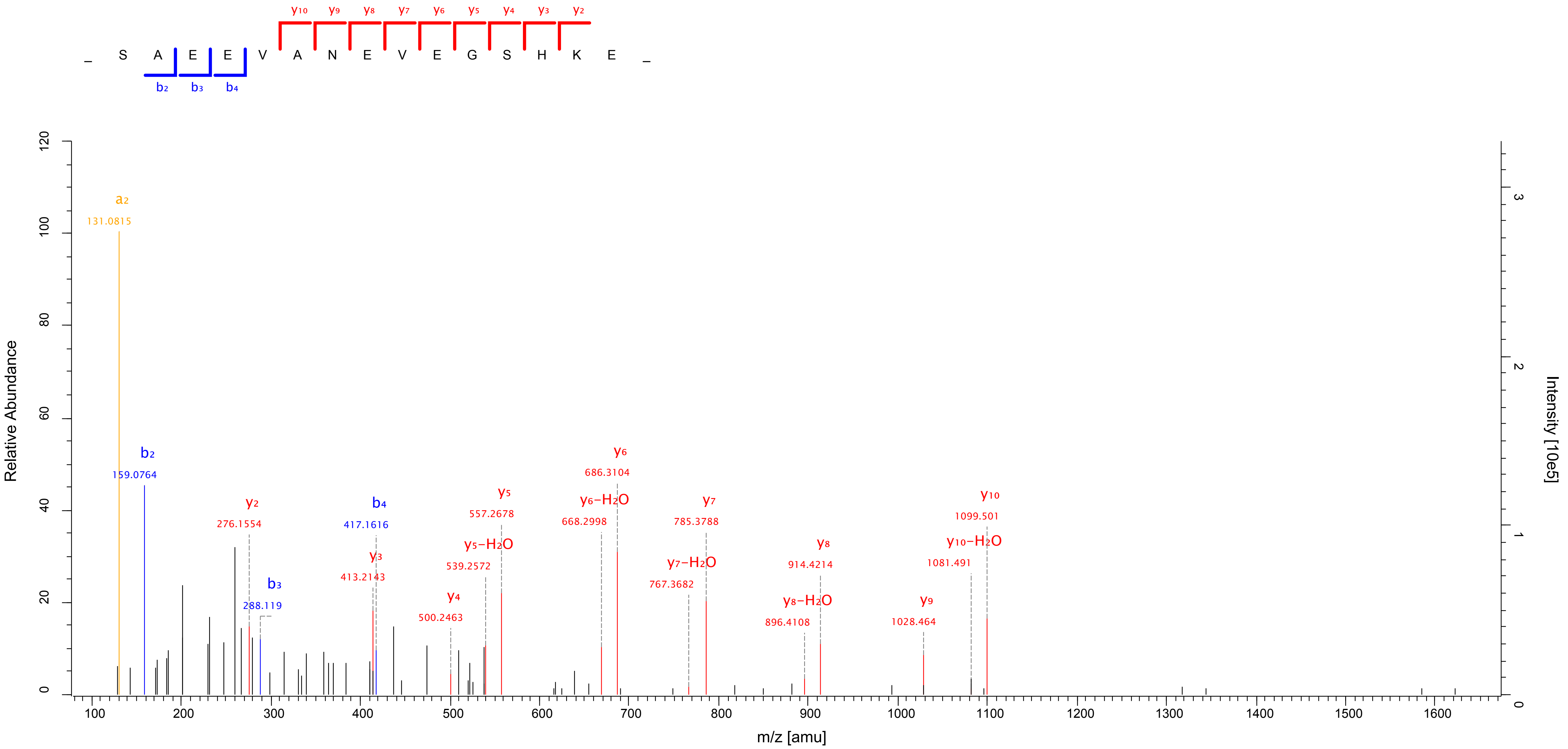

Source: 20120816_CO_0340GaJe_R02
Scannumber: 7008
Protein: pep_secretome_22782
Peptide Score: 123.63
Method: FTMS; HCD; 1

peptide ID 76

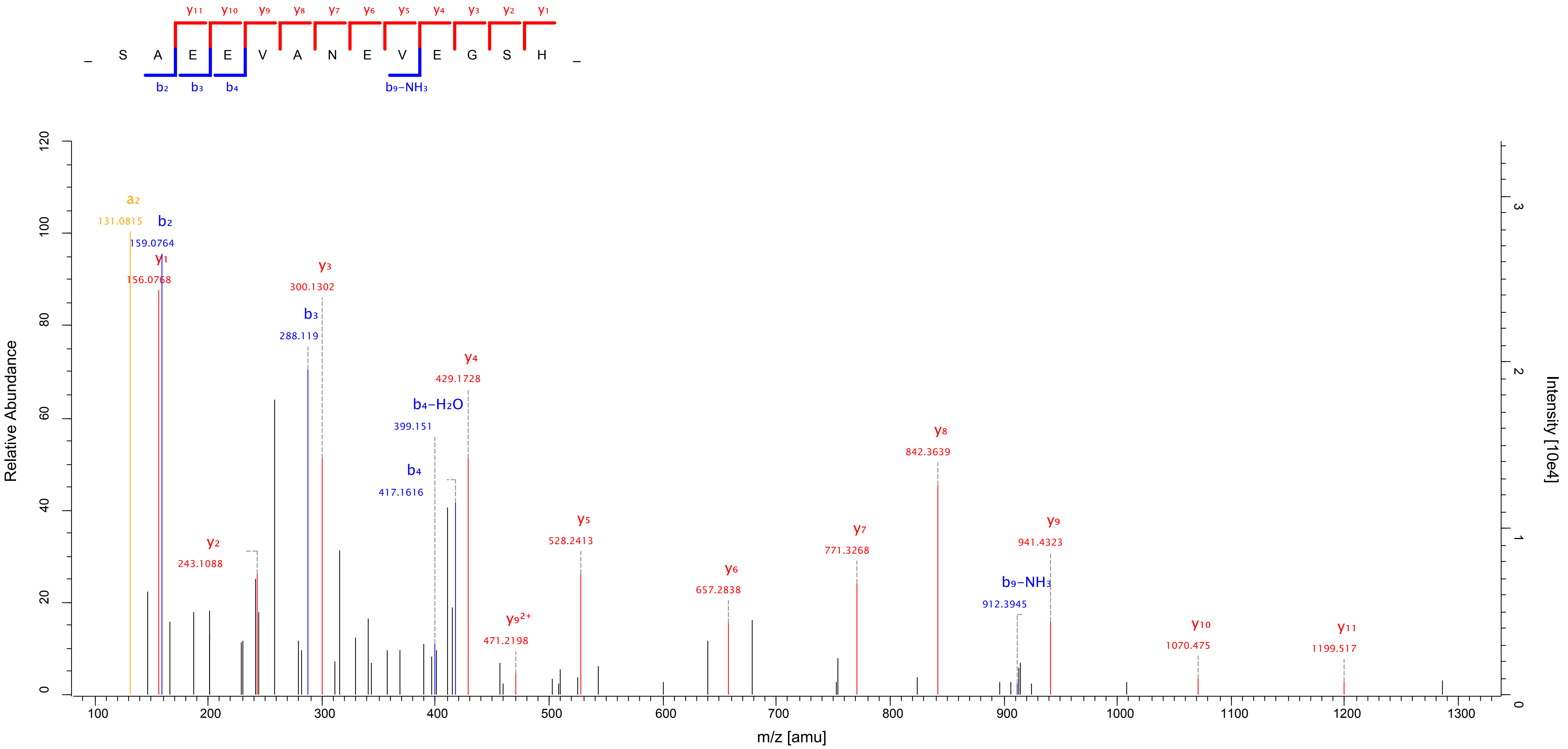

peptide ID 77

Source:	20121106_CO_0340GaJe_R02_2
Scannumber:	5800
Protein:	pep_71; pep_secretome_134; pep_secretome_131; pep_secretome_140
Peptide Score:	69.44
Method:	FTMS; HCD; 1

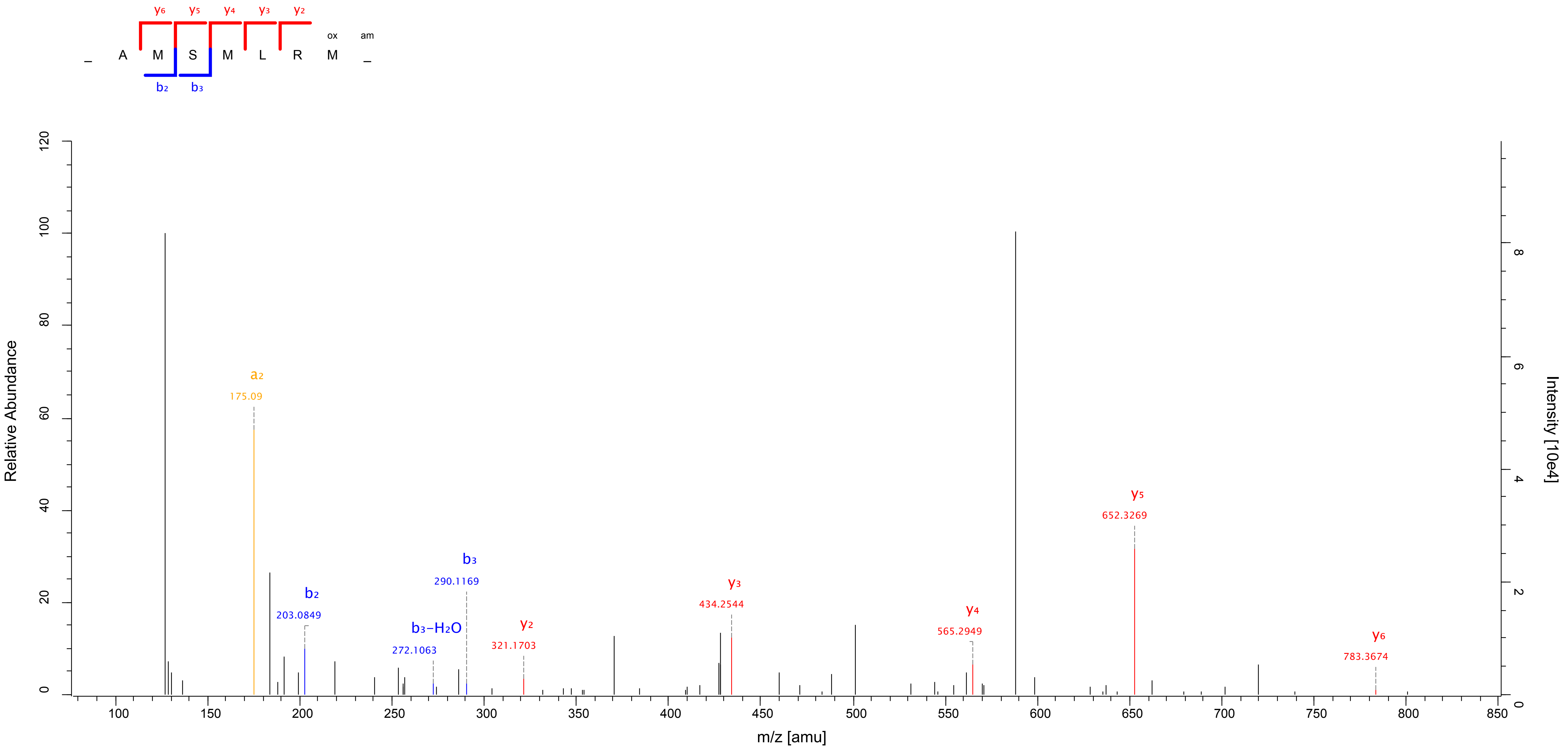

peptide ID 78

Source: 20120816_CO_0340GaJe_R02
Scannumber: 7864
Protein: pep_secretome_142; pep_secretome_144; pep_secretome_146
Peptide Score: 63.58
Method: FTMS; HCD; 1

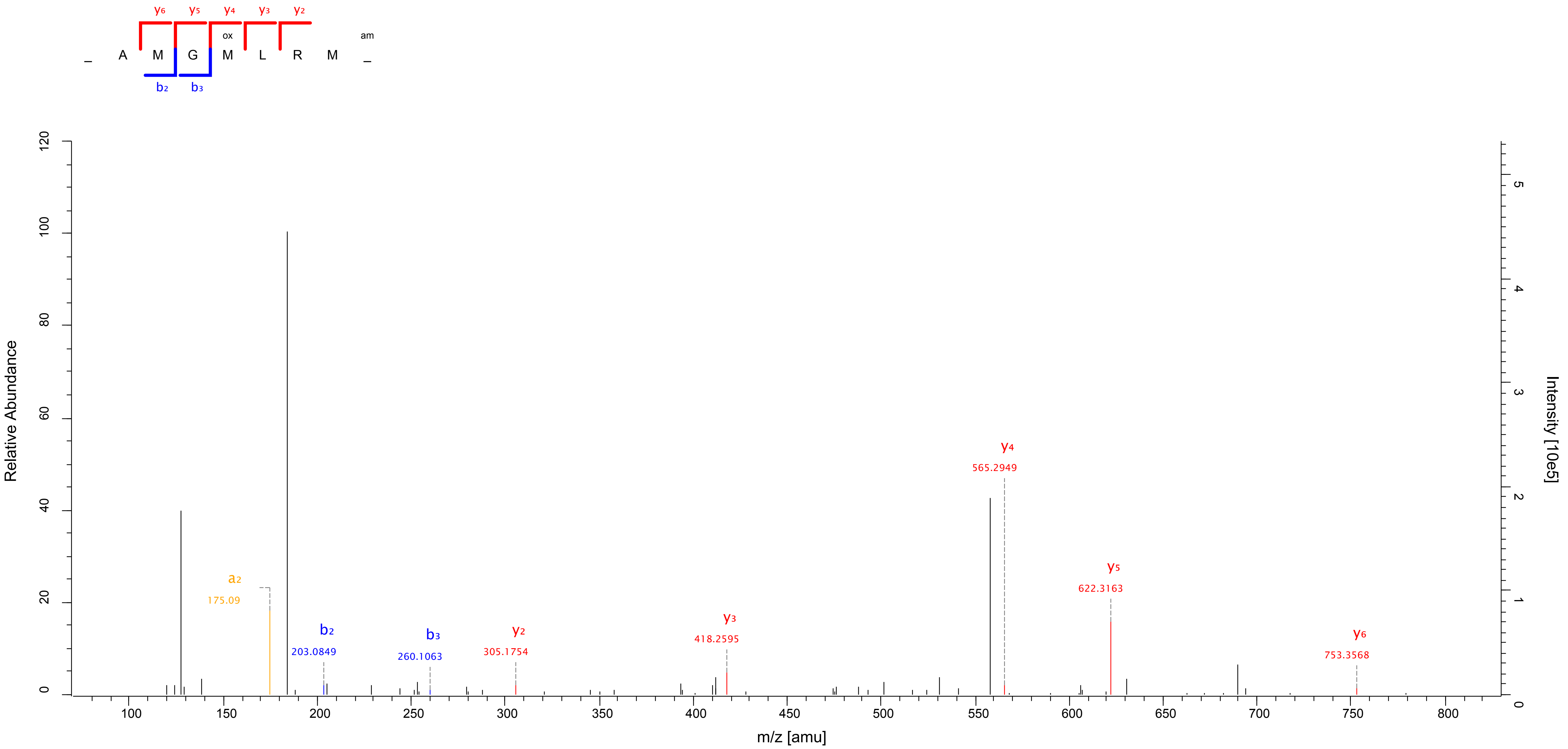

peptide ID 79

Source:	20121106_CO_0340GaJe_R02_2
Scannumber:	8024
Protein:	pep_secretome_133; pep_secretome_22803
Peptide Score:	74.46
Method:	FTMS; HCD; 1

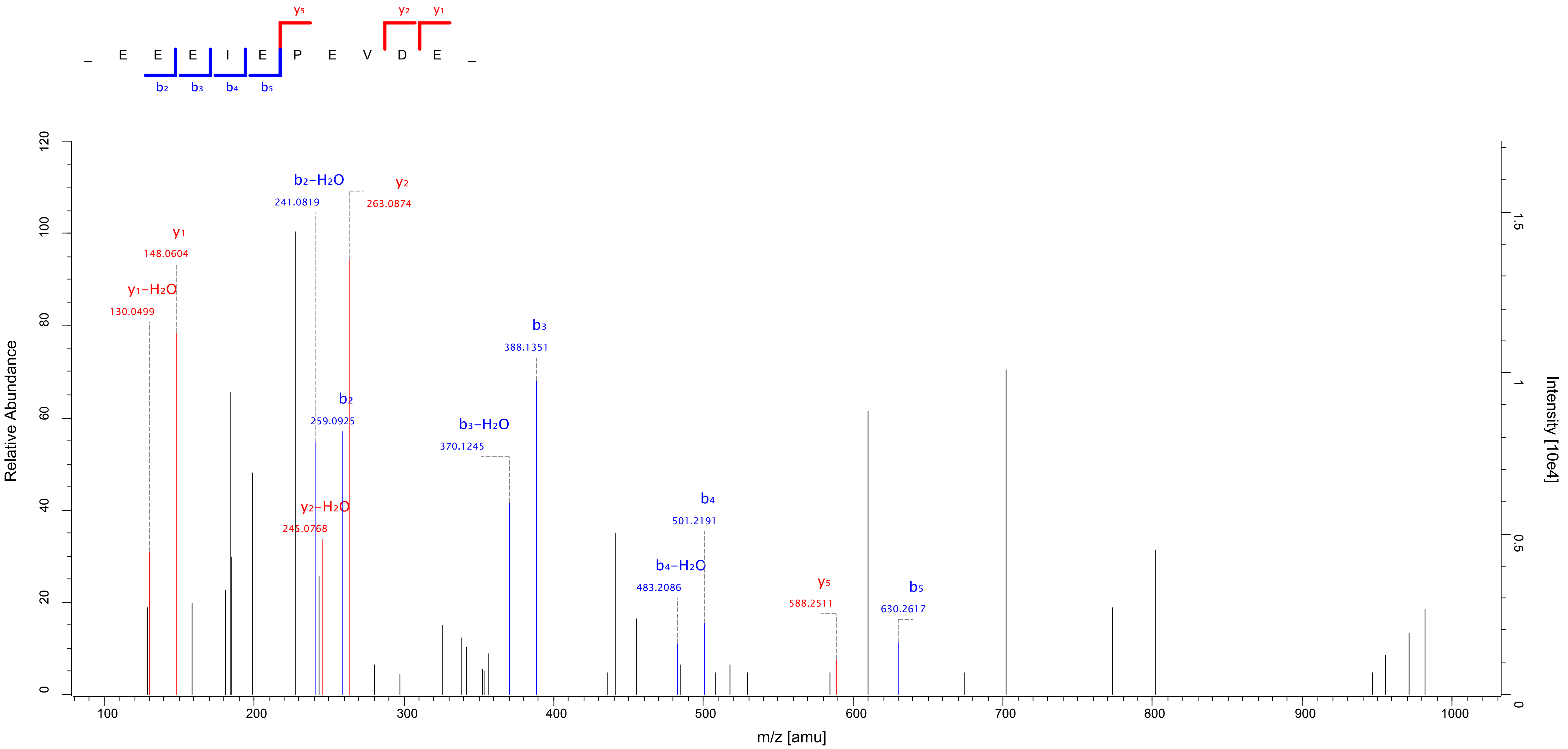

peptide ID 80

Source: 20120515_CO_0340GaJe_R01
Scannumber: 14241
Protein: pep_226; pep_secretome_623
Peptide Score: 108.71
Method: FTMS; HCD; 1

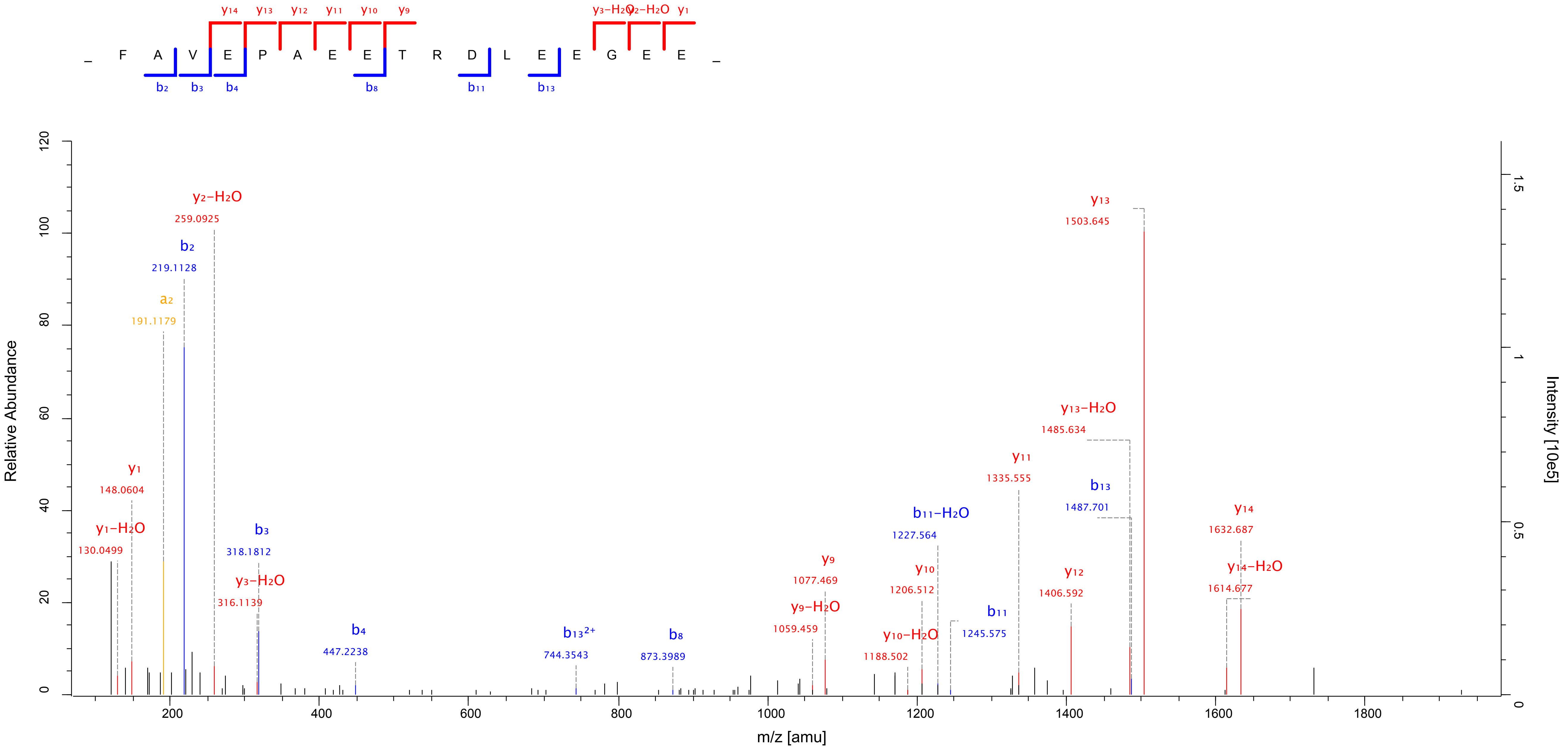

peptide ID 81

Source: 20120816_CO_0340GaJe_R02
Scannumber: 8125
Protein: pep_secretome_23546
Peptide Score: 52.72
Method: FTMS; HCD; 1

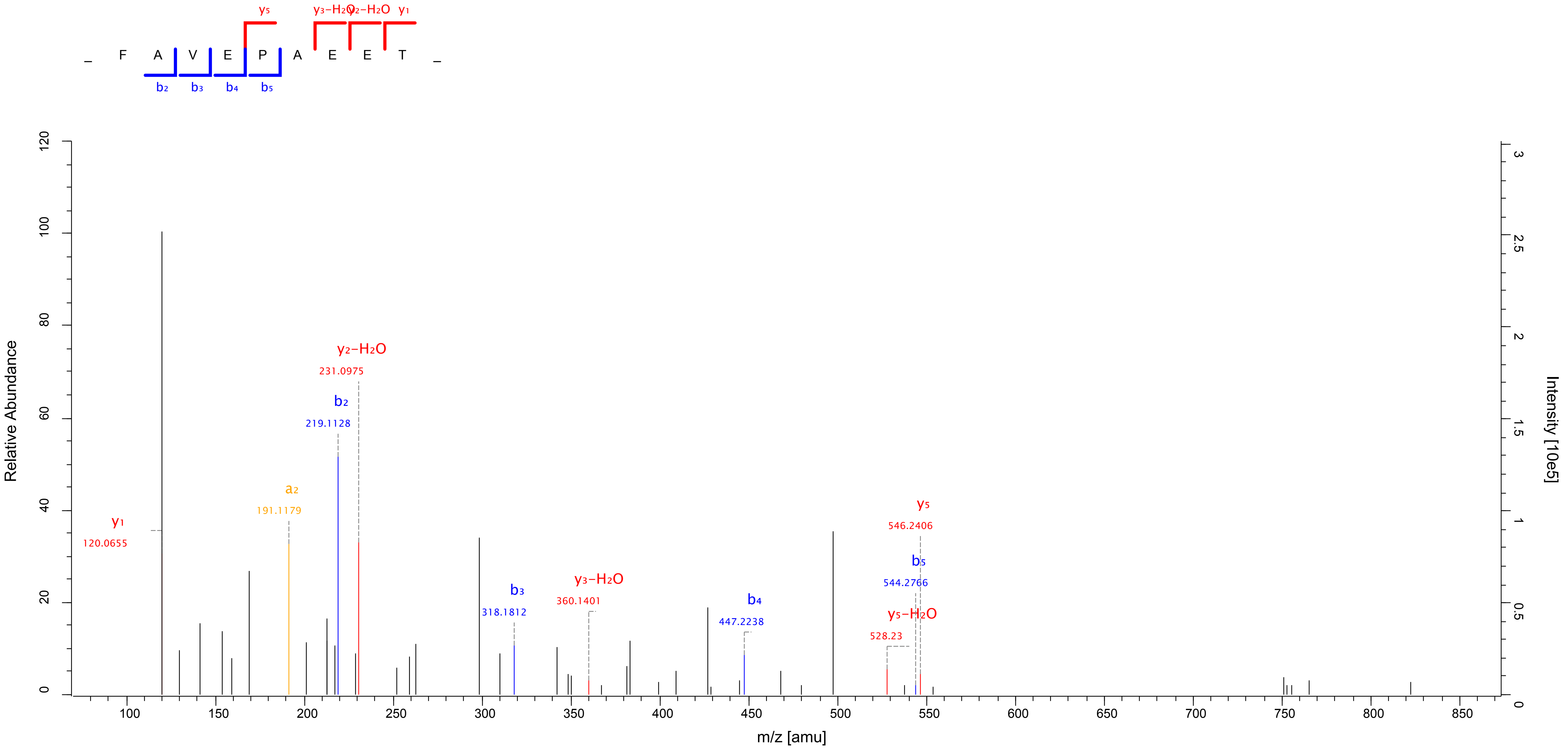

peptide ID 82

Source: 20120515_CO_0340GaJe_R01
Scannumber: 5714
Protein: orf_7277; orf_11195; pep_secretome_625
Peptide Score: 156.5
Method: FTMS; HCD; 1

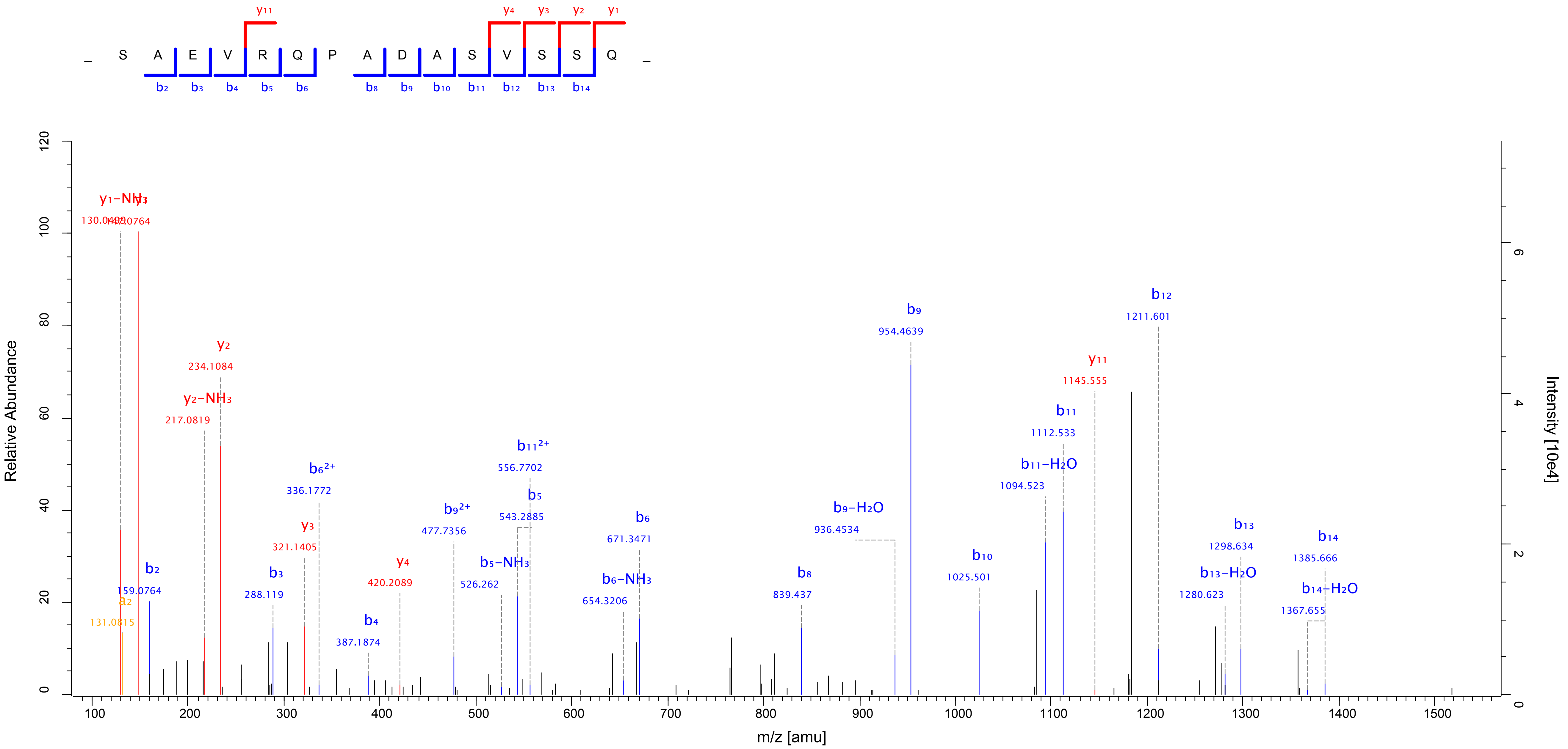

peptide ID 83

Source: 20120816_CO_0340GaJe_R02
Scannumber: 14863
Protein: pep_secretome_3207
Peptide Score: 103.22
Method: FTMS; HCD; 1

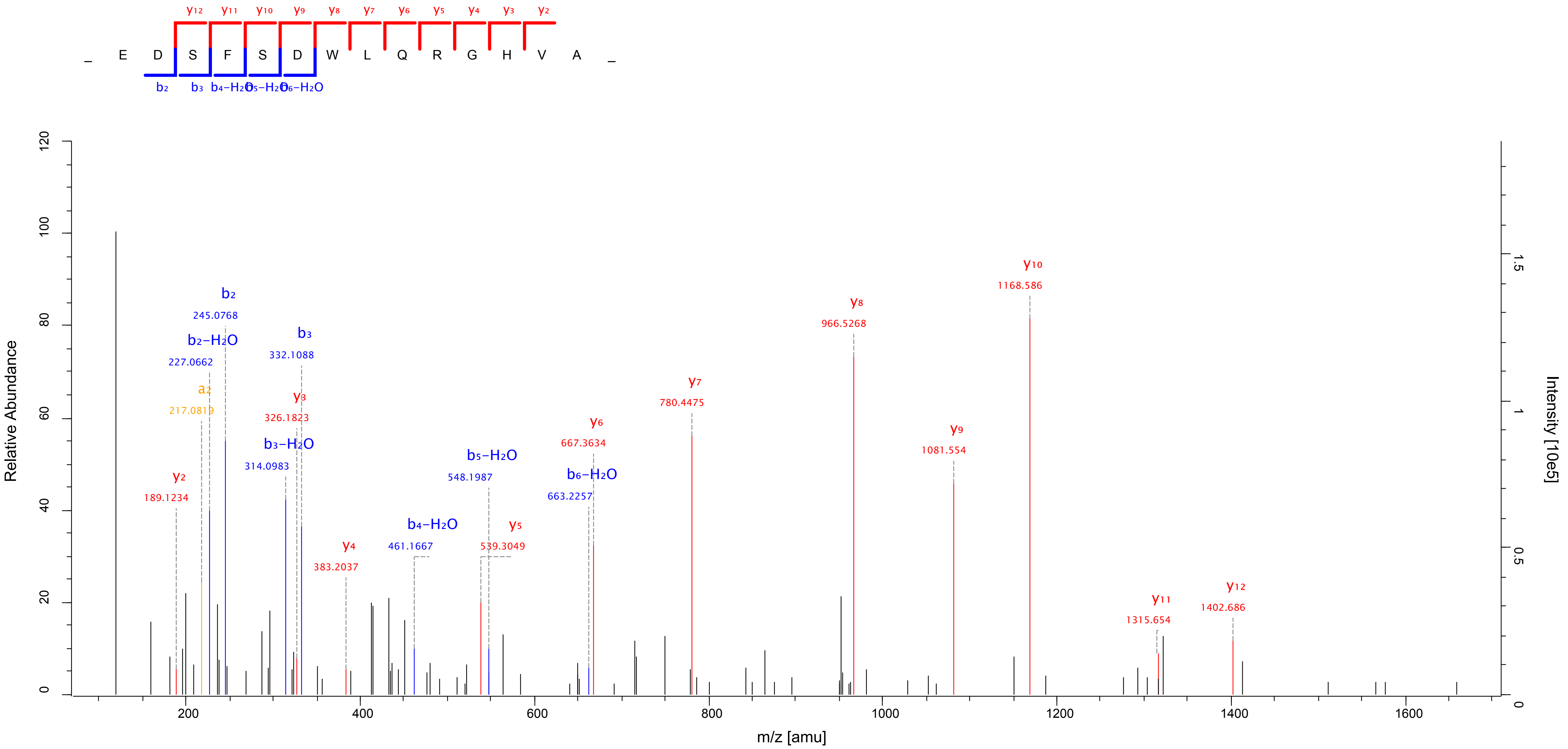

peptide ID 84

Source: 20120816_CO_0340GaJe_R02
Scannumber: 14419
Protein: pep_secretome_8447
Peptide Score: 51.03
Method: FTMS; HCD; 1

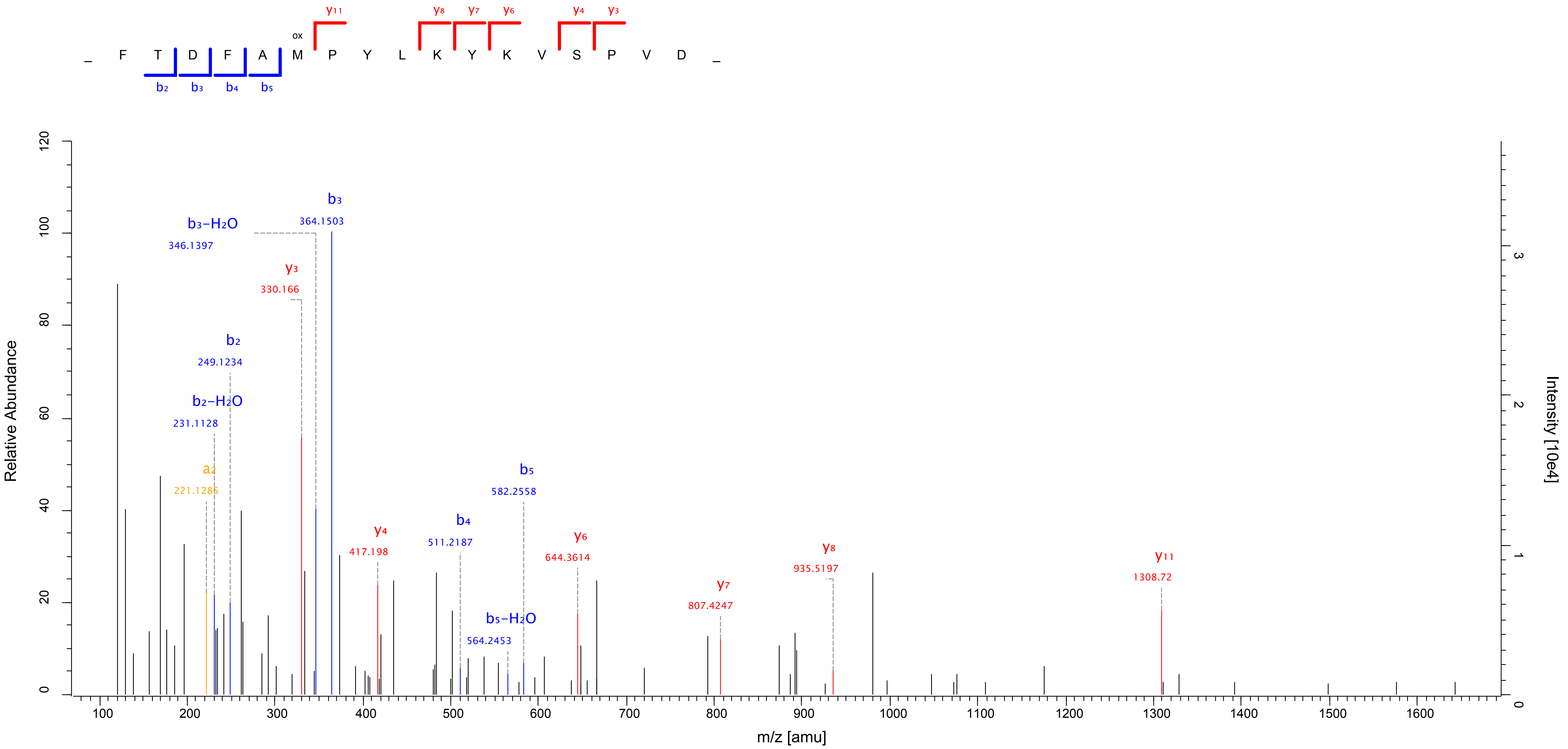

peptide ID 85

Source: 20120816_CO_0340GaJe_R02
Scannumber: 9146
Protein: pep_secretome_528
Peptide Score: 154.91
Method: FTMS; HCD; 1

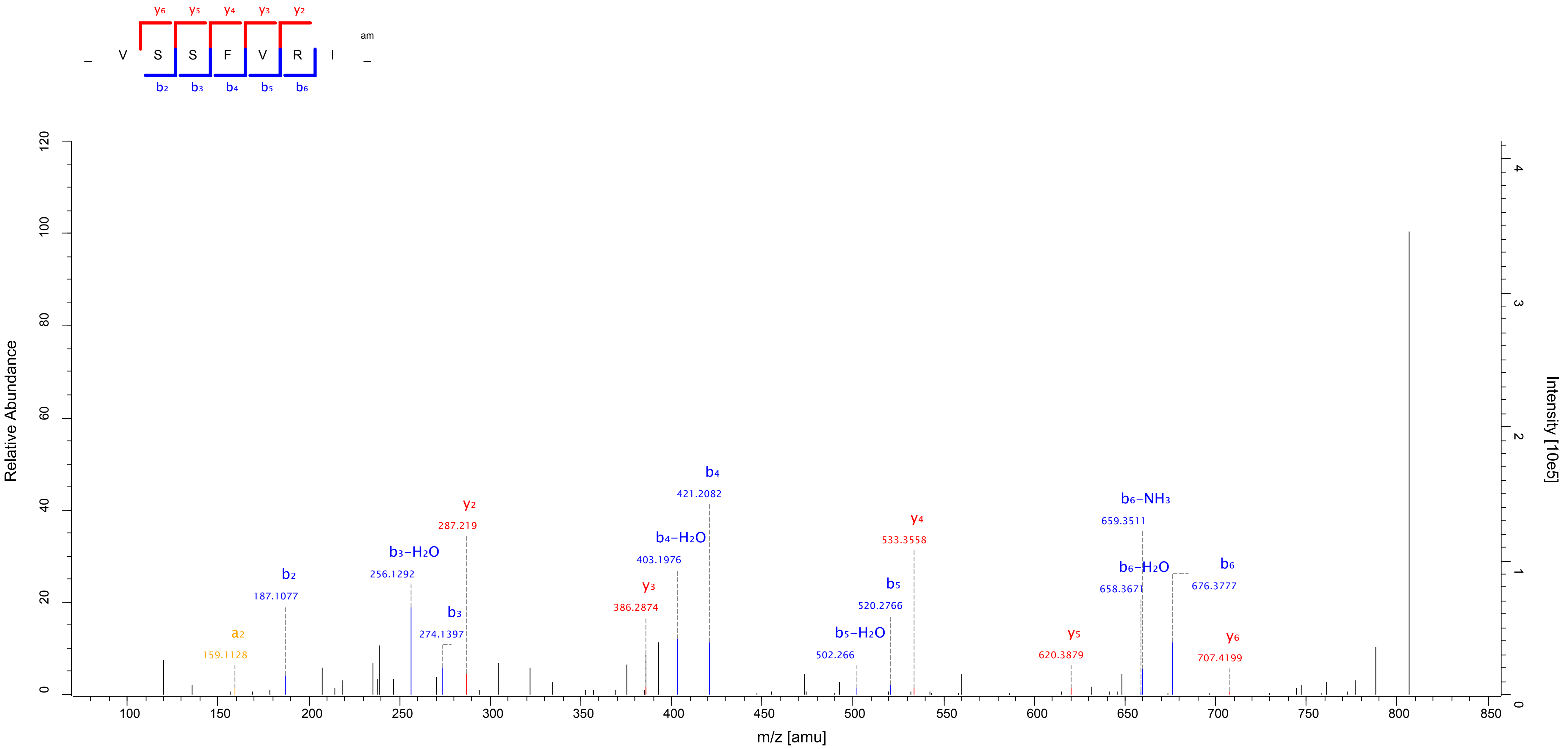

peptide ID 86

Source: 20121106_CO_0340GaJe_R02_2
Scannumber: 7182
Protein: pep_secretome_533; pep_secretome_540
Peptide Score: 163.53
Method: FTMS; HCD; 1

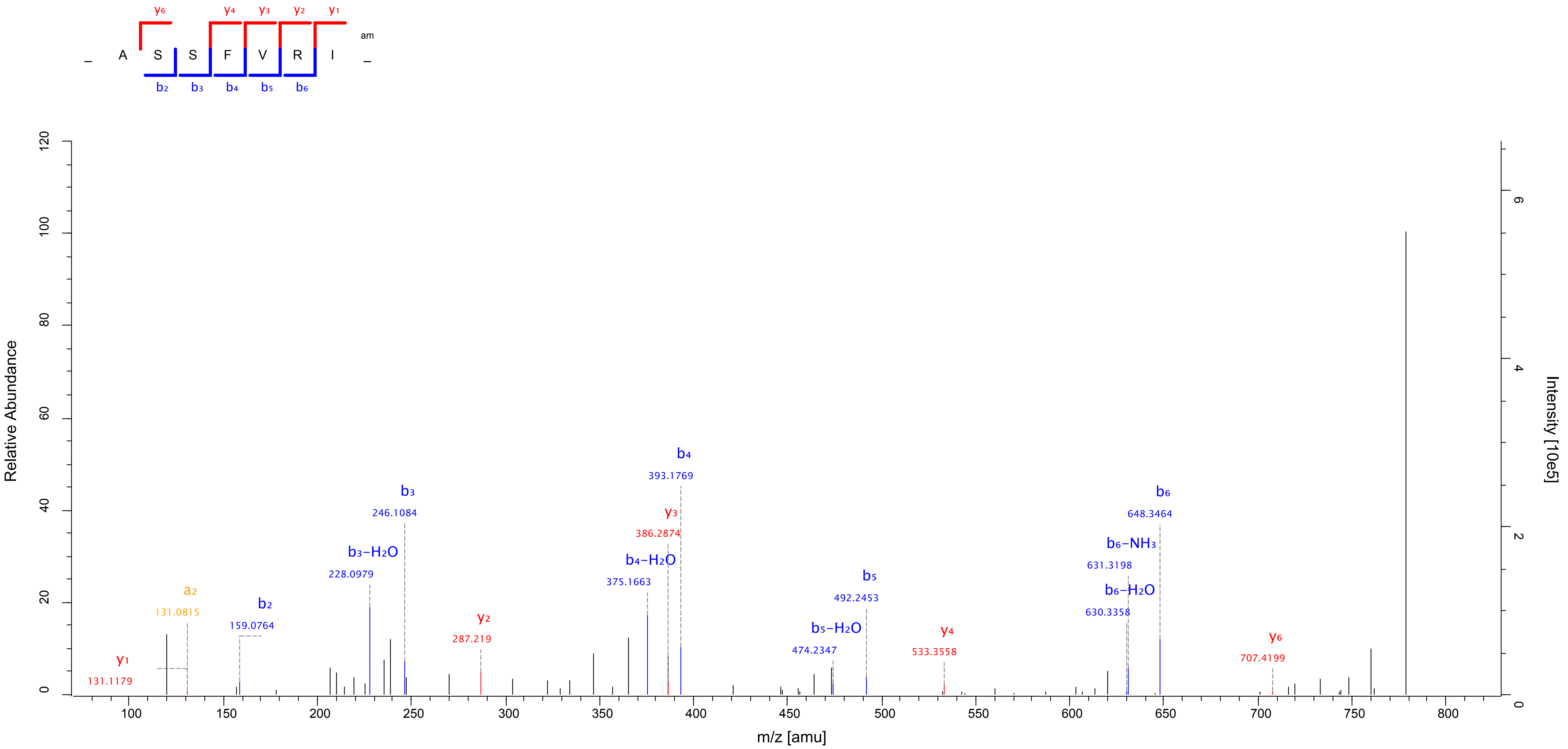

peptide ID 87

Source: 20120816_CO_0340GaJe_R02
Scannumber: 9958
Protein: pep_secretome_10386
Peptide Score: 85.55
Method: FTMS; HCD; 1

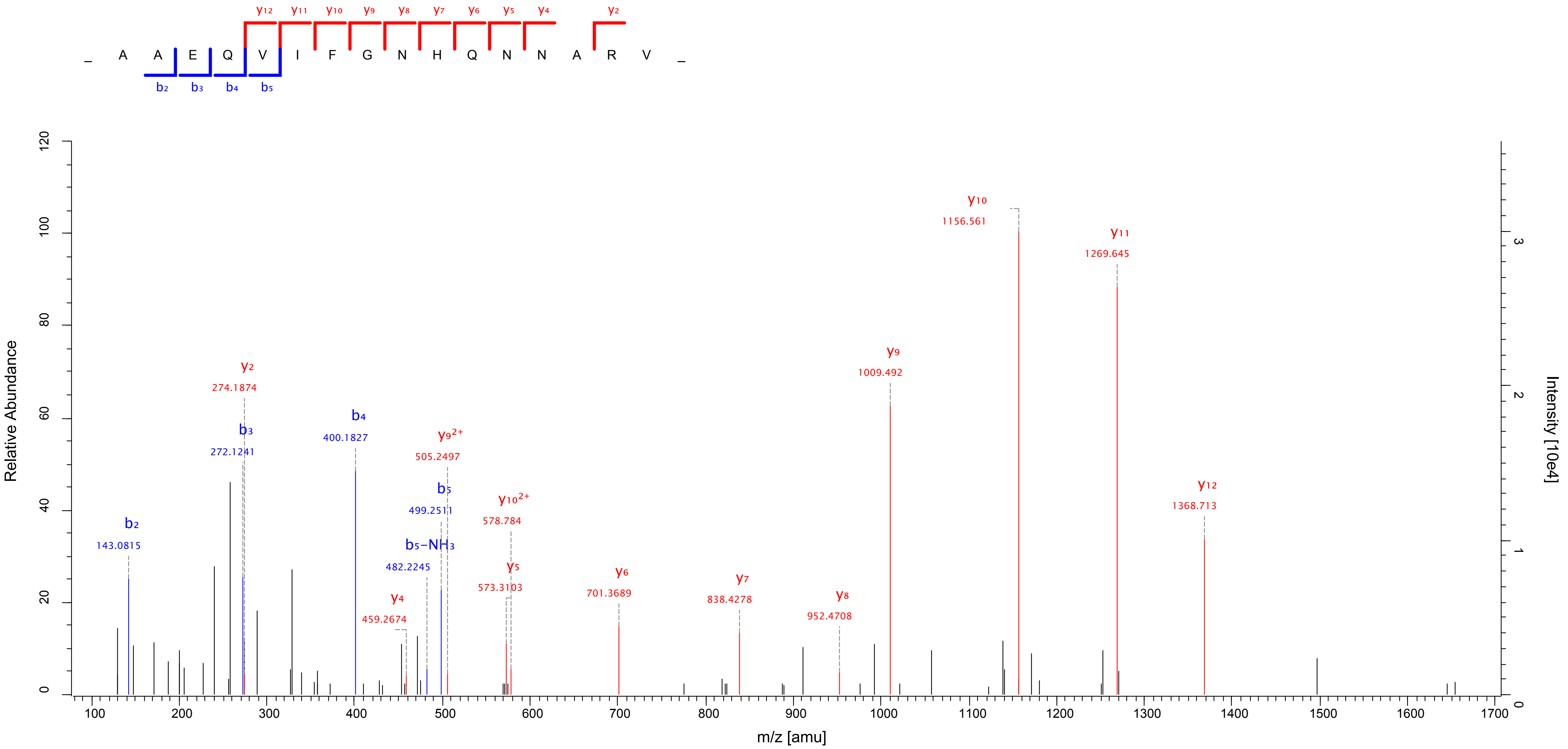

Source: 20121106_CO_0340GaJe_R02_2
Scannumber: 13762
Protein: pep_secretome_10395
Peptide Score: 73.91
Method: FTMS; HCD; 1

peptide ID 88

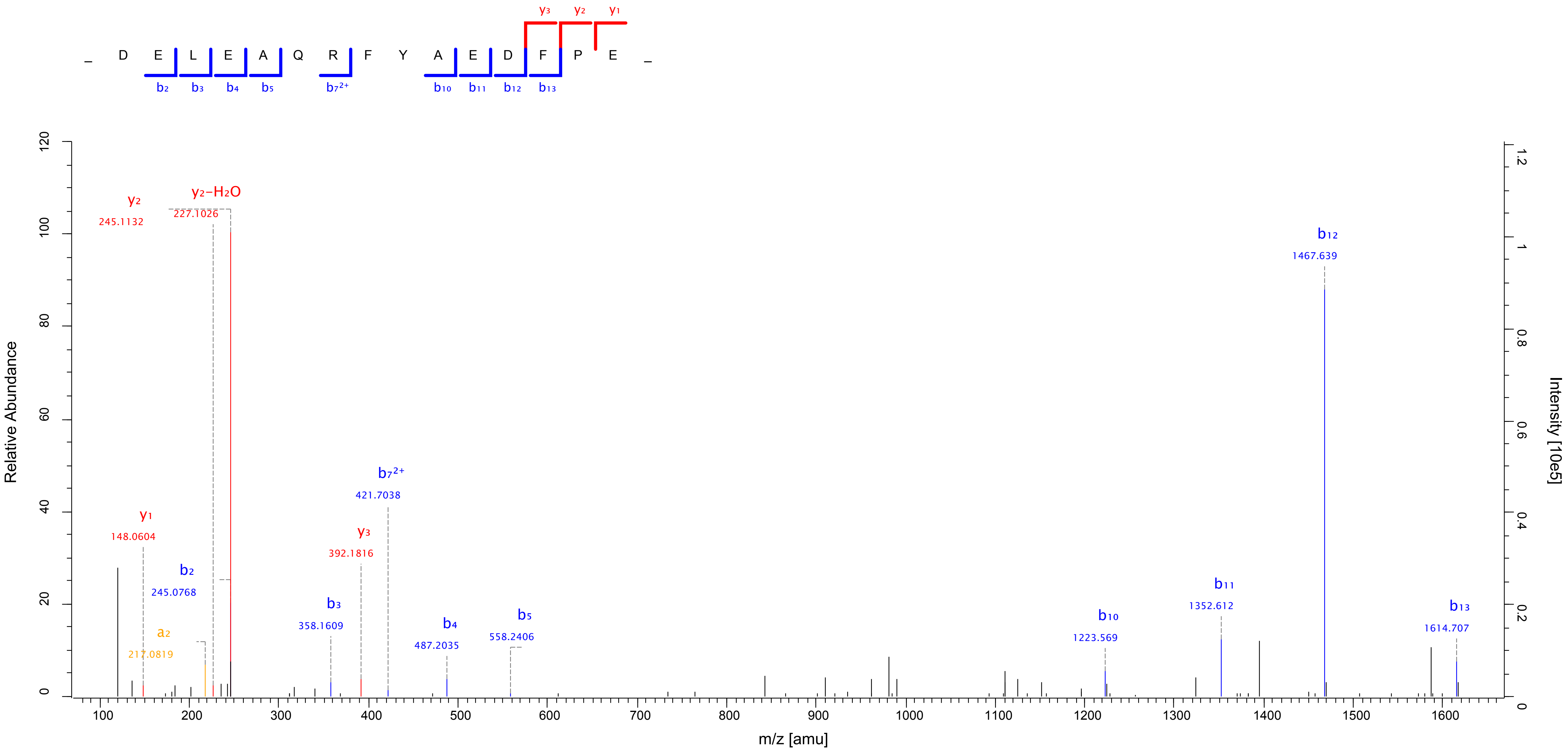

peptide ID 89

Source: 20120816_CO_0340GaJe_R02
Scannumber: 7409
Protein: pep_secretome_9793
Peptide Score: 63.73
Method: FTMS; HCD; 1

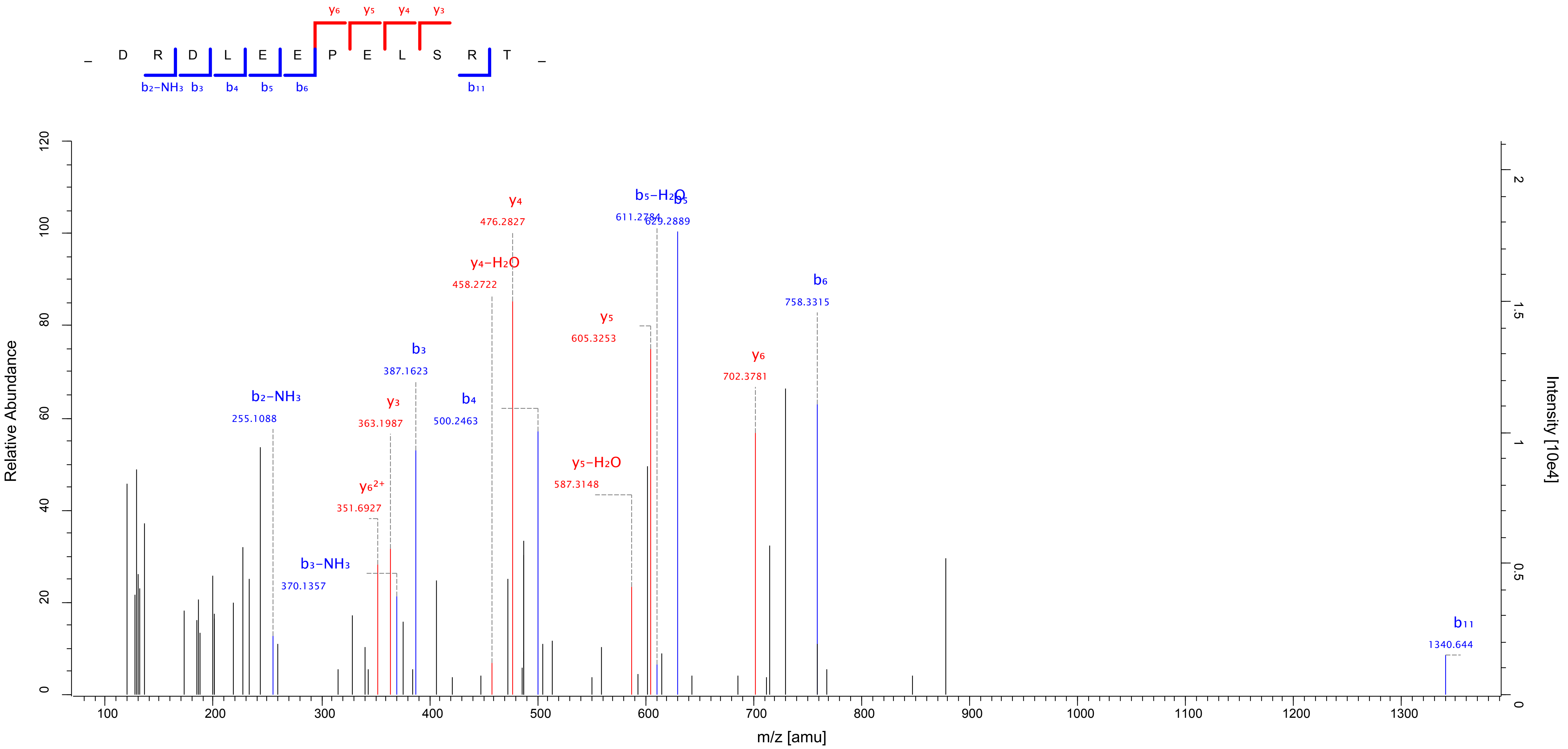

peptide ID 90

Source: 20120816_CO_0340GaJe_R02
Scannumber: 5729
Protein: pep_secretome_9794
Peptide Score: 95.57
Method: FTMS; HCD; 1

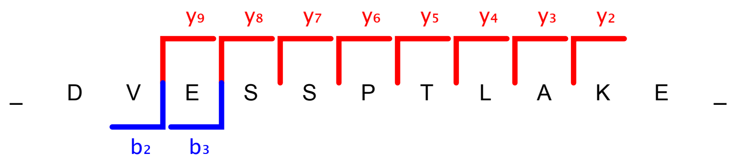

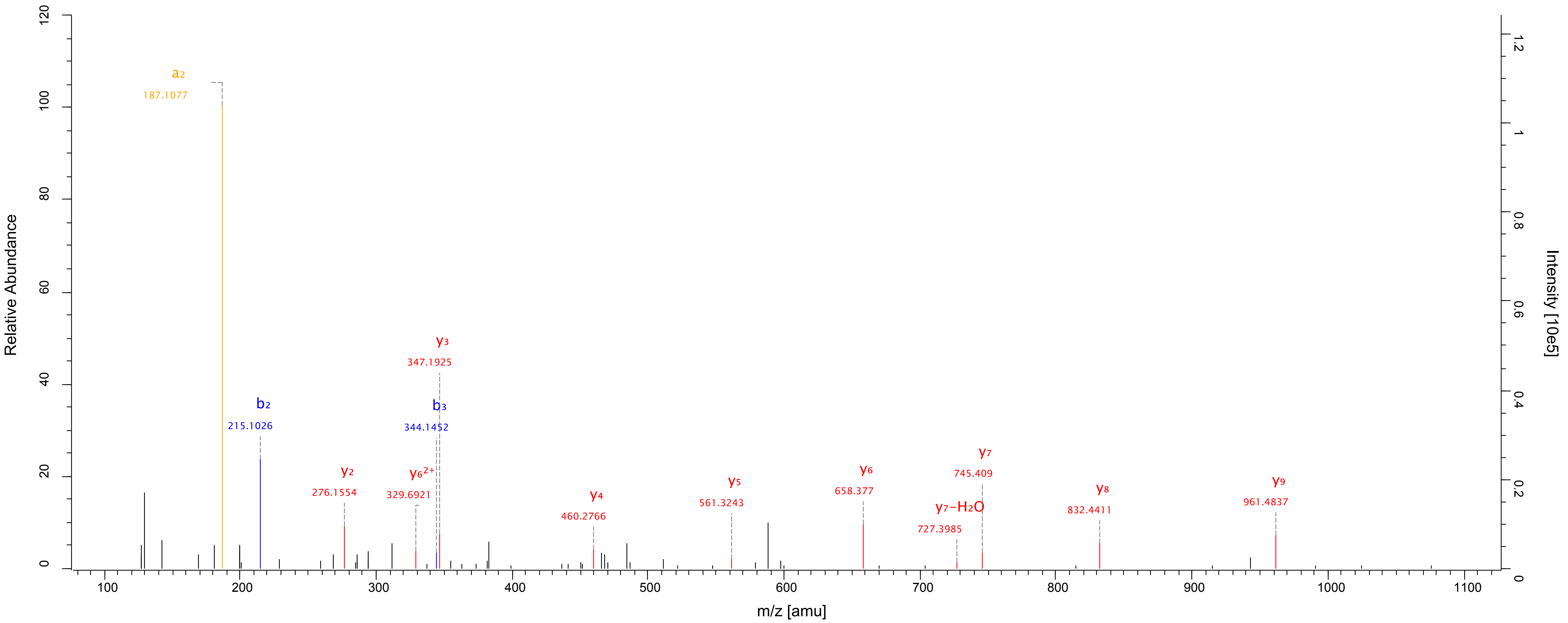

peptide ID 91

Source: 20121106_CO_0340GaJe_R02_2
Scannumber: 8478
Protein: pep_secretome_10230
Peptide Score: 120.66
Method: FTMS; HCD; 1

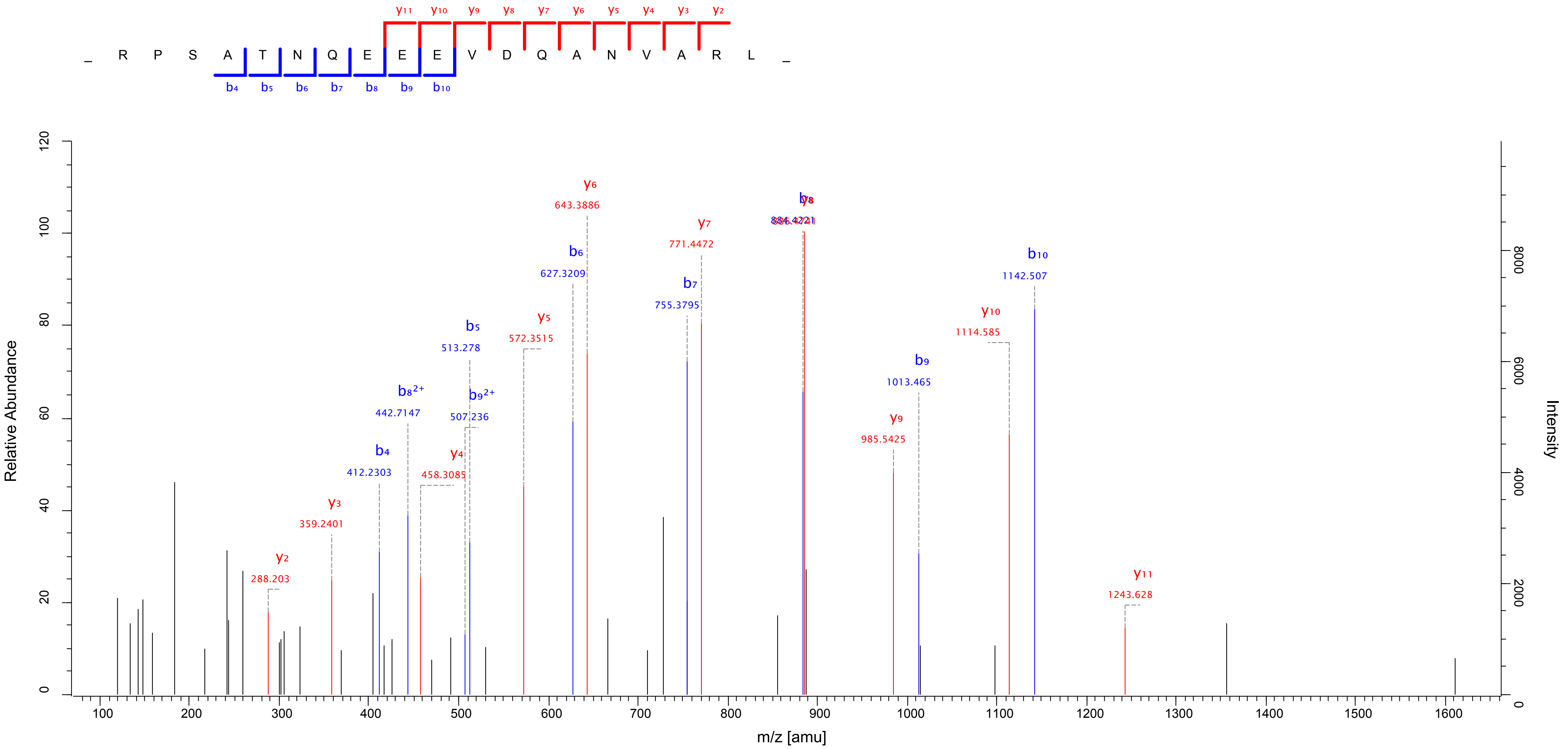

peptide ID 92

Source: 20120816_CO_0340GaJe_R02
Scannumber: 15103
Protein: pep_102; pep_secretome_264
Peptide Score: 77.12
Method: FTMS; HCD; 1

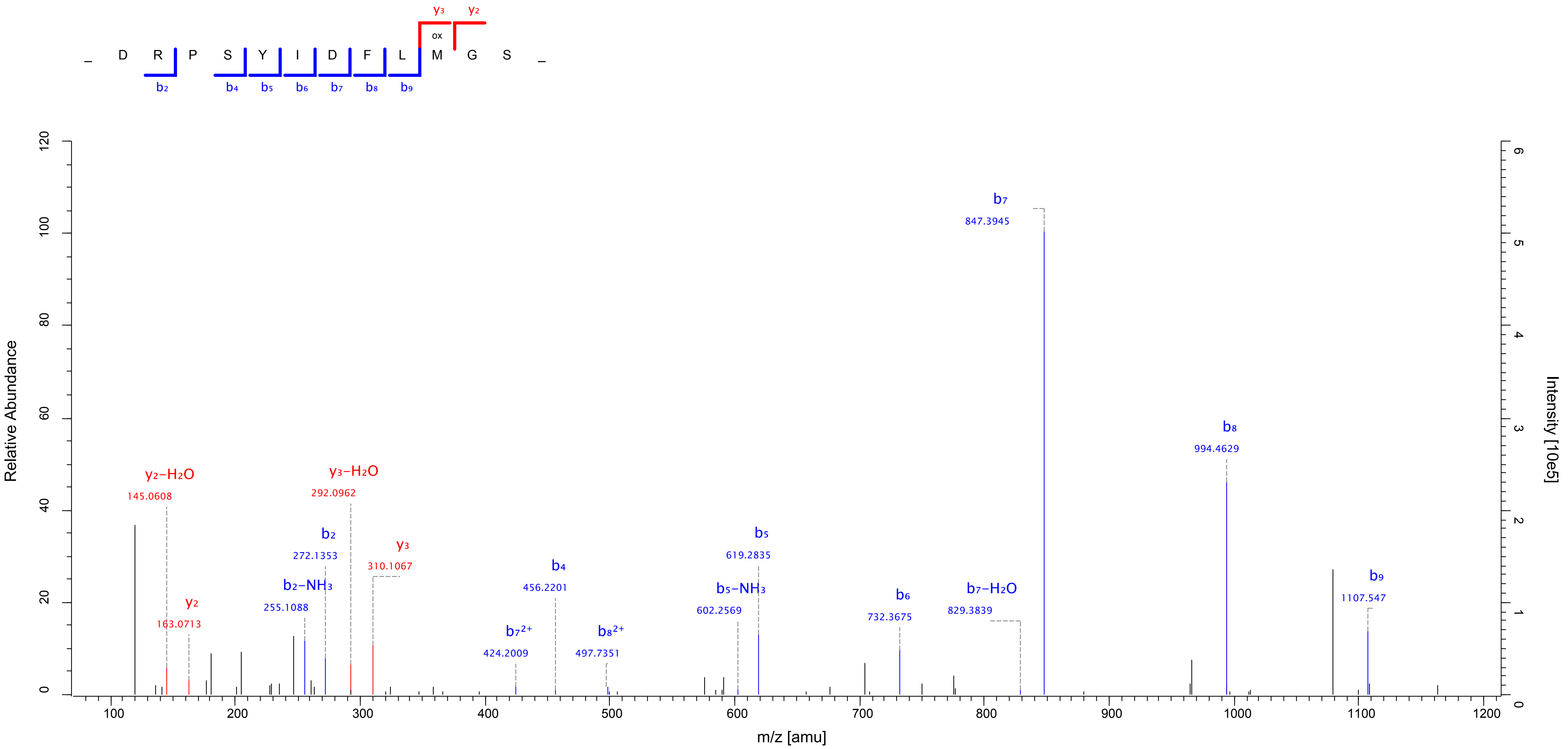

peptide ID 93

Source: 20120816_CO_0340GaJe_R02
Scannumber: 14833
Protein: pep_104; pep_secretome_268
Peptide Score: 77.64
Method: FTMS; HCD; 1

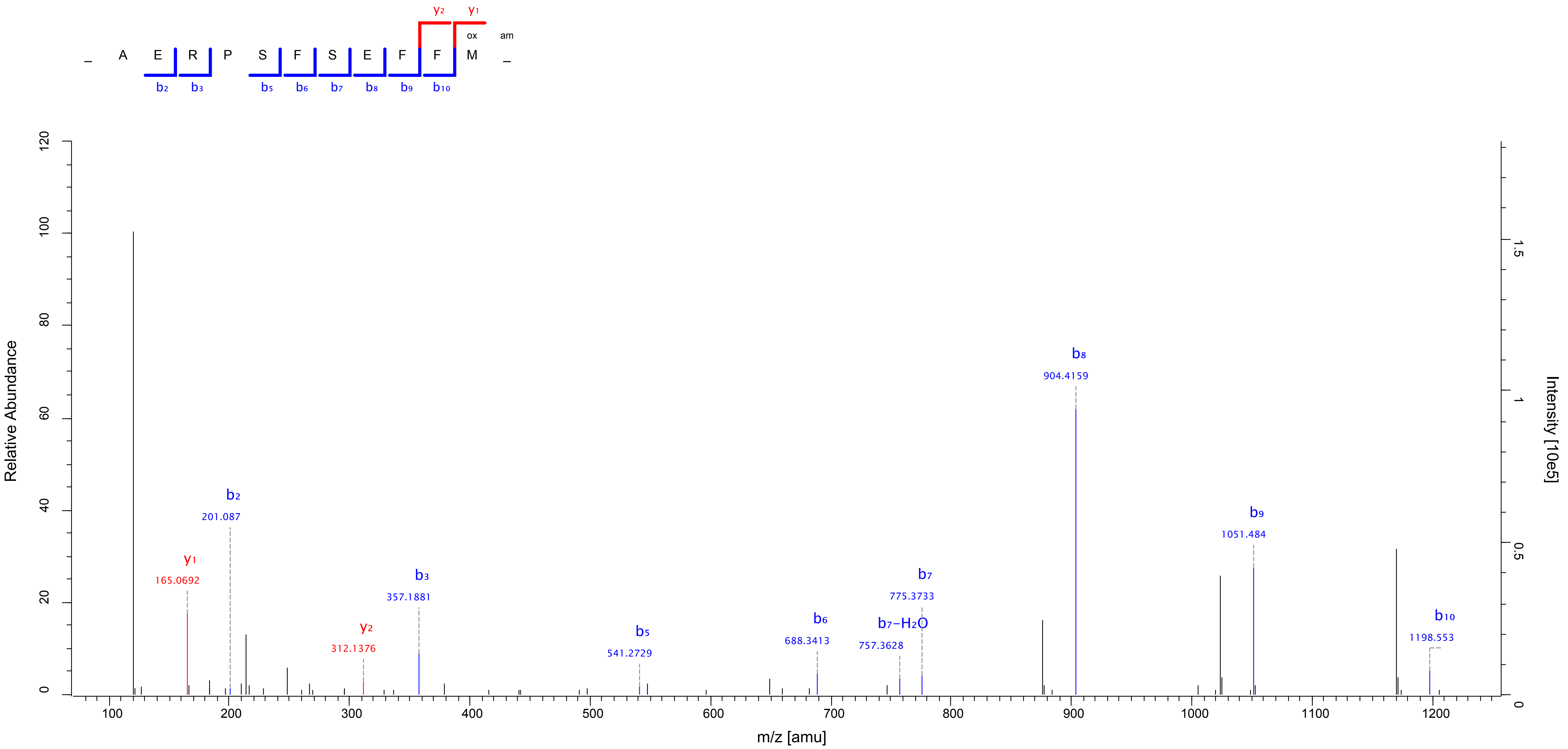

peptide ID 94

Source: 20121106_CO_0340GaJe_R02_2
Scannumber: 14942
Protein: pep_105; pep_secretome_22968; pep_secretome_270
Peptide Score: 88.09
Method: FTMS; HCD; 1

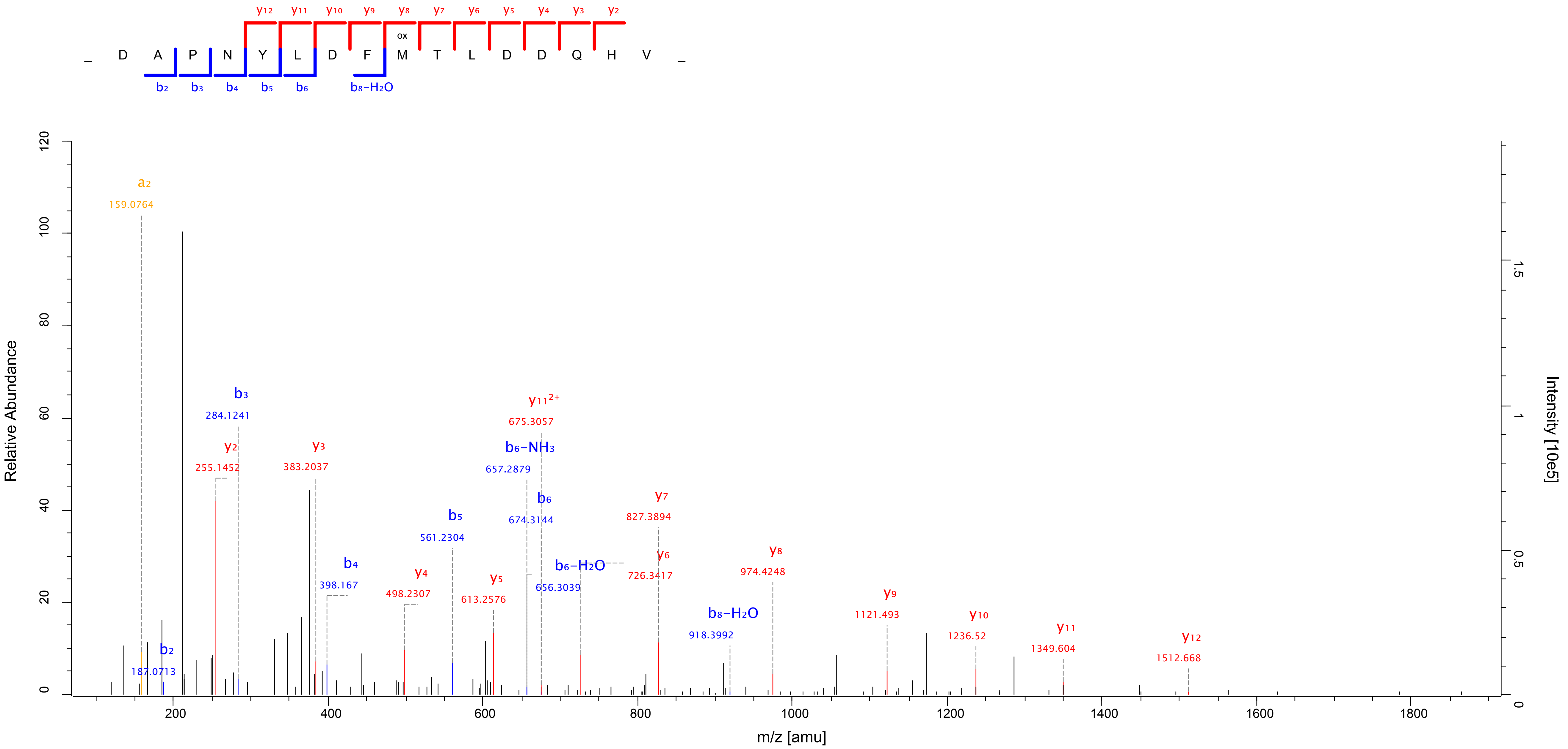

peptide ID 95

Source: 20121106_CO_0340GaJe_R02_2
Scannumber: 9715
Protein: pep_108; pep_secretome_274
Peptide Score: 84.29
Method: FTMS; HCD; 1

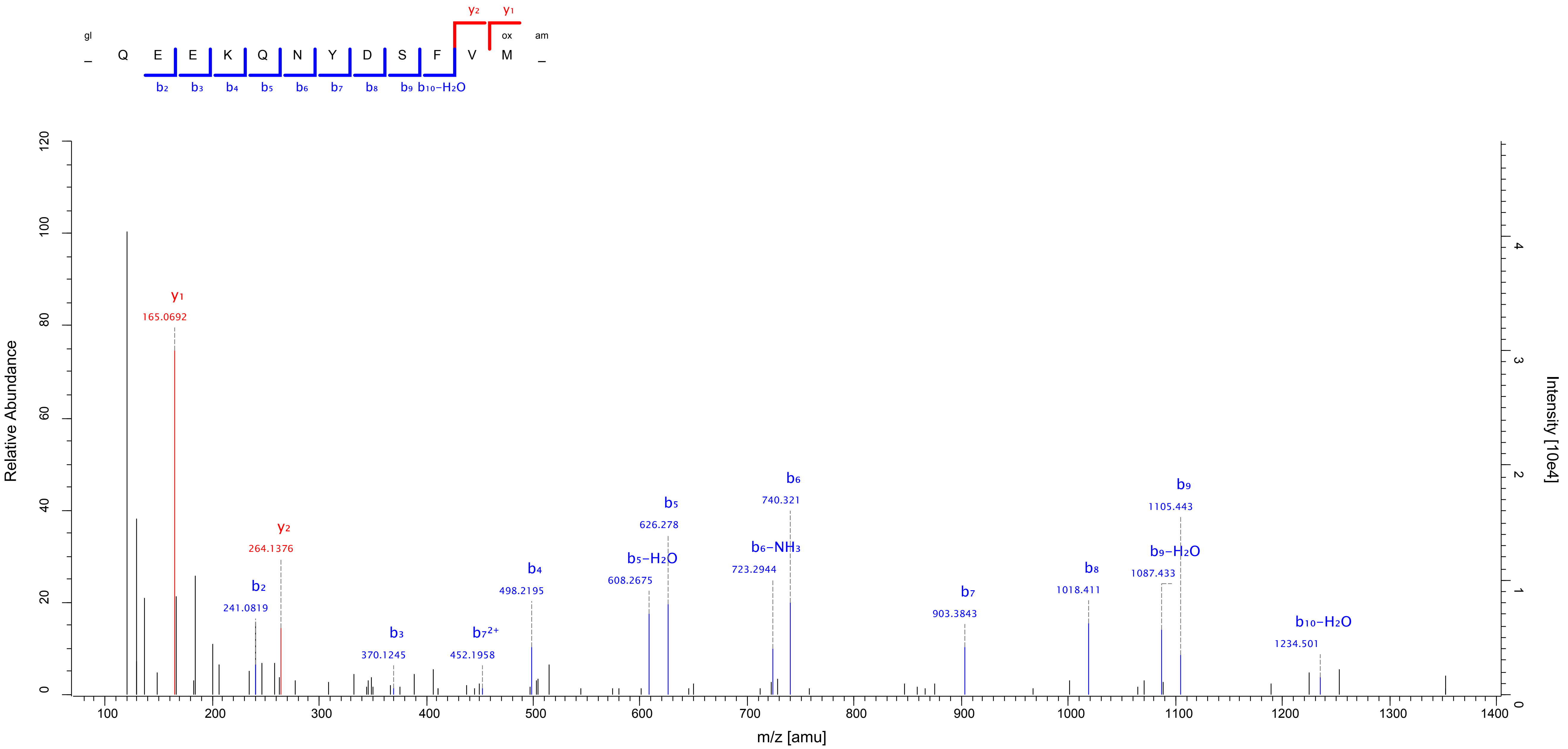

peptide ID 96

Source: 20120816_CO_0340GaJe_R02
Scannumber: 8137
Protein: pep_108; pep_secretome_274
Peptide Score: 62.47
Method: FTMS; HCD; 1

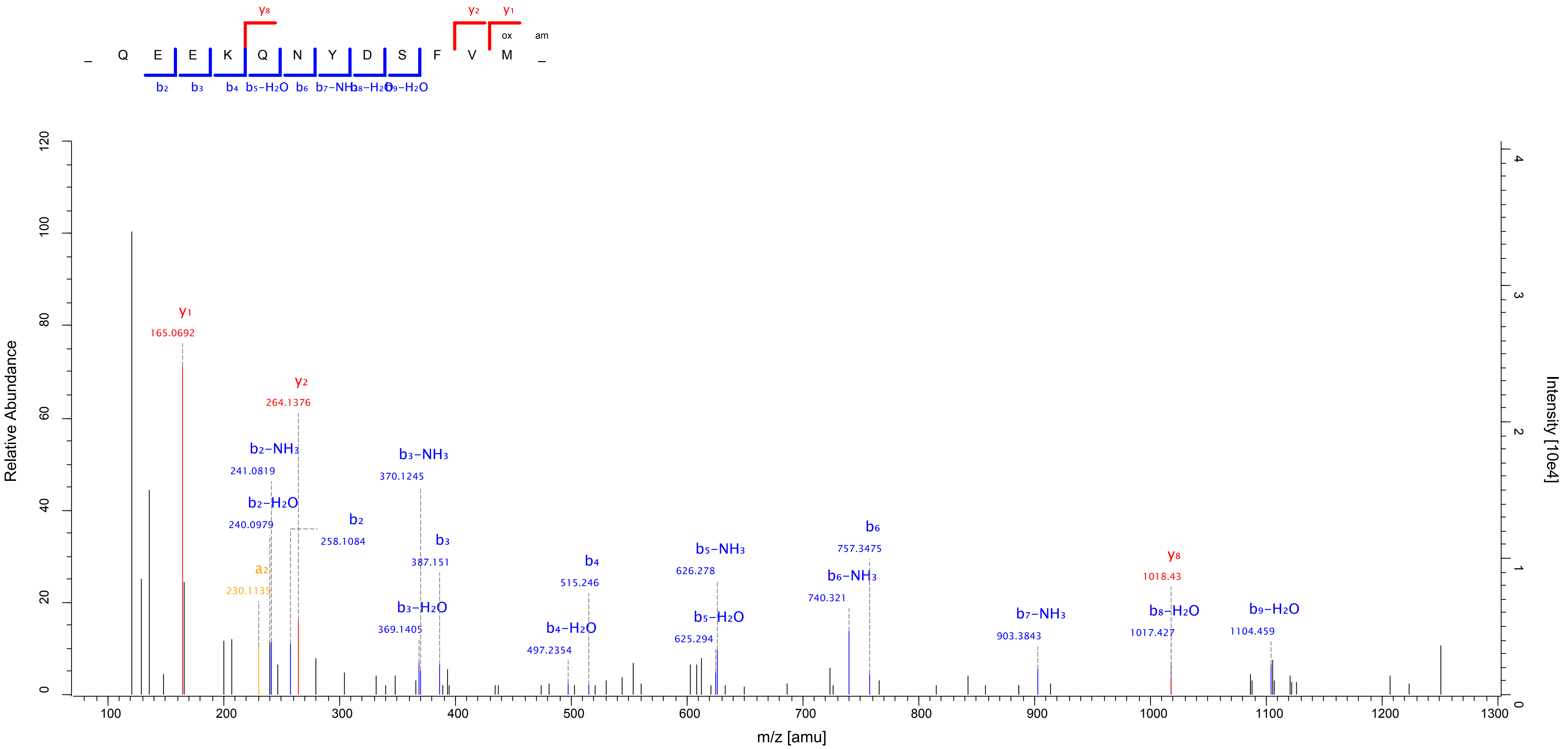

peptide ID 97

Source:	20120816_CO_0340GaJe_R02
Scannumber:	7890
Protein:	pep_secretome_276
Peptide Score:	103.7
Method:	FTMS; HCD; 1

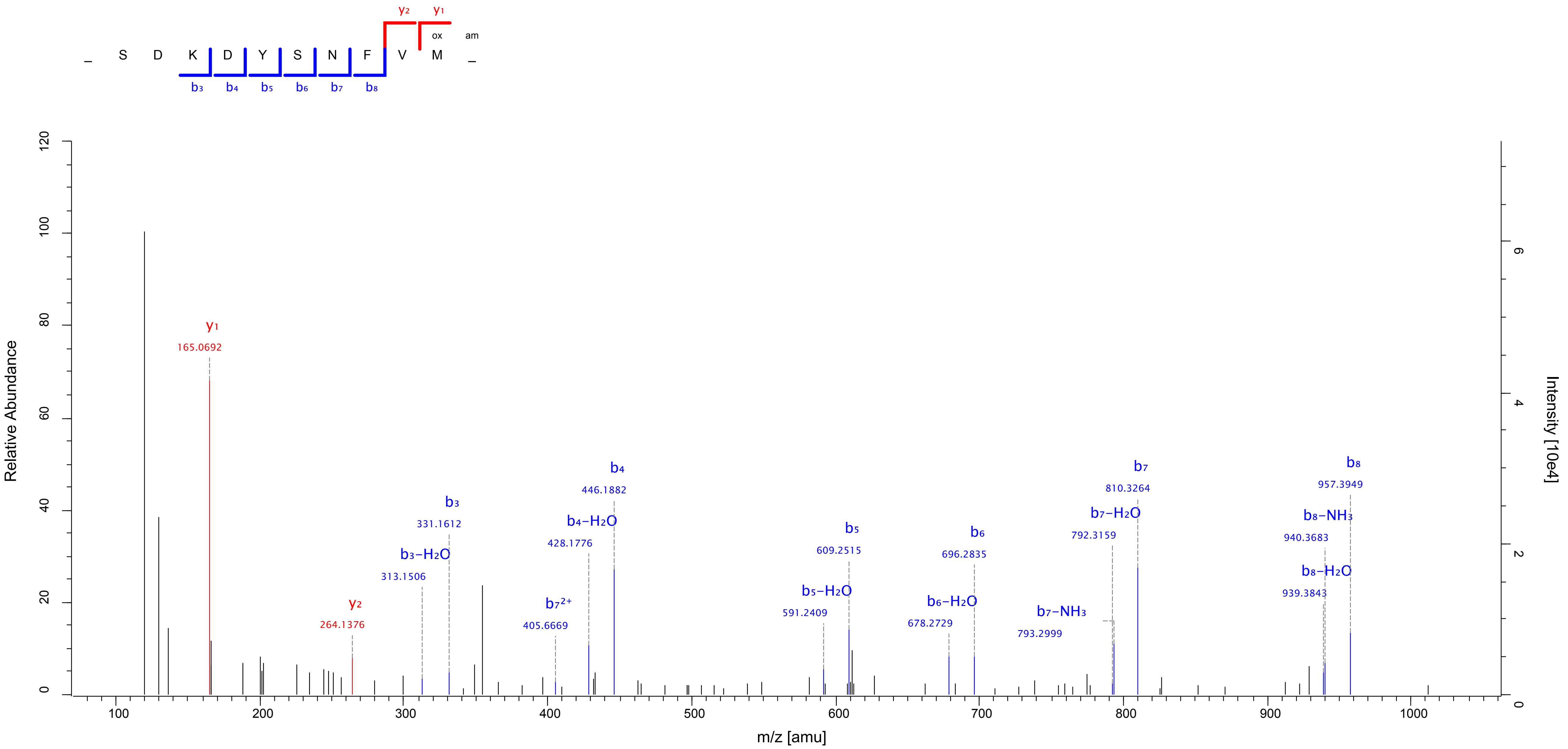

peptide ID 98

Source:	20120816_CO_0340GaJe_R02
Scannumber:	7903
Protein:	pep_secretome_279
Peptide Score:	111.61
Method:	FTMS; HCD; 1

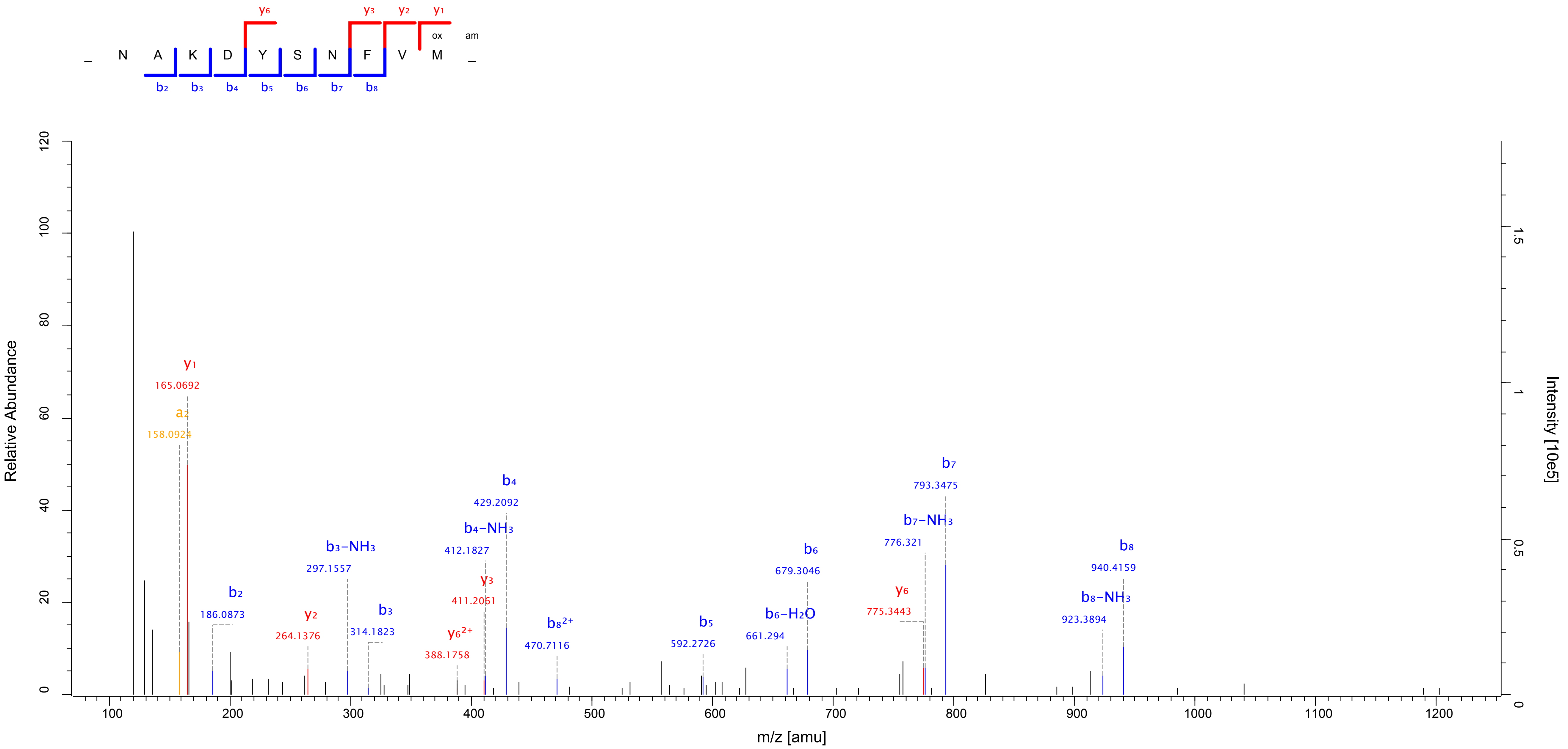

peptide ID 99

Source: 20120816_CO_0340GaJe_R02
Scannumber: 9127
Protein: pep_secretome_413; pep_secretome_414; pep_secretome_419
Peptide Score: 119.17
Method: FTMS; HCD; 1

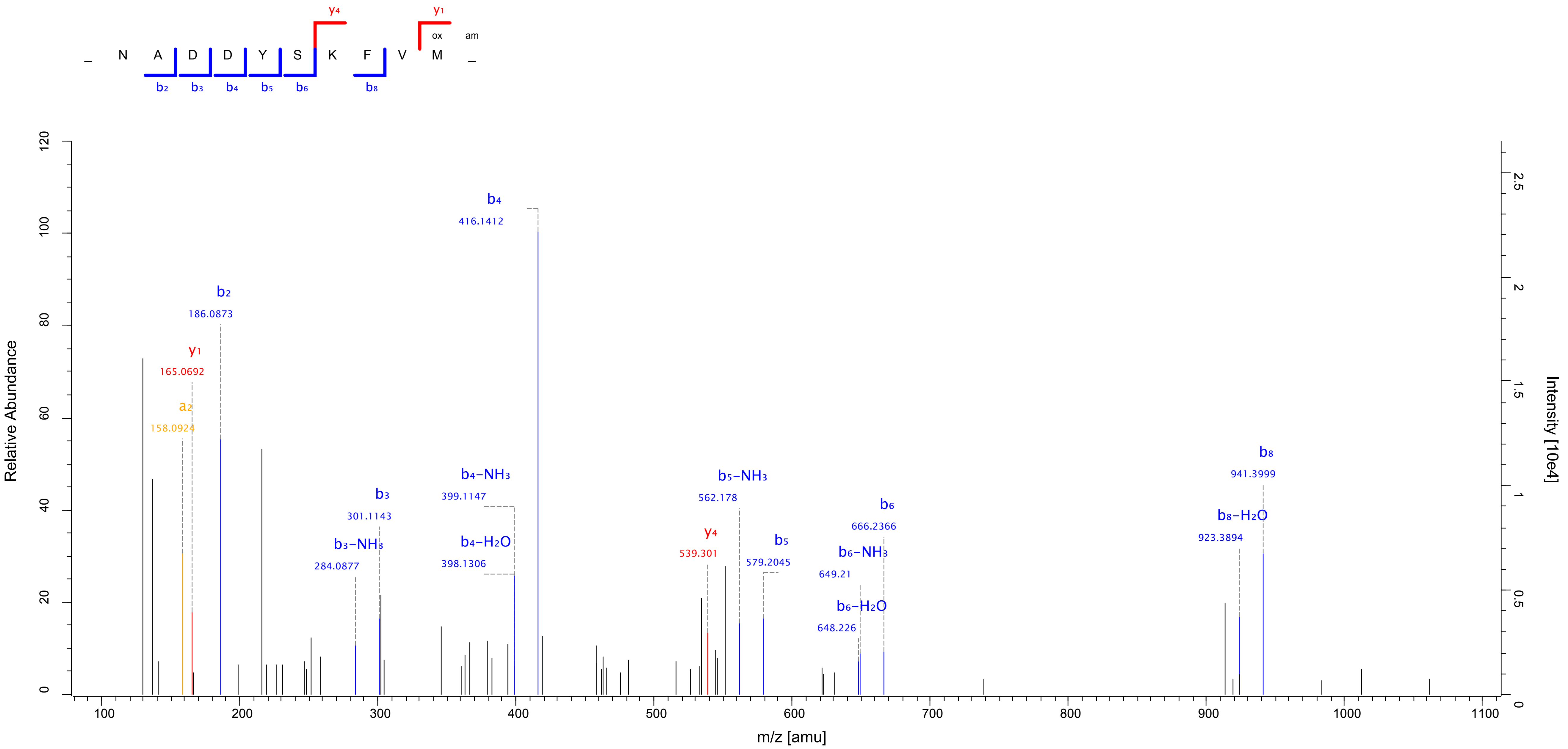

peptide ID 100

Source:	20121106_CO_0340GaJe_R02_2
Scannumber:	10162
Protein:	pep_secretome_281; pep_secretome_282; pep_secretome_415; pep_secretome_418; pep_secretome_421; pep_secretome_424; pep_secretome_427; pep_secretome_428
Peptide Score:	131.45
Method:	FTMS; HCD; 1

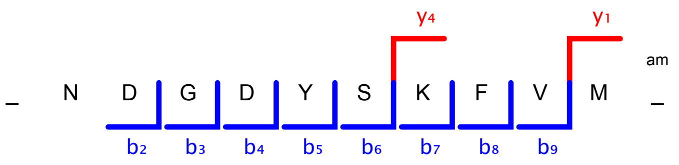

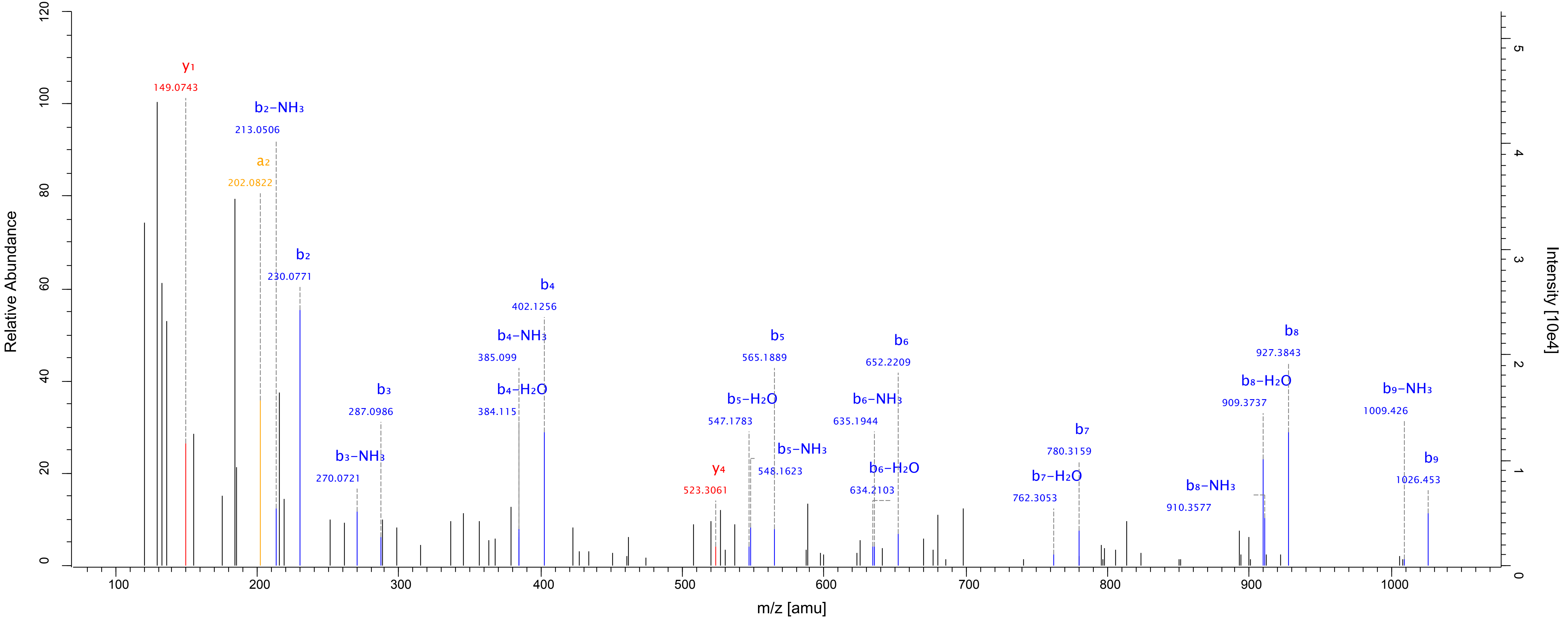

peptide ID 101

Source: 20120816_CO_0340GaJe_R02
Scannumber: 8400
Protein: pep_secretome_281; pep_secretome_282; pep_secretome_415; pep_secretome_418; pep_secretome_421; pep_secretome_424; pep_secretome_427; pep_secretome_428
Peptide Score: 111.74
Method: FTMS; HCD; 1

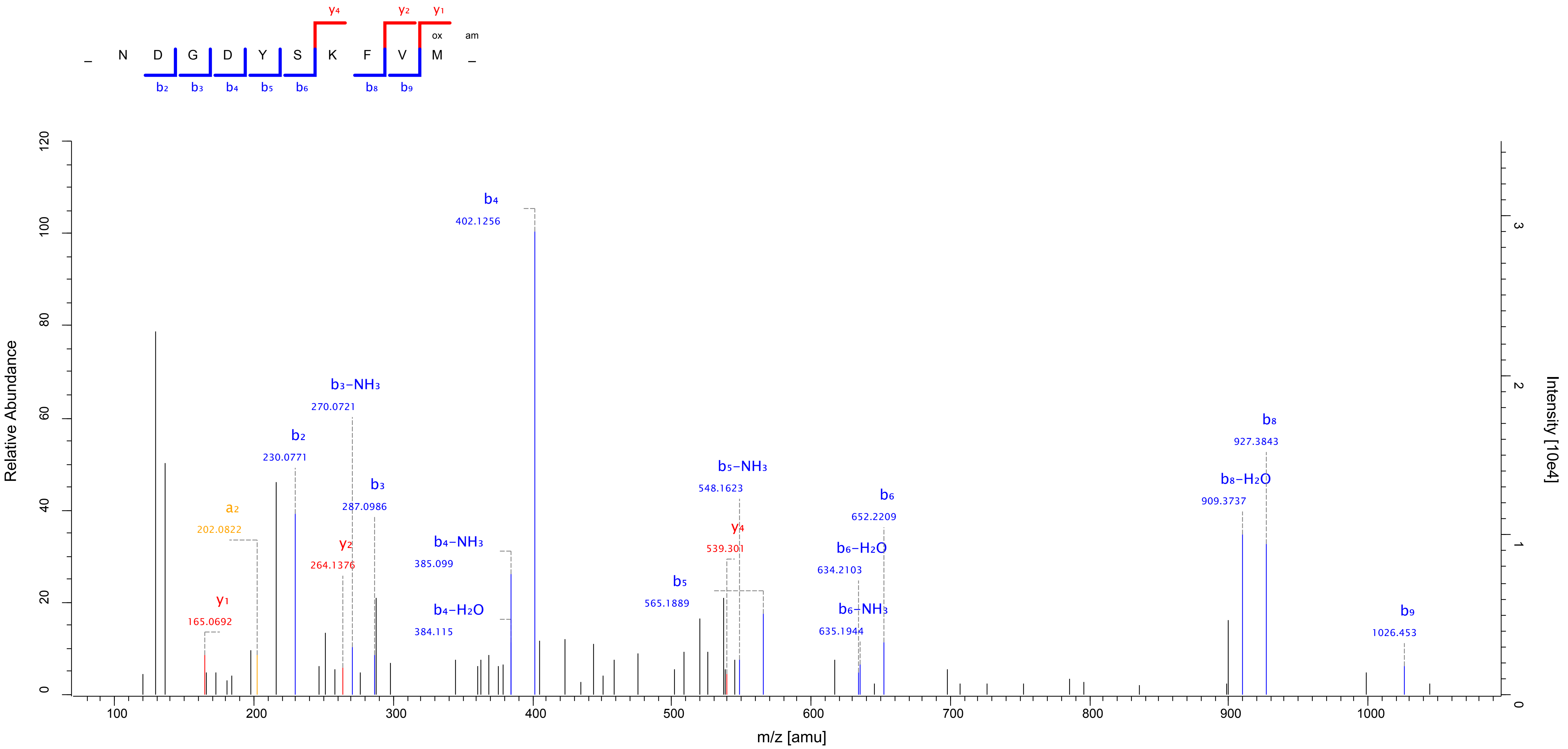

peptide ID 102

Source: 20121106_CO_0340GaJe_R02_2
Scannumber: 8224
Protein: pep_secretome_285; pep_secretome_425
Peptide Score: 138.08
Method: FTMS; HCD; 1

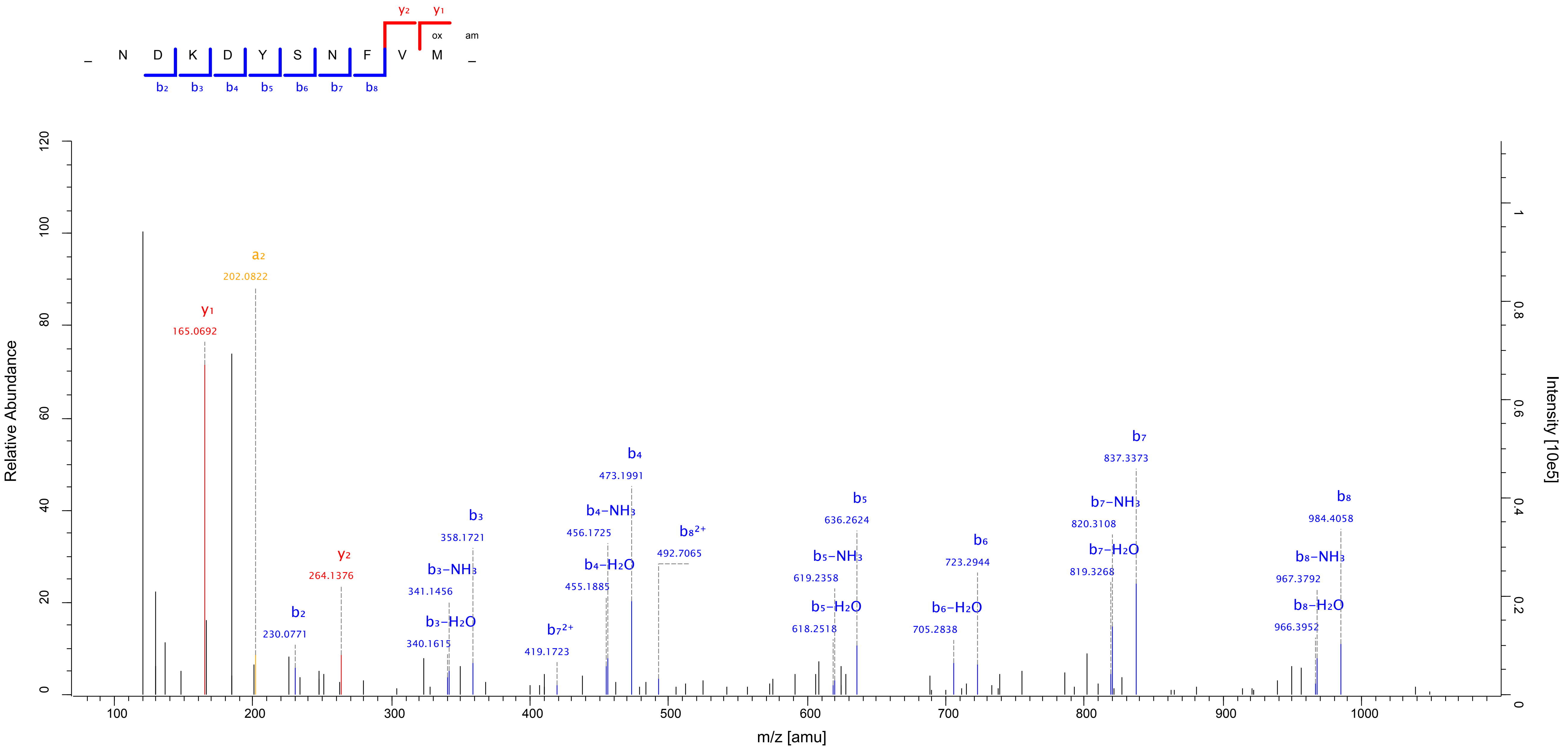

Source: 20120816_CO_0340GaJe_R02
Scannumber: 9316
Protein: pep_163; pep_secretome_23298; pep_secretome_432
Peptide Score: 79.63
Method: FTMS; HCD; 1

peptide ID 103

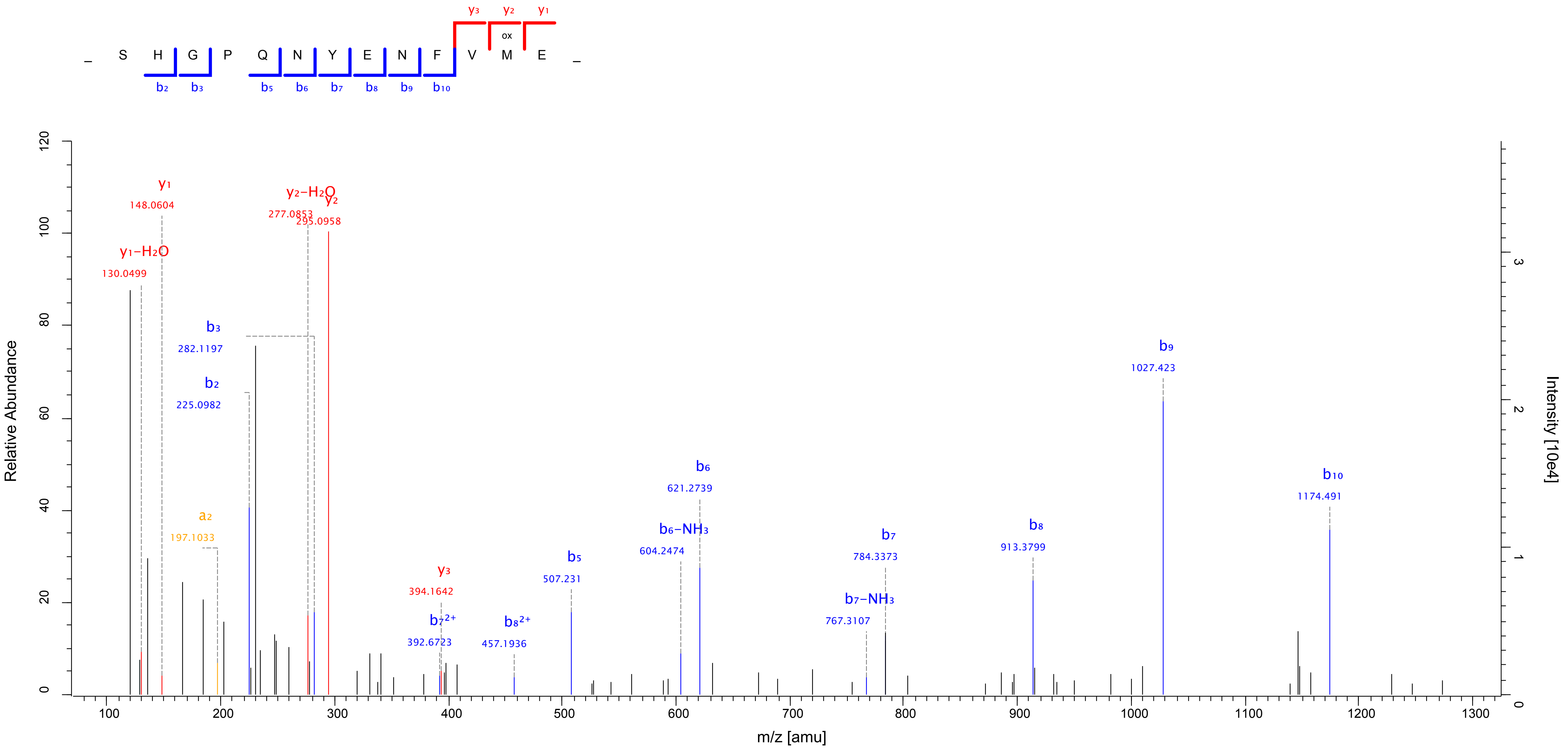

peptide ID 104

Source:	20120816_CO_0340GaJe_R02
Scannumber:	7664
Protein:	pep_secretome_434
Peptide Score:	139.28
Method:	FTMS; HCD; 1

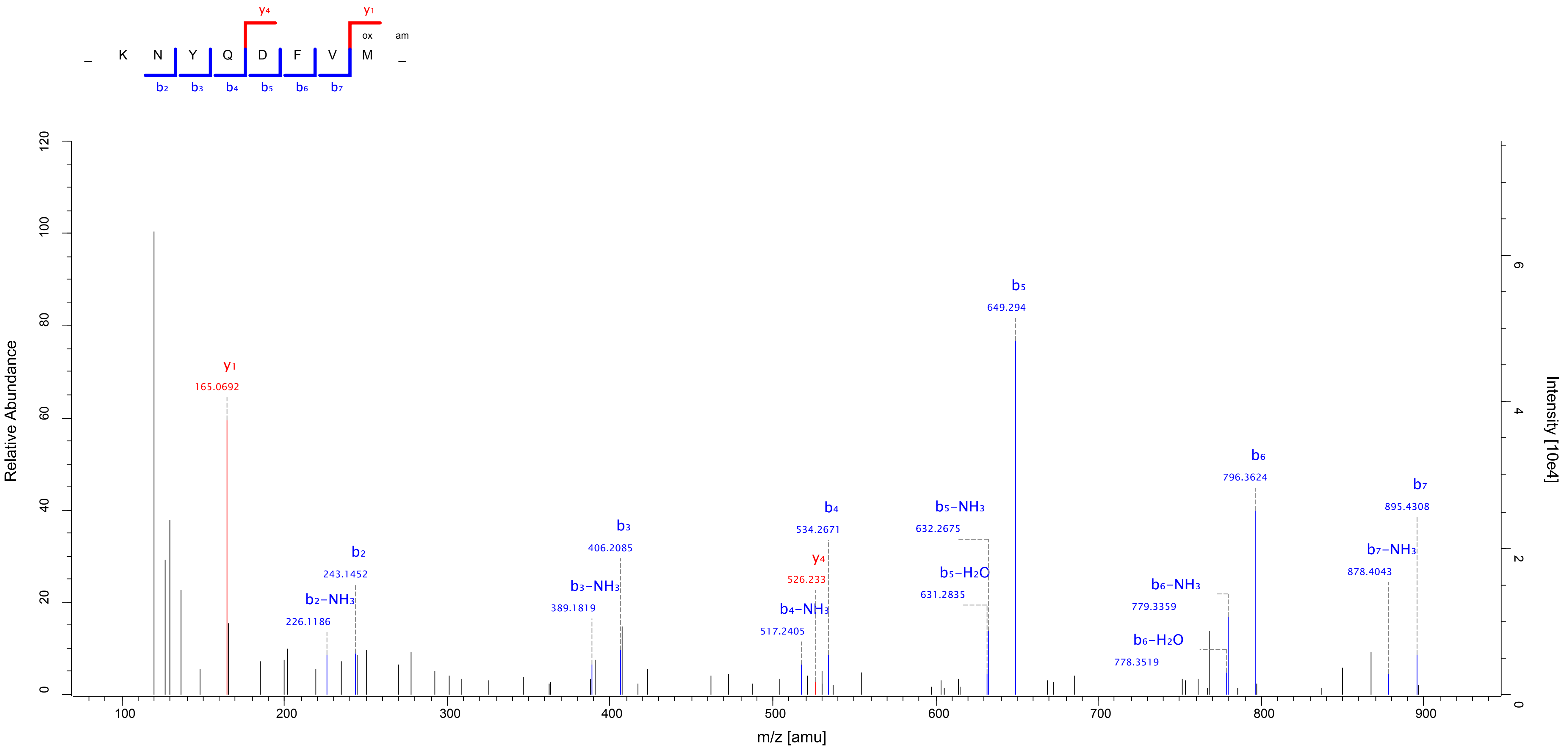

peptide ID 105

Source:	20121106_CO_0340GaJe_R02_2
Scannumber:	7932
Protein:	pep_secretome_391; pep_secretome_394
Peptide Score:	148.28
Method:	FTMS; HCD; 1

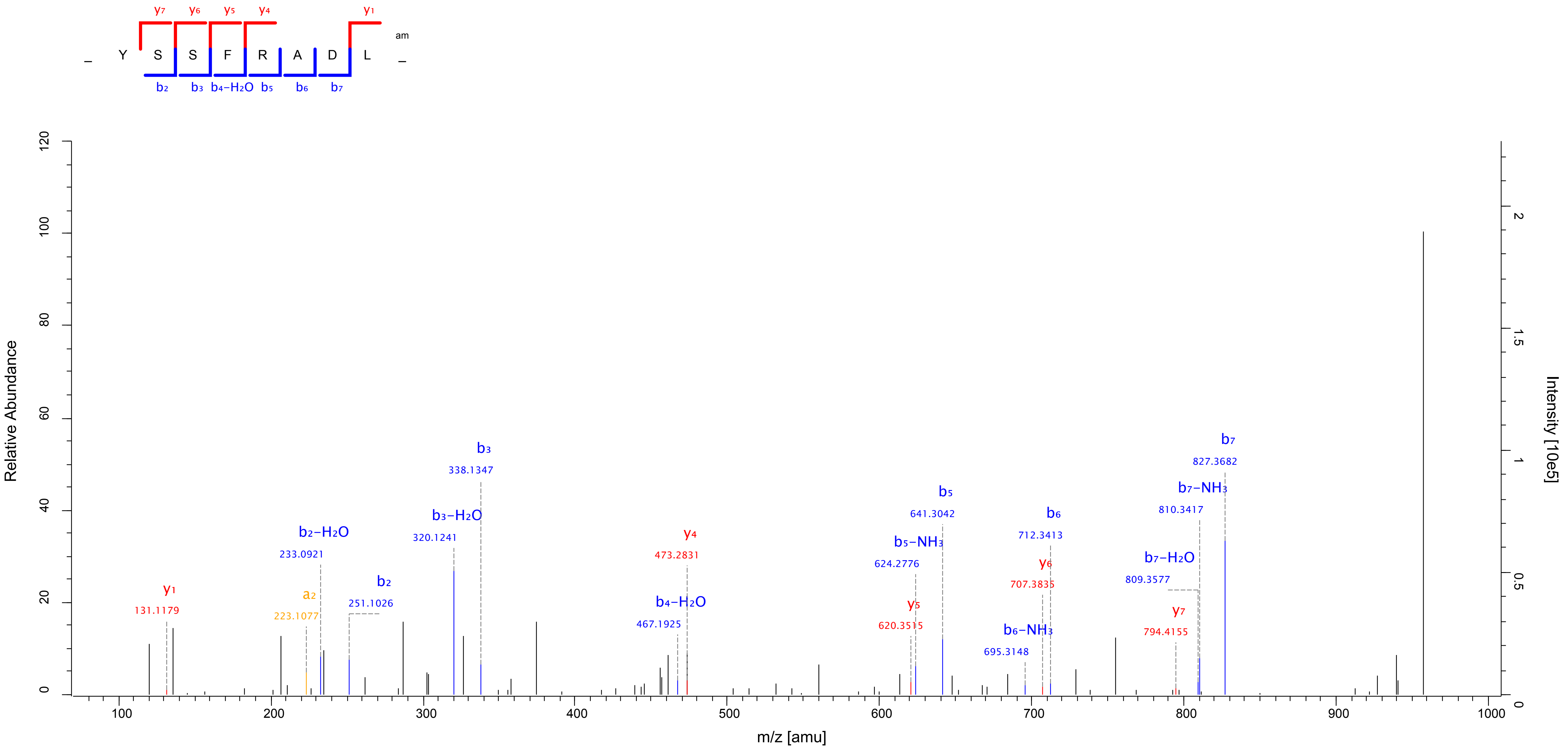

peptide ID 106

Source:	20121106_CO_0340GaJe_R02_2
Scannumber:	13674
Protein:	orf_39097
Peptide Score:	94.77
Method:	FTMS; HCD; 1

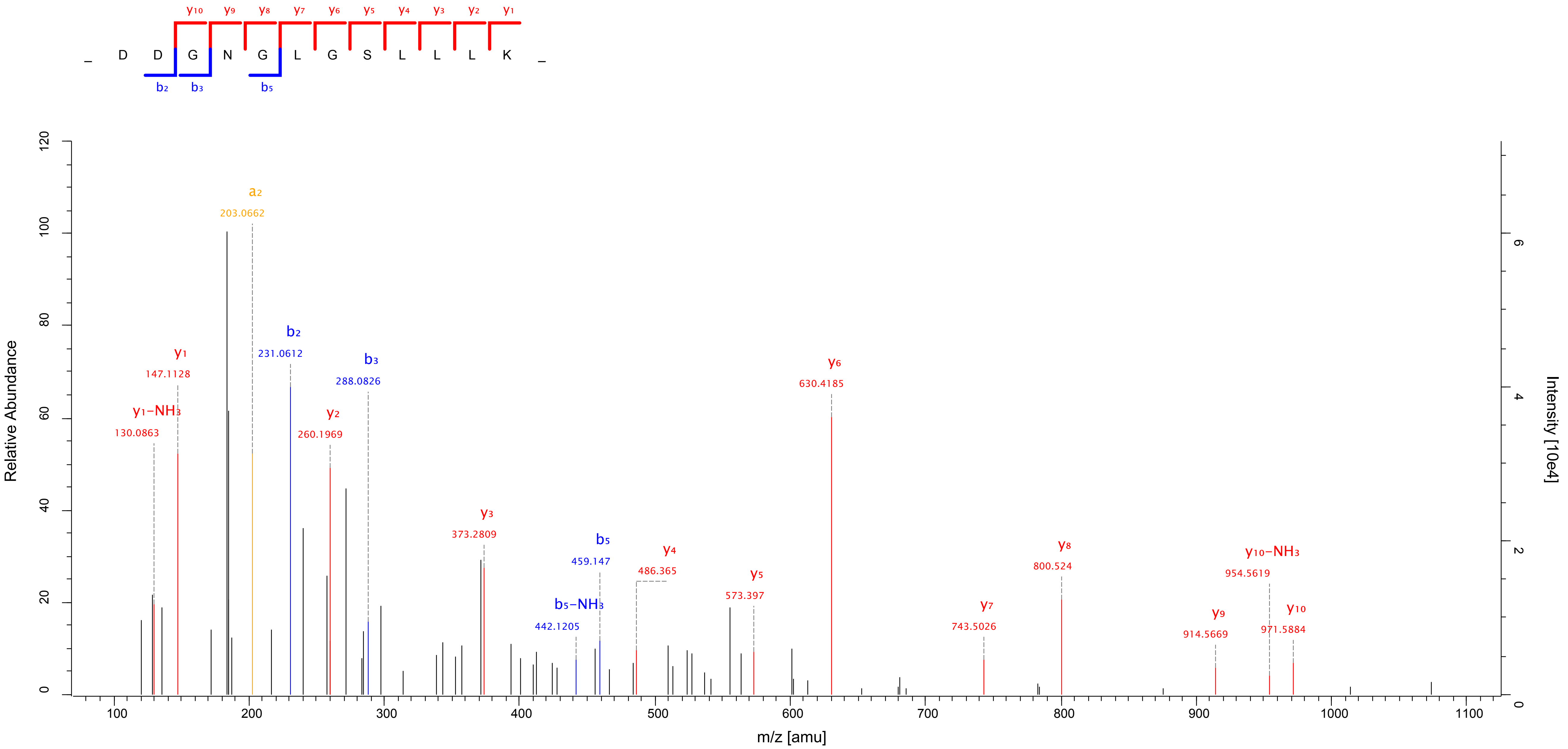

peptide ID 107

Source: 20120515_CO_0340GaJe_R01
Scannumber: 5633
Protein: pep_secretome_11312
Peptide Score: 225.85
Method: FTMS; HCD; 1

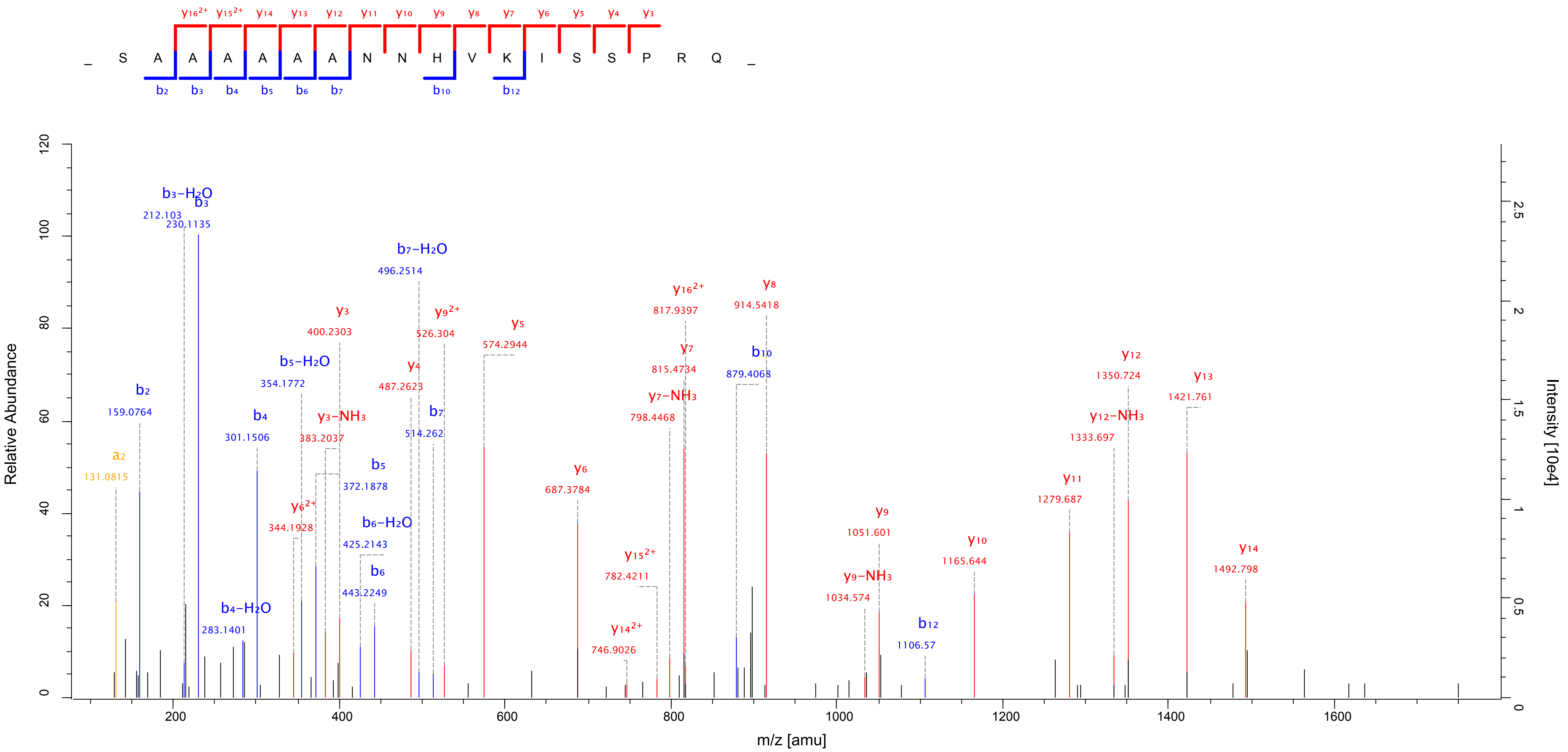

peptide ID 108

Source:	20120816_CO_0340GaJe_R02
Scannumber:	7103
Protein:	pep_secretome_8203
Peptide Score:	128.57
Method:	FTMS; HCD; 1

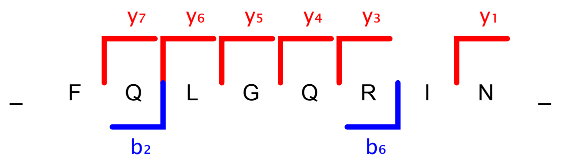

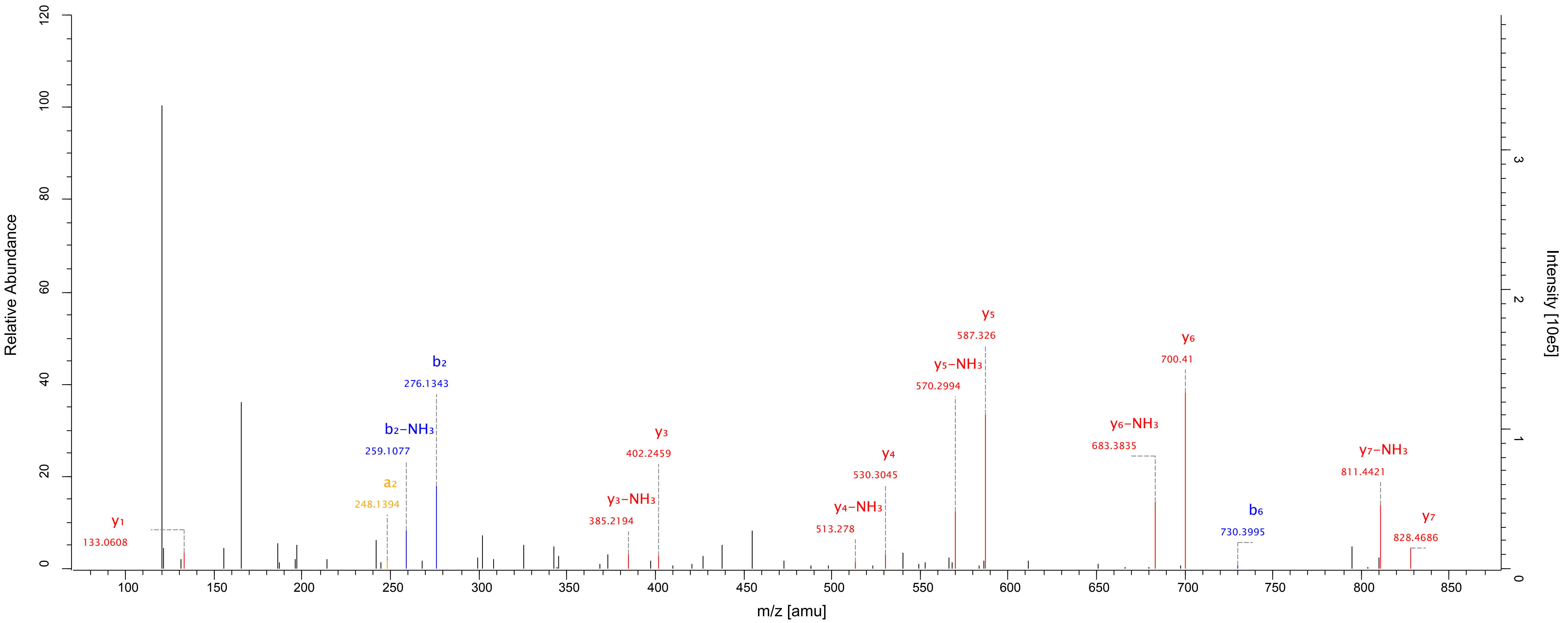

peptide ID 109

Source: 20121106_CO_0340GaJe_R02_2
Scannumber: 13572
Protein: pep_secretome_15484
Peptide Score: 84.29
Method: FTMS; HCD; 1

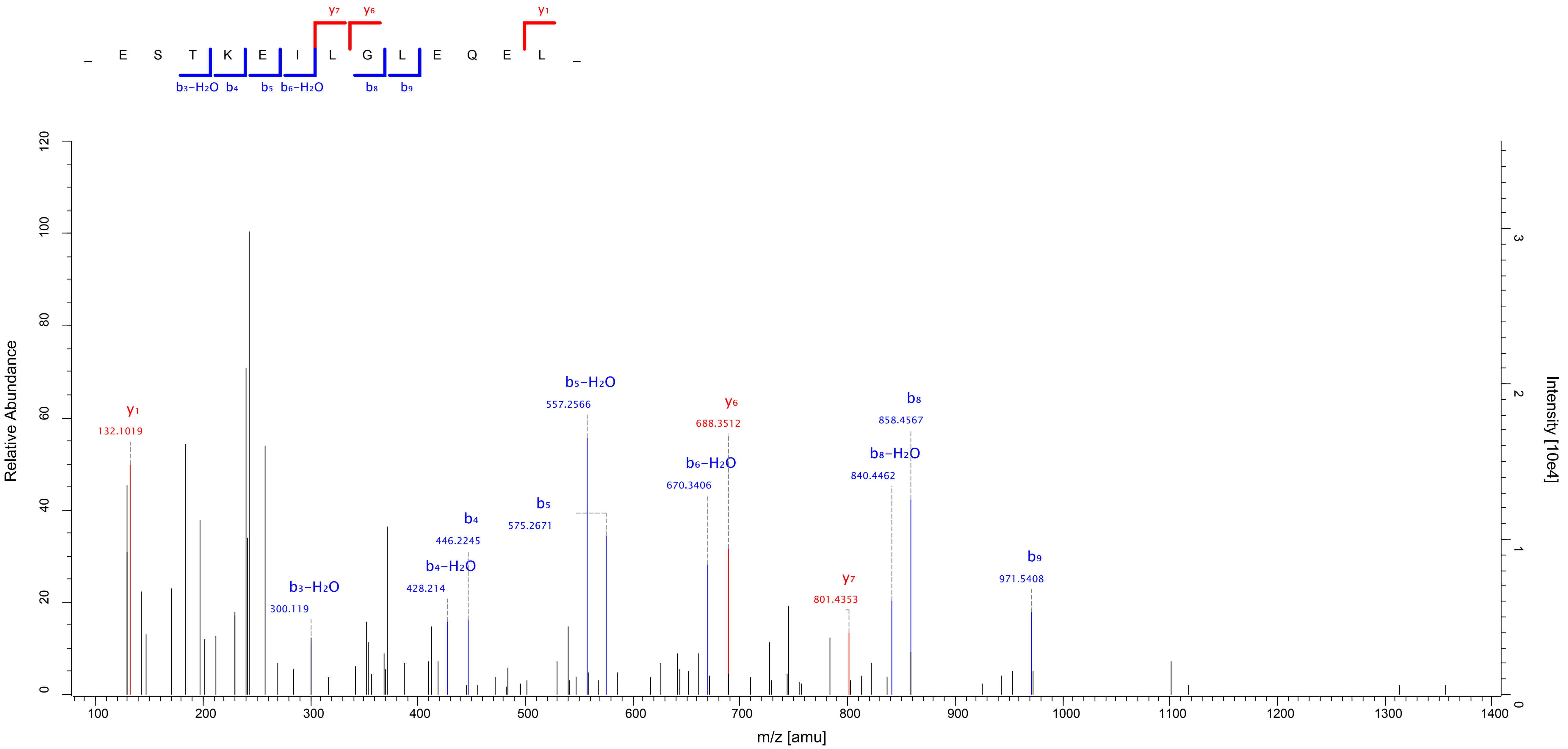

Source: 20120816_CO_0340GaJe_R02
Scannumber: 9758
Protein: pep_219; pep_secretome_557; pep_secretome_563; pep_secretome_566
Peptide Score: 61.76
Method: FTMS; HCD; 1

peptide ID 110

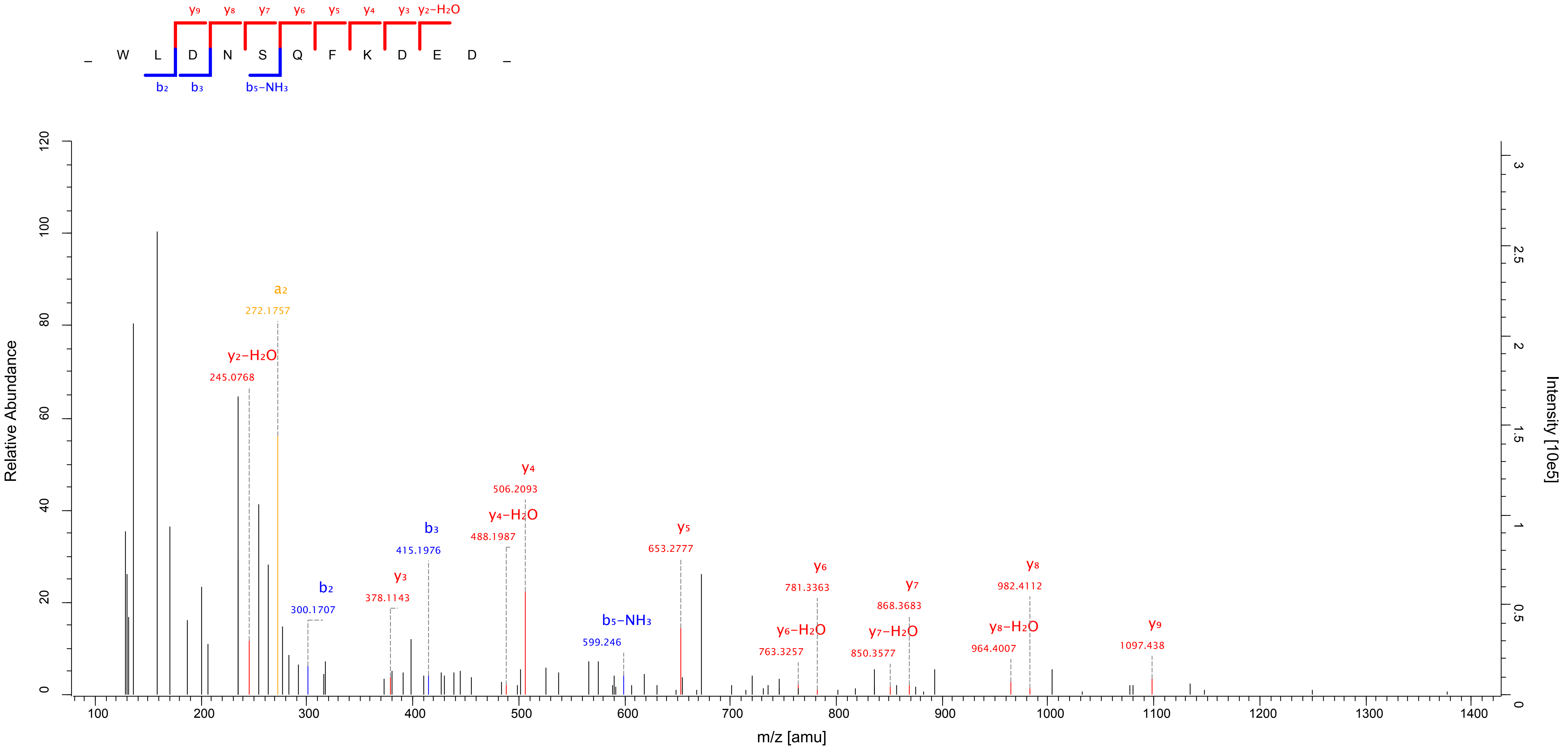

Source:	20120816_CO_0340GaJe_R02
Scannumber:	10099
Protein:	pep_secretome_558; pep_secretome_565
Peptide Score:	80.75
Method:	FTMS; HCD; 1

peptide ID 111

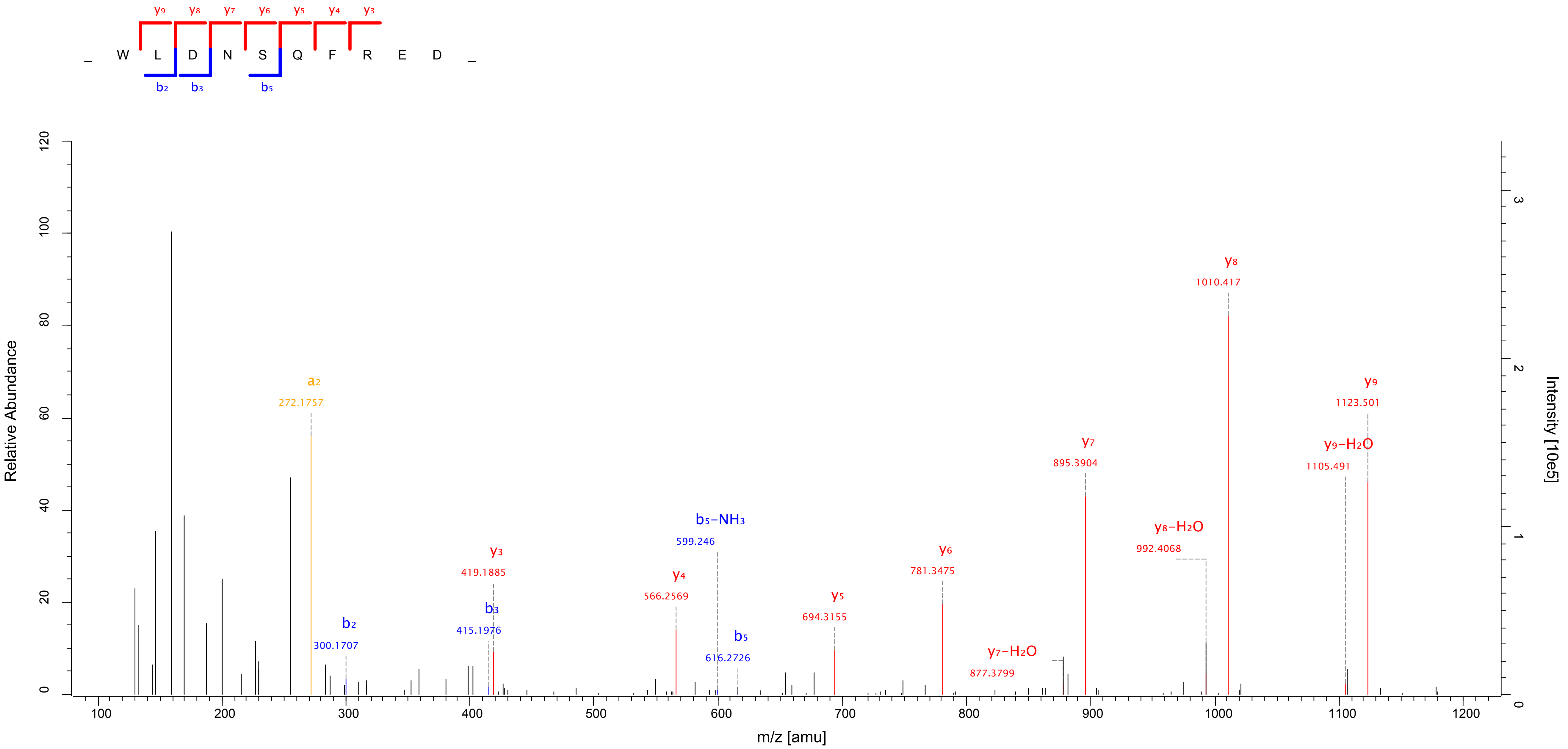

peptide ID 112

Source: 20120816_CO_0340GaJe_R02
Scannumber: 8943
Protein: pep_secretome_1287
Peptide Score: 102.66
Method: FTMS; HCD; 1

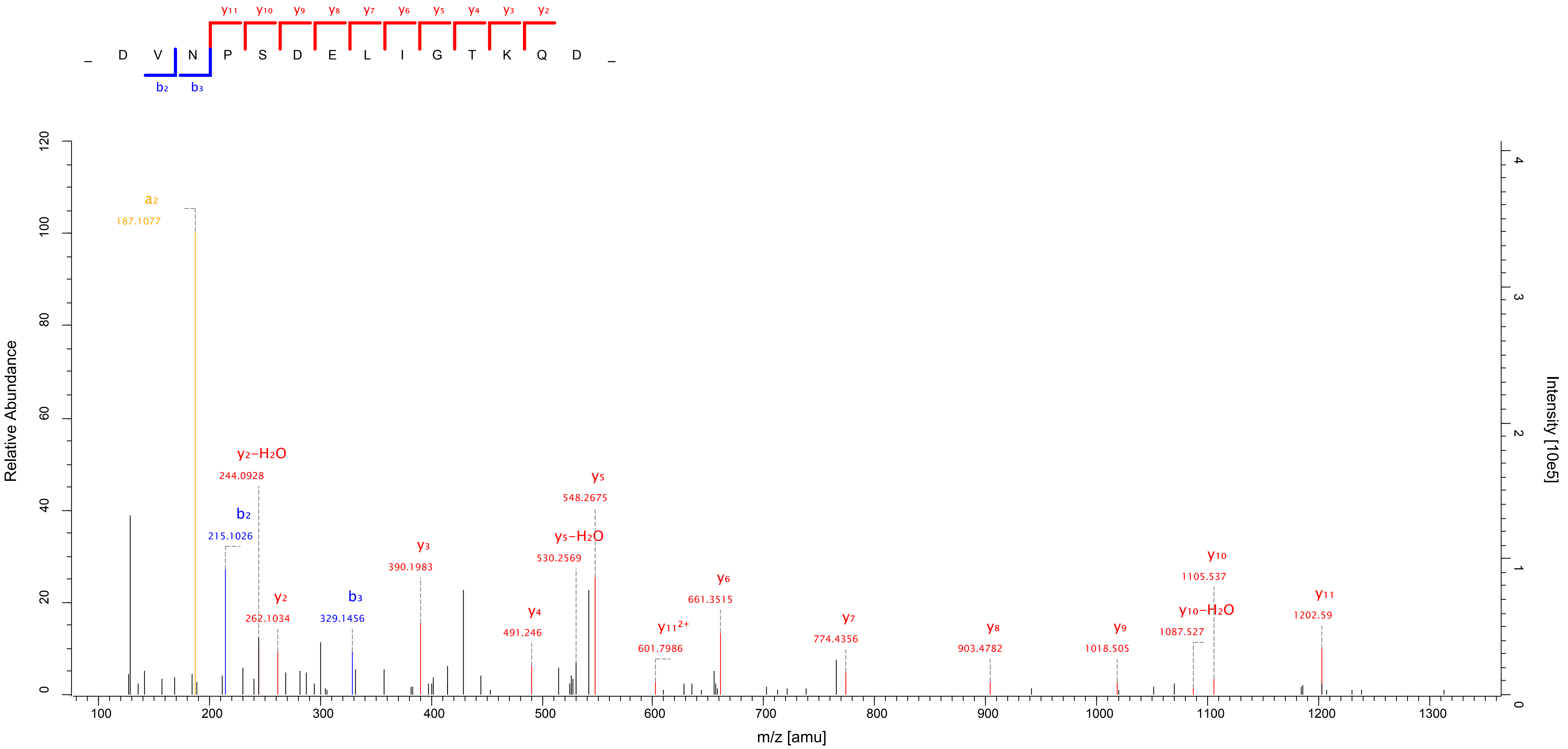

peptide ID 113

Source: 20120816_CO_0340GaJe_R02
Scannumber: 6479
Protein: pep_230; pep_secretome_1290; pep_secretome_1293; pep_secretome_1305; pep_secretome_1308
Peptide Score: 91.96
Method: FTMS; HCD; 1

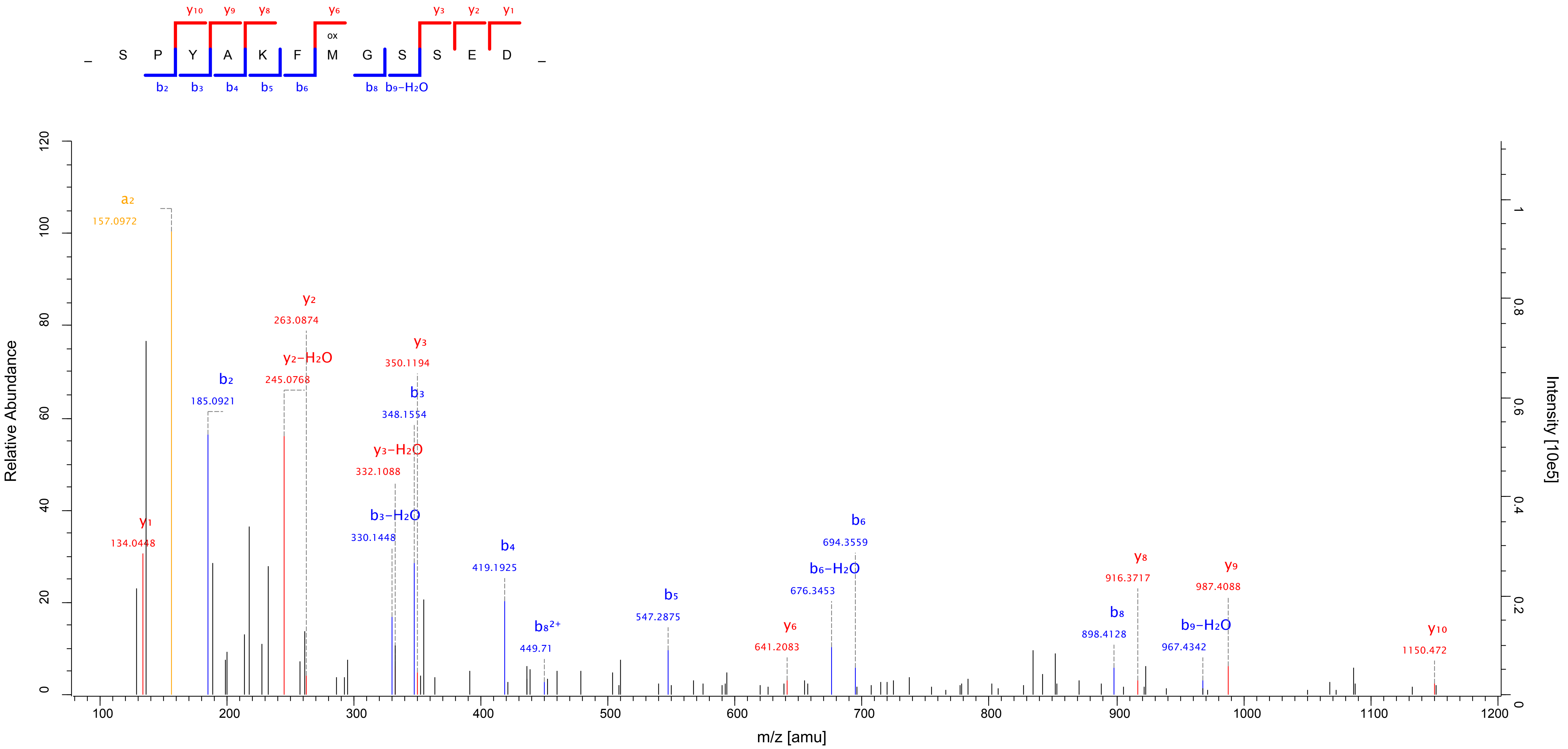

peptide ID 114

Source: 20120816_CO_0340GaJe_R02
Scannumber: 9278
Protein: pep_230; pep_secretome_1290; pep_secretome_1293; pep_secretome_1305; pep_secretome_1308
Peptide Score: 99.5
Method: FTMS; HCD; 1

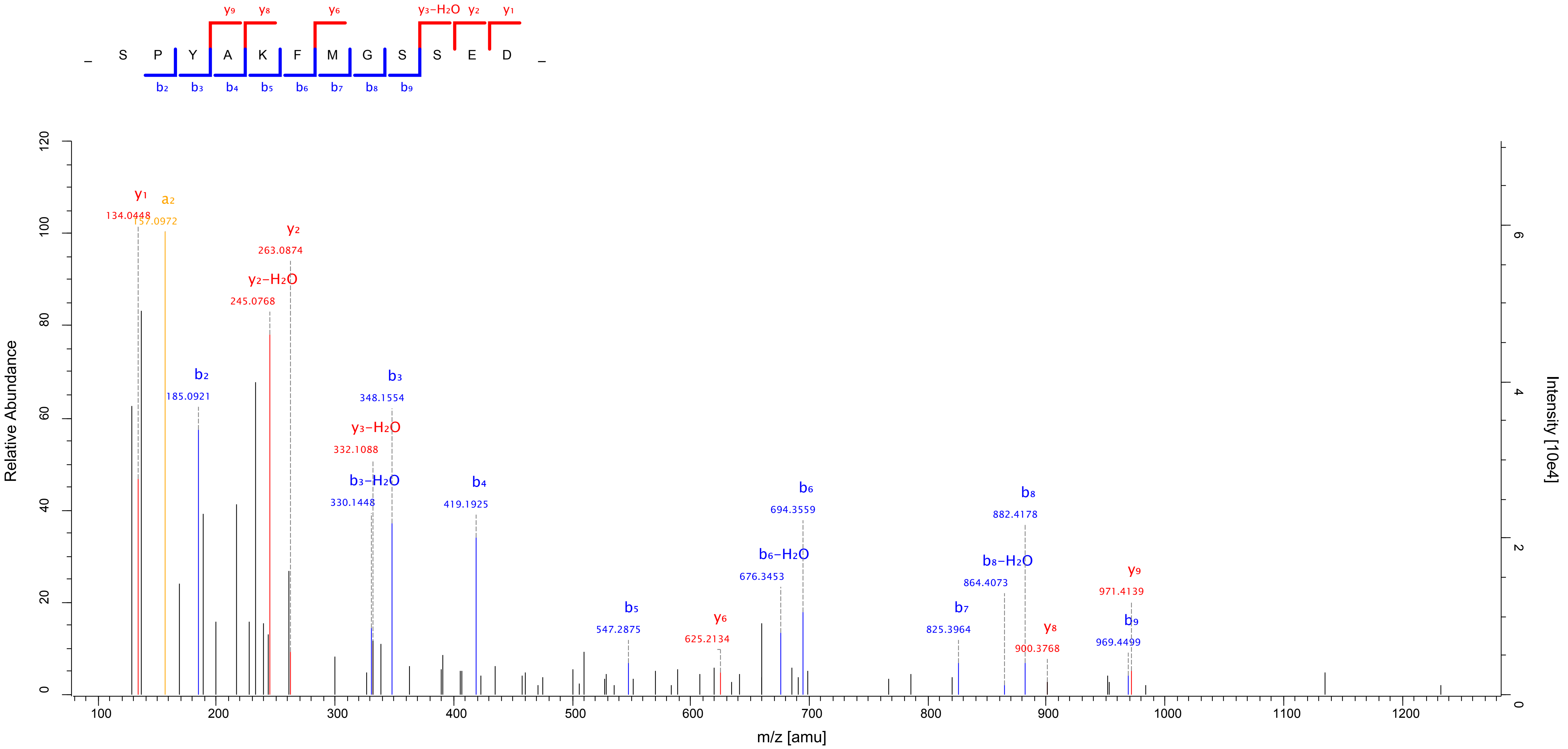

peptide ID 115

Source: 20121106_CO_0340GaJe_R02_2
Scannumber: 8215
Protein: pep_secretome_1291; pep_secretome_1297; pep_secretome_27082; pep_secretome_27093
Peptide Score: 78.34
Method: FTMS; HCD; 1

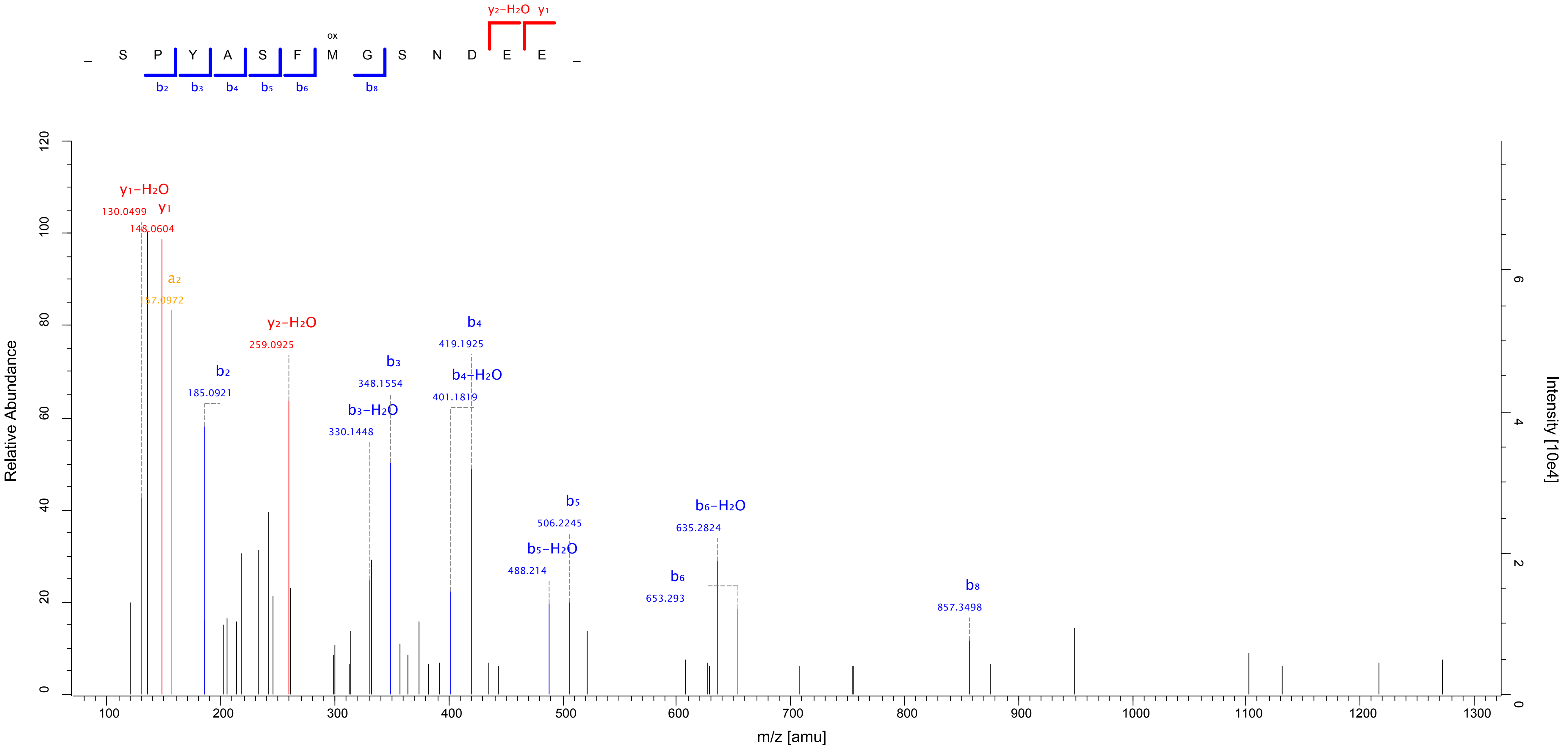

peptide ID 116

Source: 20120515_CO_0340GaJe_R01
Scannumber: 12598
Protein: pep_secretome_1292
Peptide Score: 94.62
Method: FTMS; HCD; 1

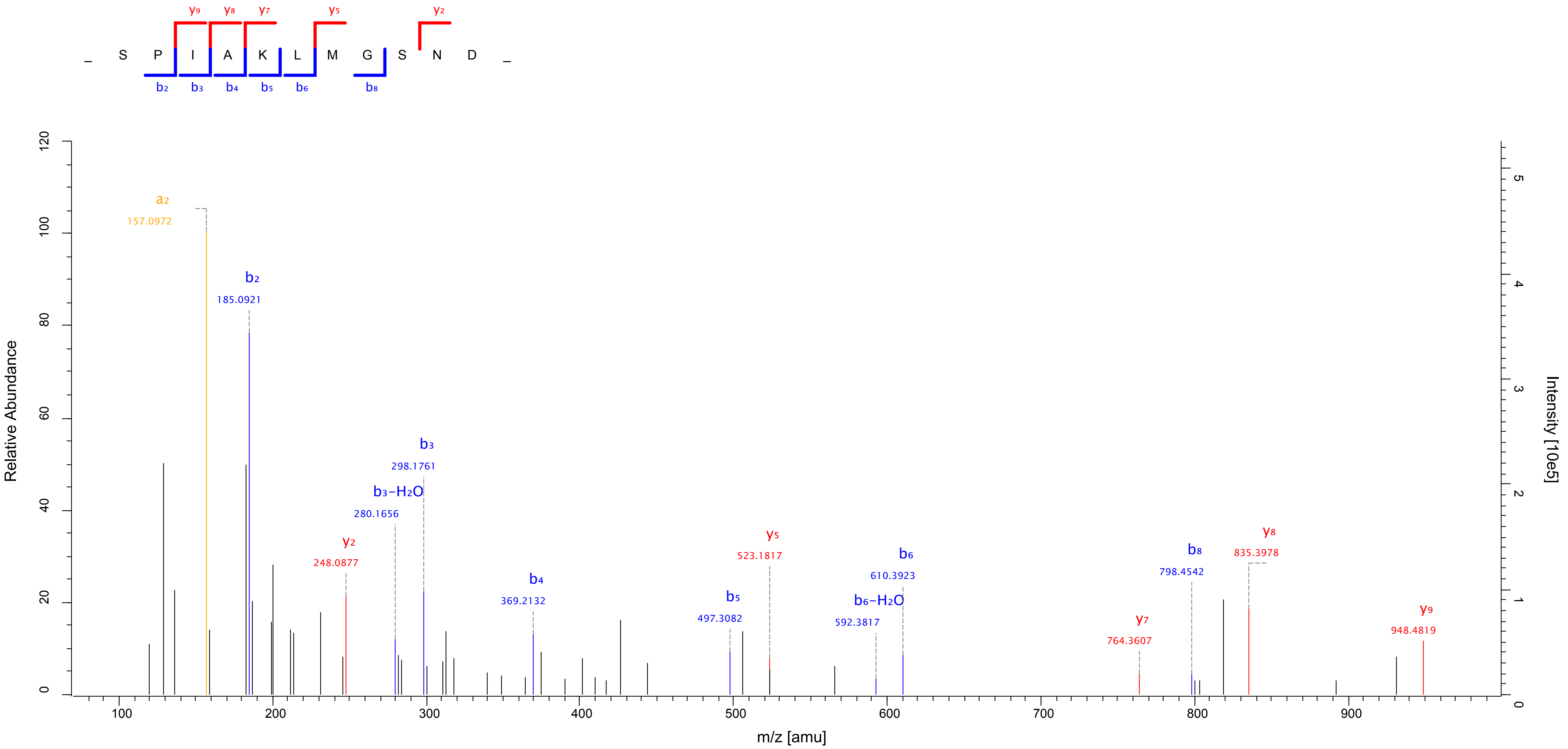

Source: 20121106_CO_0340GaJe_R02_2
Scannumber: 6535
Protein: pep_secretome_1292
Peptide Score: 97.73
Method: FTMS; HCD; 1

peptide ID 117

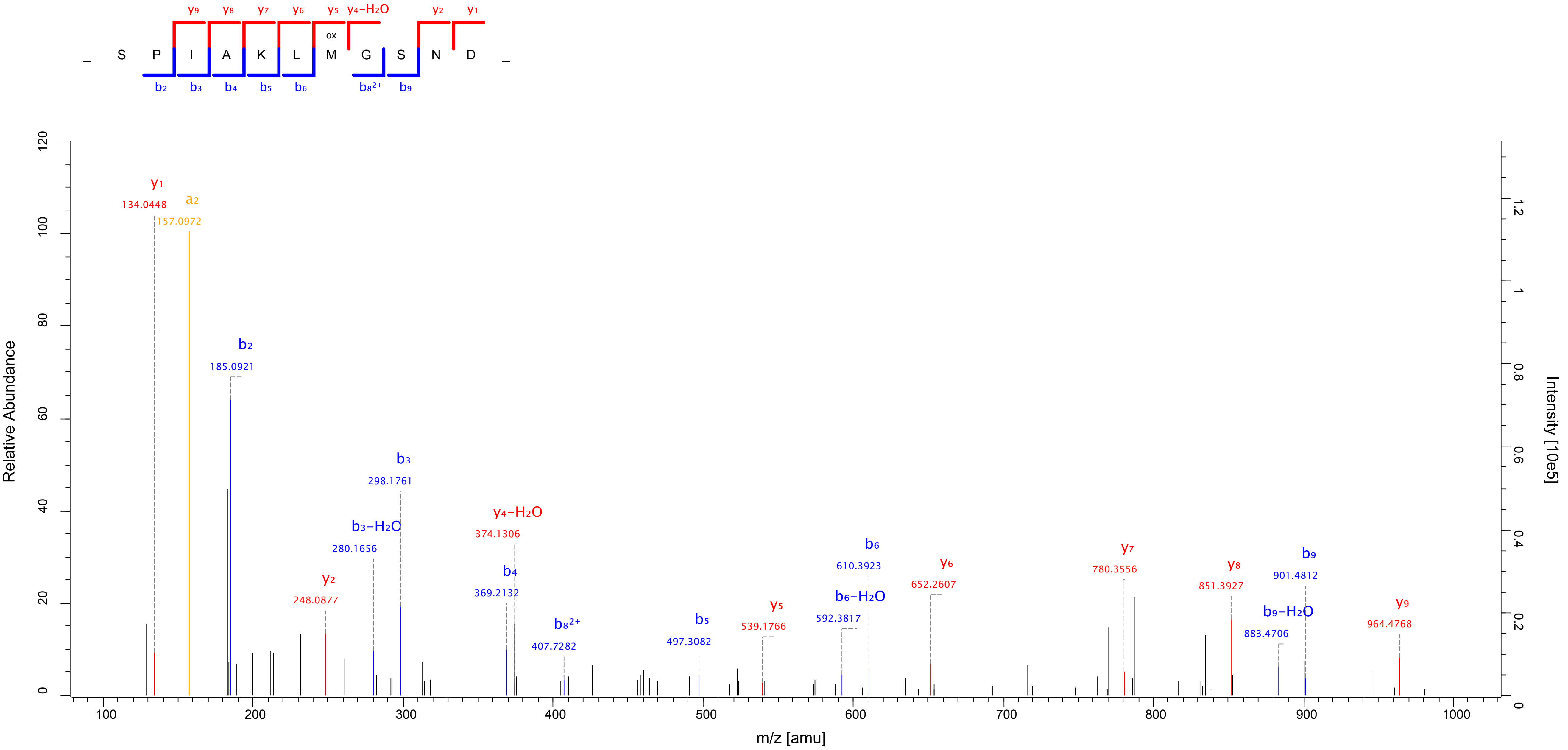

peptide ID 118

Source: 20121106_CO_0340GaJe_R02_2
Scannumber: 5579
Protein: pep_secretome_1295
Peptide Score: 76.66
Method: FTMS; HCD; 1

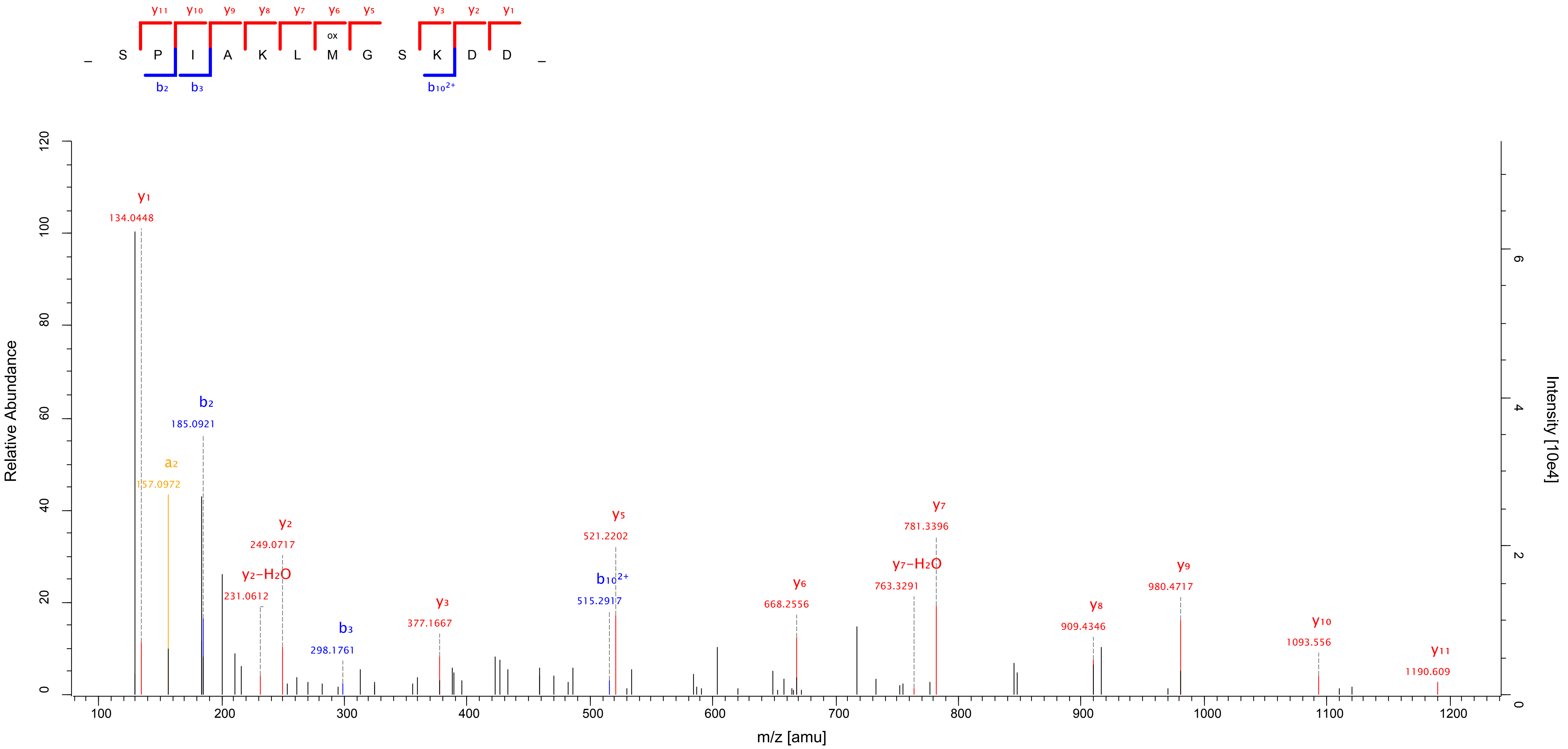

peptide ID 119

Source: 20120816_CO_0340GaJe_R02
Scannumber: 10561
Protein: pep_secretome_1298
Peptide Score: 105.03
Method: FTMS; HCD; 1

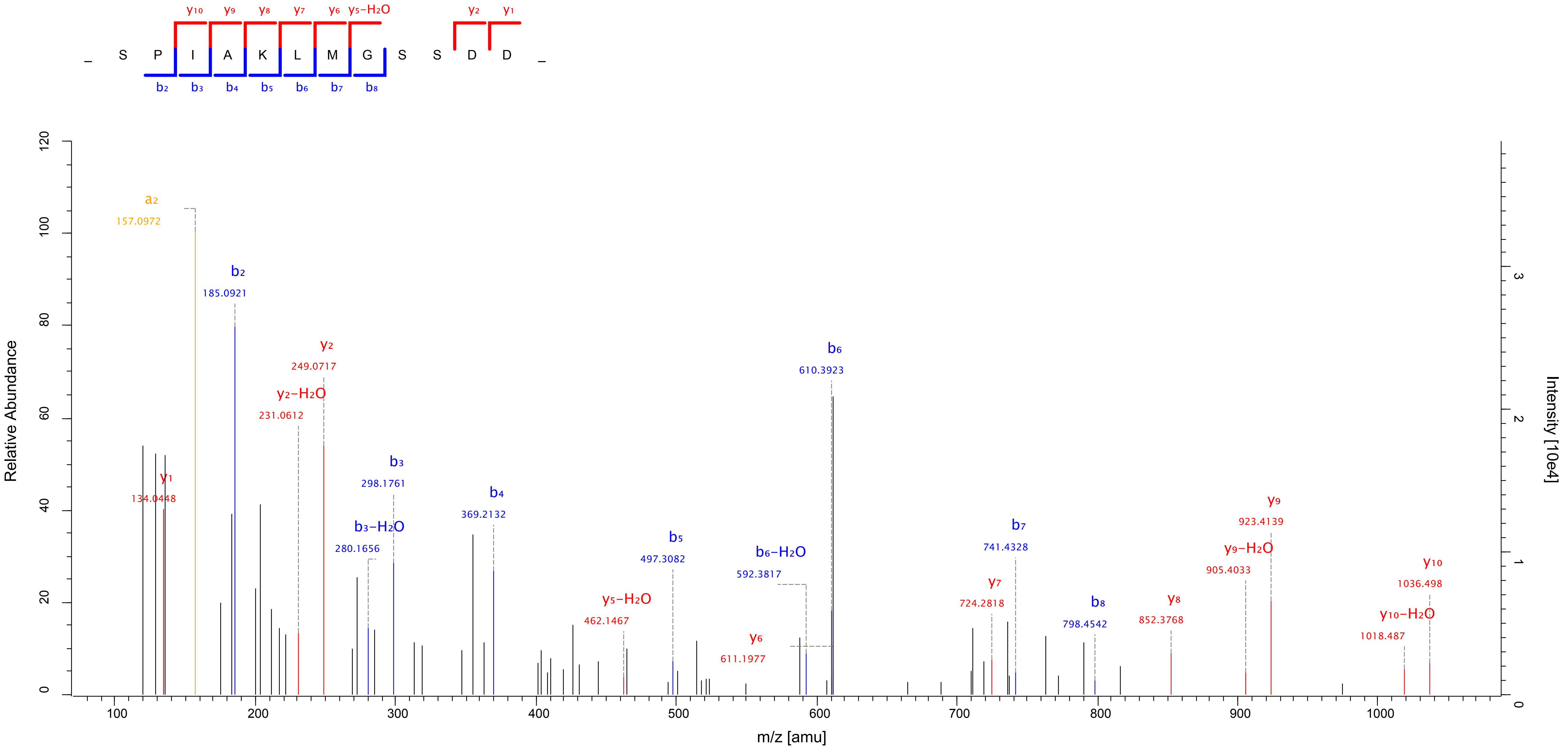

peptide ID 120

Source:	20121106_CO_0340GaJe_R02_2
Scannumber:	6727
Protein:	pep_secretome_1298
Peptide Score:	98.16
Method:	FTMS; HCD; 1

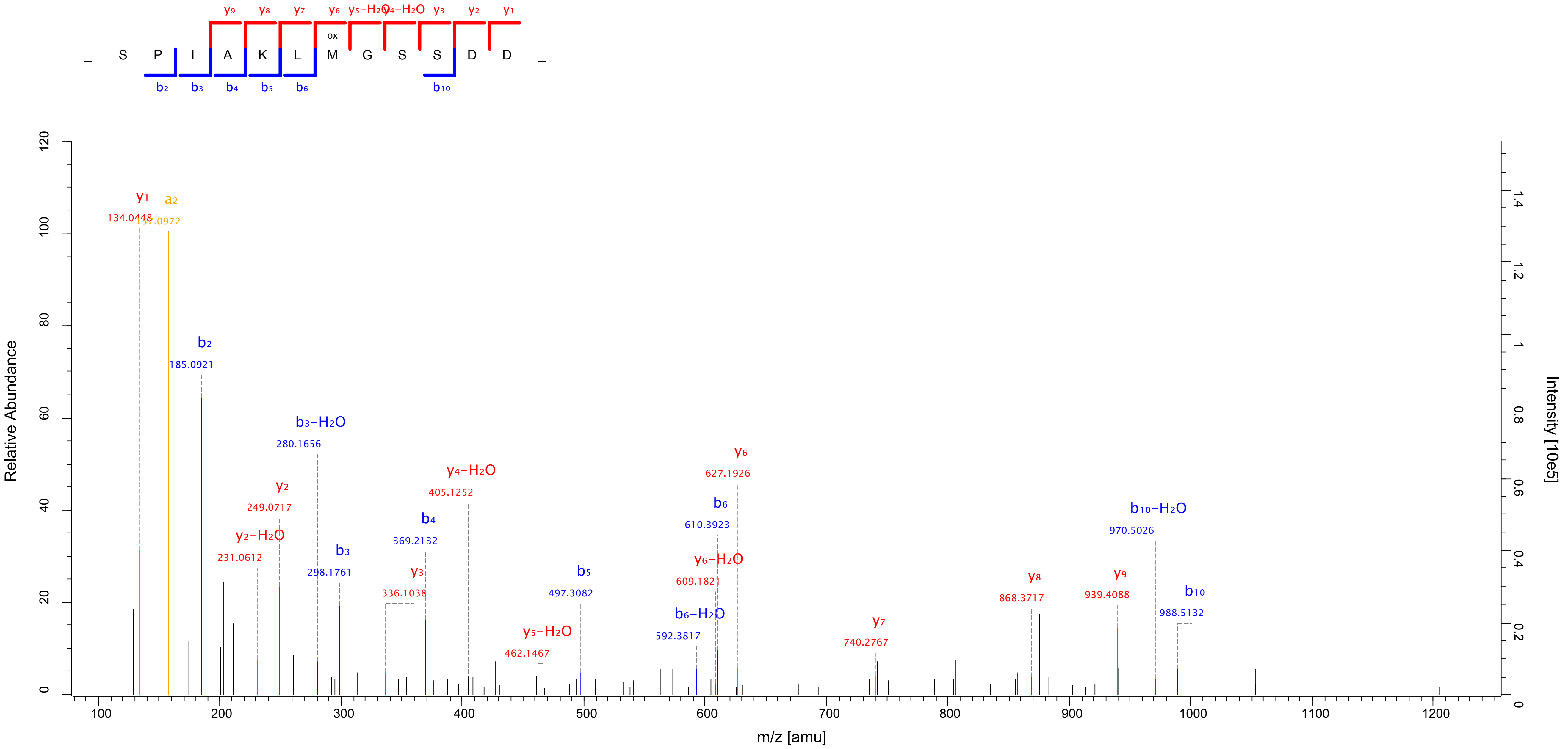

peptide ID 121

Source: 20120816_CO_0340GaJe_R02
Scannumber: 9922
Protein: pep_229; pep_secretome_1304
Peptide Score: 83.05
Method: FTMS; HCD; 1

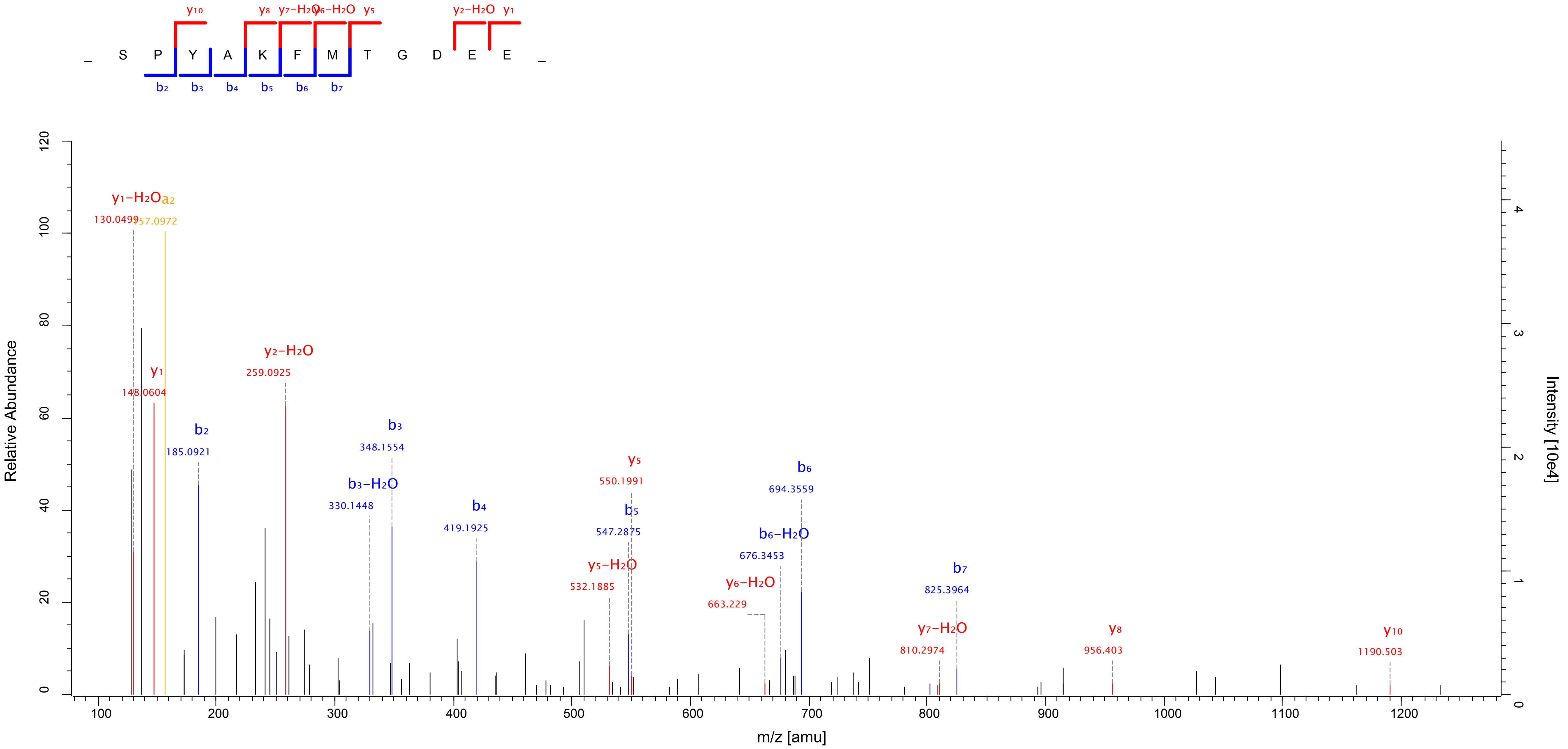

peptide ID 122

Source:	20120816_CO_0340GaJe_R02
Scannumber:	7300
Protein:	pep_229; pep_secretome_1304
Peptide Score:	99.5
Method:	FTMS; HCD; 1

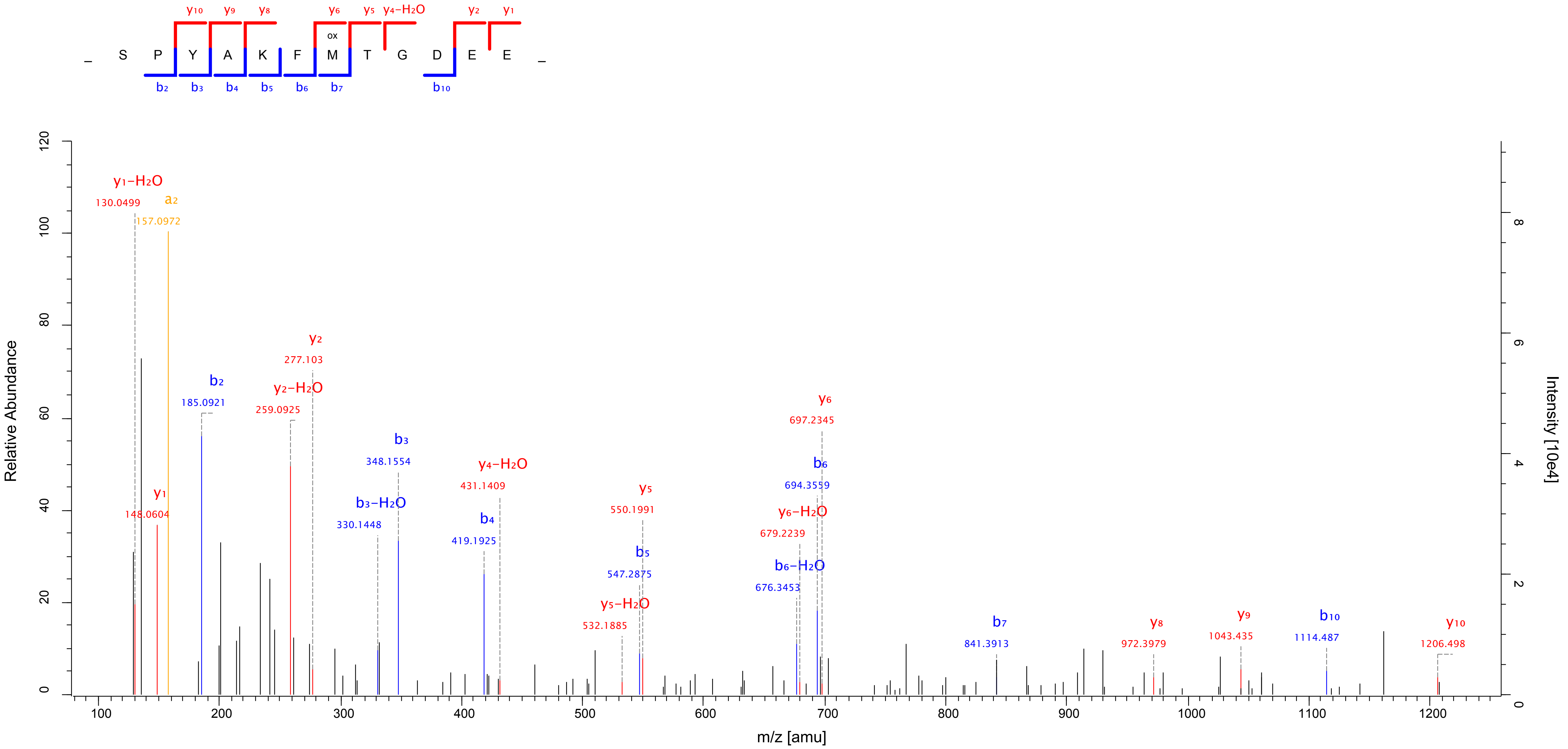

peptide ID 123

Source: 20120816_CO_0340GaJe_R02
Scannumber: 9185
Protein: pep_231; pep_secretome_1306
Peptide Score: 65.4
Method: FTMS; HCD; 1

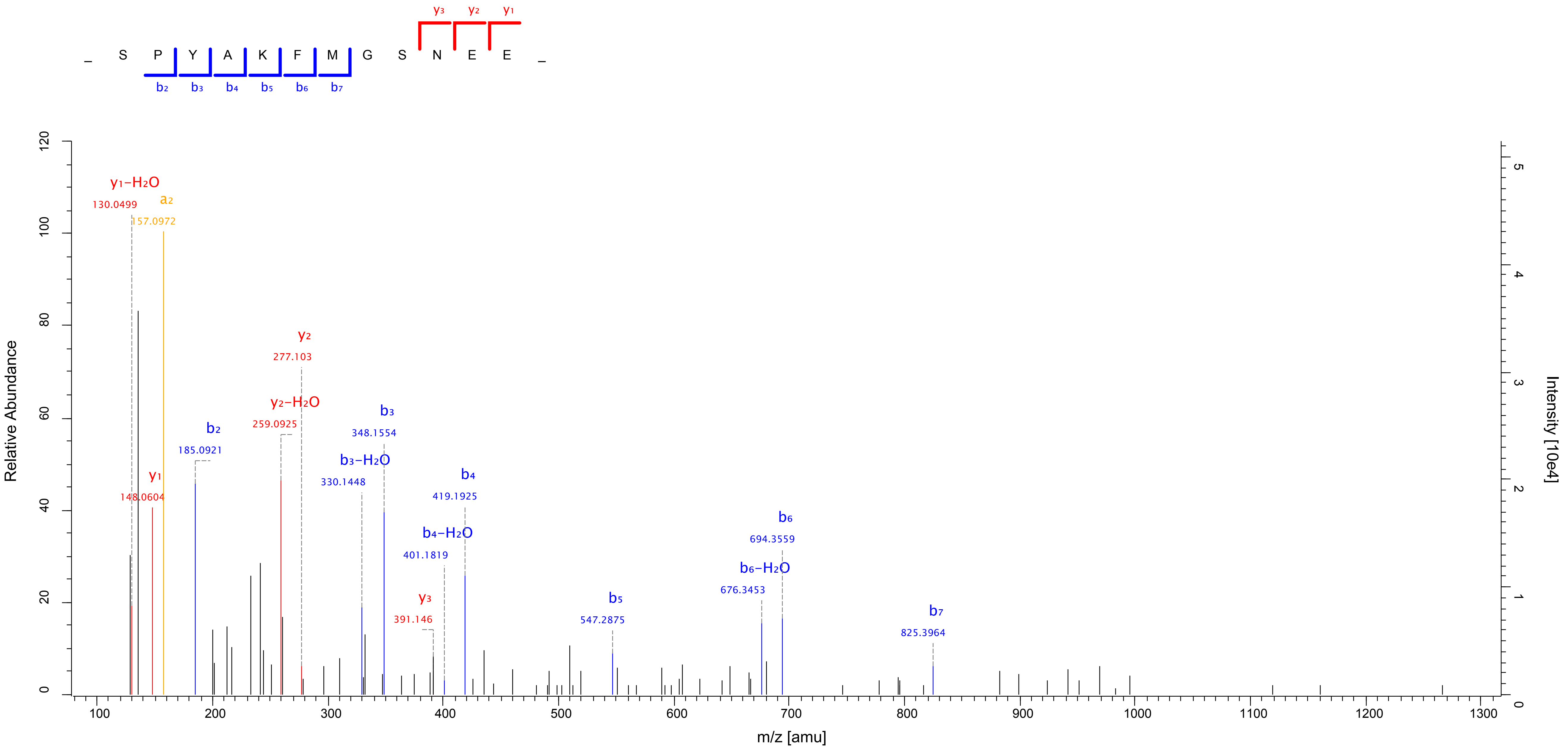

peptide ID 124

Source: 20120816_CO_0340GaJe_R02
Scannumber: 10509
Protein: pep_secretome_1310
Peptide Score: 83.87
Method: FTMS; HCD; 1

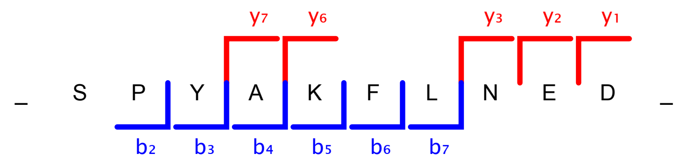

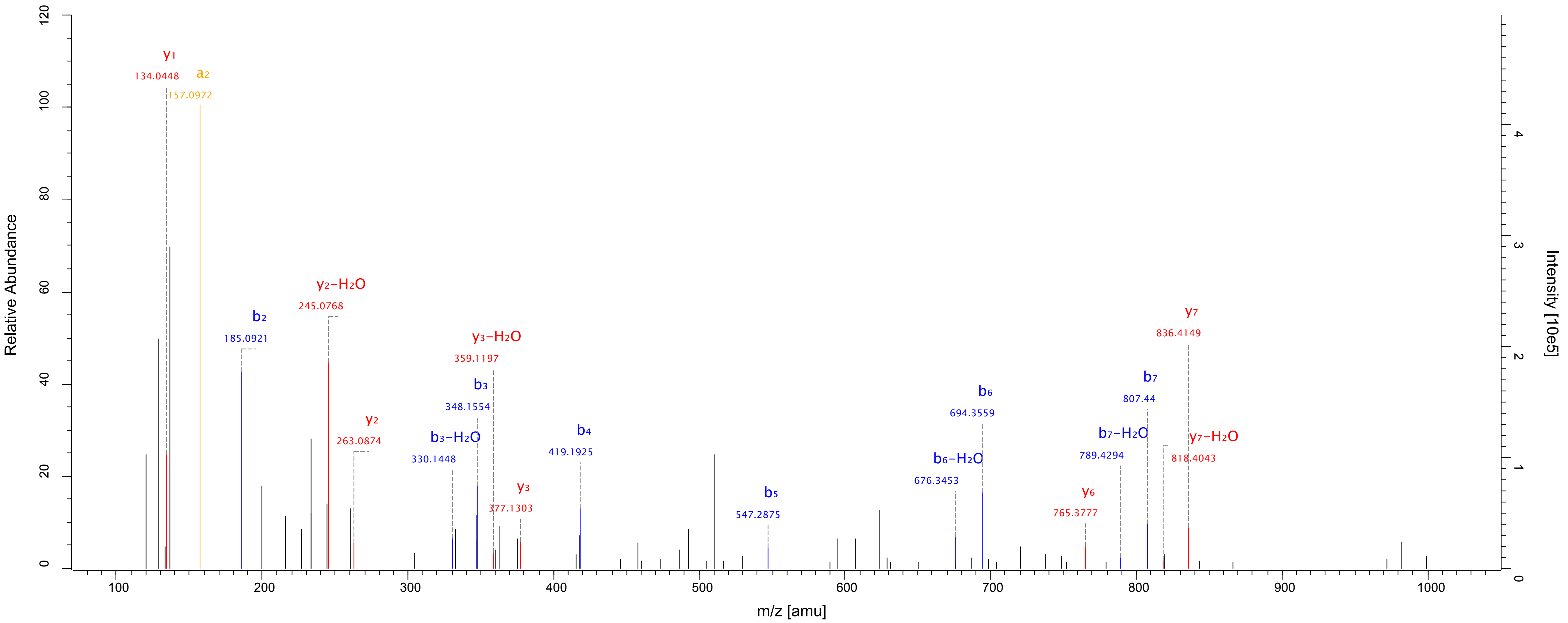

Source: 20120816_CO_0340GaJe_R02
Scannumber: 15129
Protein: pep_30; pep_secretome_79
Peptide Score: 31.82
Method: FTMS; HCD; 1

peptide ID 125

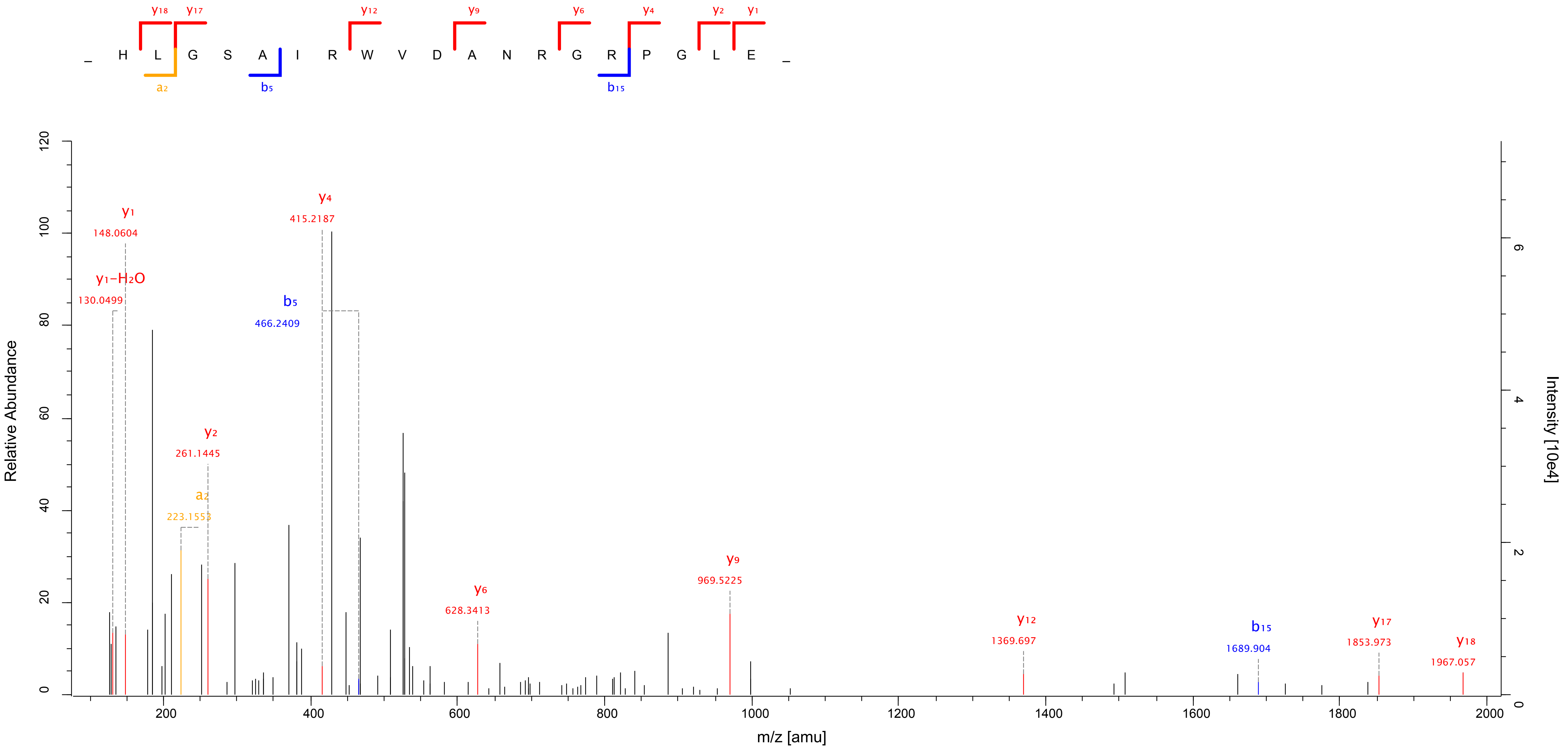

peptide ID 126

Source: 20121106_CO_0340GaJe_R02_2
Scannumber: 6318
Protein: pep_47; pep_secretome_22721; pep_secretome_96
Peptide Score: 75.46
Method: FTMS; HCD; 1

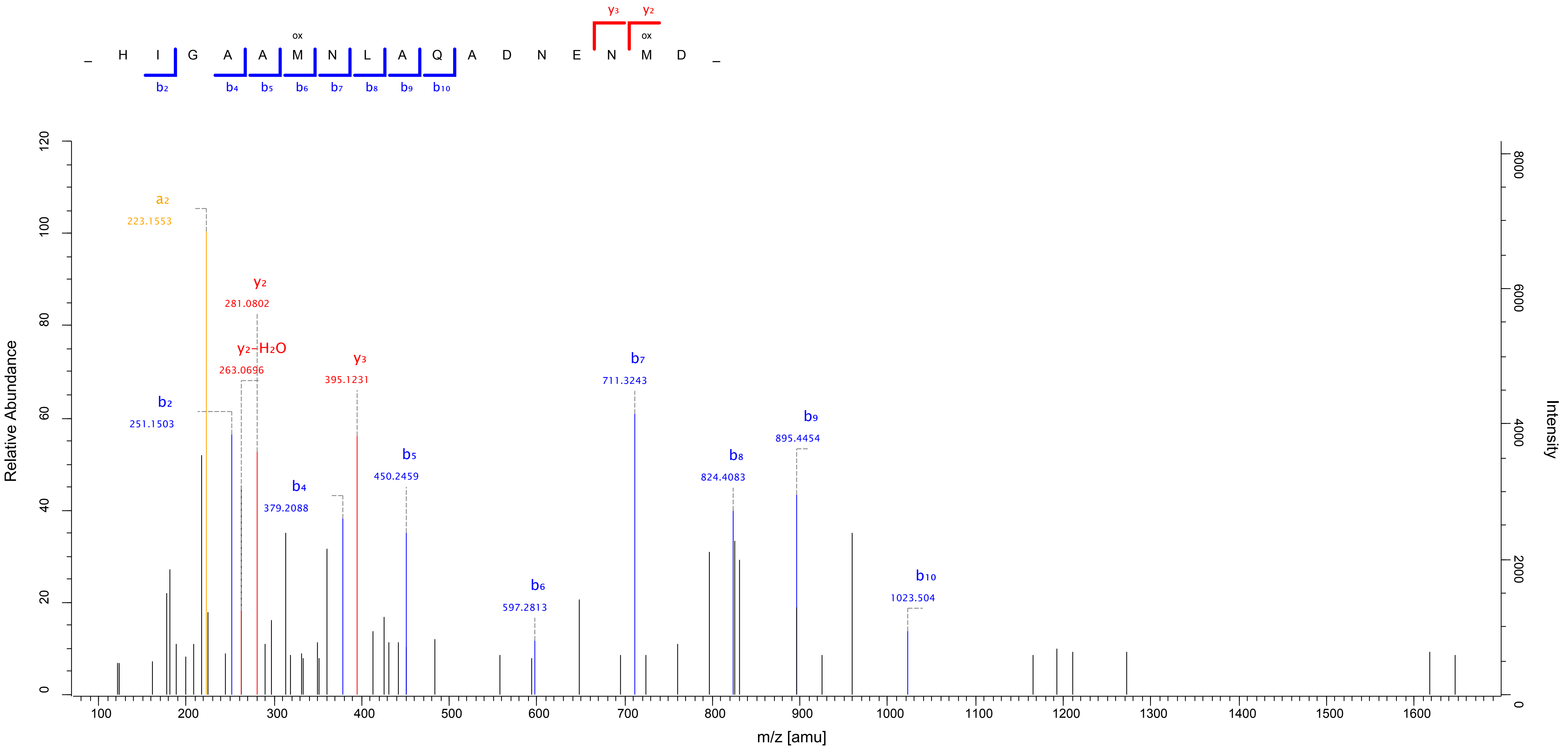

peptide ID 127

Source: 20120816_CO_0340GaJe_R02
Scannumber: 9363
Protein: pep_49; pep_secretome_98
Peptide Score: 34.91
Method: FTMS; HCD; 1

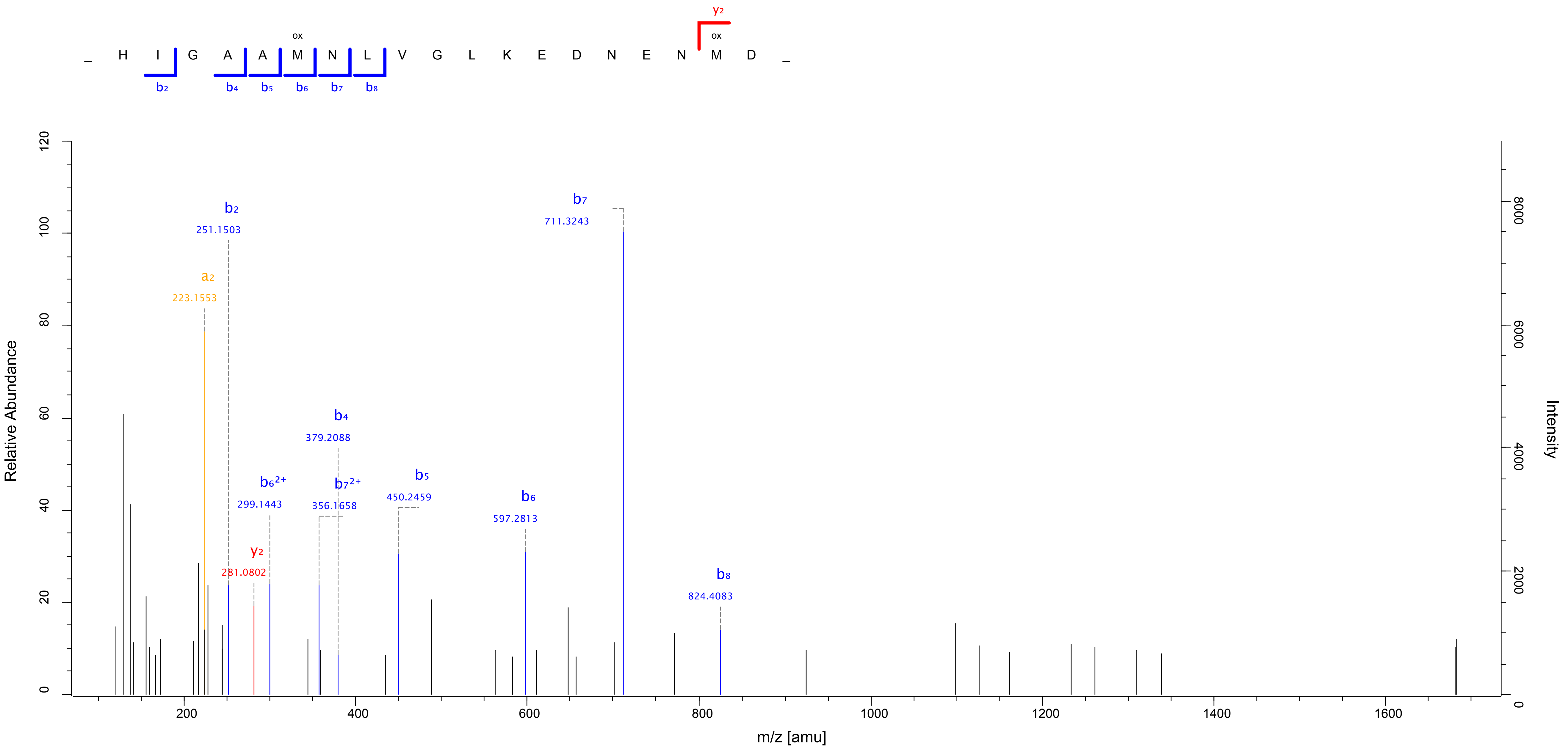

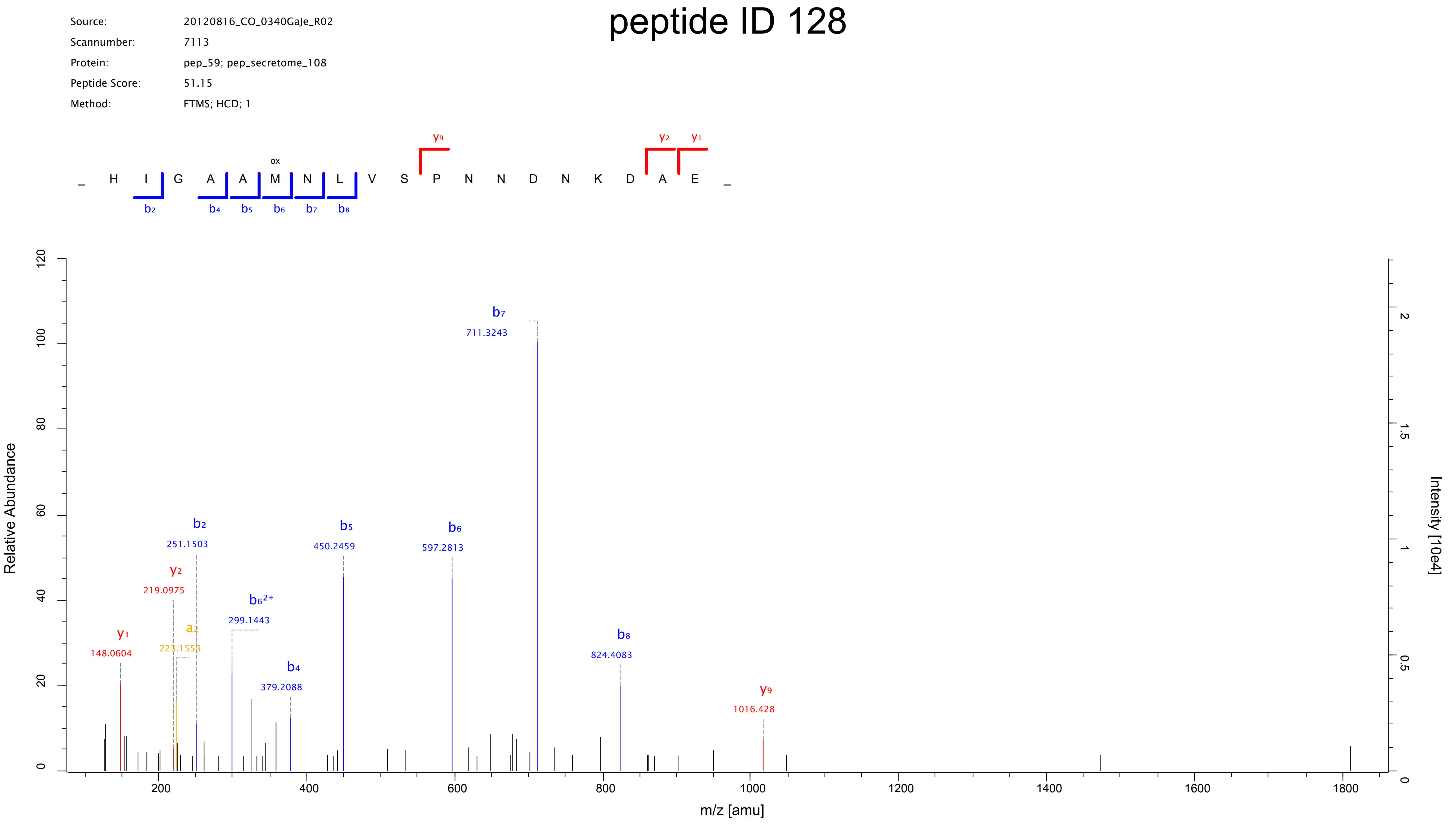

Source: 20120816_CO_0340GaJe_R02
Scannumber: 7113
Protein: pep_59; pep_secretome_108
Peptide Score: 51.15
Method: FTMS; HCD; 1
peptide ID 128
_ H I G A A M N L V S P N N D N K D A E _
ox
y9 y2 y1
b2 b4 b5 b6 b7 b8
b7 711.3243
b2 251.1503
b5 450.2459
b6 597.2813
y2 219.0975
b6 2+ 299.1443
y1 148.0604
a2 223.1553
b8 824.4083
b4 379.2088
y9 1016.428
Relative Abundance
Intensity [10e4]
m/z [amu]

peptide ID 129

Source:	20120816_CO_0340GaJe_R02
Scannumber:	16095
Protein:	pep_secretome_587
Peptide Score:	79.77
Method:	FTMS; HCD; 1

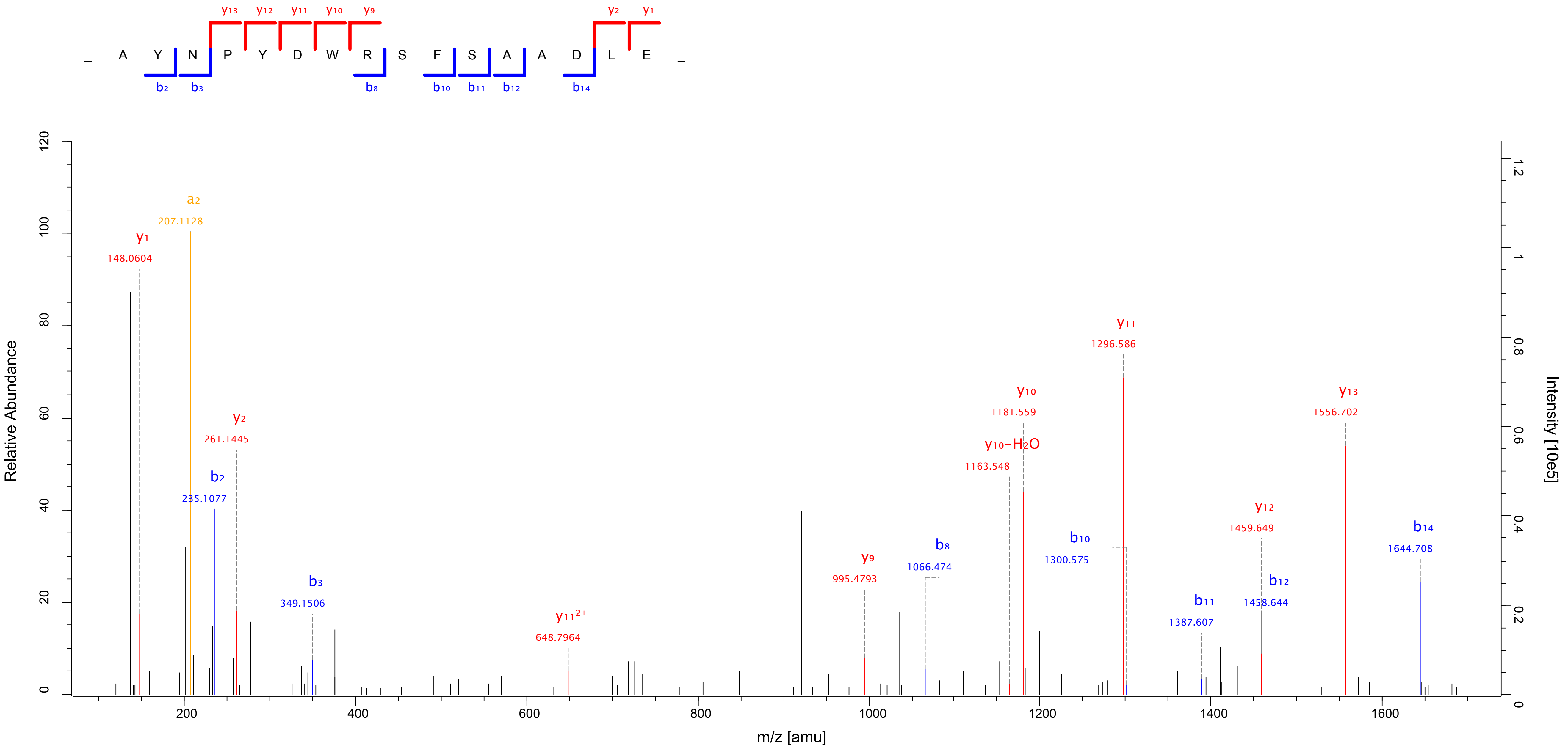

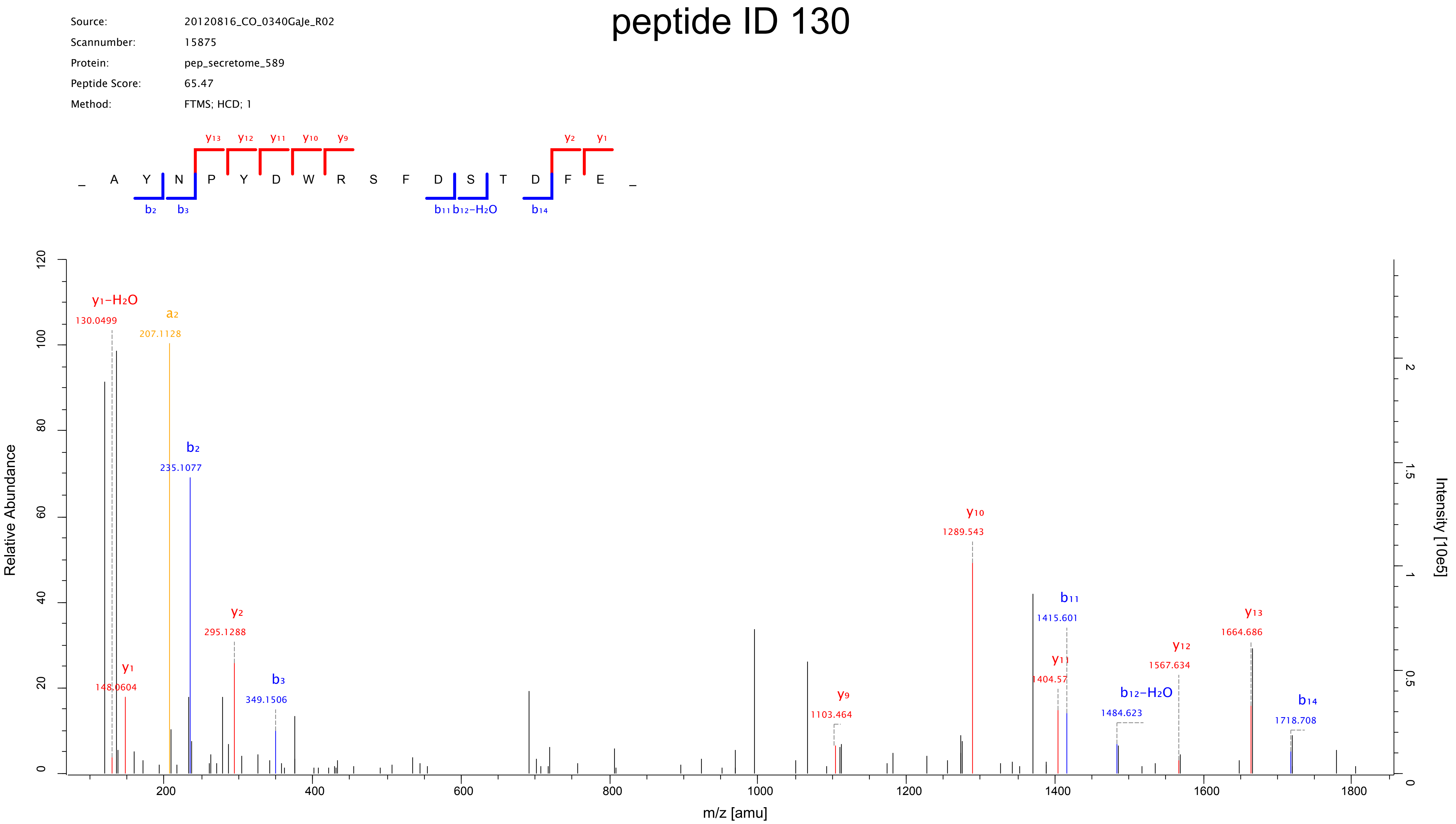
peptide ID 130
Source: 20120816_CO_0340GaJe_R02
Scannumber: 15875
Protein: pep_secretome_589
Peptide Score: 65.47
Method: FTMS; HCD; 1
_ A Y N P Y D W R S F D S T D F E _
y13 y12 y11 y10 y9 y2 y1
b2 b3 b11 b12-H2O b14
y1-H2O 130.0499
a2 207.1128
b2 235.1077
y2 295.1288
y1 148.0604
b3 349.1506
y9 1103.464
y10 1289.543
y11 1404.57
b11 1415.601
b12-H2O 1484.623
y12 1567.634
y13 1664.686
b14 1718.708
Relative Abundance
Intensity [10e5]
m/z [amu]
0
20
40
60
80
100
120
200
400
600
800
1000
1200
1400
1600
1800
0.5
1
1.5
2

Source: 20120816_CO_0340GaJe_R02
Scannumber: 12513
Protein: pep_secretome_16655; pep_secretome_87516
Peptide Score: 89.3
Method: FTMS; HCD; 1

peptide ID 131

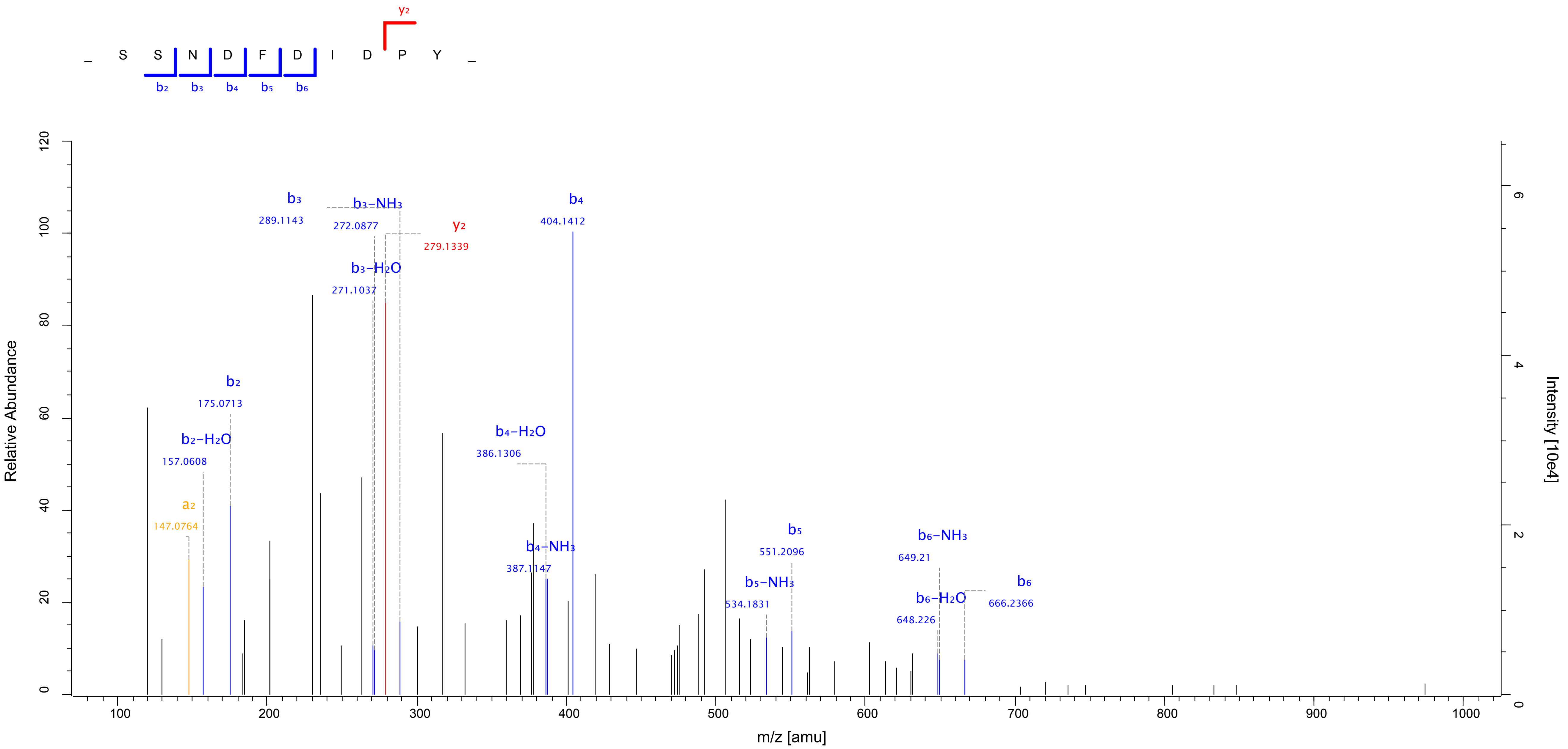

peptide ID 132

Source: 20120816_CO_0340GaJe_R02
Scannumber: 9705
Protein: pep_secretome_16657; pep_secretome_16659; pep_secretome_87519; pep_secretome_87521
Peptide Score: 57.52
Method: FTMS; HCD; 1

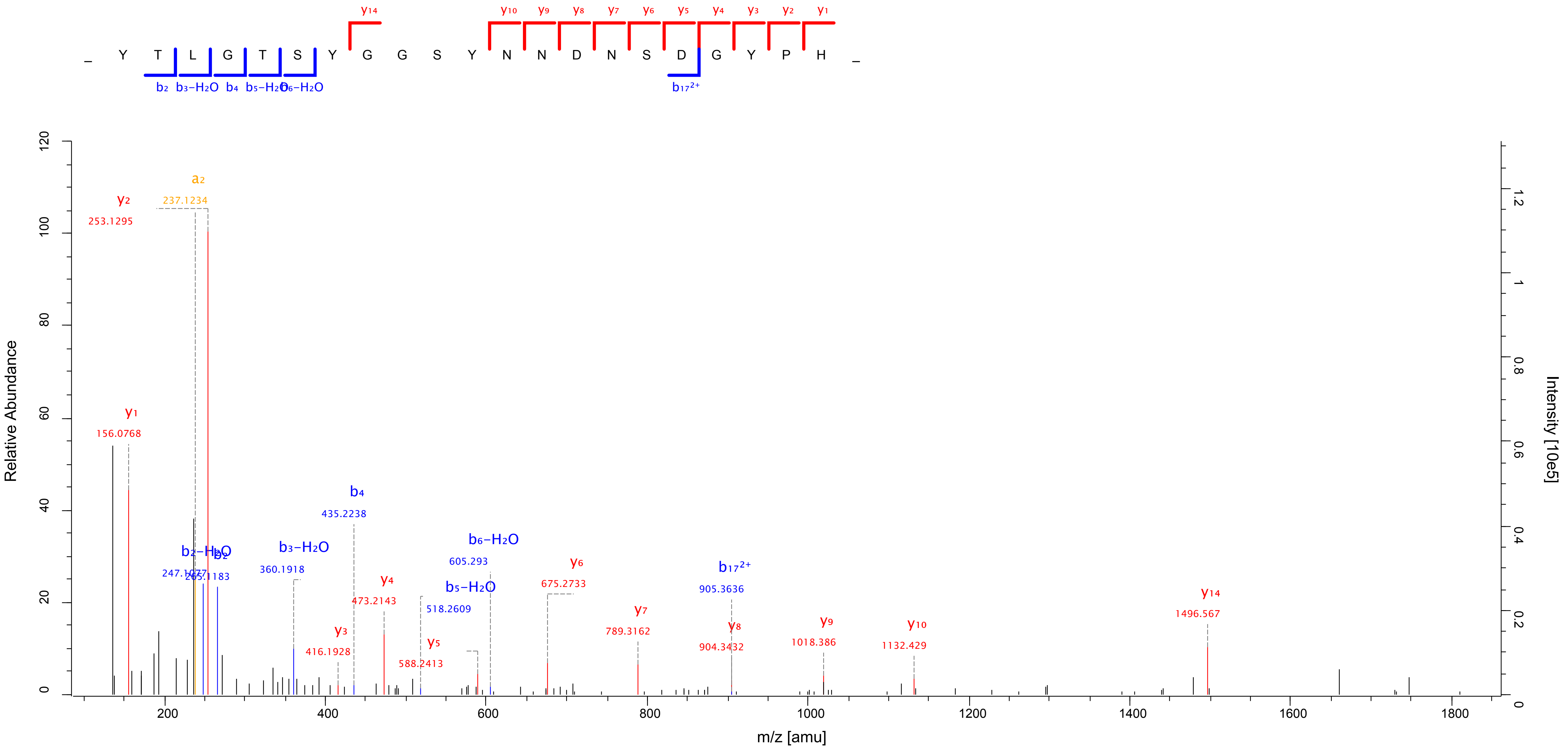

Source: 20121106_CO_0340GaJe_R02_2
Scannumber: 10810
Protein: pep_secretome_2788
Peptide Score: 83.31
Method: FTMS; HCD; 1

peptide ID 133

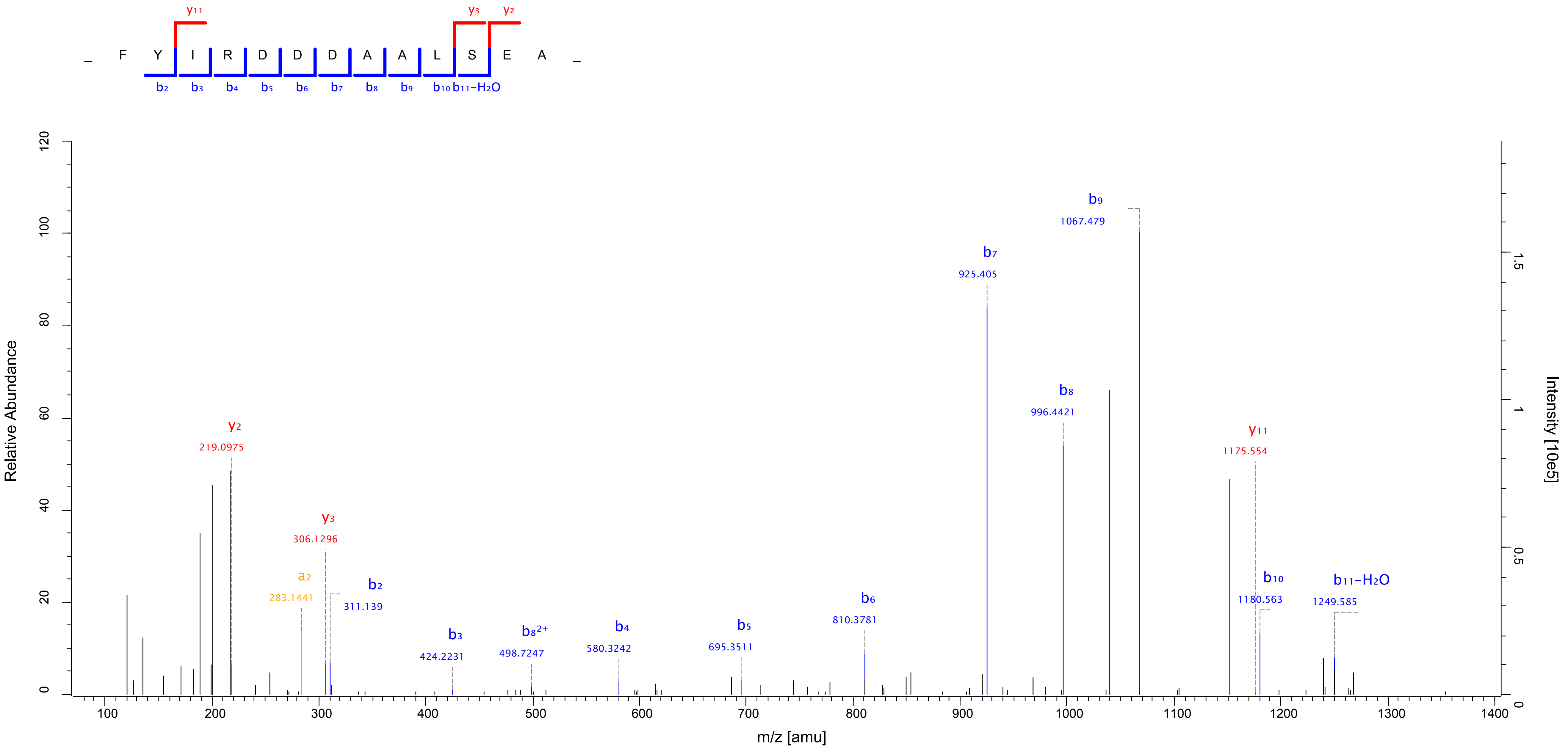

Source: 20121106_CO_0340GaJe_R02_2
Scannumber: 16846
Protein: pep_secretome_2789
Peptide Score: 135.23
Method: FTMS; HCD; 1

peptide ID 134

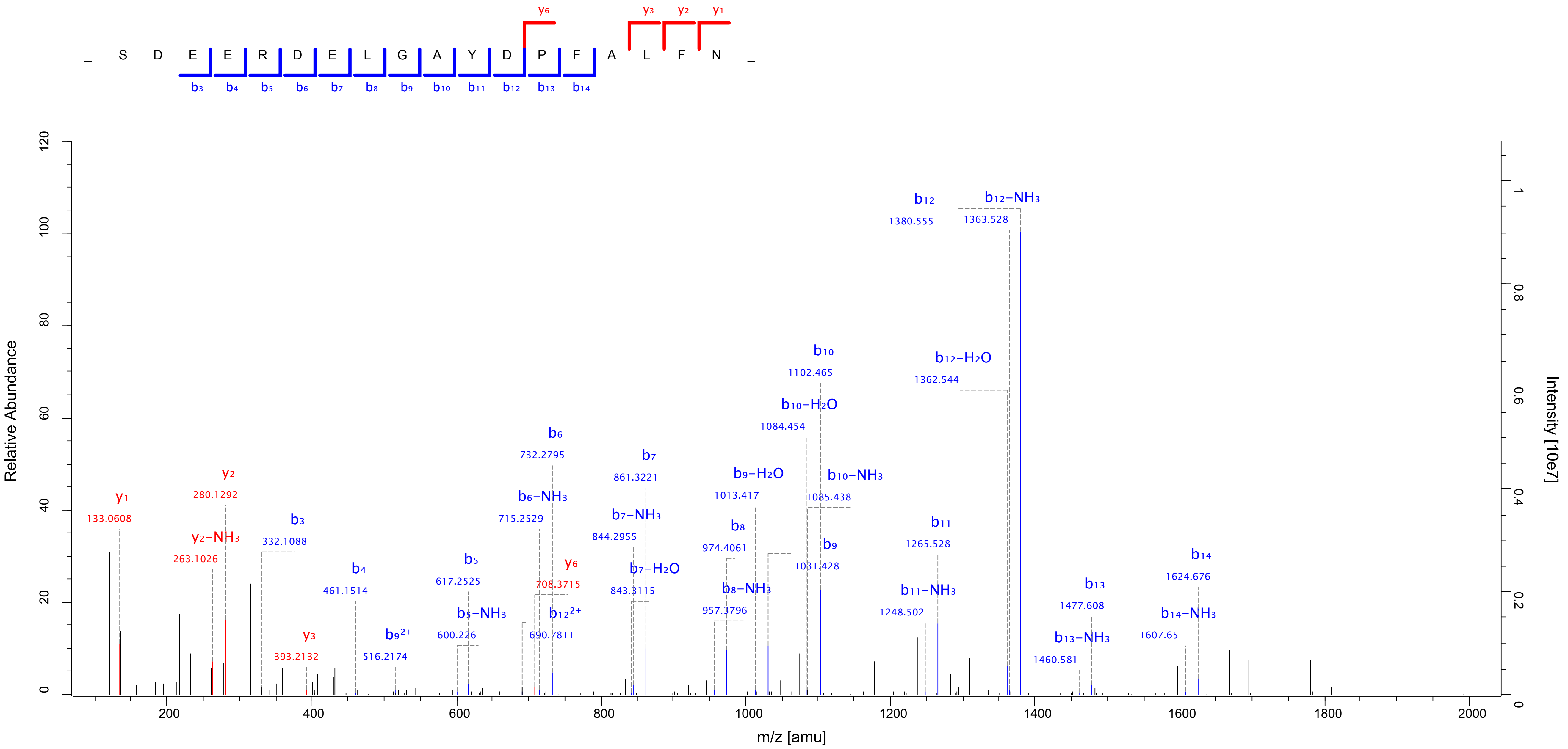

Source: 20121106_CO_0340GaJe_R02_2
Scannumber: 17118
Protein: pep_secretome_2792; pep_secretome_34564
Peptide Score: 44.5
Method: FTMS; HCD; 1

peptide ID 135

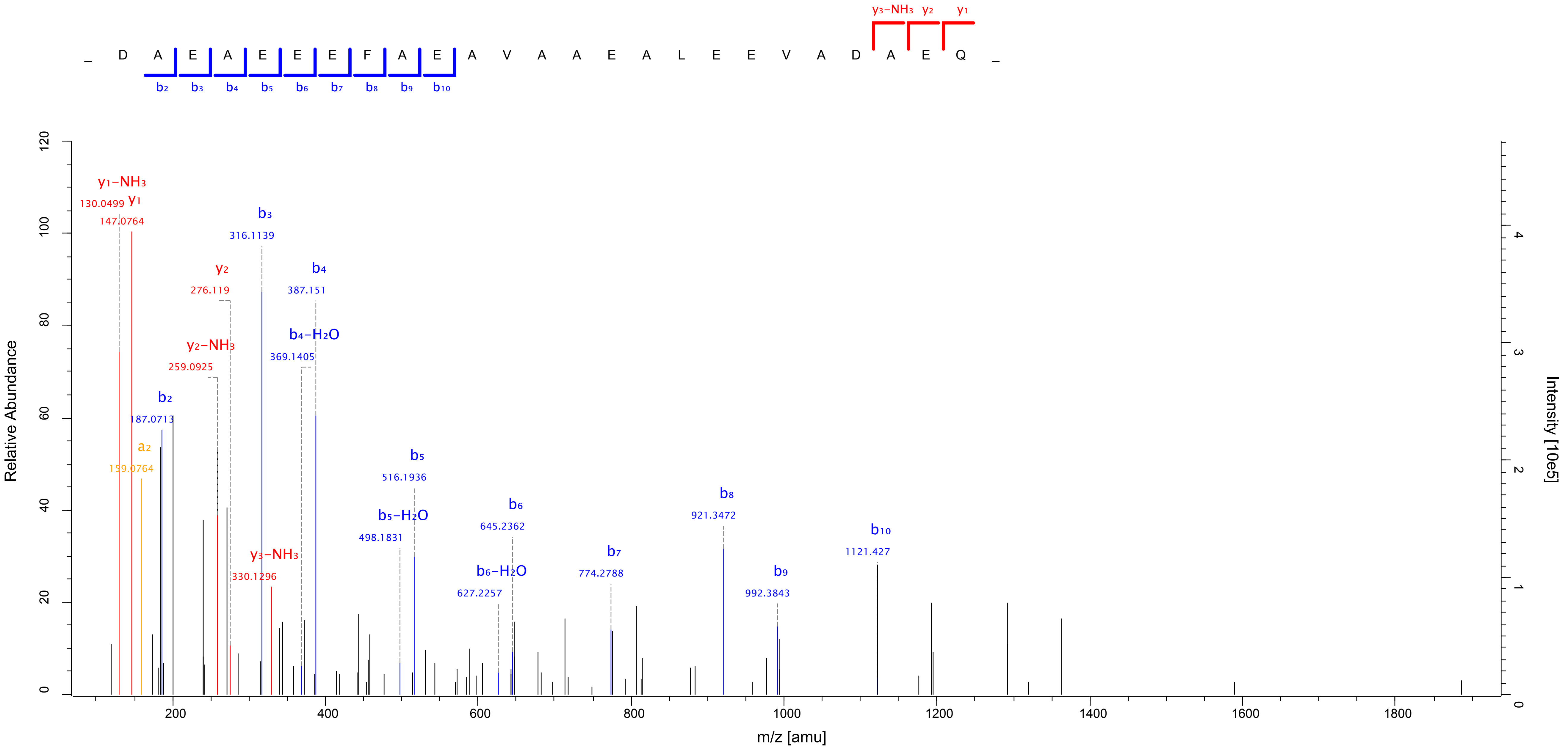

peptide ID 136

Source:	20120816_CO_0340GaJe_R02
Scannumber:	17009
Protein:	pep_secretome_2803; pep_secretome_34576
Peptide Score:	63.18
Method:	FTMS; HCD; 1

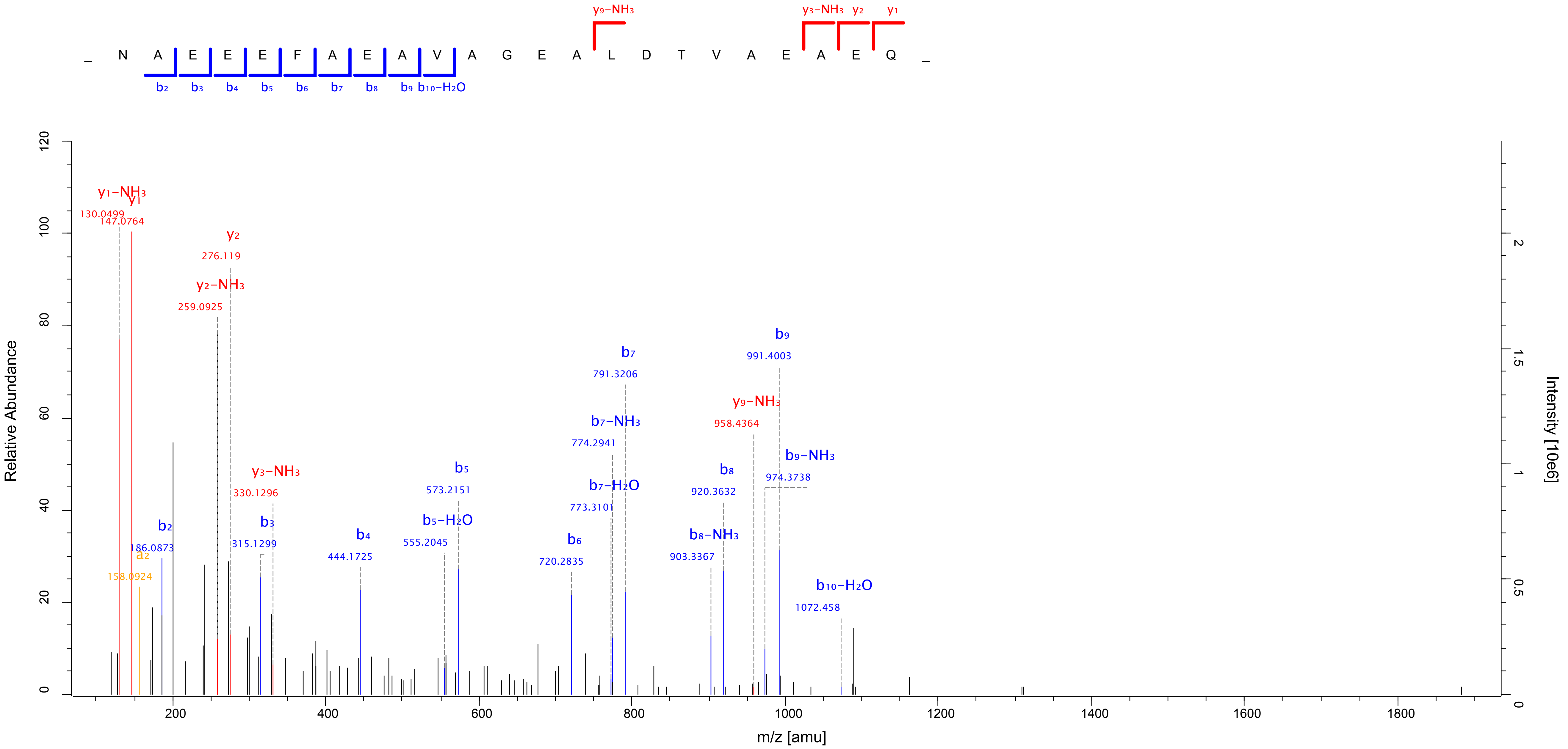

Source: 20120816_CO_0340GaJe_R02
Scannumber: 10070
Protein: pep_secretome_2806
Peptide Score: 38.61
Method: FTMS; HCD; 1

peptide ID 137

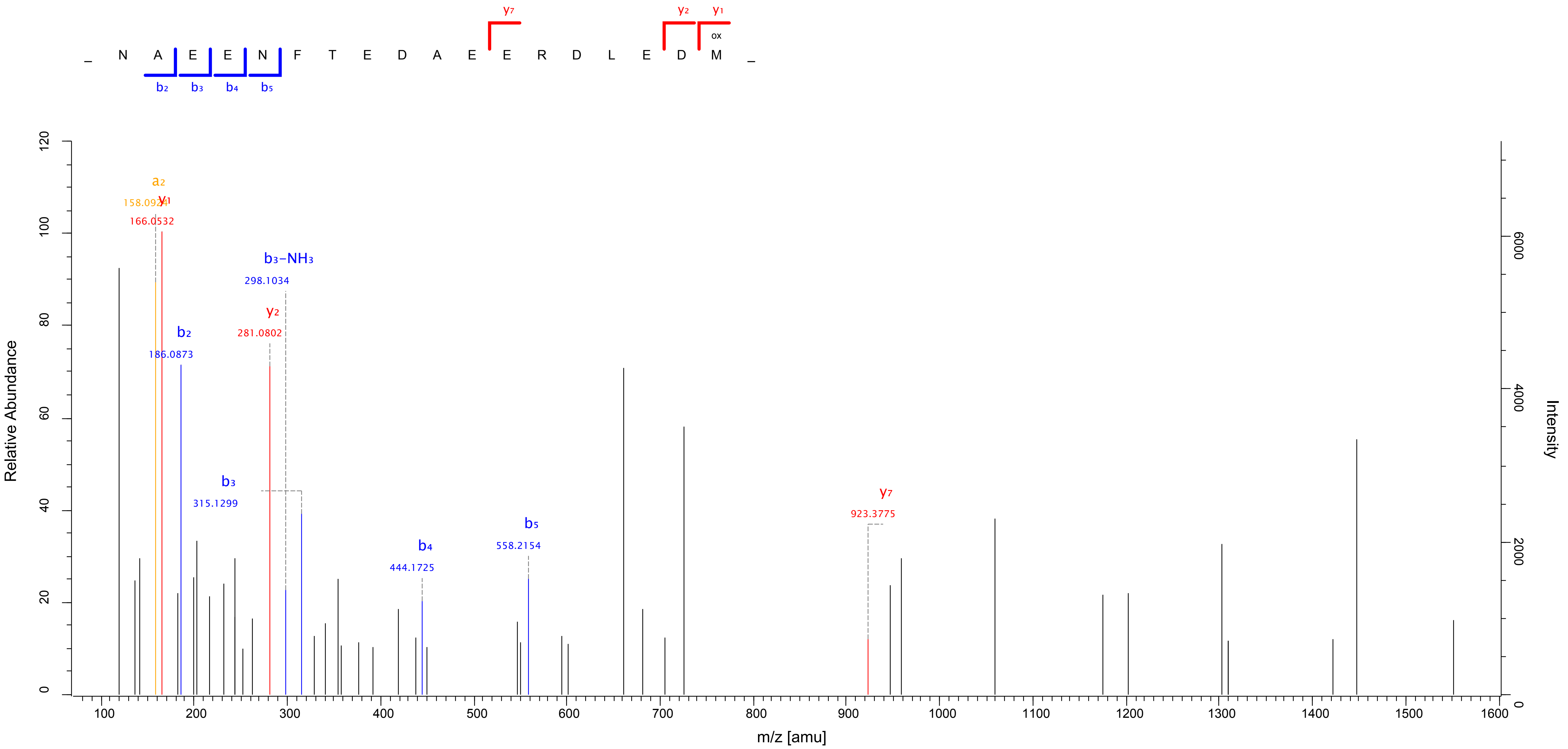

peptide ID 138

Source: 20120816_CO_0340GaJe_R02
Scannumber: 16545
Protein: pep_secretome_8011; SinglePep89
Peptide Score: 70.28
Method: FTMS; HCD; 1

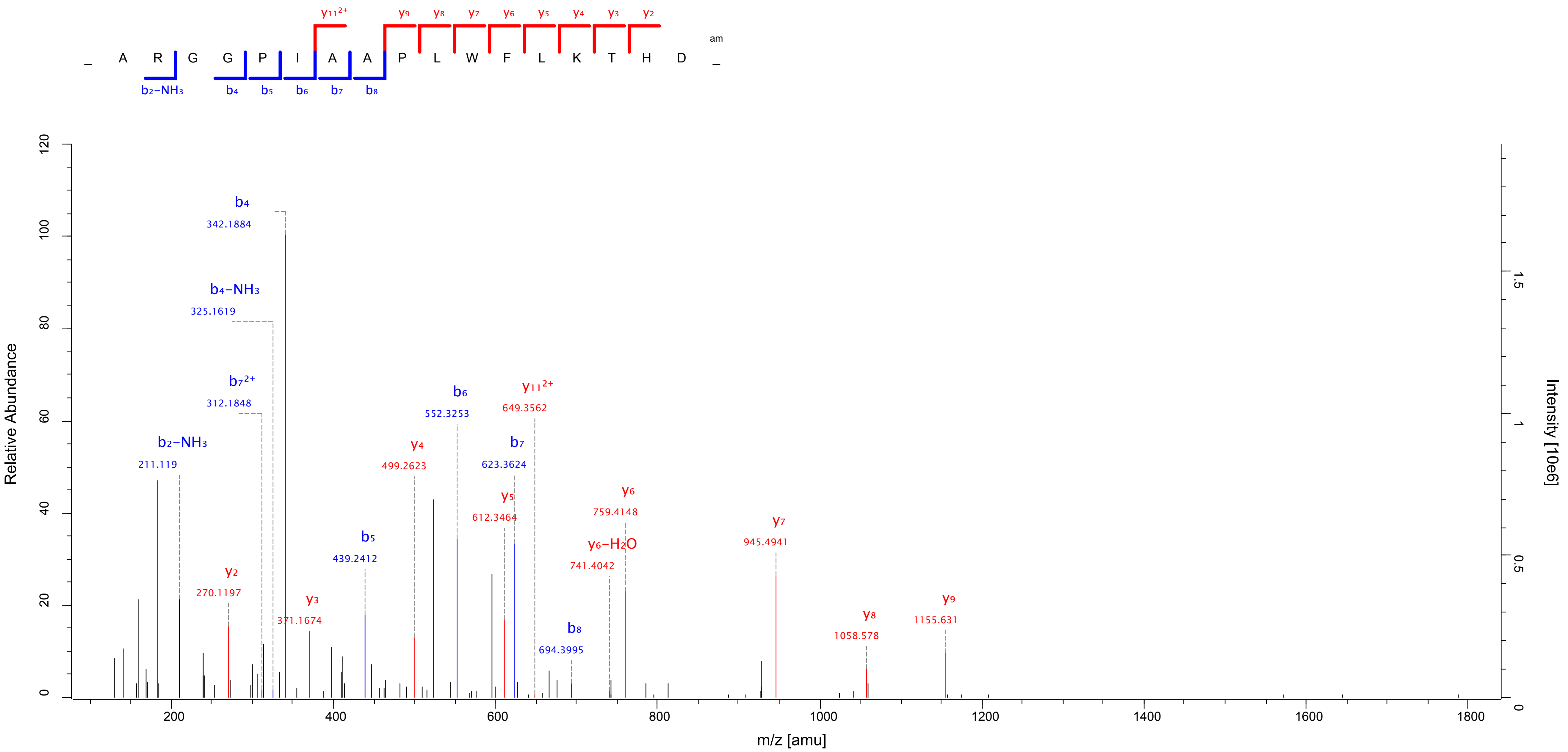

peptide ID 139

Source:	20120816_CO_0340GaJe_R02
Scannumber:	14645
Protein:	pep_secretome_4410
Peptide Score:	86.47
Method:	FTMS; HCD; 1

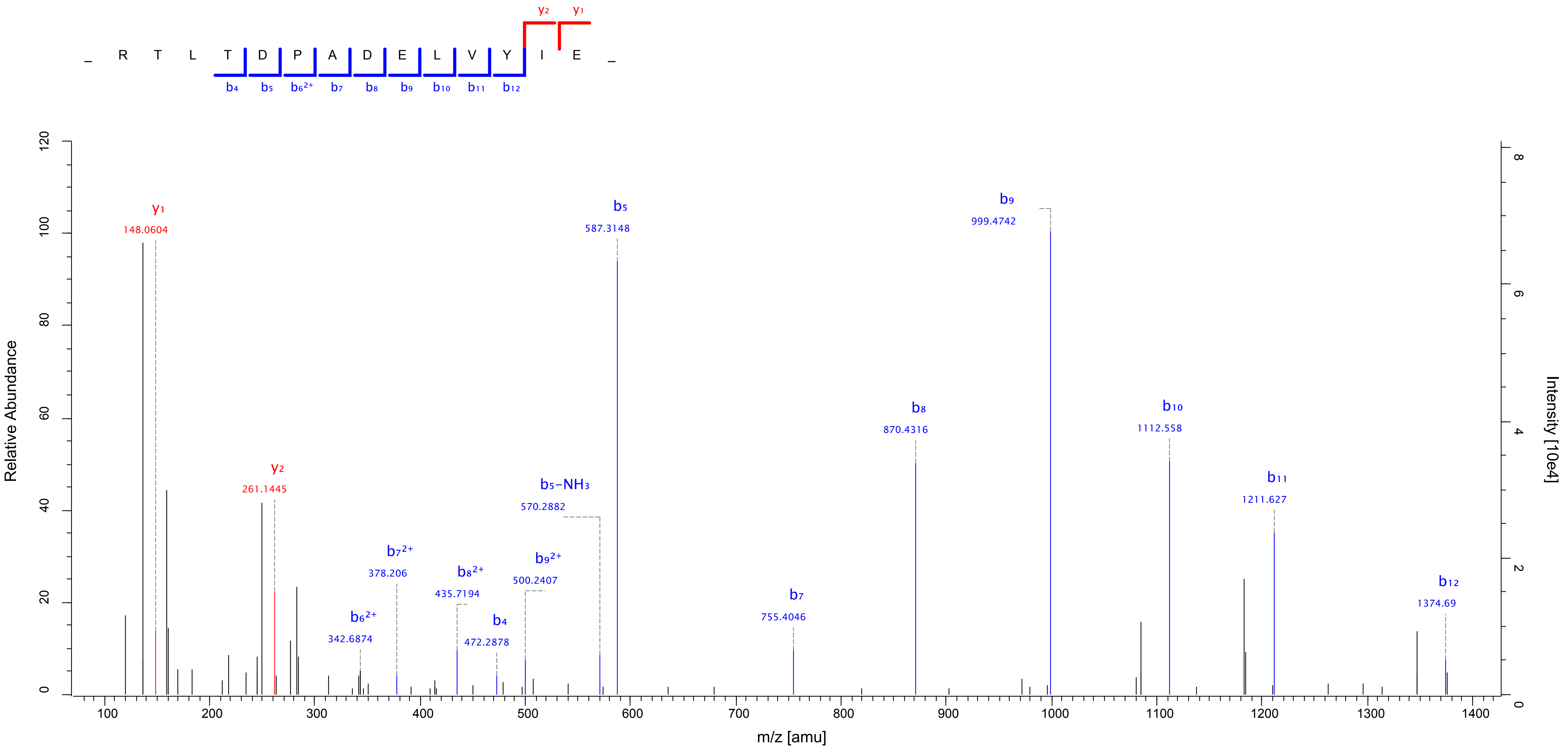

Source: 20120816_CO_0340GaJe_R02
Scannumber: 9193
Protein: pep_secretome_13893
Peptide Score: 67.7
Method: FTMS; HCD; 1

peptide ID 140

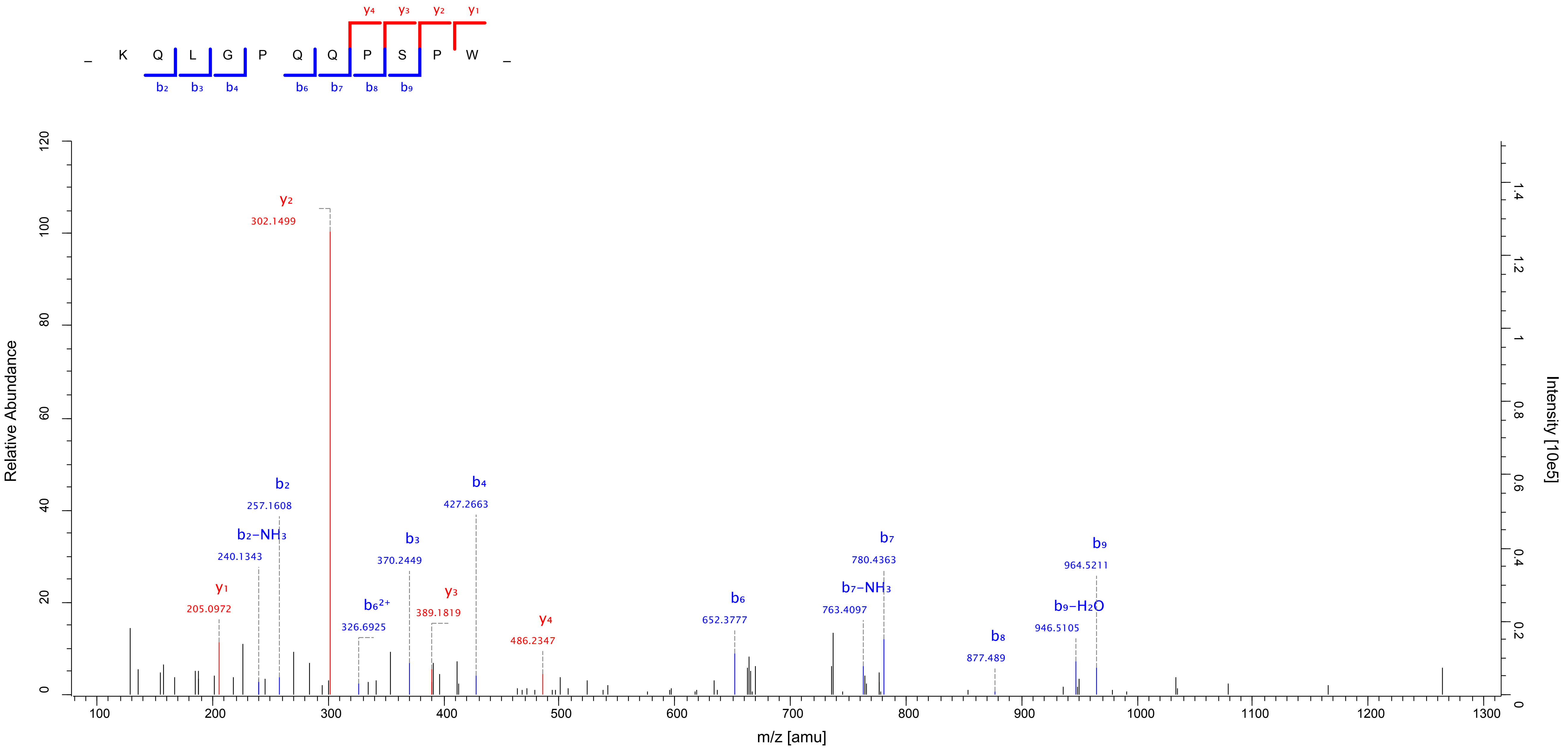

peptide ID 141

Source: 20121106_CO_0340GaJe_R02_2
Scannumber: 15102
Protein: pep_secretome_14445
Peptide Score: 116.96
Method: FTMS; HCD; 1

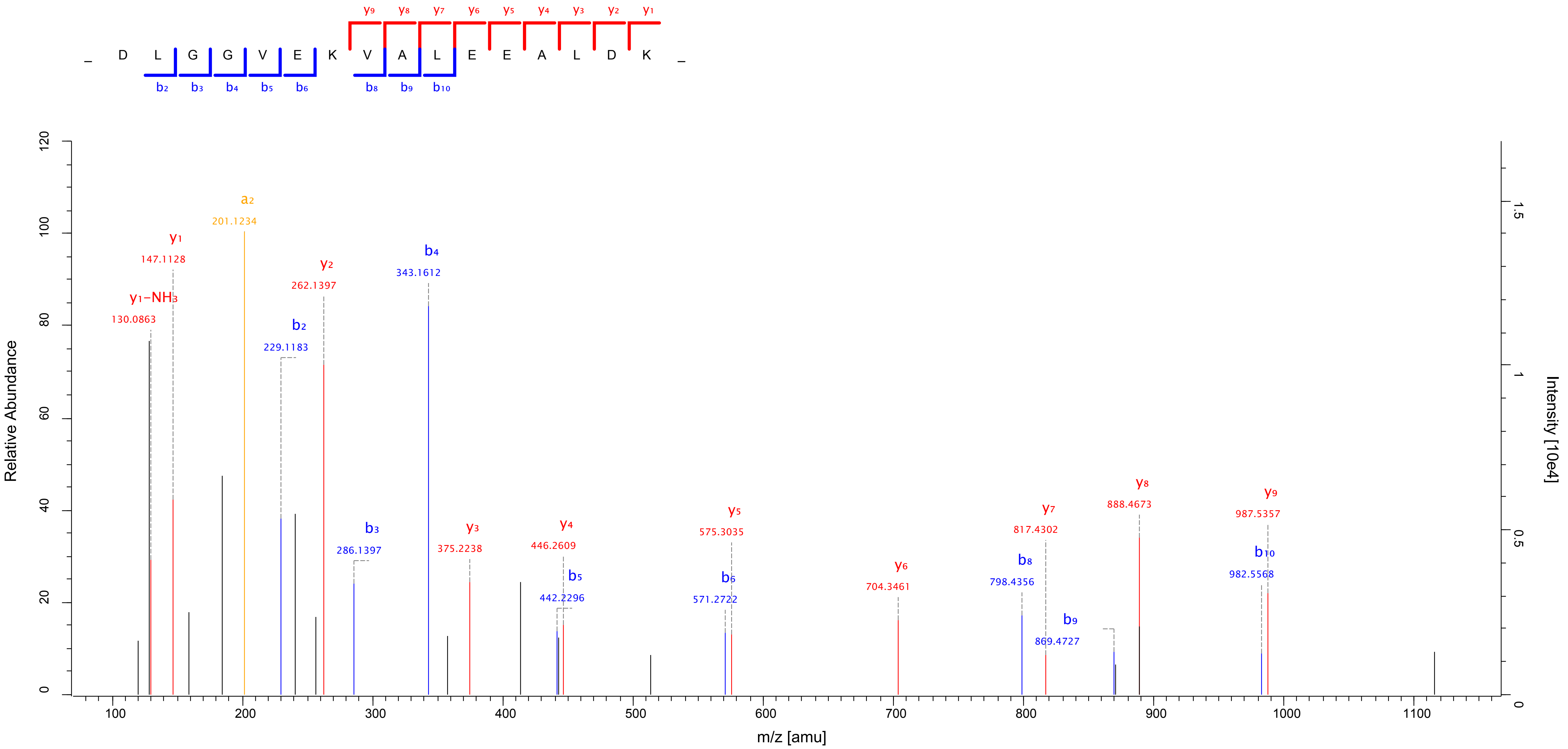

peptide ID 142

Source: 20120816_CO_0340GaJe_R02
Scannumber: 9217
Protein: pep_secretome_15969
Peptide Score: 110.84
Method: FTMS; HCD; 1

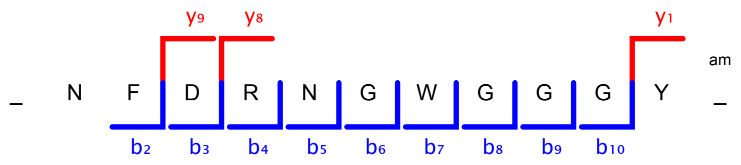

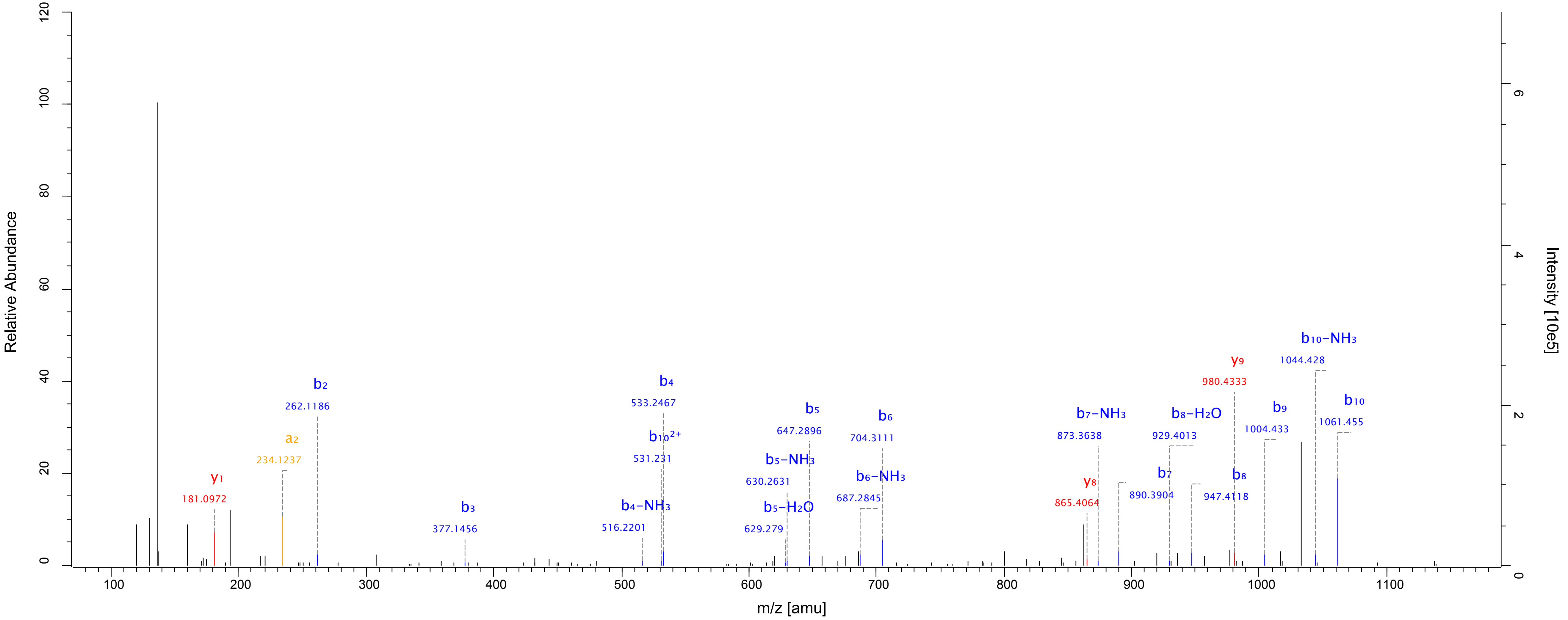

peptide ID 143

Source: 20120816_CO_0340GaJe_R02
Scannumber: 8377
Protein: pep_secretome_15047
Peptide Score: 102.4
Method: FTMS; HCD; 1

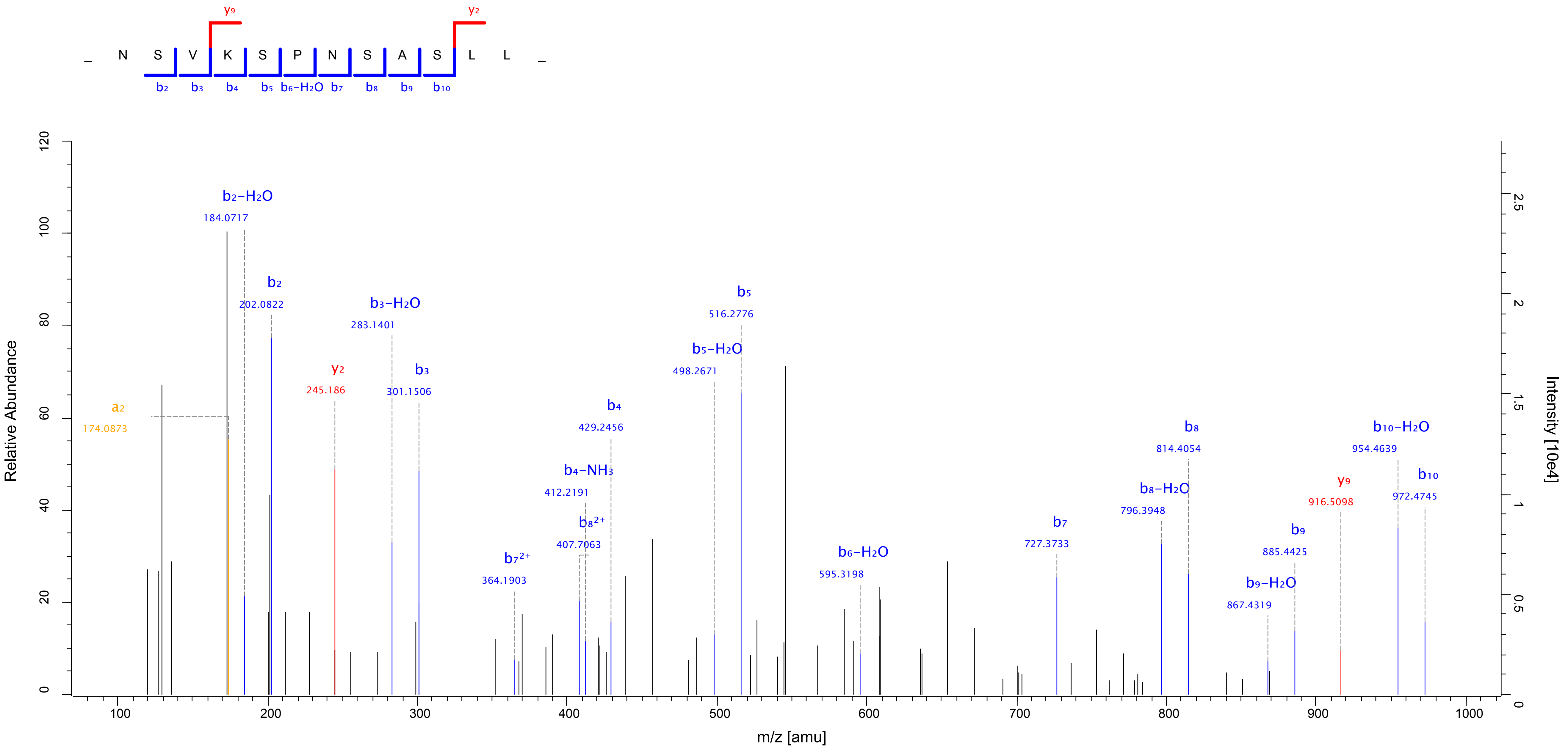

Source: 20120515_CO_0340GaJe_R01
Scannumber: 15220
Protein: pep_secretome_2821; pep_secretome_34663
Peptide Score: 104.52
Method: FTMS; HCD; 1

peptide ID 144

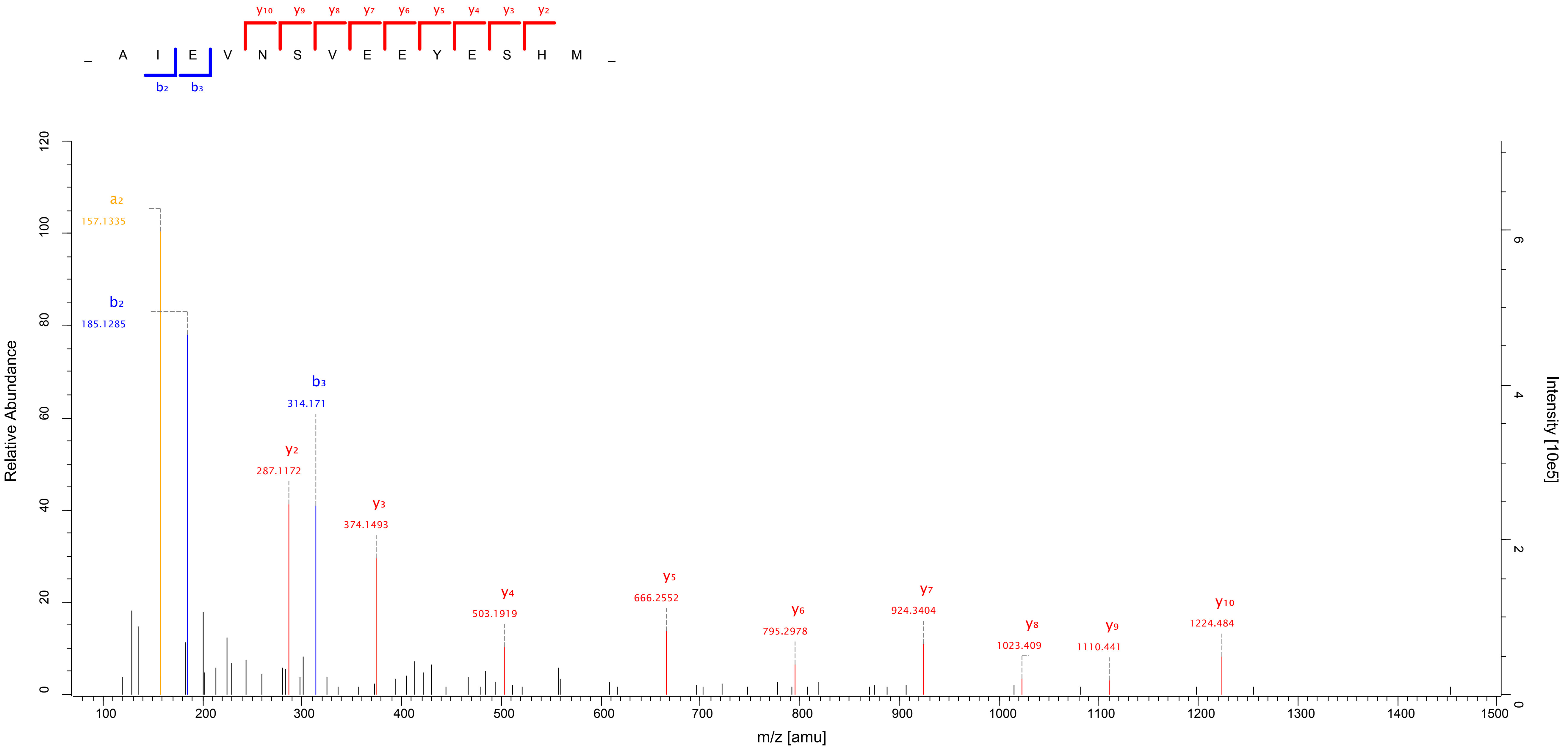

Source: 20121106_CO_0340GaJe_R02_2
Scannumber: 9377
Protein: pep_secretome_2821; pep_secretome_34663
Peptide Score: 70.49
Method: FTMS; HCD; 1

peptide ID 145

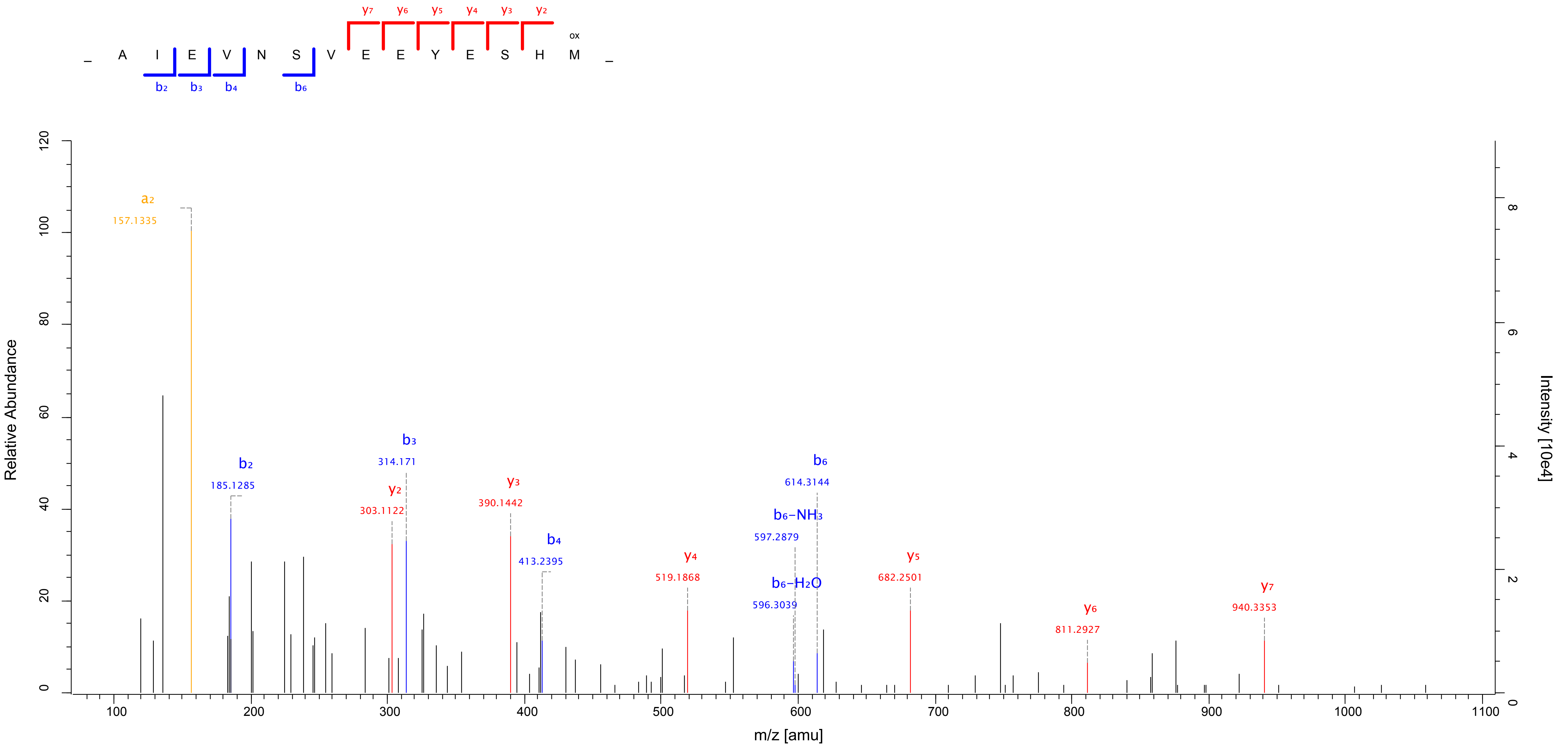

peptide ID 146

Source: 20120515_CO_0340GaJe_R01
Scannumber: 21945
Protein: pep_secretome_20173
Peptide Score: 70.37
Method: FTMS; HCD; 1

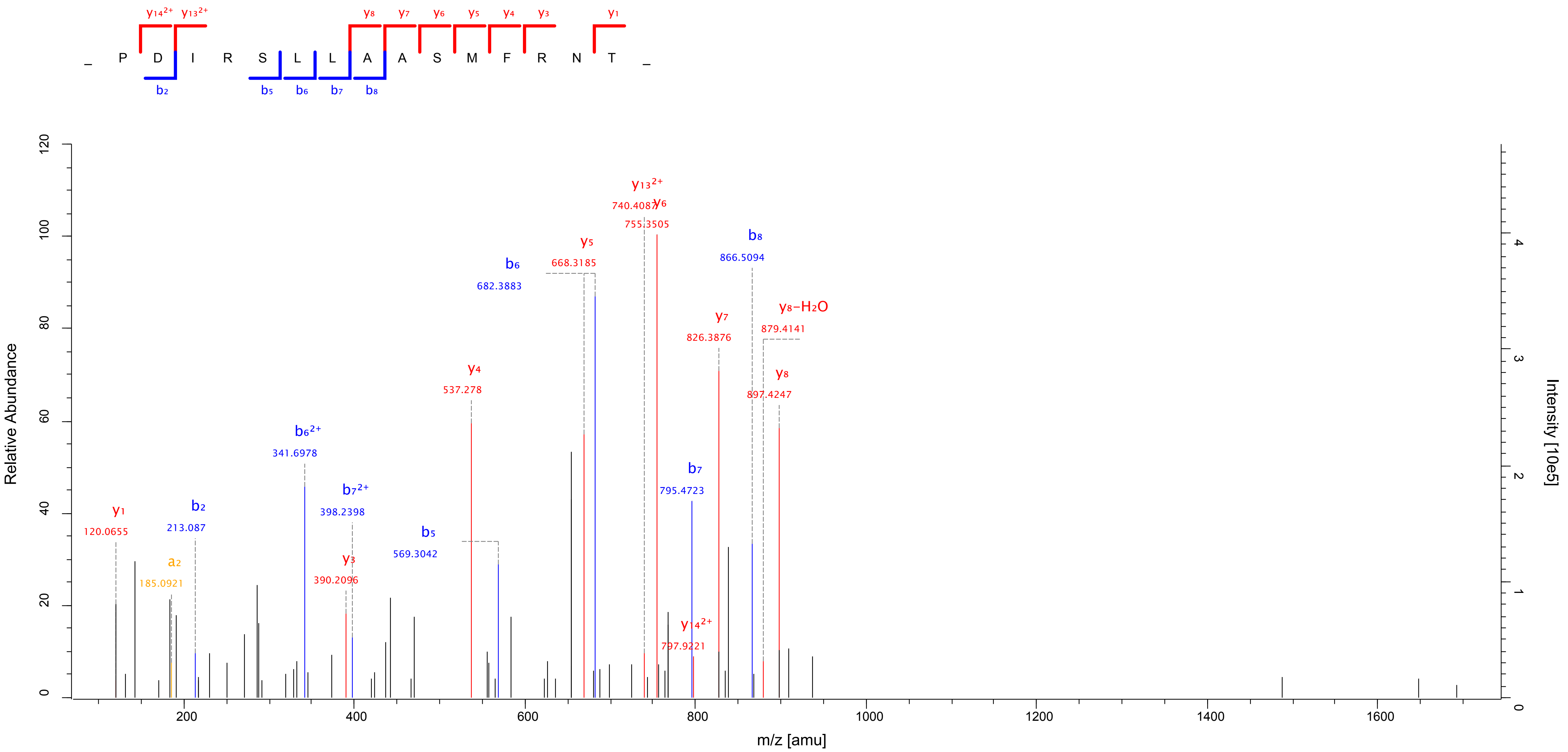

Supplement: Additional file 9 — Mass-spectrometry hits obtained by the analysis of Platynereis peptide extracts with the corresponding spectra for each hit, in portable document format. [file 1471-2164-14-906-S9.pdf]
