# Supplementary material for: The neuropeptide complement of the marine annelid Platynereis dumerilii
Source: BMC Genomics. 2013 Dec 20;14:906. doi: 10.1186/1471-2164-14-906 (PMC3890597; doi:10.1186/1471-2164-14-906)
Supplement: Additional file 10 — Comprehensive list of multiple sequence alignments of Platynereis pNPs with homologous sequences, in portable document format. [file 1471-2164-14-906-S10.pdf]

Additional file 10: list of multiple sequence alignments

Platynereis pNPs belonging to eumetazoan pNPs families:

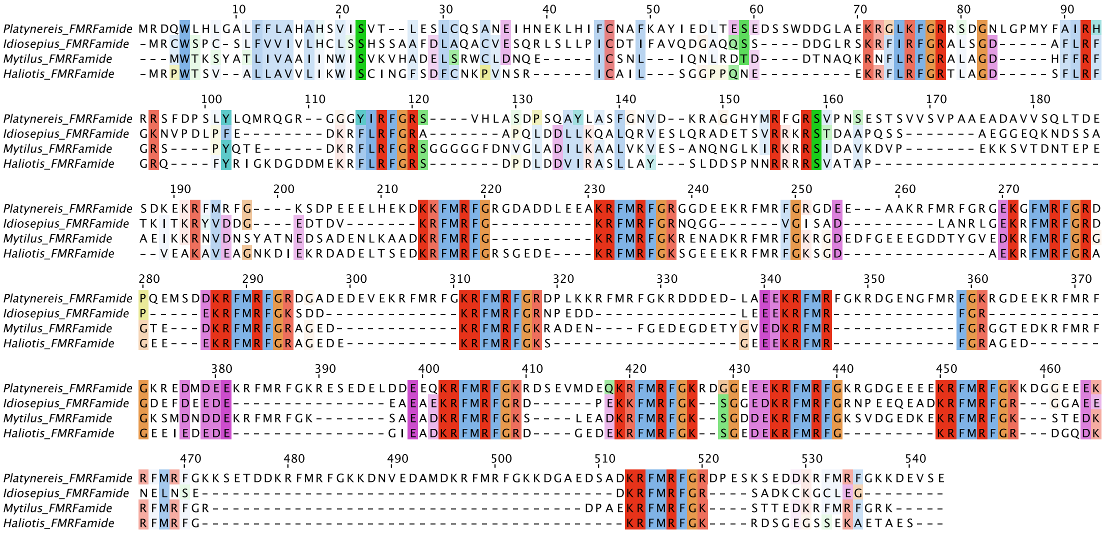

Multiple sequence alignment of annelid and mollusk FMRFa pNPs.

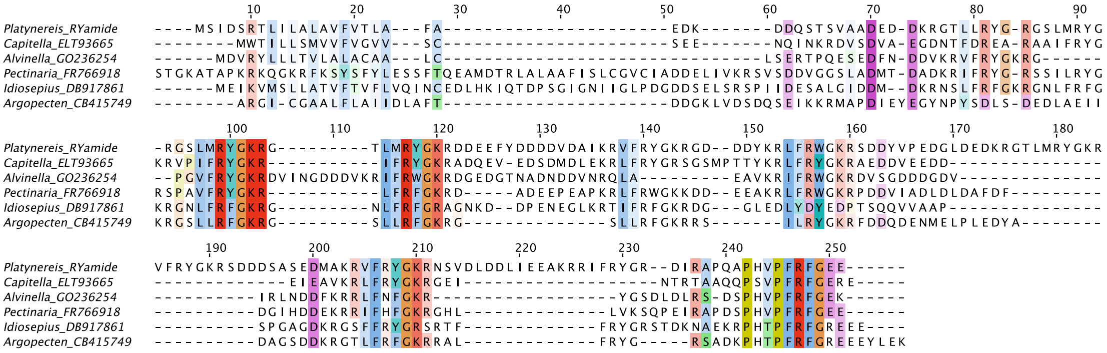

Multiple sequence alignment of annelid and mollusk RYa pNPs. These pNPs also share a conserved peptide in the C-terminus of the precursor, potentially yielding Pro rich RFamide-related peptides.

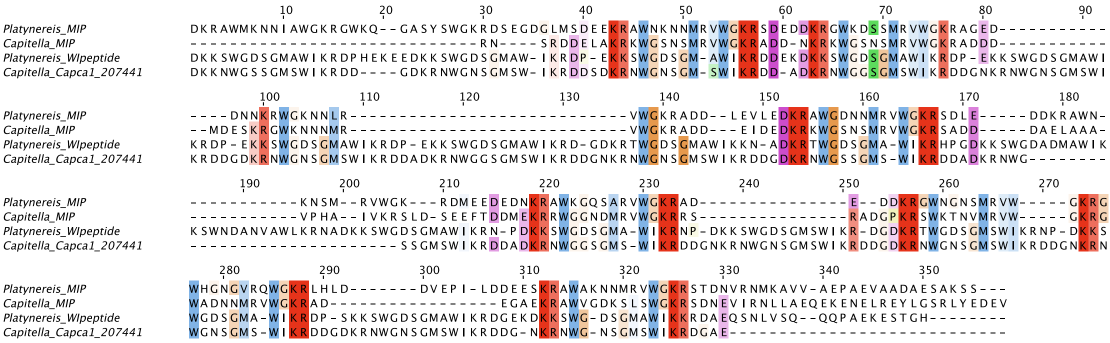

Multiple sequence alignment of trimmed *Platynereis* and *Capitella teleta* WI peptide and MIP pNPs. The mature WI peptides share two conserved Trp-residues with MIPs.

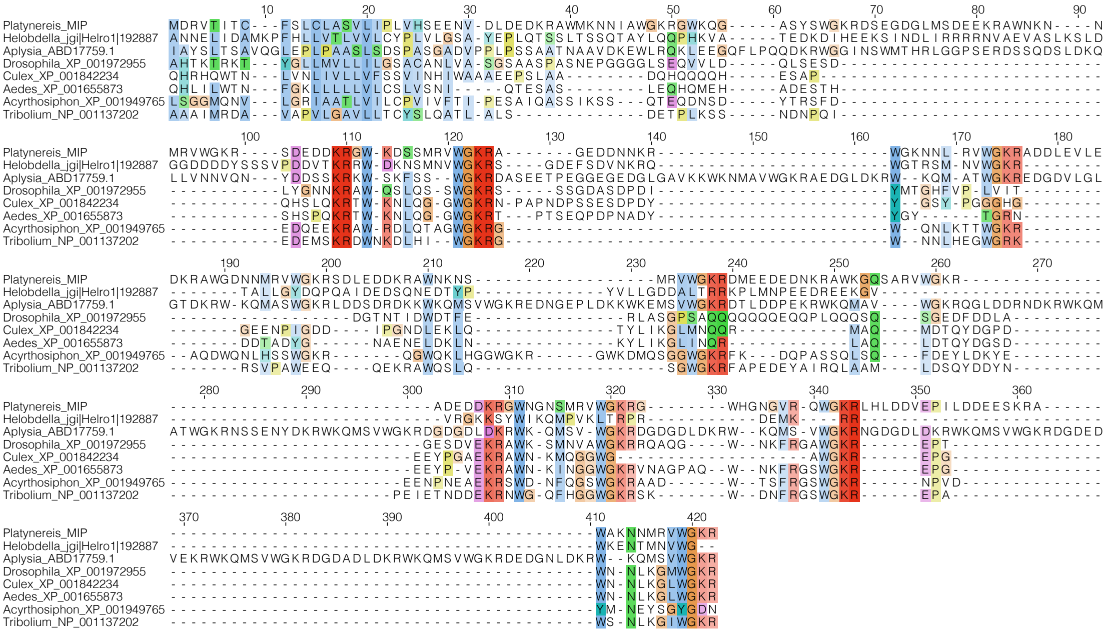

Multiple sequence alignment of *Platynereis* MIP pNP with protostome MIP/allatostatin-B pNPs.

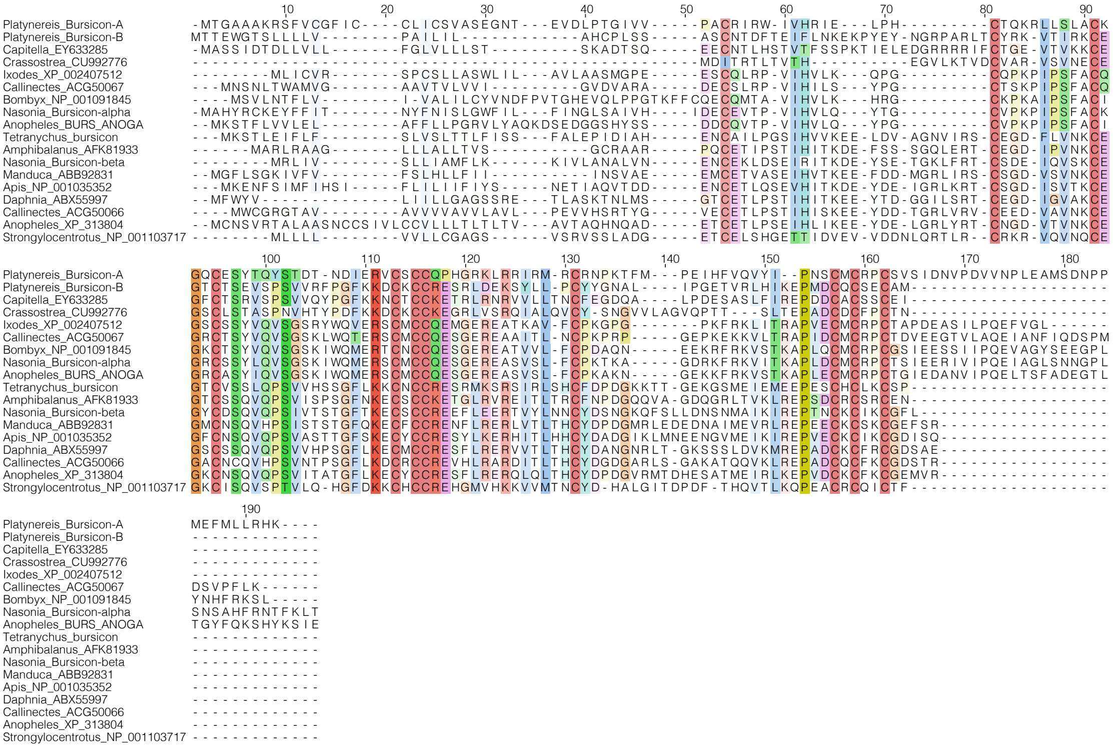

Multiple sequence alignment of *Platynereis* bursicon alpha and beta with bilaterian bursicon pNPs.

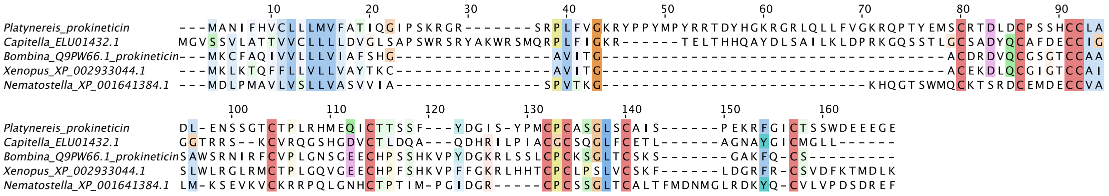

Truncated multiple sequence alignment of *Platynereis* prokineticin with bilaterian and cnidarian prokineticin pNPs. In *Platynereis* and *Capitella teleta*, this pNP also gives rise to amidated peptides located at the N-terminus of the pNP.

Platynereis pNPs belonging to bilaterian pNPs families:

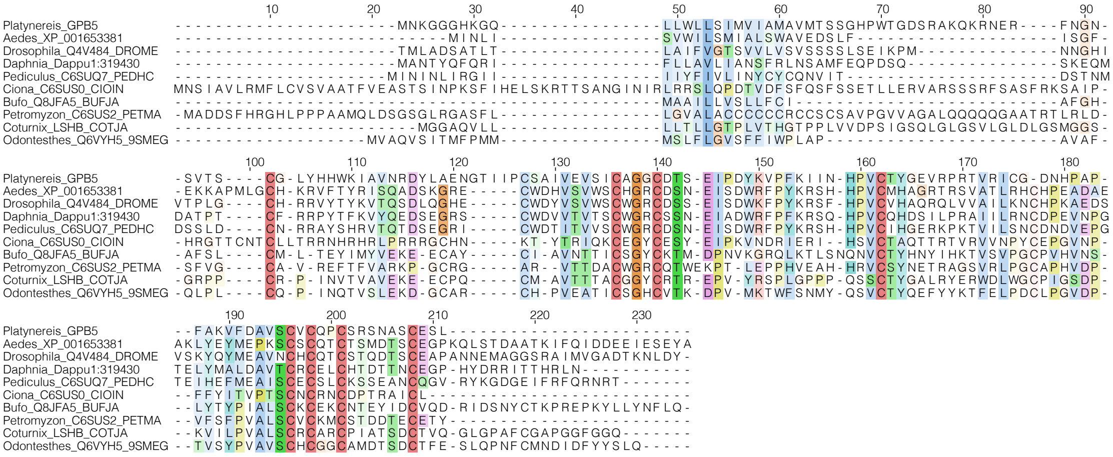

Multiple sequence alignment of *Platynereis* glycoprotein beta with bilaterian glycoprotein beta pNPs.

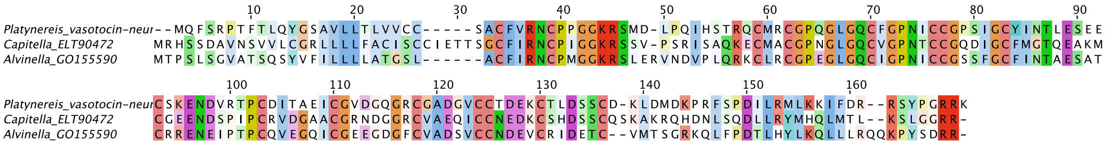

Multiple sequence alignment of annelid vasotocin-neurophysin pNPs shows a potential C-terminally located peptide (SYPamide in *Platynereis* and SLGamide in *Capitella teleta*).

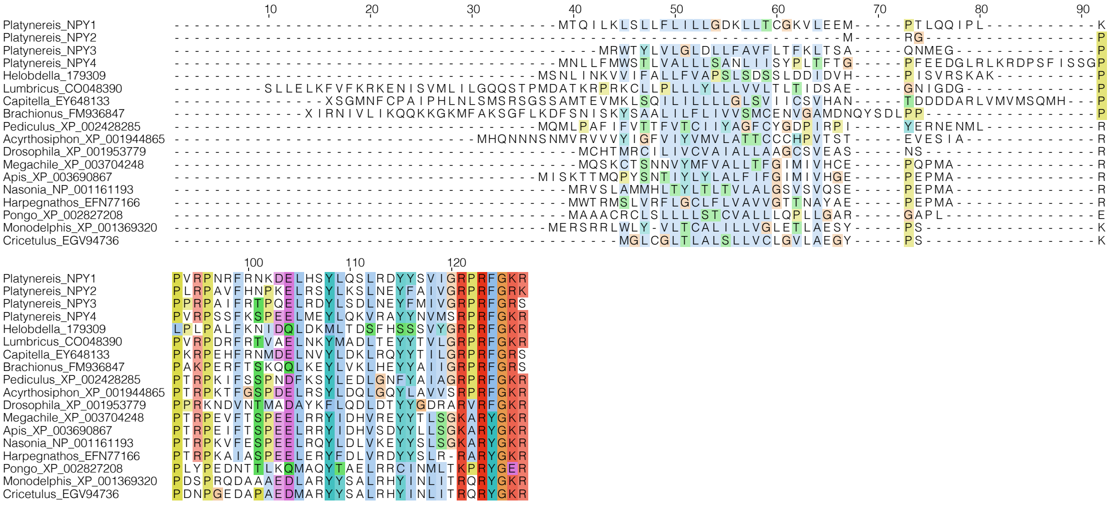

Truncated multiple sequence alignment of *Platynereis* NPY pNPs with bilaterian NPY/NPF pNPs.

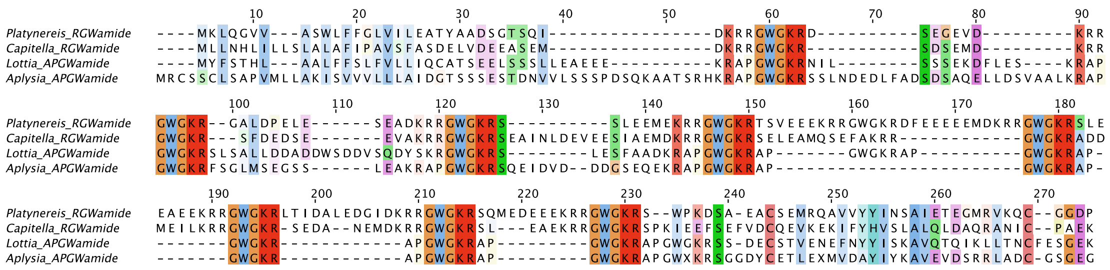

Multiple sequence alignment of annelid and mollusk RGWamide and APGWamide pNPs. These precursors share a Cys-containing stretch at the C-terminus of the precursor.

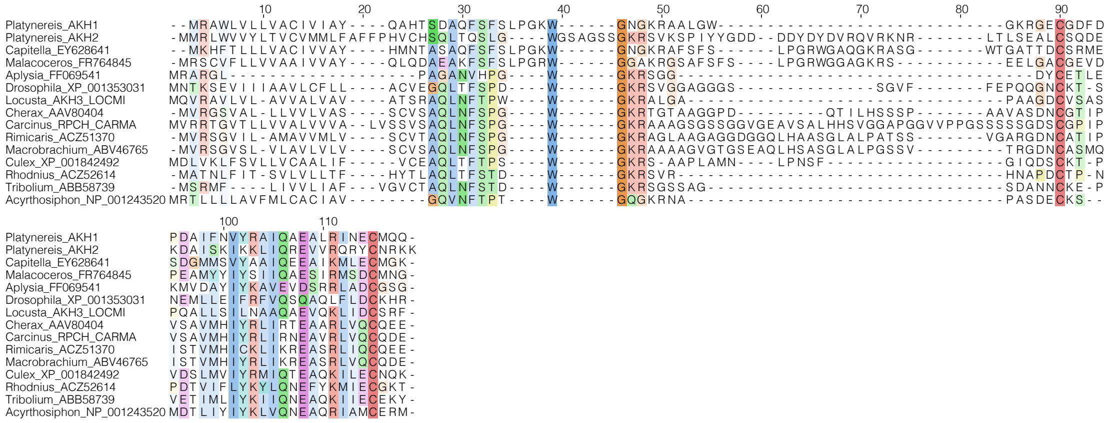

Truncated multiple sequence alignment of *Platynereis* AKH pNPs with protostome AKH/RPCH pNPs.

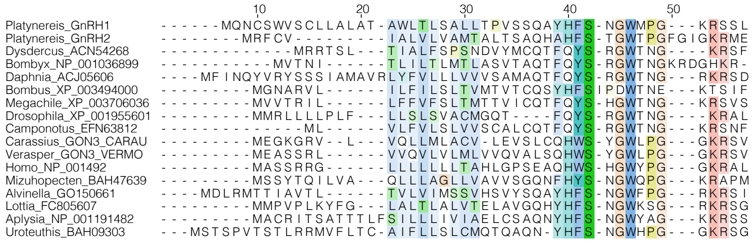

Truncated multiple sequence alignment of *Platynereis* GnRH pNPs with bilaterian GnRH/corazonin pNPs.

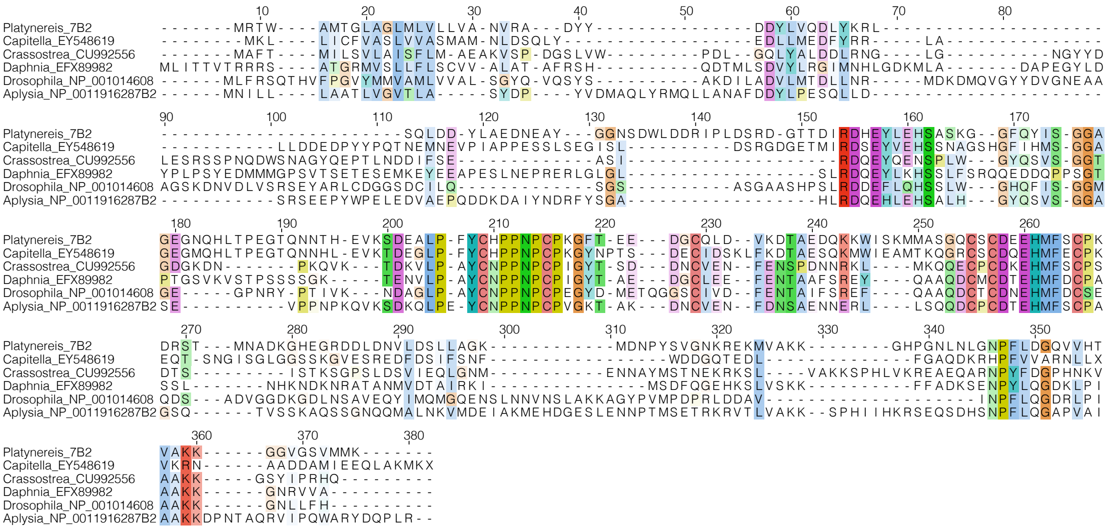

Multiple sequence alignment of *Platynereis* 7B2 pNP with protostome 7B2 pNPs.

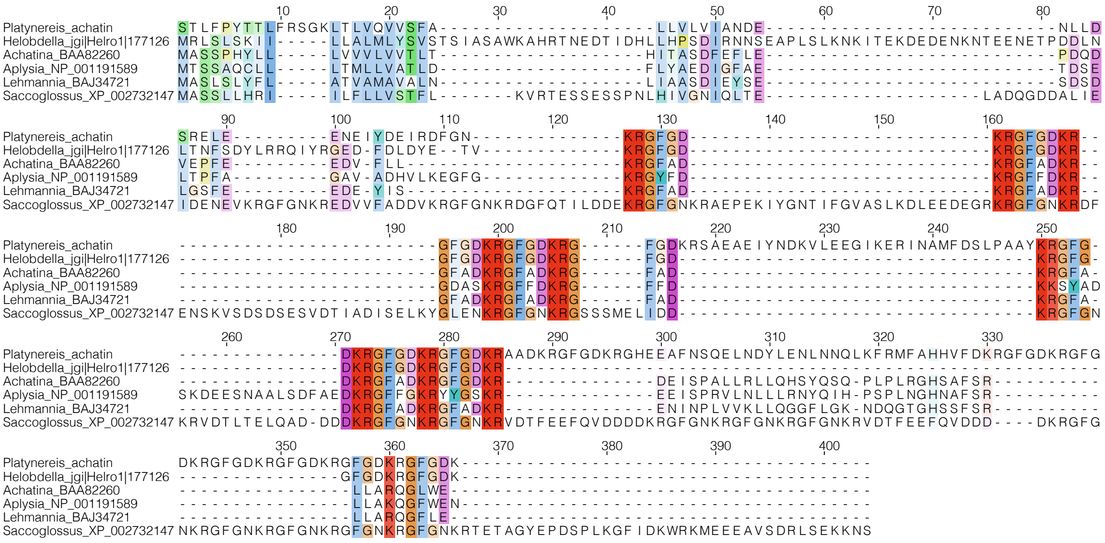

Multiple sequence alignment of *Platynereis* achatin pNP with bilaterian achatin pNPs.

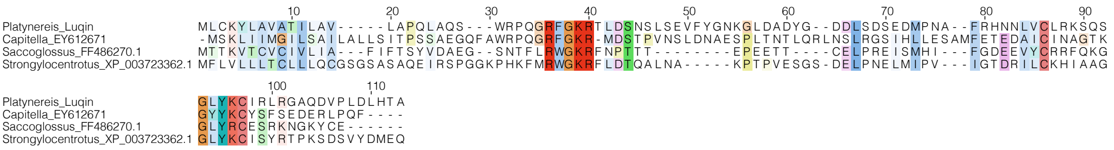

Multiple sequence alignment of *Platynereis* luqin pNP with bilaterian luqin pNPs showing the conservation of a small amidated peptide and a C-terminal Cys-containing peptide.

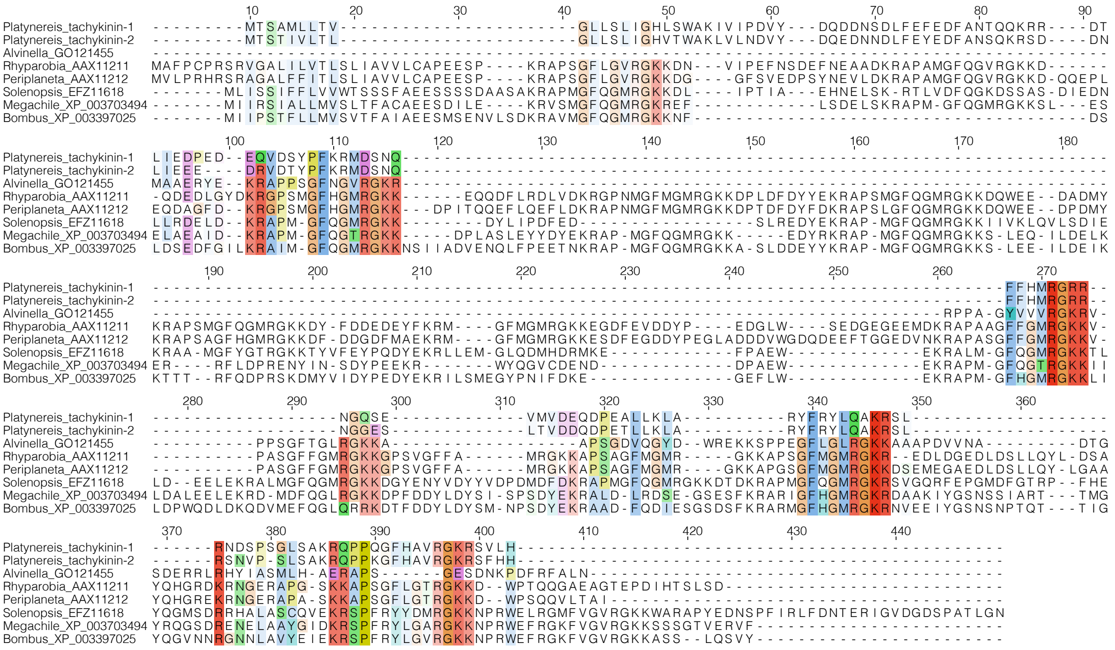

Multiple sequence alignment of *Platynereis* tachykinin pNPs with protostome tachykinin pNPs. Deuterostome sequences were not included because of low sequence identity.

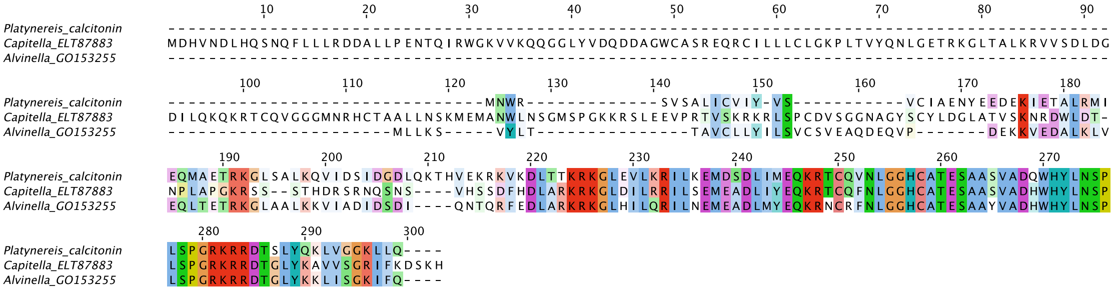

Multiple sequence alignment of annelid calcitonin pNPs shows an additional conserved C-terminal amidated peptide (e.g., DTSLYQKLVGamide in *Platynereis*).

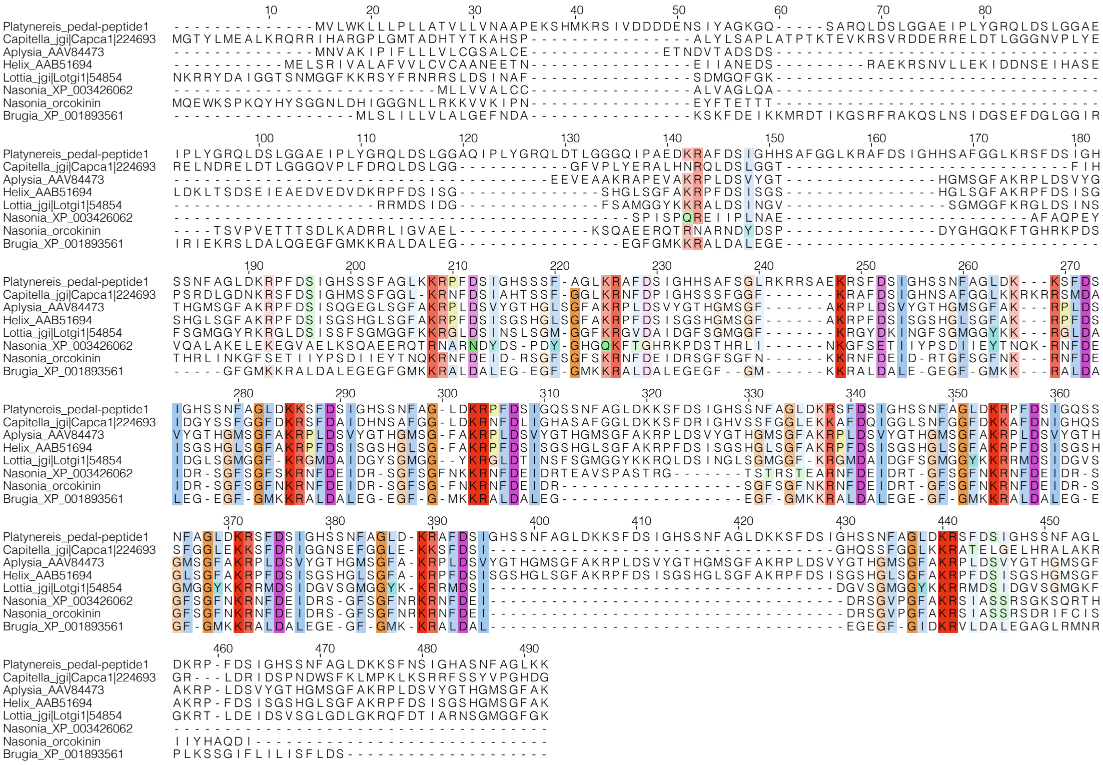

Truncated multiple sequence alignment of *Platynereis* pedal-peptide-1 pNP with protostome pedal-peptide-1 and orcokinin pNPs.

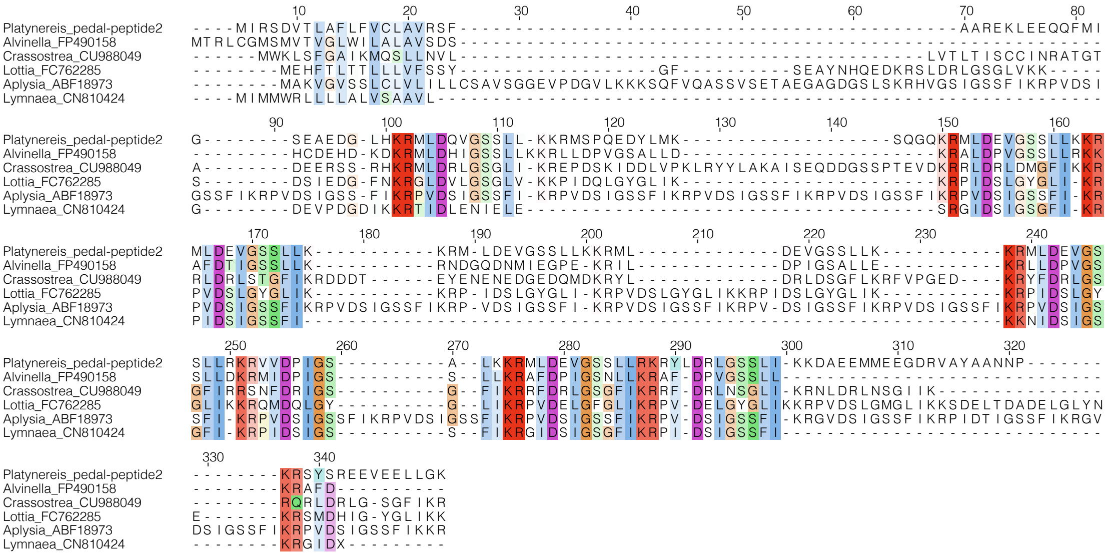

Truncated multiple sequence alignment of *Platynereis* pedal-peptide-2 pNP with annelid and mollusk pedal-peptide-2 pNPs.

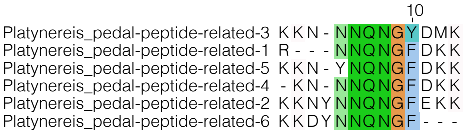

Multiple sequence alignment of *Platynereis* pedal-peptide-related peptides.

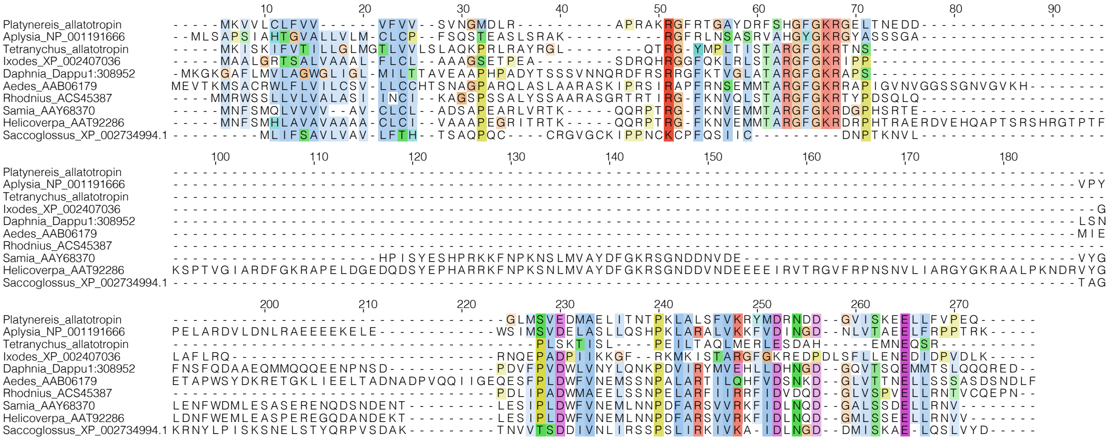

Multiple sequence alignment of *Platynereis* allatotropin pNP with bilaterian allatotropin pNPs. The *Platynereis* pNP shares an N-terminal GF/Yamide peptide with protostome pNPs, and a C-terminal conserved cryptic peptide with protostome and *Saccoglossus* pNPs.

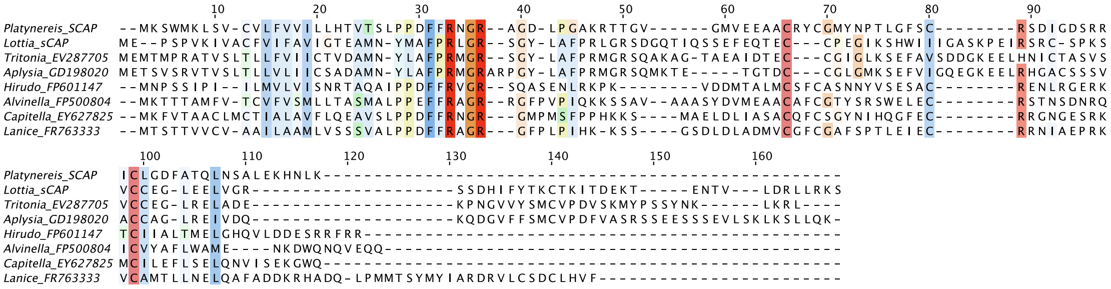

Multiple sequence alignment of annelid and mollusk pyrokinin-related sCAP pNPs.

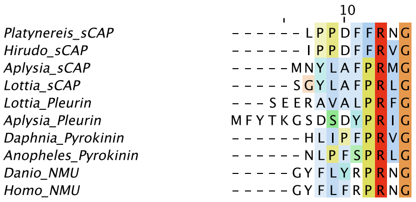

Multiple sequence alignment of bilaterian pyrokinin-related peptides, including annelid/mollusk sCAP, mollusk pleurin, insect pyrokinins and vertebrate neuromedin-U.

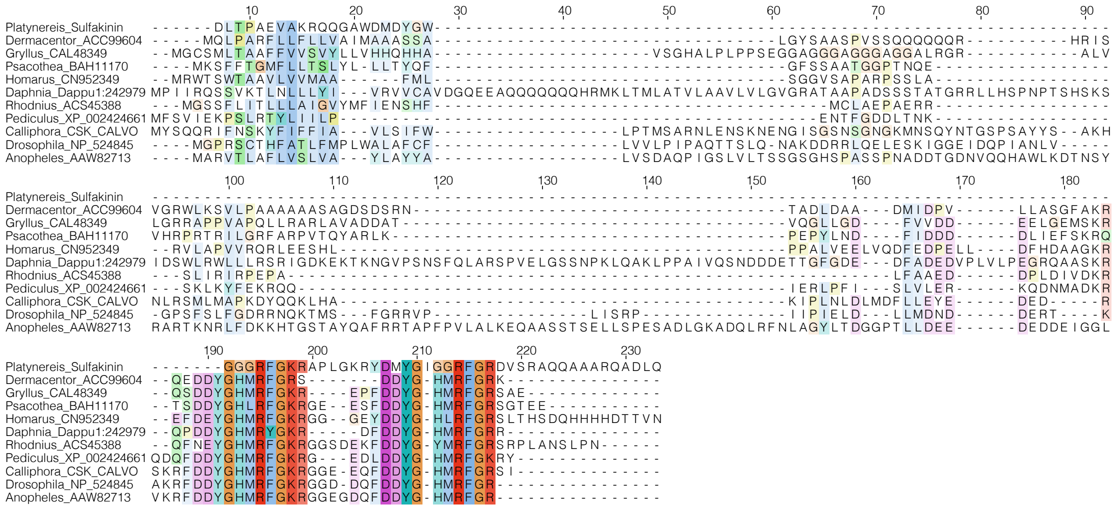

Multiple sequence alignment of *Platynereis* sulfakinin pNP with arthropod sulfakinin pNPs.

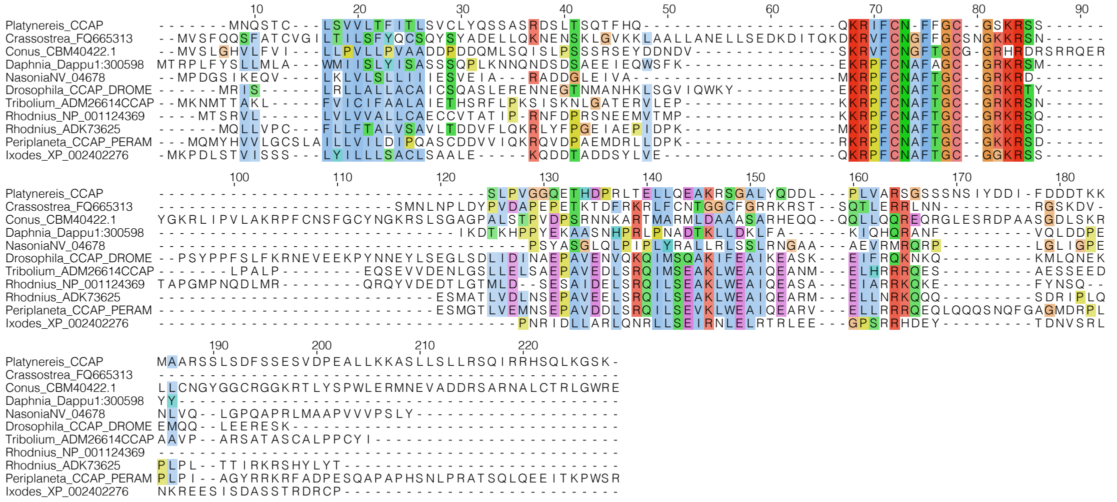

Multiple sequence alignment of *Platynereis* CCAP pNP with protostome CCAP pNPs.

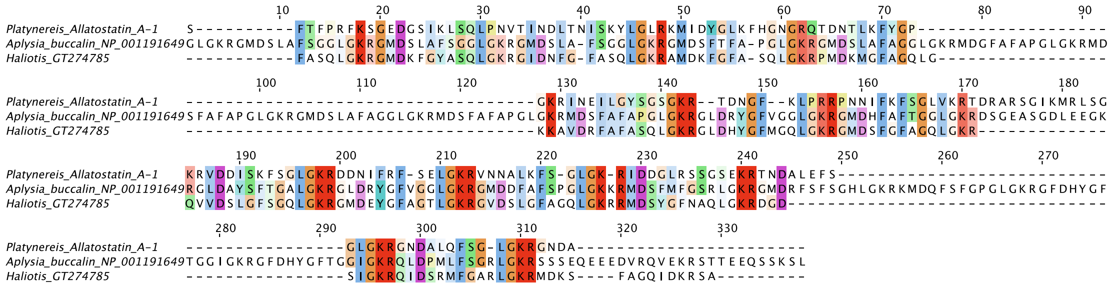

Multiple sequence alignment of trimmed *Platynereis* allatostatin-A-2 pNPs with mollusk allatostatin-A-2 pNPs.

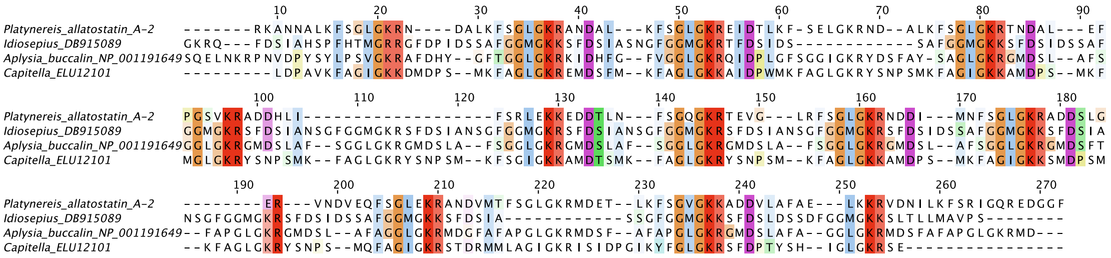

Multiple sequence alignment of trimmed annelid and mollusk allatostatin-A-2 pNPs.

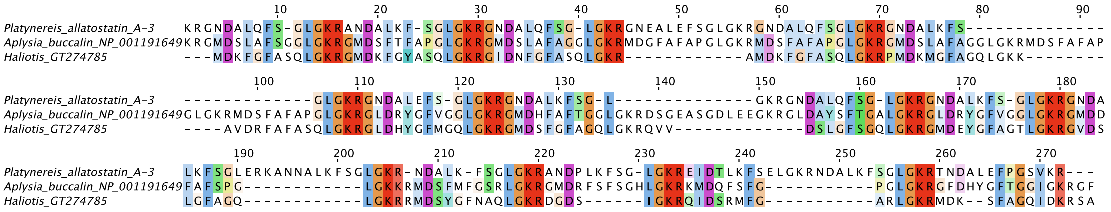

Multiple sequence alignment of trimmed annelid and mollusk allatostatin-A-3 pNPs.

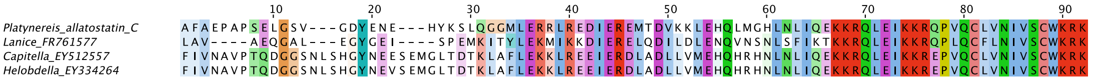

Multiple sequence alignment of trimmed annelid allatostatin-C pNPs. Besides the Cys-containing allatostatin-C peptide, these pNPs share other conserved peptide stretches (e.g., QLEI peptides).

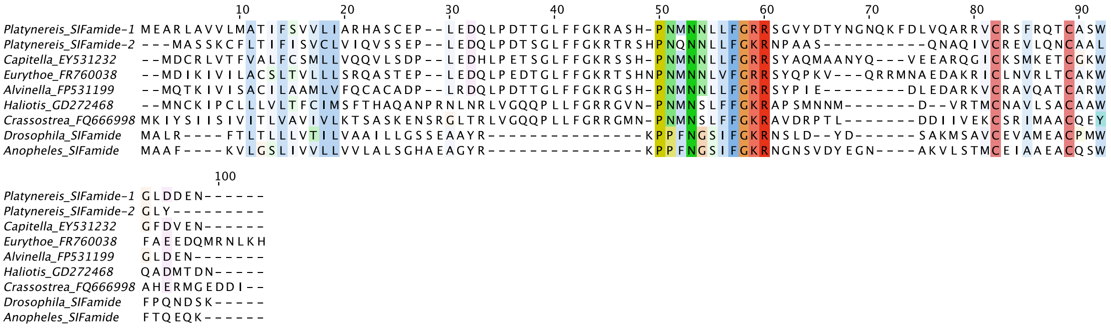

Multiple sequence alignment of lophotrochozoan FFamide and arthropod SIFamide pNPs shows besides a conserved amidated peptide conserved cystein-residues in the C-terminus of the pNPs.

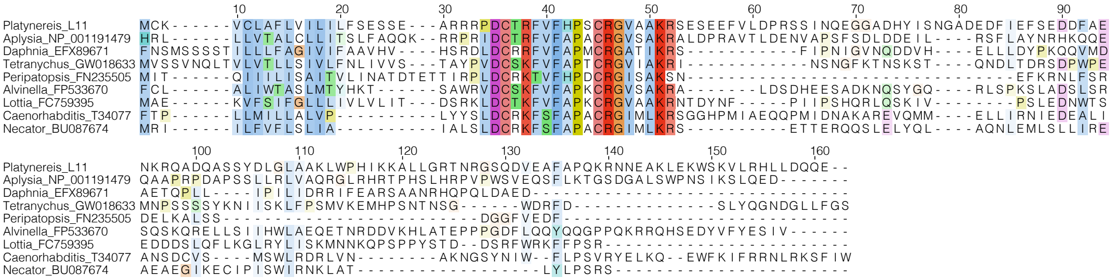

Multiple sequence alignment of *Platynereis* L11 pNP with protostome L11 pNPs.

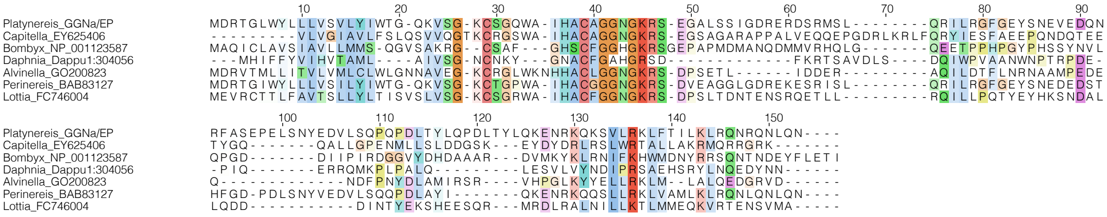

Multiple sequence alignment of *Platynereis* GGNa/excitatory peptide pNP with protostome GGNa/ excitatory-peptide/CCHa pNPs.

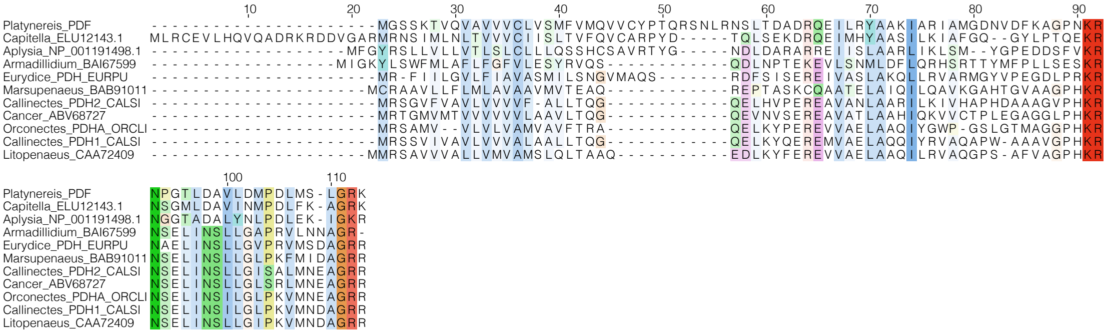

Multiple sequence alignment of *Platynereis* pdf pNP with protostome pdf/cerebrin pNPs.

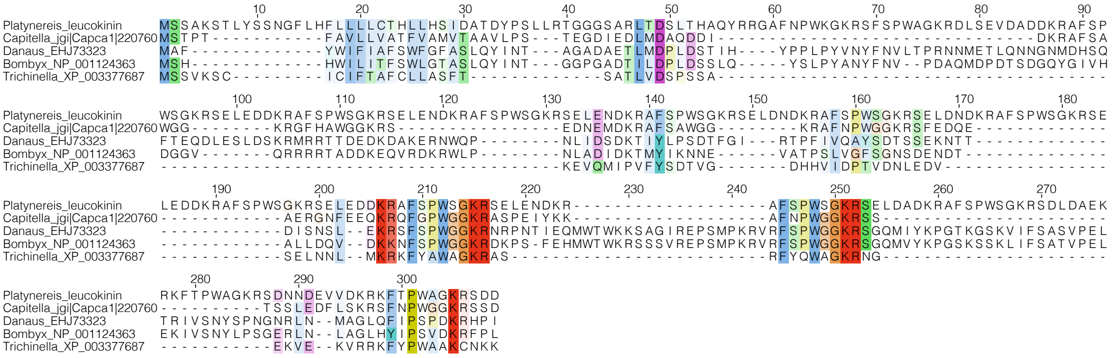

Truncated multiple sequence alignment of *Platynereis* leucokinin pNP with protostome leucokinin pNPs.

Platynereis pNPs belonging to protostome pNPs families:

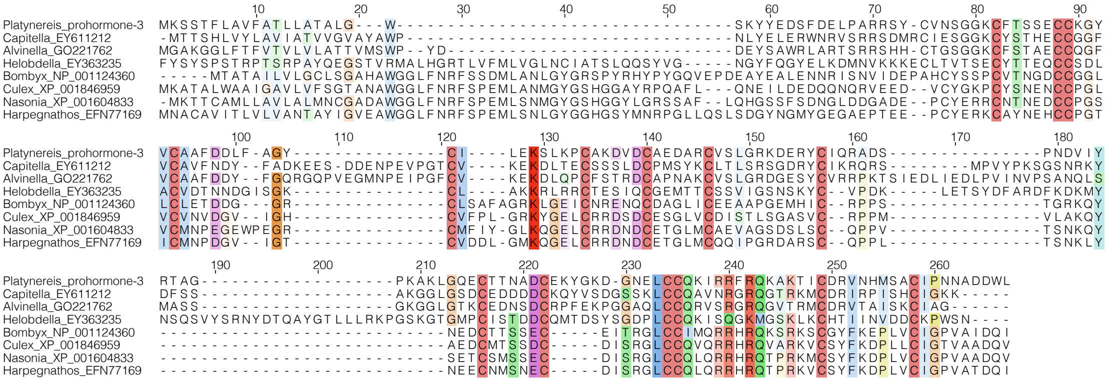

Multiple sequence alignment of *Platynereis* prohormone-3 pNP with protostome prohormone-3 pNPs.

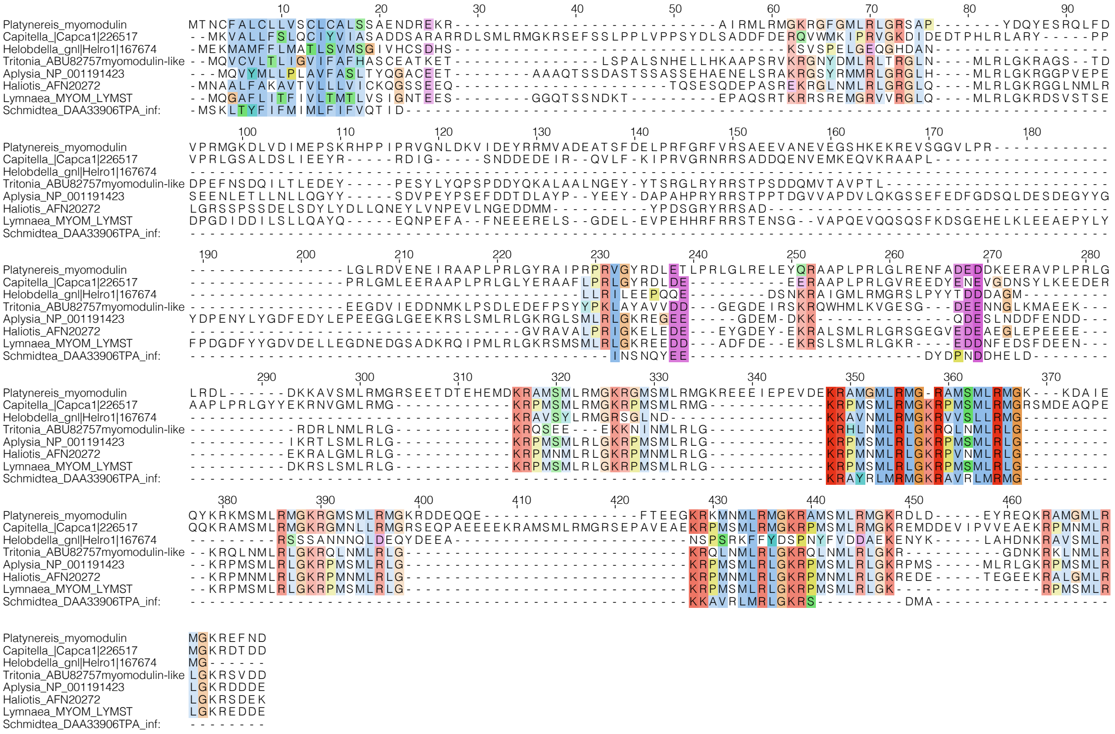

Multiple sequence alignment of truncated *Platynereis* myomodulin pNP with lophotrochozoan myomodulin pNPs.

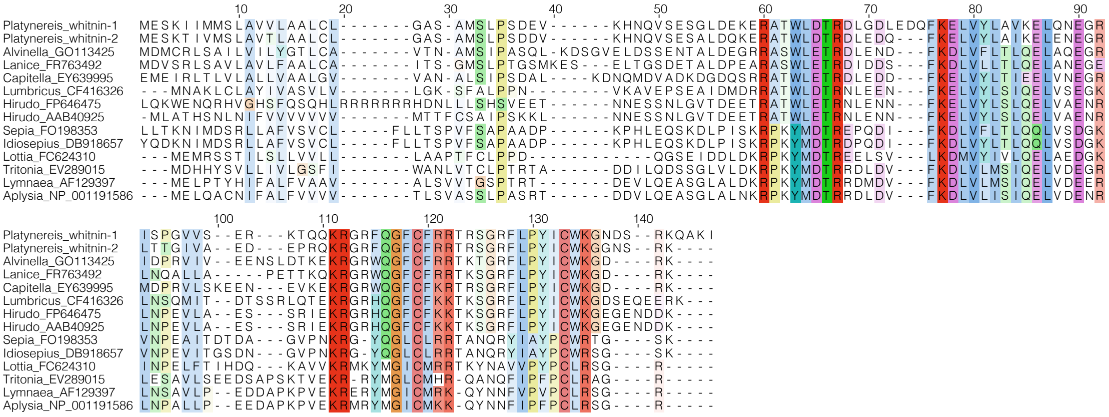

Multiple sequence alignment of *Platynereis* whitnin with annelid and mollusk whitnin/SPTR pNPs.

Platynereis pNPs belonging to lophotrochozoan pNPs families:

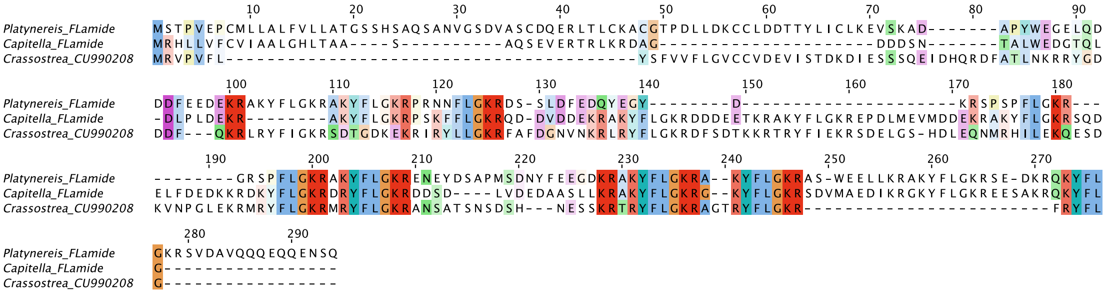

Multiple sequence alignment of annelid and mollusk FLamide/Fulicin pNPs.

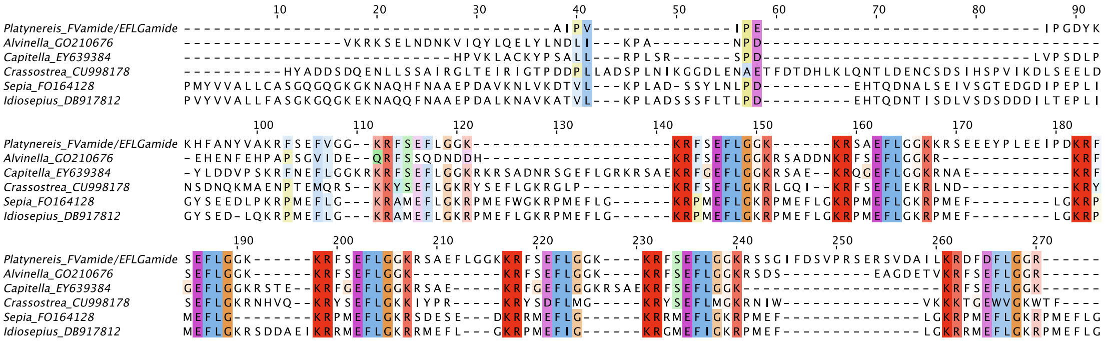

Multiple sequence alignment of annelid and mollusk EFLGamide/EFLamide containing ESTs.

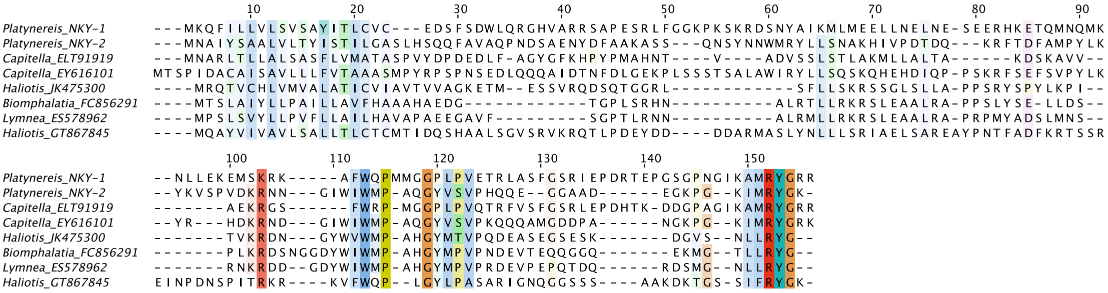

Multiple sequence alignment of annelid and mollusk NKY pNPs.

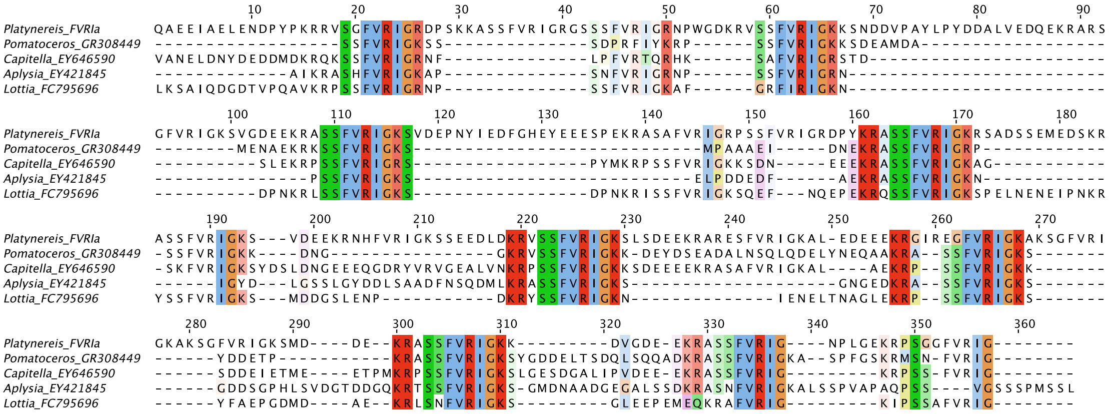

Multiple sequence alignment of trimmed annelid and mollusk FVRIamide pNPs.

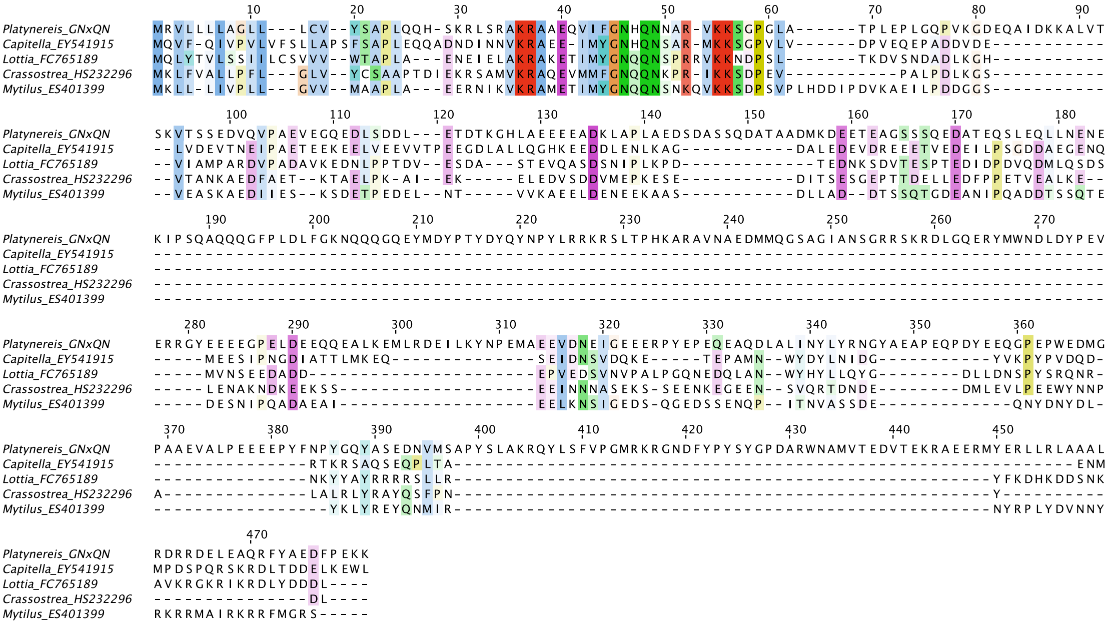

Multiple sequence alignment of annelid and mollusk GNXQN peptide pNPs.

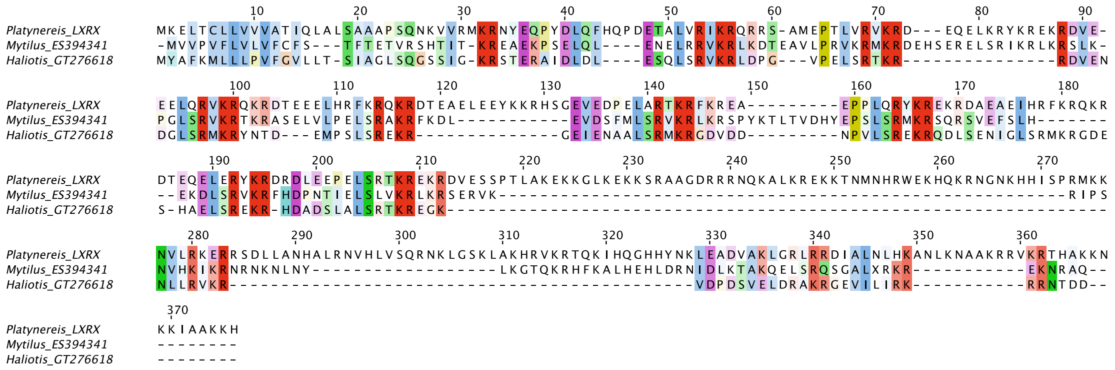

Multiple sequence alignment of annelid and mollusk LXRX peptide pNPs.

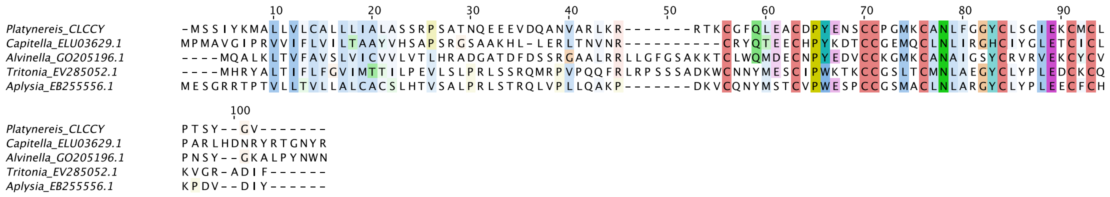

Multiple sequence alignment of annelid and mollusk CLCCY pNPs.

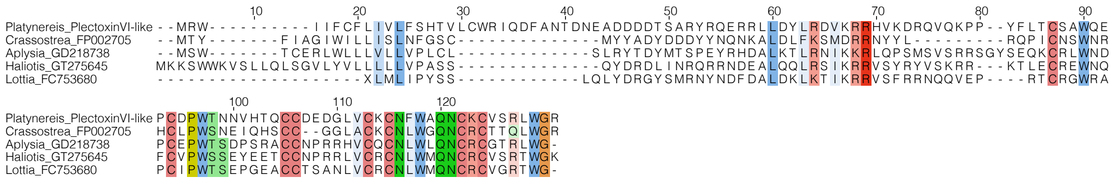

Multiple sequence alignment of annelid and mollusk CCWamide pNPs.

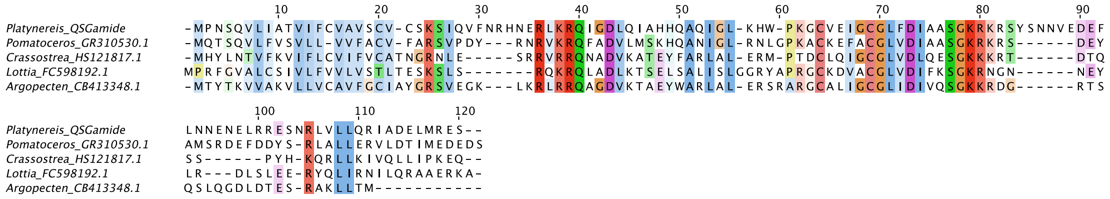

Multiple sequence alignment of annelid and mollusk QSGamide pNPs.

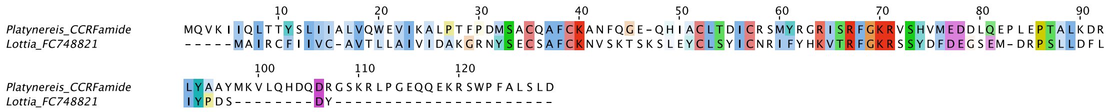

Multiple sequence alignment of *Platynereis* and *Lottia gigantea* CCRFamide pNPs.

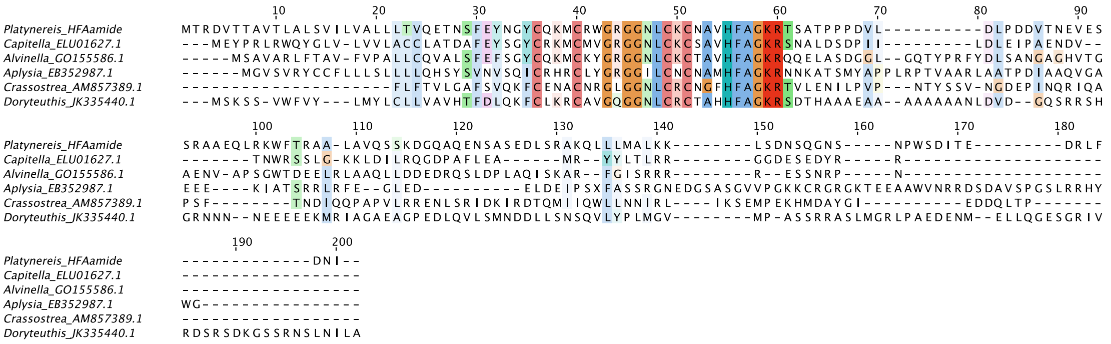

Multiple sequence alignment of annelid and mollusk HFAamide pNPs.

Platynereis pNPs belonging to annelid pNPs families:

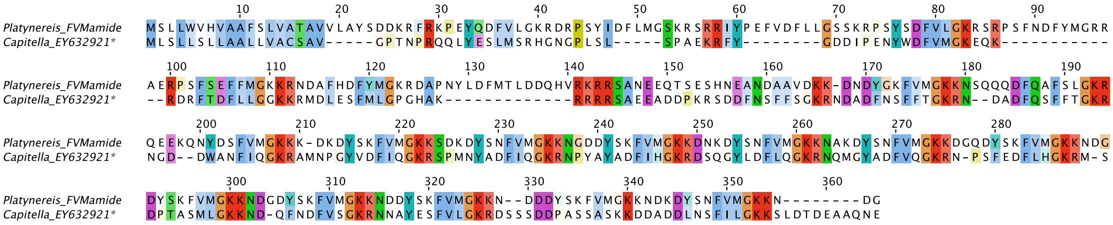

Multiple sequence alignment of annelid FVMamide pNPs.

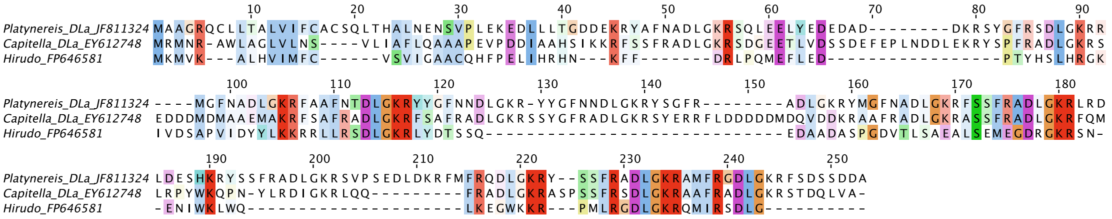

Multiple sequence alignment of annelid DLamide pNPs.

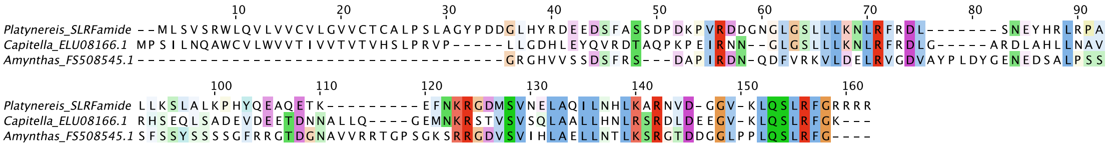

Multiple sequence alignment of annelid SLRFamide pNPs.

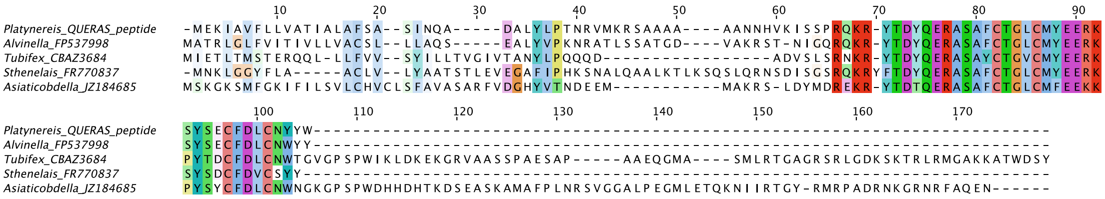

Multiple sequence alignment of annelid QERAS pNPs.

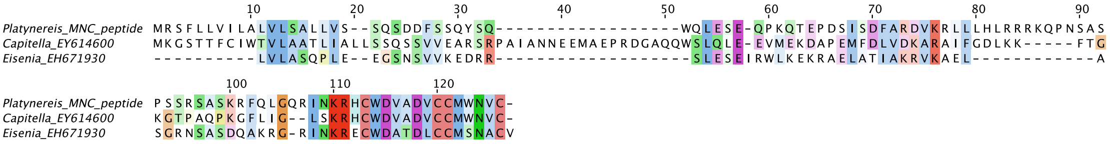

Multiple sequence alignment of annelid MNC pNPs.

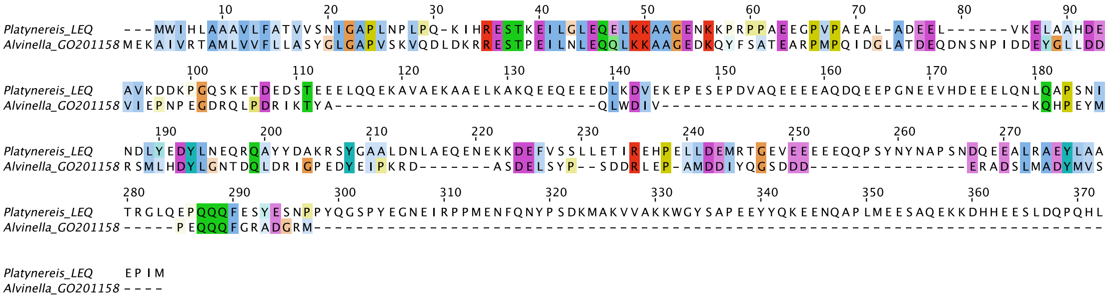

Multiple sequence alignment of *Platynereis* and *Alvinella pompejana* LEQ pNPs.
